# An *aza*-Robinson Annulation Strategy for the Synthesis of Fused Bicyclic Amides: Synthesis of (±)-Coniceine and Quinolizidine

**DOI:** 10.1021/acs.orglett.3c02798

**Published:** 2023-10-25

**Authors:** Alexander Garay-Talero, Tales A. C. Goulart, Rafael D. C. Gallo, Roberto do C. Pinheiro, Catalina Hoyos-Orozco, Igor D. Jurberg, Diego Gamba-Sánchez

**Affiliations:** † Laboratory of Organic Synthesis, Bio and Organocatalysis, Chemistry Department, Universidad de los Andes, Cra 1 No. 18A-12 Q:305, 111711 Bogota, Colombia; ‡ Institute of Chemistry, State University of Campinas, Rua Monteiro Lobato 270, 13083-862 Campinas, SP, Brazil

## Abstract

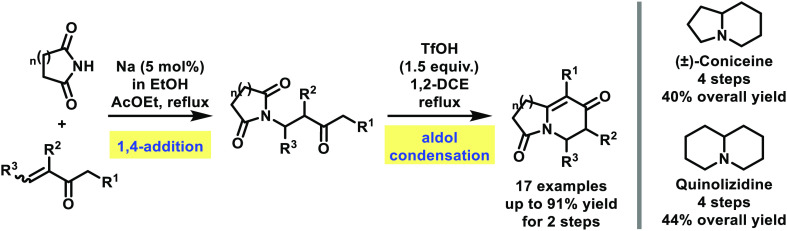

An *aza*-Robinson annulation strategy is described using a NaOEt-catalyzed conjugate addition of cyclic imides onto vinyl ketones, followed by a TfOH-mediated intramolecular aldol condensation to afford densely functionalized fused bicyclic amides. The potential use of these amides in the synthesis of alkaloids is demonstrated by the sequential conversion of appropriate precursors to (±)-coniceine and quinolizidine in two additional steps, thus allowing their preparation in overall 40 and 44% yields, respectively.

The Robinson annulation^1,2^ is a venerable reaction sequence that has been employed in the synthesis of several bicyclic diketones. Perhaps one of the most notable structures derived from this strategy is the Wieland-Miescher ketone,^3^ which has been employed in the preparation of several steroids^4^ (Scheme 1a). In contrast to the well-established methods allowing the construction of such fused bicyclic diketones, an analogous nitrogenated version of such a reaction, thus possibly allowing the synthesis of fused bicyclic amides **4** (which would be accessed via a similar mechanism, i.e., conjugate addition of cyclic imides **1** onto vinyl ketones **2**, followed by an intramolecular aldol condensation of the resulting intermediate **3**), has never been reported until the present date (Scheme 1b).^5,6^ Pioneering formal approaches toward *aza-*Robinson annulation*-type* products can be traced back to the work of Danishefsky^6a−6c^ and Heathcock,^6d,6e^ while further developments were also reported by Guarna^6f^ and Huang.^6g^ In this context, a more direct approach toward amides **4** remains highly desirable, because it would allow a more rapid construction of core structures commonly found in izidine alkaloids.^7^ Herein, we describe our results in this endeavor.

**Scheme 1:**
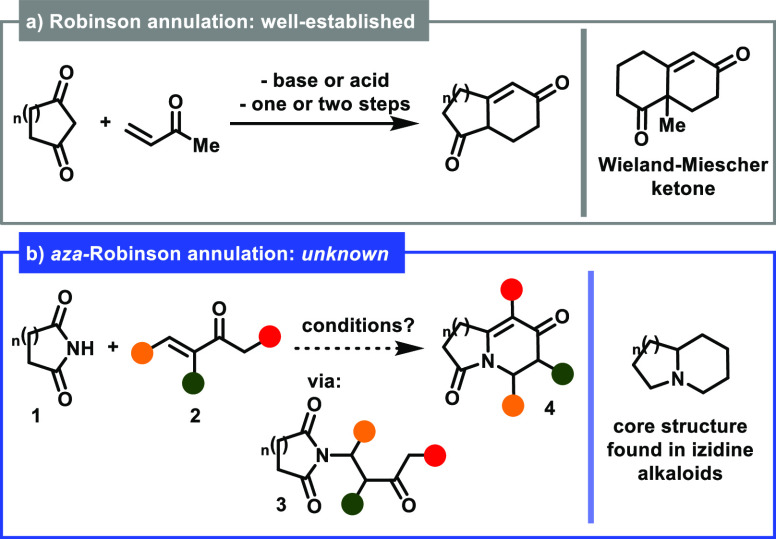
(a) Robinson Annulation: A Well-Established, Textbook Name Reaction. (b) An *aza-*Robinson Annulation Approach Using Cyclic Imides: An Unknown Transformation

Izidine alkaloids contain a fused bicyclic amine core of various ring sizes. Among them, the 5,6- and 6,6-bicycles (indolizidine and quinolizidine families, respectively) are the most well-known members found in nature.^8^ They can be obtained from plants,^9^ fungi,^10^ insects,^11^ and some animals, notably frogs.^12^ Several indolizidines and quinolizidines show important biological activities,^8a,8b,13^ such as antitumoral^14^ and antimicrobial^9a,15^ functions, and have been previously prepared in total synthesis campaigns.^16,17^ Among the numerous reported strategies leading to their bicyclic cores, a much smaller number of them have involved the formation of aldol condensation-*type* products,^18,19^ which have been accessed from the reaction of a nucleophilic functional group onto the carbonyl group of a cyclic imide moiety.

In this context, we started our investigations aiming at the development of an *aza*-Robinson annulation approach by evaluating each step of our two-step protocol starting from succinimide **1a** and methyl vinyl ketone (MVK) **2a**. The first step, an *aza*-Michael addition leading to adduct **3a**, could be performed with a catalytic amount of NaOEt (5 mol %) in EtOH/AcOEt at 77 °C,^20^ satisfactorily affording **3a** in 90% yield. (This conjugate addition does not proceed in the absence of the base.) This has set the stage for the optimization of the challenging and key intramolecular aldol condensation event aiming at the conversion of adduct **3a** to bicycle **4a**.

We started the screening of potential promoters for the desired cyclization in 1,2-DCE, at a 0.1 M concentration of the starting substrate **3a**, with the external heating temperature set to 83 °C for 16 h. The use of a stoichiometric amount of Sc(OTf)_3_ revealed a somehow promising 12% yield for the target compound **4a** (Entry 1, Table 1). The use of pyrrolidine or PTSA·H_2_O afforded only trace amounts of **4a** (Entries 2 and 3, Table 1). When moving to the use of the stronger acid TfOH, a significantly higher 42% yield for **4a** was reached in 1,2-DCE (Entry 4, Table 1), while further inquiry of reaction solvents (MeOH, MeCN, AcOEt, and toluene) afforded only lower conversions (Entries 5–8, Table 1). Finally, the use of 1.5 and 2 equiv of TfOH allowed a small increase to 55% yield of **4a** (Entries 9 and 10, Table 1). In all previous reactions, different amounts of the starting substrate **3a** could typically be observed remaining in the crude reaction mixture. When performing the reaction at a higher external temperature (ca. 100–110 °C), in order to more appropriately sustain the refluxing reaction, while also diluting it more, we were able to achieve an improved estimated yield of 83% and isolated yield of 80% for **4a** (Entry 11, Table 1). The reaction was also scaled up to 1 mmol, showing the same efficiency. Finally, we noticed that a one-step process was not feasible: (i) the use of a catalytic amount of NaOEt was required for the conjugate addition, thus producing the Michael adduct **3a** as the major or only product under the reaction conditions, without any trace of the bicycle **4a** being formed, and higher amounts of NaOEt led to decreased yields of **3a** (accompanied by degradation). (ii) Directly heating succinimide **1a** with MVK **2a** in the presence of TfOH at reflux of DCE did not produce any traces of **3a** or **4a**.

**Table 1:**
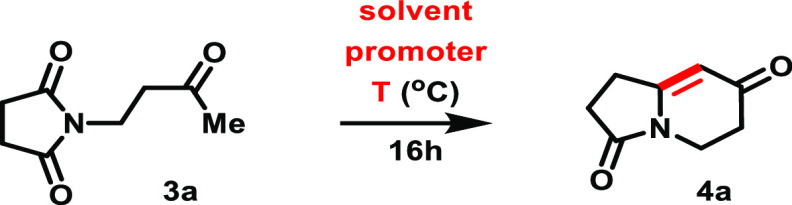
Optimization of the Reaction Conditions^a^

entry	solvent (0.1 M)	promoter	*T* (°C)^b^	yield **4a** (%)^c^
1	1,2-DCE	Sc(OTf)_3_ (1 equiv)	83	12%
2	1,2-DCE	pyrrolidine (1 equiv)	83	traces
3	1,2-DCE	PTSA·H_2_O (1 equiv)	83	traces
4	1,2-DCE	TfOH (1 equiv)	83	42%
5	MeOH	TfOH (1 equiv)	65	n.r.
6	MeCN	TfOH (1 equiv)	82	35%
7	AcOEt	TfOH (1 equiv)	77	20%
8	Toluene	TfOH (1 equiv)	90	27%
9	1,2-DCE	TfOH (1.5 equiv)	83	55%
10	1,2-DCE	TfOH (2 equiv)	83	55%
**11**	**1,2-DCE (0.04 M)**	**TfOH (1.5 equiv)**	**100**	**83%/80%**^d^

a Reactions performed using 0.2 mmol of **3a**.

b External temperature.

c Estimated yields based on ^1^H NMR analysis of the reaction crude using 1,3,5-trimethoxybenzene as internal standard.

d Isolated yield. n.r.: no reaction.

With the optimal reaction conditions in hand, we became interested in evaluating the scope of possible bicyclic amides **4** that could be accessed by this two-step strategy (Scheme 2). At the outset, we evaluated different cyclic imides **1** in the presence of MVK **2a**. Glutarimide **1b** and 4-phenylpiperidine-2,6-dione **1c** could be engaged in our protocol to afford the corresponding cyclized compounds **4b** and **4c** in 66 and 50% combined yields, respectively. 1,8-Naphthalimide **1d** could be added to MVK **2a** in a poor 15% yield (30% brsm), with this low efficiency possibly being attributed to its low solubility in the reaction medium. Notwithstanding, the corresponding intramolecular aldol condensation leading to product **4d** could be successfully performed in a 58% yield. Phthalimides **1e**–**1h** afforded their corresponding cyclized products, in a range of 53–91% yields for two steps. However, perhaps unsurprisingly, substituted phthalimides **1f**, **1g**, and **1h** led to mixtures of regioisomers **4f** + **4f′** (1:1 r.r.), **4g** + **4g′** (6.7:1 r.r., but major compound **4g** could be isolated), and **4h** + **4h′** (2.6:1 r.r.). The assignment of the regiosiomers was based on NOESY experiments (see the Supporting Information for more details). The use of *cis*-hexahydro-1*H*-isoindole-1,3(2*H*)-dione **1i** was equally high yielding, thus allowing the preparation of tricycle **4i** in a combined 78% yield. Then, we moved forward to evaluate different vinyl ketones **2**. The use of pent-1-en-3-one **2b** with succinimide **1a**, glutarimide **1b**, and phthalimide **1e** afforded the corresponding cyclized compounds **4j** (45%, 2 steps), **4k** (49%, 2 steps), and **4l** (59%, 2 steps) with similar efficiencies. The reaction sequence applied to oct-1-en-3-one **2c** and succinimide **1a** led to the successful preparation of bicycle **4m** in 49% yield for two steps.

**Scheme 2:**
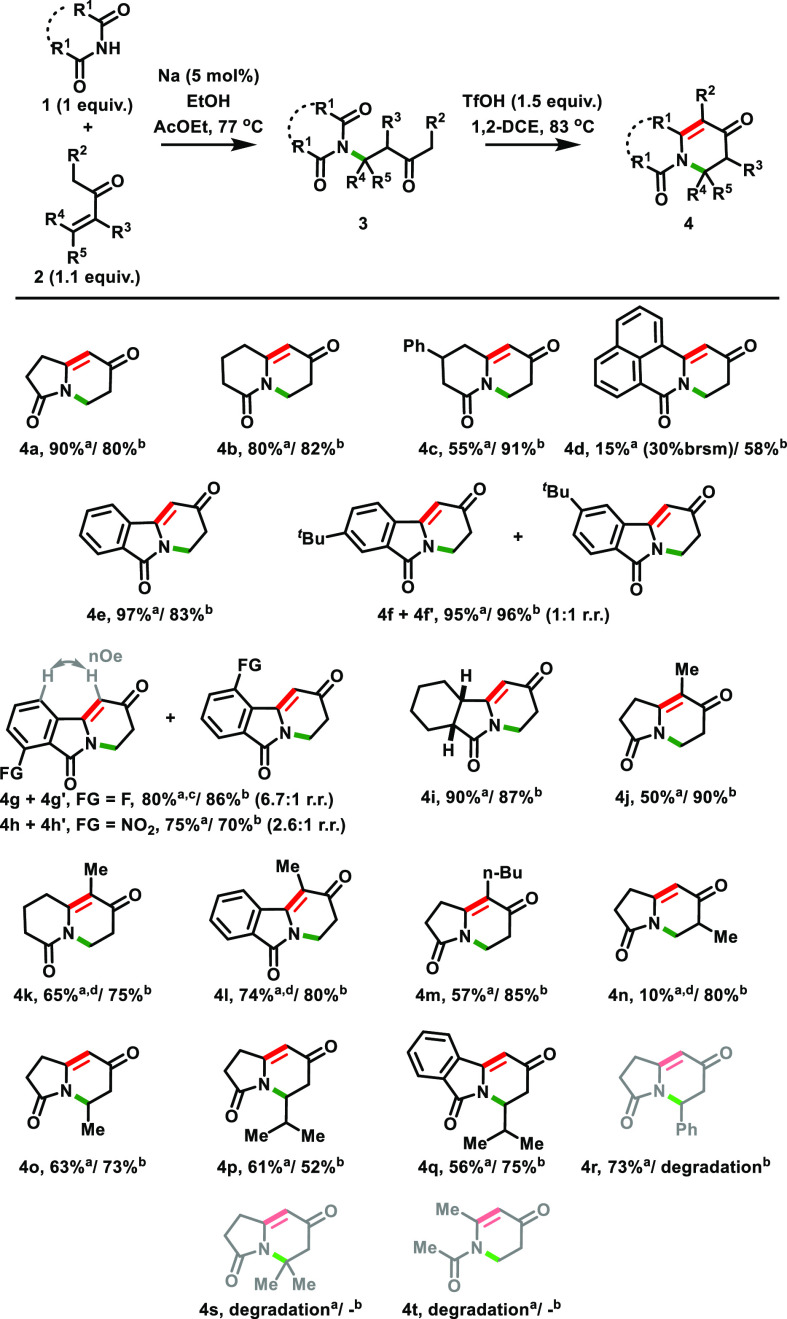
Scope for the *aza*-Robinson Annulation Result of the first step. Result of the second step. Yields correspond to isolated materials. Performed with 2.3 equiv of MVK **2a**. Performed with 1.4 equiv of the vinyl ketone **2**.

Disappointingly, the *aza*-Michael addition between 3-methylbut-3-en-2-one **2d** and succinimide **1a** led to the isolation of the corresponding adduct **3n** in a very poor 10% yield (the estimated yield based on the use of an internal reference was 16%; see the Supporting Information). However, the subsequent aldol condensation step proceeded uneventfully to afford **4n** in 80% yield. Finally, this two-step protocol was also evaluated using pent-3-en-2-one **2e** and 5-methylhex-3-en-2-one **2f** in the presence of succinimide **1a** to produce bicycles **4o** (46%, 2 steps) and **4p** (32%, 2 steps), respectively, while the use of phthalimide **1e** with 5-methylhex-3-en-2-one **2f** led to compound **4q** in 42% yield for two steps (Scheme 2). Remarkable limitations of this method were found when attempting the preparation of bicycles **4r** and **4s** and amide **4t**. The conjugate addition between succinimide **1a** and 4-phenylbut-3-en-2-one **2g** proceeded well in 73% yield, but the TfOH-mediated aldol condensation led to a complex mixture. We speculate that a possible reason for this is that the Ph ring is also appropriately placed to compete with the enol moiety as a nucleophile,^19a,19g^ thus attacking the imide carbonyl group. In the cases of the use of 4-methylpent-3-en-2-one **2h** (aiming at the synthesis of **4s**) or *N*-acetylacetamide **1j** (aiming at the synthesis of **4t**), the initial conjugate addition did not work (with degradation being observed), and the second step could not be evaluated (Scheme 2).

Then, we became also interested in evaluating the use of aldehydes and acetals in our TfOH-mediated aldol condensation strategy, as they could possibly lead to short routes to some izidine alkaloids, such as (±)-tashiromine and (±)-epilupinine. (Preliminary evaluation of ketals **5a** and **5b**, derived from ketones **3a** and **3b**, respectively, has shown only poor or moderate conversions to **4a** and **4b**, respectively. See the Supporting Information.)

In this context, we designed and evaluated their synthesis starting by a Mitsunobu reaction of the appropriate cyclic imide (succinimide **1a** or glutarimide **1b**) with hex-5-en-1-ol **6**, which was successfully performed to afford *N-*alkylated imides **7a** (71%) and **7b** (65%).^21^ Ozonolysis of these compounds afforded aldehydes **8a** (87%) and **8b** (48%), respectively.

At this point, we attempted the key cyclization event by using different amounts of TfOH and reaction temperatures, thus aiming at the preparation of putative intermediates **10a** and **10b**,^22^ which could be eventually further reduced by LiAlH_4_ to afford the targeted compounds (±)-tashiromine **11a** and (±)-epilupinine **11b**. However, in none of our attempts have we been able to observe intermediates **10a** or **10b**, and even further reduction attempts did not reveal any traces of the desired compounds **11a** or **11b**. In this pursuit, we have also transformed aldehydes **8a** and **8b** in the corresponding acetals **9a** and **9b**, respectively; new cyclization attempts toward **10a** and **10b** were investigated by using various amounts of TfOH and reaction temperatures, but again, they were all unsuccessful (Scheme 3).

**Scheme 3:**
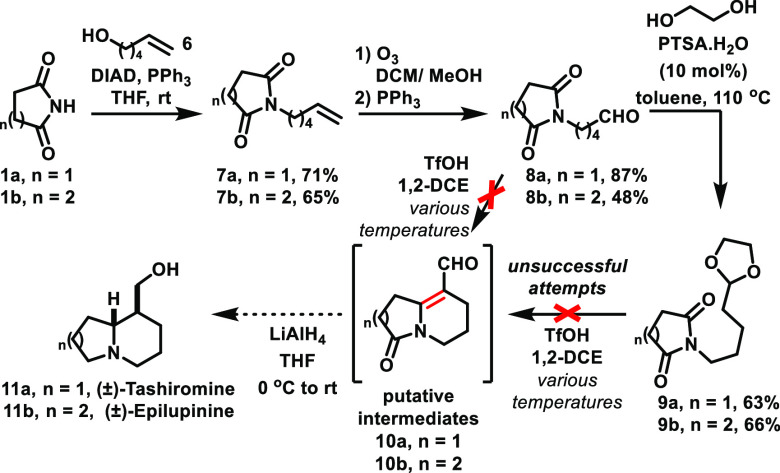
Evaluation of TfOH-Mediated Intramolecular Aldol Condensation Employing Aldehydes and Acetals. Attempts toward the Synthesis of (±)-Tashiromine and (±)-Epilupinine

Although our established intramolecular TfOH-mediated aldol condensation failed for the cyclization of aldehydes and acetals, we imagined that the successful protocol for ketones could be useful for the synthesis of a number of izidine alkaloids. In this context, our preliminary investigations allowed us to gain rapid access to (±)-coniceine **13a** and quinolizidine **13b**. Indeed, further hydrogenation of bicycles **4a** and **4b** directly afforded amides **12a** and **12b**, respectively, both in 70% yields. This full reduction was somehow surprising.^23^ Currently, we tentatively attribute this selectivity to the (stereo)electronics of the highly conjugated systems within **4a** and **4b**. In the case of **4a**, no other compound was observed. In the case of **4b**, the ketone **12b′** could be isolated in 10% yield (see the Supporting Information). Then, subsequent reduction of amides **12a** and **12b** with LiAlH_4_ readily led to target compounds **13a** (79%) and **13b** (96%) (Scheme 4a). In contrast to the observed selectivity for the hydrogenations of **4a** and **4b**, the hydrogenation of tricycle **4e** under the same previously employed conditions mostly afforded the corresponding ketone **14** (accompanied by the formation of trace amounts of desired **12c** and another compound, which can be tentatively assigned as the corresponding alcohol, not shown), thus revealing that compound **4e** is significantly more reluctant to undergo the previously observed full reduction. As a consequence, an additional Wolff–Kishner reduction step was required to produce the corresponding amide **12c**, which could be performed in 39% yield for a telescoped two-step sequence (Scheme 4b).

**Scheme 4:**
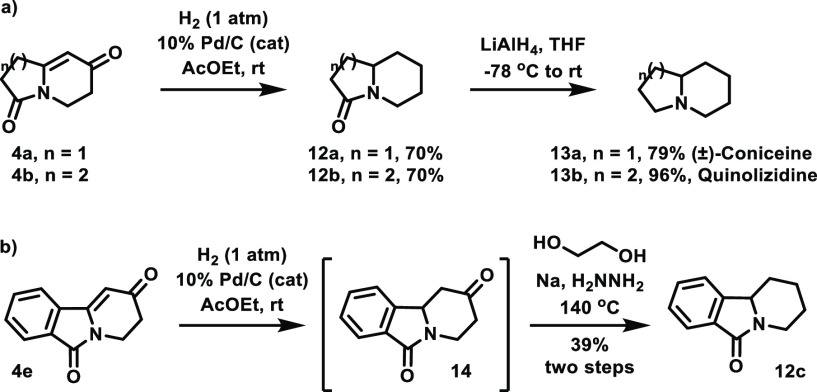
(a) Synthesis of (±)-Coniceine and Quinolizidine. (b) Reduction Sequence Allowing the Conversion of **4e** to Tricycle **12c**

In summary, a new two-step *aza*-Robinson annulation strategy was developed taking advantage of the simple combination of cyclic imides with vinyl ketones to gain access to densely functionalized bicyclic amides that can be possibly useful in the synthesis of alkaloids. The key step of this protocol is a TfOH-promoted intramolecular aldol condensation reaction between imide carbonyls and transiently formed enol moieties derived from ketones. While the preliminary use of ketals showed to be limited, attempts using aldehyde or acetal fragments were unsuccessful. Finally, we have successfully applied our new protocol for the synthesis of alkaloids (±)-coniceine and quinolizidine, both being accessed in only four steps and overall yields of 40 and 44%, respectively. Furthermore, we have also disclosed an unusual remarkable selectivity for the hydrogenation of enaminone systems present in **4a** and **4b**, thus rapidly leading to the corresponding fully saturated amides.

## Data Availability

The data underlying this study are available in the published article and its Supporting Information.

## Supporting Information Available

Experimental procedures, characterization data, and ^1^H, ^13^C, and ^19^F NMR spectra of all compounds (PDF)
FAIR data, including the primary NMR FID files, for compounds **3a**−**3r**, **4a**−**4q**, **5a**, **5b**, **7a**, **7b**, **8a**, **8b**, **9a**, **9b**, **12a**, **12b**, **12b′**, **13a**, and **13b** (ZIP)
