# Supplementary material for: An aza-Robinson Annulation Strategy for the Synthesis of Fused Bicyclic Amides: Synthesis of (±)-Coniceine and Quinolizidine
Source: Org Lett. 2023 Oct 25;25(43):7940–5. doi: 10.1021/acs.orglett.3c02798 (PMC10630962; doi:10.1021/acs.orglett.3c02798)

Supporting Information for:

An *aza*-Robinson Annulation Strategy for the Synthesis of Fused Bicyclic Amides: Synthesis of (±)-Coniceine and Quinolizidine

Alexander Garay-Talero,[a] Tales A. C. Goulart,[b†] Rafael D. C. Gallo,[b†] Roberto do C. Pinheiro,[b] Catalina Hoyos-Orozco,[a] Igor D. Jurberg,*[b] Diego Gamba-Sánchez*[a]

[a] Laboratory of Organic Synthesis, Bio and Organocatalysis, Chemistry Department, Universidad de los Andes, Cra 1 No. 18A-12 Q:305. 111711, Bogota, Colombia

[b] Institute of Chemistry, State University of Campinas, Rua Monteiro Lobato 270, 13083-862, Campinas, SP, Brazil

[†]Equal contributions

Table of Contents

1. General information S3

2. Optimization of the Intramolecular Aldol Condensation S4

3. Experimental Procedures and Characterization of Compounds S6

4. NMR Spectra S34

1. General Information

All reactions were conducted in oven-dried glassware under an inert atmosphere of dry nitrogen. All reagents were used as received from commercial suppliers, unless otherwise stated. Reactions that required heating were performed using a heating mantle or appropriate heating blocks depending on the reaction flask size. All solvents were distilled from appropriate drying agents prior to use. ^{1}H NMR spectra were recorded at 250 or 400 MHz on a Bruker AV-250 or AV-400 spectrometers, respectively. ^{13}C NMR spectra were recorded at 62.5 or 101 MHz on a 250 or 400 MHz on a Bruker AV-250 or AV-400 spectrometers, respectively. ^{19}F NMR spectra were recorded at 376.5 MHz. NMR spectra were recorded in deuterated chloroform ($CDCl_3$) solutions, chemical shifts are given in parts per million (ppm, δ), referenced to the TMS, solvent peak of $CDCl_3$ defined at δ = 7.26 ppm (^{1}H NMR) and δ = 77.00 (^{13}C NMR). Trifluoroacetic acid was used as a reference for ^{19}F NMR, δ = -75.39 ppm . Data abbreviations are reported as follows: δ, chemical shift; s, singlet; d, doublet; t, triplet; q, quartet and m, multiplet. Coupling constants are quoted in Hertz (*J*). Infrared (IR) spectra were collected on an Agilent Cary 630 FTIR, using a diamond ATR sensor or Thermo Nicolet-Nexus FTIR with Pike Miracle ATR cell spectrometers, and are reported in terms of absorption frequency (n, cm^{-1}). Mass spectrometric determinations were carried out on a Thermo Scientific LTQ FT Ultra Q Exactive Orbitrap or Agilent 5973 spectrometers, working with an electron spray ionization (ESI). Reactions were monitored by thin layer chromatography (TLC) on Silica gel 60 F_{254} aluminium plates. TLC plates were visualized using ultraviolet (UV) light at 254 nm or stained with anisaldehyde, vanillin or $KMnO_4$ solutions. Flash column chromatography was performed with silica gel 60 Å (230 – 400 mesh) from Aldrich, using the stated mixture of solvents. Melting points were measured on pure solids using a 1101D-MEL-TEMP melting point apparatus.

2. Optimization of the Intramolecular Aldol Condensation

Table S1. Optimization of Reaction Conditions based on the Conversion of **3a** to **4a**.

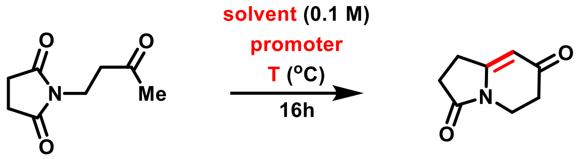

3a (0.2 mmol) 4a

entry	Solvent (0.1 M)	promoter	T (°C)	yield of 4a (%)[a]
1	1,2-DCE	-	83	< 5% (+ >90% **3a**)
2	1,2-DCE	$Sc(OTf)_3$ (1 equiv.)	83	12% (+ 85% **3a**)
3	1,2-DCE	$BF_3.OEt_2$ (1 equiv.)	83	< 5% (+ >90% **3a**)
4	1,2-DCE	$SnCl_4$ (1 equiv.)	83	< 5% (+ >90% **3a**)
5	1,2-DCE	pTSA·H_2O (1 equiv.)	83	< 5% (+ >90% **3a**)
6	1,2-DCE	TfOH (1 equiv.)	83	42% (+ 30% **3a**)
7	1,2-DCE	Aniline (1 equiv.)	83	< 5% (+ >90% **3a**)
8	1,2-DCE	Benzylamine (1 equiv.)	83	< 5% (+ 80% **3a**)
9	1,2-DCE	Piperidine (1 equiv.)	83	< 5% (+ >90% **3a**)
10	1,2-DCE	Morpholine (1 equiv.)	83	< 5% (+ >90% **3a**)
11	1,2-DCE	Pyrrolidine (1 equiv.)	83	< 5% (+ >90% **3a**)
12	1,2-DCE	KOH (1 equiv.)	83	< 5% (+ 80% **3a**)
13	1,2-DCE	4-DMAP (1 equiv.)	83	< 5% (+ >90% **3a**)
14	H_2O	TfOH (1 equiv.)	100	< 5% (+ >97% **3a**)
15	MeOH	TfOH (1 equiv.)	65	< 5% (+ 57% **3a**)
16	DMF	TfOH (1 equiv.)	153	< 5% (+ 0% **3a**)
17	MeCN	TfOH (1 equiv.)	82	35% (+ 50% **3a**)
18	Acetone	TfOH (1 equiv.)	56	< 5% (**3a** remaining, messy)
19	THF	TfOH (1 equiv.)	65	< 5% (+ 100% **3a**)
20	AcOEt	TfOH (1 equiv.)	77	20% (+ 66% **3a**)
21	DCM	TfOH (1 equiv.)	40	< 5% (+ 90% **3a**)
22	Et_2O	TfOH (1 equiv.)	34	< 5% (+ 95% **3a**)
23	Hexane	TfOH (1 equiv.)	69	7% (+ 12% **3a**)
24	Toluene	TfOH (1 equiv.)	90	27% (+ 15% **3a**)
25	Toluene	TfOH (0.1 equiv.)	90	5% (+ 70% **3a**)
26	Toluene	TfOH (0.2 equiv.)	90	10% (+ 70% **3a**)
27	Toluene	TfOH (0.4 equiv.)	90	15% (+ 44% **3a**)
28	Toluene	TfOH (1.5 equiv.)	90	22% (+ 10% **3a**)
29	Toluene	TfOH (2 equiv.)	90	23% (+ 5% **3a**)
30	1,2-DCE	TfOH (1.5 equiv.)	83	55% (+20% **3a**)

31	1,2-DCE	TfOH (2 equiv.)	83	55% (+20% **3a**)
**32**	**1,2-DCE (0.04M)**	**TfOH (1.5 equiv.)**	**100**	**83% (+12% 3a)**

[a]Estimated based on the ^1H NMR analysis of the crude reaction mixture using 1,3,5-trimethoxybenzene as internal reference.

As in entry 30, scaling-up to 1 mmol - effect of concentration (yields estimated using 1,3,5-trimethoxybenzene):

i) 1,2-DCE (0.25M) – 23% **14** + 23% **3a**
ii) 1,2-DCE (0.1 M) – 35% **14** + 28% **3a**
iii) 1,2-DCE (0.04 M) – 55% **14** + 30% **3a** → **isolated yield of 4a**: **51%**

<u>Important observation:</u> All previous reactions of our optimization studies were performed in 4-mL vials being heated in an aluminum block using 0.2-mmol of the starting ketone **3a** with the external temperature heating the aluminum block set as indicated in Table 1. When moving forward to increasing the reaction scale to 1-mmol, we used a round-bottom flask equipped with a reflux condenser, while heating with an oil bath. We observed that in 16h of reaction, *<u>the conversion toward the target product **4a** was improved from 55% to 83%</u>* (both estimated yields by ^1H NMR of crude reaction mixture using 1,3,5-trimethoxy benzene as internal standard) when setting the external temperature high enough (100 - 110 °C) to make sure that the internal solution of the flask would be under intense reflux during the whole transformation (Table S1, Entry 32).

2.1 Preliminary Evaluation of TfOH-Mediated Intramolecular Aldol Condensation Employing Ketals 5a and 5b.

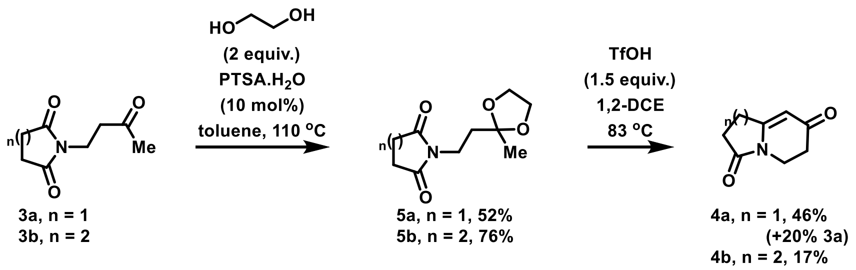

Reactions worked in limited extension. Apparently, in the presence of TfOH, ketals **5a** and **5b** undergo hydrolysis back to corresponding ketones (**3a** and **3b**, respectively), which cyclize to amides **4a** and **4b**, respectively.

3. Experimental Procedures and Characterization of Compounds

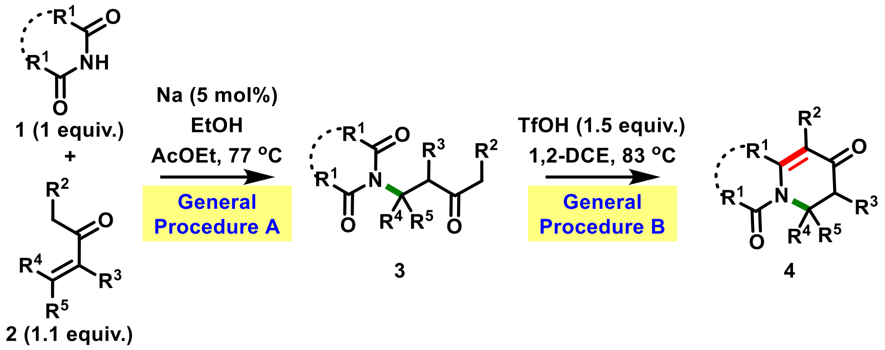

General Procedure A: The procedure employed here was originally reported by Rasapalli and co-workers.[1] Under N_2, at room temperature, Na (0.05 equiv.) is added to anhydrous EtOH (0.2 mL/ mmol of **1**). After *ca.* 15 min, when the Na is completely dissolved, the resulting solution of NaOEt in EtOH is fully transferred using a syringe to a round bottom flask containing imide **1** (1 equiv.) in anhydrous AcOEt (0.9 M in relation to **1**, dried in 3Å MS) at room temperature, under N_2. The resulting solution is stirred at room temperature for additional 5 min; then vinyl ketone **2** (1.1 equiv.) is added. Then, the resulting mixture is stirred and heated at reflux (external temperature ~ 90 °C) overnight. At this point, the reaction mixture is cooled to room temperature and water is added. The aqueous phase is extracted with AcOEt (3x). The combined organic phases are dried ($MgSO_4$), filtered and concentrated under reduced pressure. Purification by flash column chromatography (SiO_2) affords the title product in the state yield.

General Procedure B: At room temperature, under air, a round bottom flask equipped with a reflux condenser is charged with ketone **3** (1 equiv.) and 1,2-DCE (0.04 M in relation to **3**). Then, TfOH (1.5 equiv.) is added, and the mixture is heated at reflux (external temperature ~100 °C) and the reaction is accompanied by TLC. Upon reaction completion, the reaction temperature is allowed to cool down to room temperature and the reaction is quenched with an aqueous saturated solution of $NaHCO_3$. The resulting mixture is extracted with DCM (3x), dried ($MgSO_4$) and concentrated under reduced pressure. Purification by flash column chromatography (SiO_2) affords the title compound in the stated yield.

[1] Rasapalli, S.; Kumbam, V.; Dhawane, A. N.; Golen, J. A.; Lovely, C. J.; Rheingold, A. L., *Org. Biomol. Chem.* **2013,** *11*, 4133-4137.

1-(3-oxobutyl)pyrrolidine-2,5-dione (3a)

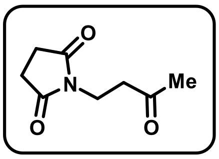

General Procedure A is employed with Na (6 mg, 0.25 mmol, 0.05 equiv.), anhydrous EtOH (1 mL), succinimide **1a** (495 mg, 5 mmol, 1 equiv.) in anhydrous AcOEt (5.5 mL, 0.9 M in relation to **1a**, dried in 3Å MS) and MVK **2a** (90% grade, 500 μL, 5.5 mmol, 1.1 equiv.). Reaction time 12h. Purification by flash column chromatography (SiO_2, gradient: Hex – 8:2 Hex:AcOEt – 1:1 Hex:AcOEt – 7:3 AcOEt:Hex) affords the title product as a pale yellow oil, that solidifies in the freezer producing a white solid: 760 mg, 90%.[2]

Alternative procedure: To a solution of succinimide **1a** (990 mg, 10 mmol, 1 equiv.) and 4-DMAP (61 mg, 0.5 mmol, 0.05 equiv.) in water (7 mL, 1.4 M in relation to **1a**), MVK **2a** (90% grade, 1.36 mL, 15 mmol, 1.5 equiv.) is added. The resulting solution is vigorously stirred at room temperature. Reaction time 12h. Then, the reaction mixture is extracted with AcOEt (3x), dried ($MgSO_4$), filtered and concentrated under reduced pressure. Purification by flash column chromatography (SiO_2, gradient: Hex – 8:2 Hex:AcOEt – 1:1 Hex:AcOEt – 7:3 AcOEt:Hex) affords the title product as a pale yellow oil, that solidifies in the freezer producing a white solid: 1.52 g, 90%.

^{1}H NMR (250 MHz, $CDCl_3$) δ: 3.73 (t, *J* = 7.4 Hz, 2H), 2.72 (t, *J* = 7.4 Hz, 2H), 2.67 (s, 4H), 2.13 (s, 3H).
^{13}C{^{1}H} NMR (62.5 MHz, $CDCl_3$) δ: 205.7, 176.9, 40.5, 33.7, 29.8, 28.0.
M.P.: 48 - 50 ^{o}C.
IR (neat, ATR): 2965, 2928, 1773, 1683, 1423, 1367, 1342, 1160 cm^{-1}.
HRMS (ESI-ToF) m/z: $[M + H]^+$ Calcd. for $C_8H_{12}NO_3$: 170.0812, found: 170.0812.

1,2,5,6-tetrahydroindolizine-3,7-dione (4a)

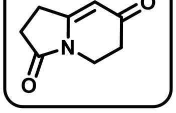

General Procedure B is employed with 1-(3-oxobutyl)pyrrolidine-2,5-dione **3a** (169 mg, 1 mmol, 1 equiv.), 1,2-DCE (25 mL, 0.04 M in relation to **3a**) and TfOH (135 μL, 1.5 mmol, 1.5 equiv.). Reaction time: 16h. Purification by flash column

[2] ^{1}H NMR is in good agreement with the literature. See: Lochead, A. W.; Proctor, G. R.; *J. Chem. Soc., Perkin Trans. 1*, **1984**, 2477-2489.

chromatography (SiO_2, gradient: 8:2 Hex:AcOEt – 1:1 Hex:AcOEt – AcOEt) affords the title compound as a pale yellow solid: 120 mg, 80%.[3]

^{1}H NMR (250 MHz, $CDCl_3$) δ: 5.32 (s, 1H), 3.88 (t, *J* = 7.7 Hz, 2H), 2.93 – 2.87 (m, 2H), 2.65 – 2.59 (m, 2H), 2.53 (t, *J* = 7.7 Hz, 2H).

^{13}C{^{1}H} NMR (62.5 MHz, $CDCl_3$) δ: 192.2, 174.7, 161.6, 102.7, 37.8, 33.8, 28.0, 23.6.

M.P.: 70 - 72 °C.

IR (neat, ATR): 3071, 2941, 1724, 1648, 1592, 1335, 1212, 1147 cm^{-1}.

HRMS (ESI-ToF) m/z: [M + H]$^+$ Calcd. for $C_8H_{10}NO_2$: 152.0706, found: 152.0706.

1-(3-oxobutyl)piperidine-2,6-dione (3b)

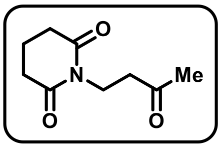

General Procedure A is employed with Na (6 mg, 0.25 mmol, 0.05 equiv.), anhydrous EtOH (1 mL), glutarimide **1b** (565 mg, 5 mmol, 1 equiv.) in anhydrous AcOEt (5.5 mL, 0.9 M in relation to **1b**, dried in 3Å MS) and MVK **2a** (90% grade, 500 μL, 5.5 mmol, 1.1 equiv.). Reaction time 12h. Purification by flash column chromatography (SiO_2, gradient: Hex – 8:2 Hex:AcOEt – 1:1 Hex:AcOEt – 7:3 AcOEt:Hex) affords the title product as a pale yellow oil, that solidifies in the freezer producing a colorless solid: 732 mg, 80% (contaminated with a small amount of glutarimide **1b**).

^{1}H NMR (250 MHz, $CDCl_3$) δ: 3.93 (t, *J* = 7.2 Hz, 2H), 2.60 – 2.54 (m, 6H), 2.07 (s, 3H), 1.91 – 1.81 (m, 2H).

^{13}C{^{1}H} NMR (62.5 MHz, $CDCl_3$) δ: 206.5, 172.2, 41.3, 34.6, 32.5, 29.7, 16.8.

M.P.: 38 - 40 ^{o}C.

IR (neat, ATR): 2978, 1712, 1674, 1354, 1168, 1138 cm^{-1}.

HRMS (ESI-ToF) m/z: [M + H]$^+$ Calcd. for $C_9H_{14}NO_3$: 184.0968, found 184.0968.

3,4,8,9-tetrahydro-2H-quinolizine-2,6(7H)-dione (4b)

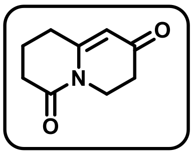

General Procedure B is employed with 1-(3-oxobutyl)piperidine-2,6-dione **3b** (183 mg, 1 mmol, 1 equiv.), 1,2-DCE (25 mL, 0.04 M in relation to **3b**) and TfOH (135 μL, 1.5 mmol, 1.5 equiv.). Reaction time: 16h. Purification by flash column

[3] ^{1}H and ^{13}C NMRs are in good agreement with the literature. See: Flitsch, W.; Pandl, K., *Liebigs Ann. Chem.*, **1987,** *1987*, 649-654.

chromatography (SiO_2, gradient: 8:2 Hex:AcOEt – 1:1 Hex:AcOEt – AcOEt) affords the title compound as a white solid: 135 mg, 82%.[4]

^{1}H NMR (250 MHz, $CDCl_3$) δ: 5.23 (s, 1H), 4.11 (t, *J* = 7.1 Hz, 2H), 2.63 (t, *J* = 6.2 Hz, 4H), 2.49 (t, *J* = 7.1 Hz, 2H), 1.98 – 1.88 (m, 2H).

^{13}C{^{1}H} NMR (62.5 MHz, $CDCl_3$) δ: 193.3, 168.9, 156.3, 106.7, 40.3, 35.4, 32.9, 29.7, 18.9.

M.P.: 95 – 97 °C.

IR (neat, ATR): 3060, 2960, 1695, 1660, 1650, 1590 cm^{-1}.

HRMS (ESI-ToF) m/z: [M + H]$^+$ Calcd. for $C_9H_{12}NO_2$: 166.0863, found: 166.0863.

1-(3-oxobutyl)-4-phenylpiperidine-2,6-dione (3c)

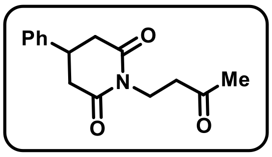

General Procedure A is employed with Na (2.4 mg, 0.1 mmol, 0.05 equiv.), anhydrous EtOH (0.4 mL), 4-phenylpiperidine-2,6-dione **1c** (400 mg, 2.12 mmol, 1 equiv.) in anhydrous AcOEt (2.4 mL, 0.9 M in relation to **1c**, dried in 3Å MS) and MVK **2a** (99% grade, 195 μL, 2.3 mmol, 1.1 equiv.). Reaction time 12 h. Purification by flash column chromatography (SiO_2, gradient: 1:1 DCM:*c*-Hex – 2:1 DCM:*c*-Hex – DCM) affords the title product as a white solid: 300 mg, 55%.

^{1}H NMR (400 MHz, $CDCl_3$) δ: 7.38 – 7.35 (m, 2H), 7.31 – 7.27 (m, 1H), 7.20 – 7.18 (m, 2H), 4.07 (t, *J* = 7.6 Hz, 2H), 3.39 – 3.31 (m, 1H), 2.99 (dd, *J* = 17.2 Hz, *J* = 4.4 Hz, 2H), 2.79 (dd, *J* = 17.2 Hz, *J* = 12.0 Hz, 2H), 2.68 (t, *J* = 7.2 Hz, 2H), 2.17 (s, 3H).

^{13}C{^{1}H} NMR (100 MHz, $CDCl_3$) δ: 206.6, 171.6, 140.5, 129.1, 127.6, 126.3, 41.4, 39.8, 35.1, 34.6, 29.9.

M.P.: 158 - 159 °C.

IR (neat, ATR): 1713, 1667, 1343, 1269, 1196, 1142 cm^{-1}.

HRMS (ESI-ToF) m/z: [M + H]$^+$ Calcd for $C_{15}H_{18}NO_3$: 260.1281, found: 260.1281.

[4] ^{1}H and ^{13}C NMRs are in good agreement with the literature. See: Goti, A.; Brandi, A.; Danza, G.; Guarna, A.; Donati, D.; De Sarlo, F., *J. Chem. Soc., Perkin Trans. 1,* **1989**, 1253-1258.

8-phenyl-3,4,8,9-tetrahydro-2H-quinolizine-2,6(7H)-dione (4c)

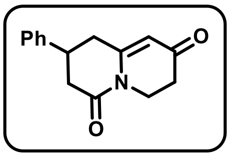

General Procedure B is employed with 1-(3-oxobutyl)-4-phenylpiperidine-2,6-dione **3c** (130 mg, 0.5 mmol, 1 equiv.), 1,2-DCE (12.5 mL, 0.04 M in relation to **3c**) and TfOH (66 µL, 0.75 mmol, 1.5 equiv.). Reaction time: 16 h. Purification by flash column chromatography (SiO_2, gradient: DCM – 10:1 DCM:AcOEt – 8:1 DCM:AcOEt) affords the title compound as a white solid: 110 mg, 91%.

^{1}H NMR (400 MHz, $CDCl_3$) δ: 7.39 – 7.34 (m, 2H), 7.31 – 7.27 (m, 1H), 7.22 – 7.20 (m, 2H), 5.29 (s, 1H), 4.35 – 4.28 (m, 1H), 4.08 – 4.01 (m, 1H), 3.35 – 3.27 (m, 1H), 3.01 – 2.95 (m, 1H), 2.91 – 2.85 (m, 2H), 2.77 (dd, *J* = 17.2 Hz, *J* = 11.6 Hz, 1H), 2.62 – 2.48 (m, 2H).
^{13}C{^{1}H} NMR (100 MHz, $CDCl_3$) δ: 193.3, 168.6, 155.1, 140.9, 129.0, 127.6, 126.3, 107.3, 40.4, 39.9, 36.9, 36.2, 35.4.
M.P.: 112 - 113°C.
IR (neat, ATR): 3070, 1686, 1655, 1582, 1408, 1342, 1234, 1160 cm^{-1}.
HRMS (ESI-ToF) m/z: [M + H]$^+$ Calcd. for $C_{15}H_{16}NO_2$: 242.1176, found: 242.1175.

2-(3-oxobutyl)-1H-benzo[de]isoquinoline-1,3(2H)-dione (3d)

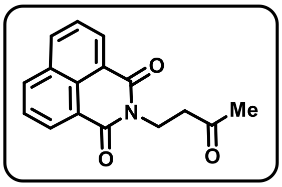

General Procedure A is employed with Na (6 mg, 0.25 mmol, 0.05 equiv.), anhydrous EtOH (1 mL), 1,8-naphthalimide **1d** (985 mg, 5 mmol, 1 equiv.) in anhydrous AcOEt (5.5 mL, 0.9 M in relation to **1d**, dried in 3Å MS) and MVK **2a** (99% grade, 460 µL, 5.5 mmol, 1.1 equiv.). Reaction time 12 h. Purification by flash column chromatography (SiO_2, gradient: 1:1 DCM:*c*-Hex – DCM) affords the title product as a white solid: 200 mg, 15% (30% brsm).

^{1}H NMR (400 MHz, $CDCl_3$) δ: 8.58 (dd, *J* = 7.4 Hz, *J* = 1.2 Hz, 2H), 8.21 (dd, *J* = 8.2 Hz, *J* = 1.2 Hz, 2H), 7.73 (dd, *J* = 8.2Hz, *J* = 7.4 Hz, 2H), 4.45 (t, *J* = 7.6 Hz, 2H), 2.90 (t, *J* = 7.6 Hz, 2H), 2.21 (s, 3H).
^{13}C{^{1}H} NMR (100 MHz, $CDCl_3$) δ: 206.6, 164.0, 134.1, 131.6, 131.3, 128.1, 126.9, 122.4, 41.6, 35.6, 29.9.
M.P.: 154 - 155 ^{o}C.
IR (neat, ATR): 3083, 1697, 1655, 1585, 1331, 1230, 1146, 1038 cm^{-1}.
HRMS (ESI-ToF) m/z: [M + H]$^+$ Calcd. for $C_{16}H_{14}NO_3$: 268.0968, found: 268.0967.

9,10-dihydro-7H,11H-benzo[de]pyrido[2,1-a]isoquinoline-7,11-dione (4d)

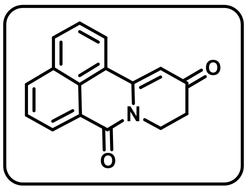

General Procedure B is employed with 2-(3-oxobutyl)-1H-benzo[de]isoquinoline-1,3(2H)-dione **3d** (100 mg, 0.37 mmol, 1 equiv.), 1,2-DCE (9.5 mL, 0.04 M in relation to **3c**) and TfOH (50 μL, 0.56 mmol, 1.5 equiv.). Reaction time: 16 h. Purification by flash column chromatography (SiO_2, 4:1 DCM:AcOEt) affords the title compound as a yellow solid: 53 mg, 58%

^{1}H NMR (400 MHz; $CDCl_3$) δ: 8.53 (dd, *J* = 7.4 Hz, *J* = 0.8 Hz, 2H), 8.18 (d, *J* = 7.4 Hz, 1H), 8.12 (dd, *J* = 8.0 Hz, *J* = 0.8 Hz, 1H), 8.08 (d, J = 8.0 Hz, 1H), 7.72 (t, *J* = 7.8Hz, 2H), 7.66 (t, *J* = 8.0 Hz, 1H), 6.38 (s, 1H), 4.55 (t, *J* = 7.2 Hz, 2H), 2.71 (t, *J* = 7.2 Hz, 2H).

^{13}C{^{1}H} NMR (100 MHz; $CDCl_3$) δ: 193.7, 161.1, 150.2, 133.5, 132.2, 131.9, 129.9, 127.9, 127.1, 126.8, 125.4, 123.0, 122.7, 103.4, 40.5, 35.1.

M.P.: 229 – 231 °C.

IR (neat, ATR): 3083, 1636, 1558, 1462, 1250, 1146 cm^{-1}.

HRMS (ESI-ToF) m/z: [M + H]$^+$ Calcd. for $C_{16}H_{12}NO_2$: 250.0863, found: 250.0860.

2-(3-oxobutyl)isoindoline-1,3-dione (3e)

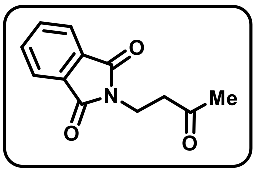

General Procedure A is employed with Na (6 mg, 0.25 mmol, 0.05 equiv.), anhydrous EtOH (1 mL), phthalimide **1e** (735 mg, 5 mmol, 1 equiv.) in anhydrous AcOEt (5.5 mL, 0.9 M in relation to **1e**, dried in 3Å MS) and MVK **2a** (90% grade, 500 μL, 5.5 mmol, 1.1 equiv.). Reaction time 12h. Purification by flash column chromatography (SiO_2, gradient: Hex – 9:1 Hex:AcOEt – 7:3 Hex:AcOEt – 1:1 AcOEt:Hex) affords the title product as a white solid: 1.056 g, 97%.[5]

^{1}H NMR (250 MHz, $CDCl_3$) δ: 7.86 – 7.80 (m, 2H), 7.74 – 7.67 (m, 2H), 3.95 (t, *J* = 7.4 Hz, 2H), 2.87 (t, *J* = 7.4 Hz, 2H), 2.18 (s, 3H).

^{13}C{^{1}H} NMR (62.5 MHz, $CDCl_3$) δ: 205.8, 168.1, 134.0, 132.0, 123.3, 41.6, 33.0, 29.9.

M.P.: 110 – 111°C.

IR (neat, ATR): 1770, 1705, 1438, 1392, 1303, 1176 cm^{-1}.

[5] ^{1}H and ^{13}C NMRs are in good agreement with the literature. See: Hu, K.-F.; Ning, X.-S.; Qu, J.-P.; Kang, Y.-B.; *J. Org. Chem.*, **2018**, *83*, 11327-11332.

HRMS (ESI-ToF) m/z: [M + Na]+ Calcd. for $C_{12}H_{11}NO_3Na$: 240.0631, found: 240.0629.

3,4-dihydropyrido[2,1-a]isoindole-2,6-dione (4e)

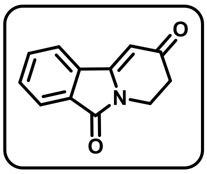

General Procedure B is employed with 2-(3-oxobutyl)isoindoline-1,3-dione **3e** (217 mg, 1 mmol, 1 equiv.), 1,2-DCE (25 mL, 0.04 M in relation to **3e**) and TfOH (135 µL, 1.5 mmol, 1.5 equiv.). Reaction time: 16h. Purification by flash column chromatography (SiO_2, gradient: 9:1 Hex:AcOEt – 8:2 Hex:AcOEt – 7:3 Hex:AcOEt – 1:1 Hex:AcOEt) affords the title compound as a yellow solid: 165 mg, 83%.[3]

^{1}H NMR (250 MHz, $CDCl_3$) δ: 7.92 – 7.88 (m, 1H), 7.76 – 7.72 (m, 1H), 7.71 – 7.63 (m, 2H), 6.01 (s, 1H), 4.13 (t, *J* = 7.4 Hz, 2H), 2.77 (t, *J* = 7.4 Hz, 2H).

^{13}C{^{1}H} NMR (62.5 MHz, $CDCl_3$) δ: 193.6, 166.0, 151.1, 134.2, 132.9, 132.3, 129.8, 124.1, 122.0, 101.7, 37.3, 35.5.

M.P.: 183 – 185 °C.

IR (neat, ATR): 3062, 2978, 1705, 1616, 1354, 1122 cm^{-1}.

HRMS (ESI-ToF) m/z: [M + H]+ Calcd. for $C_{12}H_{10}NO_2$: 200.0706, found: 200.0710.

5-(tert-butyl)-2-(3-oxobutyl)isoindoline-1,3-dione (3f)

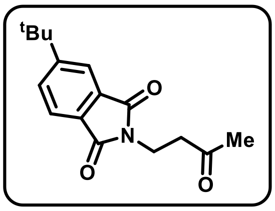

General Procedure A is employed with Na (3.4 mg, 0.15 mmol, 0.05 equiv.), anhydrous EtOH (0.6 mL), 5-(tert-butyl)isoindoline-1,3-dione **1f** (600 mg, 2.96 mmol, 1 equiv.) in anhydrous AcOEt (3.3 mL, 0.9 M in relation to **1f**, dried in 3Å MS) and MVK **2a** (99% grade, 275 µL, 3.25 mmol, 1.1 equiv.). Reaction time 12 h. Purification by flash column chromatography (SiO_2, gradient: 2:1 *c*-Hex:DCM – DCM) affords the title product as a colorless oil: 770 mg, 95%.

^{1}H NMR (400 MHz, $CDCl_3$) δ: 7.87 (d, *J* = 1.2 Hz, 1H), 7.75 (d, *J* = 7.6 Hz, 1H), 7.72 (dd, *J* = 7.6 Hz, *J* = 1.2 Hz, 1H), 3.94 (t, *J* = 7.4 Hz, 2H), 2.86 (t, *J* = 7.4 Hz, 2H), 2.18 (s, 3H), 1.37 (s, 9H).

^{13}C{^{1}H} NMR (100 MHz, $CDCl_3$) δ: 205.9, 168.6, 168.2, 158.7, 132.2, 131.0, 129.3, 123.1, 120.5, 41.6, 35.7, 32.9, 31.1, 29.9.

IR (neat, ATR): 2963, 1705, 1366, 1169, 1029 cm^{-1}.

HRMS (ESI-ToF) m/z: [M + H]+ Calcd. for $C_{16}H_{20}NO_3$: 274.1438, found: 274.1439.

8-(tert-butyl)-3,4-dihydropyrido[2,1-a]isoindole-2,6-dione (4f) and 9-(tert-butyl)-3,4-dihydropyrido[2,1-a]isoindole-2,6-dione (4f')

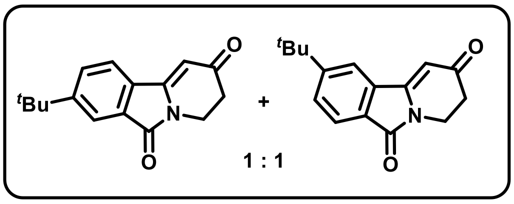

General Procedure B is employed with 5-(tert-butyl)-2-(3-oxobutyl)isoindoline-1,3-dione **3f** (273 mg, 1 mmol, 1 equiv.), 1,2-DCE (25 mL, 0.04 M in relation to **3f**) and TfOH (135 µL, 1.5 mmol, 1.5 equiv.). Reaction time: 16 h. Purification by flash column chromatography (SiO_2, gradient: DCM – 16:1 DCM:AcOEt) affords the title compounds as an inseparable mixture, yellow solid: 245 mg, 96% (1:1 r.r.).

^{1}H NMR (400 MHz, $CDCl_3$) δ: 7.93 (s, 1H), 7.82 (d, *J* = 7.6 Hz, 1H), 7.73 – 7.65 (m, 4H), 6.03 (s, 1H), 5.98 (s, 1H), 4.14 – 4.10 (m, 4H), 2.76 (t, *J* = 7.2 Hz, 4H), 1.391 (s, 9H), 1.390 (s, 9H)
^{13}C{^{1}H} NMR (100 MHz, $CDCl_3$) δ: 193.8, 193.7, 166.5, 166.1, 157.2, 156.9 151.6, 151.2, 134.3, 131.4, 130.1, 129.8 (x2), 127.2, 123.8, 121.8, 121.0, 118.8, 101.4, 101.3, 37.2 (x2), 35.6 (x2), 35.5 (x2), 31.2 (x2).
M.P.: 155 - 172°C.
IR (neat, ATR): 2955, 1717, 1620, 1473, 1357, 1231, 1123, 1034 cm^{-1}.
HRMS (ESI-ToF) m/z: [M + H]$^+$ Calcd. for $C_{16}H_{18}NO_2$: 256.1332, found: 256.1330.

4-fluoro-2-(3-oxobutyl)isoindoline-1,3-dione (3g)

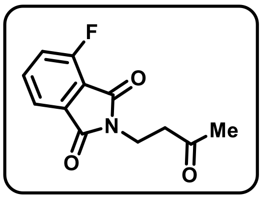

General Procedure A is employed with Na (1.2 mg, 0.05 mmol, 0.05 equiv.), anhydrous EtOH (0.2 mL), 4-fluoroisoindoline-1,3-dione **1g** (173 mg, 1.05 mmol, 1 equiv.) in anhydrous AcOEt (4 mL, 0.26 M in relation to **1g**, dried in 3Å MS) and MVK **2a** (99% grade, 200 µL, 2.4 mmol, 2.3 equiv.). Reaction time 12 h. Purification by flash column chromatography (SiO_2, gradient: 2:1 *c*-Hex:DCM – DCM – 13:1 DCM:AcOEt) affords the title product as a white solid: 197 mg, 80%.

^{1}H NMR (400 MHz, $CDCl_3$) δ: 7.74 – 7.69 (m, 1H), 7.65 (d, *J* = 7.6 Hz, 1H), 7.37 (t, *J* = 8.8 Hz, 1H), 3.93 (t, *J* = 7.4 Hz, 2H), 2.87 (t, *J* = 7.4 Hz, 2H), 2.18 (s, 3H).
^{13}C{^{1}H} NMR (100 MHz, $CDCl_3$) δ: 205.7, 166.8 (d, J_{C-F}= 2.9 Hz), 164.8 (d, J_{C-F}= 2.0 Hz), 157.5 (d, J_{C-F}= 264 Hz), 136.6. (d, J_{C-F}= 8.0 Hz),134.2, (d, J_{C-F}= 1.0 Hz), 122.4, (d, J_{C-F}= 19.0 Hz), 119.5 (d, J_{C-F}= 4.0 Hz), 117.7 (d, J_{C-F}= 13.0 Hz), 41.3, 33.1, 29.9.
^{19}F NMR (376.5 MHz, $CDCl_3$) δ: -112.40.

M.P.: 106 - 108 ºC.

IR (neat, ATR): 2932, 1775, 1705, 1481, 1393, 1176, 1041 cm^{-1}.

HRMS (ESI-ToF) m/z: $[M + H]^+$ Calcd. for $C_{12}H_{11}FNO_3$: 236.0717, found: 236.0717.

7-fluoro-3,4-dihydropyrido[2,1-a]isoindole-2,6-dione (4g)

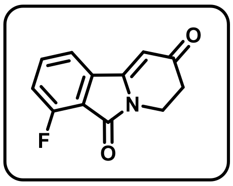

General Procedure B is employed with 4-fluoro-2-(3-oxobutyl)isoindoline-1,3-dione **3g** (107 mg, 0.46 mmol, 1 equiv.), 1,2-DCE (12 mL, 0.04 M in relation to **3g**) and TfOH (60 µL, 0.68 mmol, 1.5 equiv.). Reaction time: 20 h. Purification by flash column chromatography (SiO_2, gradient: DCM – 16:1 DCM:AcOEt) affords the title compounds as a yellow solid: 85 mg, 86%. (A mixture of 6.7:1 r.r. is observed in the ^1H NMR of the crude reaction mixture, but the major compound could be isolated).

^1H NMR (400 MHz, $CDCl_3$) δ: δ 7.68 – 7.63 (m, 1H), 7.54 (d, J = 7.6 Hz, 1H), 7.29 (t, J = 8.6 Hz, 1H), 6.00 (s, 1H), 4.11 (t, J = 7.4 Hz, 2H), 2.76 (t, J = 7.4 Hz, 2H).

$^{13}C\{^1H\}$ NMR (100 MHz, $CDCl_3$) δ: 193.4, 162.7 (d, J_{C-F}= 2.0 Hz), 158.5 (d, J_{C-F} = 262.0 Hz), 150.0 (d, J_{C-F}= 2.0 Hz), 136.5 (d, J_{C-F}= 3.0 Hz), 135.1 (d, J_{C-F}= 8.0 Hz), 119.9 (d, J_{C-F}= 20.0 Hz), 118.1 (d, J_{C-F}= 4.0 Hz), 116.2 (d, J_{C-F} = 14.0 Hz), 102.3, 37.2, 35.3.

^{19}F NMR (376.5 MHz, $CDCl_3$) δ: -114.28.

M.P.: 218 - 221 ºC.

IR (neat, ATR): 3078, 1713, 1620, 1481, 1358, 1227, 1123, 1042 cm^{-1}.

HRMS (ESI-ToF) m/z: $[M + H]^+$ Calcd. for $C_{12}H_9FNO_2$: 218.0612, found: 218.0609.

4-nitro-2-(3-oxobutyl)isoindoline-1,3-dione (3h)

General Procedure A is employed with Na (6 mg, 0.25 mmol, 0.05 equiv.), anhydrous EtOH (1 mL), 4-nitroisoindoline-1,3-dione **1h** (960 mg, 5 mmol, 1 equiv.) in anhydrous AcOEt (5.5 mL, 0.9 M in relation to **1h**, dried in 3Å MS) and MVK **2a** (99% grade, 460 µL, 5.5 mmol, 1.1 equiv.). In this case, DMF (0.9 mL) is also added to the reaction mixture to improve the solubility of imide

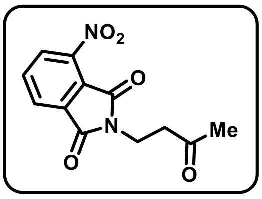

1h. Reaction time 20h. Purification by flash column chromatography (SiO_2, DCM) affords the title product as a white solid: 977 mg, 75%.[6]

^{1}H NMR (400 MHz; $CDCl_3$) δ: 8.12 - 8.10 (m, 2H), 7.91 (dd, *J* = 8.4 Hz, *J* = 7.2 Hz, 1H), 3.99 (t, *J* = 7.2 Hz, 2H). 2.90 (t, *J* = 7.2 Hz, 2H), 2.19 (s, 3H).
^{13}C{^{1}H} NMR (100 MHz; $CDCl_3$) δ: 205.5, 165.4, 162.7, 145.0, 135.4, 134.0, 128.6, 127.1, 123.7, 41.0, 33.6, 29.9.
M.P.: 122 – 123 °C.
IR (neat, ATR): 3090, 1701, 1535, 1350, 1126, 1042 cm^{-1}.
HRMS (ESI-ToF) m/z: [M + H]$^+$ Calcd. for $C_{12}H_{11}N_2O_5$: 263.0662, found: 263.0663.

7-nitro-3,4-dihydropyrido[2,1-a]isoindole-2,6-dione (4h) and 10-nitro-3,4-dihydropyrido[2,1-a]isoindole-2,6-dione (4h')

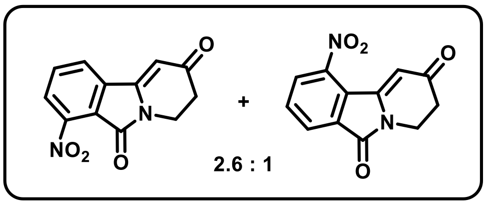

General Procedure B is employed with 4-nitro-2-(3-oxobutyl)isoindoline-1,3-dione **3h** (100 mg, 0.38 mmol, 1 equiv.), 1,2-DCE (9.5 mL, 0.04 M in relation to **3h**) and TfOH (51 μL, 0.57 mmol, 1.5 equiv.). Reaction time: 16 h. Purification by flash column chromatography (SiO_2, 4:1 DCM:AcOEt) affords the title compounds as an inseparable mixture, orange solid: 65 mg, 70% (2.6:1 r.r.).

^{1}H NMR (400 MHz; $CDCl_3$) δ: 8.26 (d, *J* = 8.0Hz, 1H), 8.22 (d, *J* = 7.6 Hz, 1H), 8.05 (d, *J* = 8.0 Hz, 2.6H), 7.99 (d, *J* = 7.6 Hz, 2.6H), 7.88 - 7.83 (m, 3.6H), 6.61 (s, 1H), 6.10 (s, 2.6H), 4.23 - 4.16 (m, 7.2H), 2.84 - 2.78 (m, 7.2H).
^{13}C{^{1}H} NMR (100 MHz; $CDCl_3$) δ: 193.9, 193.0, 163.2, 160.5, 148.2, 146.2, 145.9, 145.3, 136.5, 133.7, 132.8, 132.5, 128.8 (x2), 126.8, 126.5, 125.7, 121.5, 109.8, 103.0, 37.7, 37.4, 35.3, 35.0.
M.P.: 239 – 244 °C.
IR (neat, ATR): 3094, 1717, 1628, 1531, 1342, 1223, 1126 cm^{-1}.
HRMS (ESI-ToF) m/z: [M + H]$^+$ Calcd. for $C_{12}H_9N_2O_4$: 245.0557, found: 245.0560.

[6] ^{1}H NMR is in good agreement with the literature. See: Boulahjar, R.; Ouach, A.; Matteo, C.; Bourg, S.; Ravache, M.; le Guével, R.; Marionneau, S.; Oullier, T.; Lozach, O.; Meijer, L.; Guguen-Guillouzo, C.; Lazar, S.; Akssira, M.; Troin, Y.; Guillaumet, G.; Routier, S.; *J. Med. Chem.*, **2012**, *55*, 9589 – 9606.

2-(3-oxobutyl)hexahydro-1H-isoindole-1,3(2H)-dione (3i)

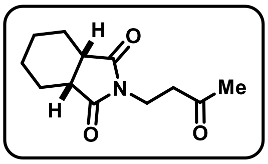

General Procedure A is employed with Na (6 mg, 0.25 mmol, 0.05 equiv.), anhydrous EtOH (1 mL), hexahydro-1H-isoindole-1,3(2H)-dione **1i** (765 mg, 5 mmol, 1 equiv.) in anhydrous AcOEt (5.5 mL, 0.9 M in relation to **1i**, dried in 3Å MS) and MVK **2a** (99% grade, 460 μL, 5.5 mmol, 1.1 equiv.). Reaction time 12 h. Purification by flash column chromatography (SiO_2, gradient: *c*-Hex – 1:1 *c*-Hex:DCM - DCM) affords the title product as white solid: 998 mg, 90%.

^{1}H NMR (400 MHz, $CDCl_3$) δ: 3.73 (t, *J* = 7.2 Hz, 2H), 2.85 – 2.79 (m, 2H), 2.73 (t, *J* = 7.2 Hz, 2H), 2.15 (s, 3H), 1.88 – 1.80 (m, 2H), 1.76 – 1.68 (m, 2H), 1.49 – 1.36 (m, 4H).

^{13}C{^{1}H} NMR (100 MHz, $CDCl_3$) δ: 205.8, 179.5, 40.7, 39.7, 33.5, 29.8, 23.6, 21.6.

M.P.: 80 - 82 ^{o}C.

IR (neat, ATR): 2928, 2859, 1686, 1362, 1161, 1042 cm^{-1}.

HRMS (ESI-ToF) m/z: [M + H]$^+$ Calcd. for $C_{12}H_{18}NO_3$: 224.1281, found: 224.1277.

3,4,6a,7,8,9,10,10a-octahydropyrido[2,1-a]isoindole-2,6-dione (4i)

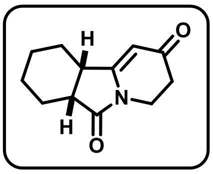

General Procedure B is employed with 2-(3-oxobutyl)hexahydro-1H-isoindole-1,3(2H)-dione **3i** (100 mg, 0.45 mmol, 1 equiv.), 1,2-DCE (11 mL, 0.04 M in relation to **3i**) and TfOH (60 µL, 0.68 mmol, 1.5 equiv.). Reaction time: 16 h. Purification by flash column chromatography (SiO_2, 4:1 DCM:AcOEt) affords the title compounds as yellow solid: 80 mg, 87%.

^{1}H NMR (400 MHz; $CDCl_3$) δ: 5.32 (s, 1H), 4.00 – 3.95 (m, 1H), 3.82 – 3.75 (m, 1H), 3.06 (q, *J* = 7.2 Hz, 1H), 2.73 (q, *J* = 6.8 Hz, 1H), 2.61 - 2.48 (m, 2H), 1.94 – 1.86 (m, 2H), 1.82 - 1.73 (m, 1H), 1.56 – 1.46 (m, 2H), 1.44 – 1.29 (m, 3H).

^{13}C{^{1}H} NMR (100 MHz; $CDCl_3$) δ: 192.9, 176.2, 165.1, 101.7, 40.0, 37.8, 36.8, 34.3, 27.6, 23.2, 22.0, 21.9.

M.P.: 110 – 111 °C.

IR (neat, ATR): 2928, 2851, 1686, 1362, 1161, 1041 cm^{-1}.

HRMS (ESI-ToF) m/z: [M + H]$^+$ Calcd. for $C_{12}H_{16}NO_2$: 206.1176, found: 206.1174.

1-(3-oxopentyl)pyrrolidine-2,5-dione (3j)

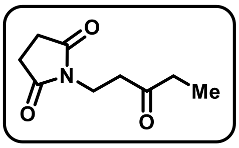

General Procedure A is employed with Na (6 mg, 0.25 mmol, 0.05 equiv.), anhydrous EtOH (1 mL), succinimide **1a** (495 mg, 5 mmol, 1 equiv.) in anhydrous AcOEt (5.5 mL, 0.9 M in relation to **1a**, dried in 3Å MS) and pent-1-en-3-one **2b** (545 μL, 5.5 mmol, 1.1 equiv.). Reaction time 12 h. Purification by flash column chromatography (SiO_2, gradient: Hex – 9:1 Hex:AcOEt – 7:3 Hex:AcOEt – 1:1 AcOEt:Hex) affords the title product as a pale yellow oil, that solidifies in the freezer producing a white solid: 457 mg, 50%.

^{1}H NMR (250 MHz, $CDCl_3$) δ: 3.78 (t, J = 7.4 Hz, 2H), 2.76 – 2.69 (m, 6H), 2.43 (q, J = 7.4 Hz, 2H), 1.04 (t, J = 7.4 Hz, 3H).
^{13}C{^{1}H} NMR (62.5 MHz, $CDCl_3$) δ: 208.5, 176.9, 39.2, 35.9, 33.9, 28.1, 7.6.
M.P.: 53 - 55 °C.
IR (neat, ATR): 2940, 1769, 1691, 1407, 1340, 1156, 1105 cm^{-1}.
HRMS (ESI-Orbitrap) m/z: [M + H]$^+$ Calcd. for $C_9H_{14}NO_3$: 184.0968, found: 184.0966.

8-methyl-1,2,5,6-tetrahydroindolizine-3,7-dione (4j)

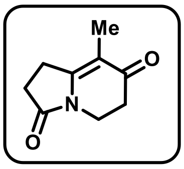

General Procedure B is employed with 1-(3-oxopentyl)pyrrolidine-2,5-dione **3j** (37 mg, 0.2 mmol, 1 equiv.), 1,2-DCE (5 mL, 0.04 M in relation to **3j**) and TfOH (27 µL, 0.3 mmol, 1.5 equiv.). Reaction time: 48 h. Purification by flash column chromatography (SiO_2, gradient: 8:2 Hex:AcOEt – 6:4 Hex:AcOEt – 6:4 AcOEt:Hex) affords the title compounds as a white solid: 30 mg, 90%.

^{1}H NMR (250 MHz, $CDCl_3$) δ: 3.85 (t, J = 7.8 Hz, 2H), 2.88 – 2.82 (m, 2H), 2.66 – 2.60 (m, 2H), 2.55 (t, J = 7.8 Hz, 2H), 1.72 (s, 3H).
^{13}C{^{1}H} NMR (62.5 MHz, $CDCl_3$) δ: 192.2, 174.7, 156.6, 109.5, 37.7, 34.0, 28.4, 22.9, 9.2.
M.P.: 50 - 52 °C.
IR (neat, ATR): 2920, 1719, 1568, 1334, 1269, 1176, 1129 cm^{-1}.
HRMS (ESI-Orbitrap) m/z: [M + H]$^+$ Calcd. for $C_9H_{12}NO_2$: 166.0863, found: 166.0860.

1-(3-oxopentyl)piperidine-2,6-dione (3k)

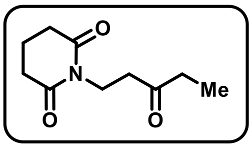

General Procedure A is employed with Na (6 mg, 0.25 mmol, 0.05 equiv.), anhydrous EtOH (1 mL), glutarimide **1b** (565 mg, 5 mmol, 1 equiv.) in anhydrous AcOEt (5.5 mL, 0.9 M in relation to **1b**, dried in 3Å MS) and pent-1-en-3-one **2b** (695 μL, 7 mmol, 1.4 equiv.). Reaction time 12 h. Purification by flash column chromatography (SiO_2, gradient: Hex – 9:1 Hex:AcOEt – 7:3 Hex:AcOEt – 1:1 AcOEt:Hex) affords the title product as a colorless oil: 640 mg, 65%.

^{1}H NMR (250 MHz, $CDCl_3$) δ: 4.01 (t, *J* = 7.4 Hz, 2H), 2.65 – 2.58 (m, 6H), 2.42 (q, *J* = 7.4 Hz, 2H), 1.97 – 1.87 (m, 2H), 1.02 (t, *J* = 7.4 Hz, 3H).

^{13}C{^{1}H} NMR (62.5 MHz, $CDCl_3$) δ: 209.3, 172.4, 40.1, 35.9, 35.0, 32.7, 17.0, 7.6.

IR (neat, ATR): 2972, 2942, 1709, 1667, 1348, 1168, 1100, 1055 cm^{-1}.

HRMS (ESI-Orbitrap) m/z: $[M + H]^+$ Calcd. for $C_{10}H_{16}NO_3$: 198.1125, found: 198.1122.

1-methyl-3,4,8,9-tetrahydro-2H-quinolizine-2,6(7H)-dione (4k)

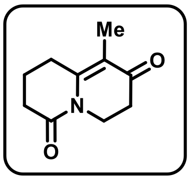

General Procedure B is employed with 1-(3-oxopentyl)piperidine-2,6-dione **3k** (39 mg, 0.2 mmol, 1 equiv.), 1,2-DCE (5 mL, 0.04 M in relation to **3j**) and TfOH (27 μL, 0.3 mmol, 1.5 equiv.). Reaction time: 24 h. Purification by flash column chromatography (SiO_2, gradient: 8:2 Hex:AcOEt – 6:4 Hex:AcOEt – 1:1 Hex:AcOEt) affords the title compounds as a white solid: 27 mg, 75%.

^{1}H NMR (250 MHz, $CDCl_3$) δ: 4.11 (t, *J* = 7.4 Hz, 2H), 2.69 (t, *J* = 6.4 Hz, 2H), 2.61 – 2.49 (m, 4H), 1.98 – 1.88 (m, 2H), 1.79 (s, 3H).

^{13}C{^{1}H} NMR (62.5 MHz, $CDCl_3$) δ: 192.9, 169.1, 151.4, 113.0, 39.9, 35.6, 32.6, 26.8, 18.6, 10.1.

M.P.: 107 - 109 °C.

IR (neat, ATR): 2967, 2892, 1680, 1639, 1560, 1343, 1173, 1139 cm^{-1}.

HRMS (ESI-Orbitrap) m/z: $[M + H]^+$ Calcd. for $C_{10}H_{14}NO_2$: 180.1019, found: 180.1017.

2-(3-oxopentyl)isoindoline-1,3-dione (3l)

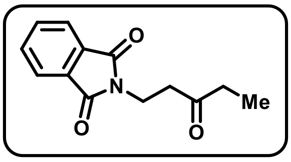

General Procedure A is employed with Na (6 mg, 0.25 mmol, 0.05 equiv.), anhydrous EtOH (1 mL), phthalimide **1e** (735 mg, 5 mmol, 1 equiv.) in anhydrous AcOEt (5.5 mL, 0.9 M in relation to **1e**, dried in 3Å MS) and pent-1-en-3-one **2b** (695 μL, 7 mmol, 1.4 equiv.). Reaction time 12 h. Purification by flash column chromatography (SiO_2, gradient: Hex – 9:1 Hex:AcOEt – 7:3 Hex:AcOEt – 1:1 AcOEt:Hex - 7:3AcOEt:Hex) affords the title product as a white solid: 853 mg, 74%.[7]

^{1}H NMR (250 MHz, $CDCl_3$) δ: 7.86 – 7.79 (m, 2H), 7.74 – 7.67 (m, 2H), 3.95 (t, *J* = 7.4 Hz, 2H), 2.84 (t, *J* = 7.4 Hz, 2H), 2.46 (q, *J* = 7.4 Hz, 2H), 1.05 (t, *J* = 7.4 Hz, 3H).

^{13}C{^{1}H} NMR (62.5 MHz, $CDCl_3$) δ: 208.5, 168.1, 134.0, 132.0, 123.2, 40.2, 35.9, 33.1, 7.6.

M.P.: 90 - 92 ^{o}C.

IR (neat, ATR): 2976, 1768, 1701, 1408, 1369, 1330, 1118 cm^{-1}.

HRMS (ESI-Orbitrap) m/z: [M + H]$^+$ Calcd. for $C_{13}H_{14}NO_3$: 232.0968, found: 232.0964.

1-methyl-3,4-dihydropyrido[2,1-a]isoindole-2,6-dione (4l)

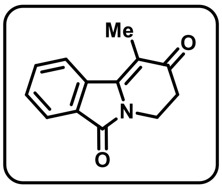

General Procedure B is employed with 2-(3-oxopentyl)isoindoline-1,3-dione **3l** (46 mg, 0.2 mmol, 1 equiv.), 1,2-DCE (5 mL, 0.04 M in relation to **3l**) and TfOH (27 µL, 0.3 mmol, 1.5 equiv.). Reaction time: 24 h. Purification by flash column chromatography (SiO_2, gradient: 9:1 Hex:AcOEt – 8:2 Hex:AcOEt – 7:3 Hex:AcOEt) affords the title compounds as a white solid: 34 mg, 80%.

^{1}H NMR (250 MHz, $CDCl_3$) δ: 7.95 – 7.90 (m, 2H), 7.71 – 7.59 (m, 2H), 4.10 (t, *J* = 7.4 Hz, 2H), 2.78 (t, *J* = 7.4 Hz, 2H), 2.27 (s, 3H).

^{13}C{^{1}H} NMR (62.5 MHz, $CDCl_3$) δ: 193.9, 165.6, 146.0, 134.7, 132.6, 131.2, 130.3, 125.2, 124.1, 114.1, 36.9, 35.2, 9.9.

M.P.: 173 - 175 °C.

IR (neat, ATR): 2919, 1636, 1558, 1469, 1343, 1144, 1084 cm^{-1}.

HRMS (ESI-Orbitrap) m/z: [M + H]$^+$ Calcd. for $C_{13}H_{12}NO_2$: 214.0863, found: 214.0859.

---

[7] ^{1}H and ^{13}C NMRs are in good agreement with the literature. See: Griffin, J. D.; Vogt, D. B.; Du Bois, J.; Sigman, M. S.; *ACS Catal.*, **2021**, *11*, 10479 – 10486.

1-(3-oxooctyl)pyrrolidine-2,5-dione (3m)

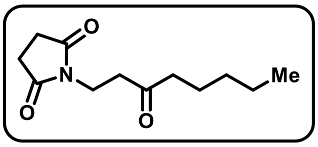

General Procedure A is employed with Na (6 mg, 0.25 mmol, 0.05 equiv.), anhydrous EtOH (1 mL), succinimide **1a** (495 mg, 5 mmol, 1 equiv.) in anhydrous AcOEt (5.5 mL, 0.9 M in relation to **1a**, dried in 3Å MS) and oct-1-en-3-one **2c** (96% grade, 870 µL, 5.5 mmol, 1.1 equiv.). Reaction time 12 h. Purification by flash column chromatography (SiO_2, gradient: Hex – 8:2 Hex:AcOEt – 1:1 Hex:AcOEt – 7:3 AcOEt:Hex) affords the title product as a colorless oil, that solidifies in the freezer producing a white solid: 644 mg, 57%.

^{1}H NMR (250 MHz, $CDCl_3$) δ: 3.77 (t, *J* = 7.4 Hz, 2H), 2.75 – 2.69 (m, 6H), 2.40 (t, *J* = 7.4 Hz, 2H), 1.59 – 1.50 (m, 2H), 1.36 – 1.20 (m, 4H), 0.88 (t, *J* = 6.8 Hz, 3H).
^{13}C{^{1}H} NMR (62.5 MHz, $CDCl_3$) δ: 208.2, 177.0, 42.8, 39.5, 33.9, 31.3, 28.1, 23.3, 22.4, 13.9.
M.P.: 36 - 37 ^{o}C.
IR (neat, ATR): 2932, 2865, 1693, 1403, 1372, 1154 cm^{-1}.
HRMS (ESI-Orbitrap) m/z: [M + H]$^+$ Calcd. for $C_{12}H_{20}NO_3$: 226.1438, found: 226.1433.

8-butyl-1,2,5,6-tetrahydroindolizine-3,7-dione (4m)

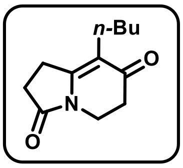

General Procedure B is employed with 1-(3-oxooctyl)pyrrolidine-2,5-dione **3m** (45 mg, 0.2 mmol, 1 equiv.), 1,2-DCE (5 mL, 0.04 M in relation to **3m**) and TfOH (27 µL, 1.5 mmol, 1.5 equiv.). Reaction time: 28 h. Purification by flash column chromatography (SiO_2, gradient: 8:2 Hex:AcOEt – 1:1 Hex:AcOEt – AcOEt) affords the title compounds as a yellowish solid: 35 mg, 85%.

^{1}H NMR (250 MHz, $CDCl_3$) δ 3.85 (t, *J* = 7.6 Hz, 2H), 2.91 – 2.85 (m, 2H), 2.69 – 2.60 (m, 2H), 2.55 (t, *J* = 7.6 Hz, 2H), 2.17 (t, *J* = 7.6 Hz, 2H), 1.43 – 1.24 (m, 4H), 0.90 (t, *J* = 7.0 Hz, 3H).
^{13}C{^{1}H} NMR (62.5 MHz, $CDCl_3$) δ: 192.0, 174.7, 156.5, 114.6, 37.7, 34.2, 31.2, 28.4, 24.3, 22.7, 22.6, 13.9.
M.P.: 53 - 54 °C.
IR (neat, ATR): 2930, 2863, 1726, 1609, 1413, 1337, 1193, 1130 cm^{-1}.
HRMS (ESI-Orbitrap) m/z: [M + H]$^+$ Calcd. for $C_{12}H_{18}NO_2$: 208.1332, found: 208.1329.

1-(2-methyl-3-oxobutyl)pyrrolidine-2,5-dione (3n)

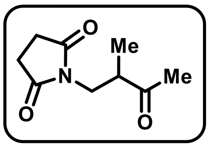

General Procedure A is employed with Na (3 mg, 0.125 mmol, 0.05 equiv.), anhydrous EtOH (0.5 mL), succinimide **1a** (247 mg, 2.5 mmol, 1 equiv.) in anhydrous AcOEt (3 mL, 0.9 M in relation to **1a**, dried in 3Å MS) and 3-methylbut-3-en-2-one **2d** (345 μL, 3.5 mmol, 1.4 equiv.). Reaction time 48h. Purification by flash column chromatography (SiO_2, gradient: Hex:AcOEt – 7:3 Hex:AcOEt – 1:1 AcOEt:Hex) affords the title product as a colorless oil: 46 mg, 10%.

^{1}H NMR (250 MHz, $CDCl_3$) δ: 3.74 (dd, *J* = 13.5 Hz, *J* = 6.8 Hz, 1H), 3.56 (dd, *J* = 13.5 Hz, *J* = 7.4 Hz, 1H), 3.02 – 2.88 (m, 1H), 2.69 (s, 4H), 2.18 (s, 3H), 1.10 (d, *J* = 7.0 Hz, 3H).
^{13}C{^{1}H} NMR (62.5 MHz, $CDCl_3$) δ: 209.6, 177.1, 45.0, 40.5, 28.1, 27.8, 14.4.
IR (neat, ATR): 2975, 2941, 1691, 1401, 1354, 1200, 1163, 1119 cm^{-1}.
HRMS (ESI-Orbitrap) m/z: $[M + H]^+$ Calcd. for $C_9H_{14}NO_3$: 184.0968, found: 184.0968.

6-methyl-1,2,5,6-tetrahydroindolizine-3,7-dione (4n)

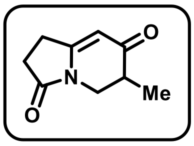

General Procedure B is employed with 1-(2-methyl-3-oxobutyl)pyrrolidine-2,5-dione **3n** (27 mg, 0.15 mmol, 1 equiv.), 1,2-DCE (5 mL, 0.03 M in relation to **3n**) and TfOH (20 µL, 0.225 mmol, 1.5 equiv.). Reaction time: 24 h. Purification by flash column chromatography (SiO_2, gradient: 8:2 Hex:AcOEt – 6:4 Hex:AcOEt – 4:6 Hex:AcOEt) affords the title compounds as awhite solid: 20 mg, 80%.

^{1}H NMR (250 MHz, $CDCl_3$) δ: 5.30 (s, 1H), 4.08 (dd, *J* = 13.5 Hz, *J* = 6.3 Hz, 1H), 3.34 (dd, *J* = 13.5 Hz, *J* = 11.0 Hz, 1H), 2.93 – 2.87 (m, 2H), 2.66 – 2.60 (m, 2H), 2.58 – 2.43 (m, 1H), 1.16 (d, *J* = 7.0 Hz, 3H).
^{13}C{^{1}H} NMR (62.5 MHz, $CDCl_3$) δ: 195.3, 174.9, 160.6, 101.8, 44.0, 37.4, 28.0, 23.4, 13.5.
M.P.: 113 - 115 °C.
IR (neat, ATR): 3066, 2970, 2933, 1723, 1602, 1424, 1325, 1154 cm^{-1}.
HRMS (ESI-Orbitrap) m/z: $[M + H]^+$ Calcd. for $C_9H_{12}NO_2$: 166.0868, found: 166.0863.

1-(4-oxopentan-2-yl)pyrrolidine-2,5-dione (3o)

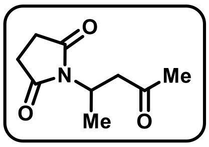

General Procedure A is employed with Na (6 mg, 0.25 mmol, 0.05 equiv.), anhydrous EtOH (1 mL), succinimide **1a** (495 mg, 5 mmol, 1 equiv.) in anhydrous AcOEt (5.5 mL, 0.9 M in relation to **1a**, dried in 3Å MS) and pent-3-en-2-one **2e** (70% grade, 770 μL, 5.5 mmol, 1.1 equiv.). Reaction time 12 h. Purification by flash column chromatography (SiO_2, gradient: Hex – 8:2 Hex:AcOEt – 6:4 Hex:AcOEt – 1:1 AcOEt:Hex) affords the title product as a yellow oil: 576 mg, 63% (contaminated with a small amount of succinimide **1a**).

^{1}H NMR (250 MHz, $CDCl_3$) δ: 4.69 – 4.55 (m, 1H), 3.21 (dd, *J* = 17.8 Hz, *J* = 8.5 Hz, 1H), 2.86 (dd, *J* = 17.8Hz, *J* = 6.3 Hz, 1H), 2.61 (s, 4H), 2.09 (s, 3H), 1.30 (d, *J* = 7.0 Hz, 3H).
^{13}C{^{1}H} NMR (62.5 MHz, $CDCl_3$) δ: 205.8, 177.2, 45.6, 43.1, 30.0, 27.9, 17.9.
IR (neat, ATR): 2984, 1770, 1688, 1365, 1163, 1107, 1002 cm^{-1}.
HRMS (ESI-Orbitrap) m/z: [M + H]$^+$ Calcd. for $C_9H_{14}NO_3$: 184.0968, found: 184.0965.

5-methyl-1,2,5,6-tetrahydroindolizine-3,7-dione (4o)

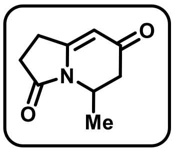

General Procedure B is employed with 1-(4-oxopentan-2-yl)pyrrolidine-2,5-dione **3o** (37 mg, 0.2 mmol, 1 equiv.), 1,2-DCE (5 mL, 0.04 M in relation to **3o**) and TfOH (27 µL, 0.30 mmol, 1.5 equiv.). Reaction time: 46 h. Purification by flash column chromatography (SiO_2, gradient: 8:2 Hex:AcOEt – 6:4 Hex:AcOEt – 1:1 Hex:AcOEt) affords the title compounds as a brownish solid: 24 mg, 73%.

^{1}H NMR (250 MHz, $CDCl_3$) δ: 5.31 (s, 1H), 4.62 – 4.51 (m, 1H), 2.92 – 2.85 (m, 2H), 2.74 (dd, *J* = 16.5 Hz, *J* = 7.5 Hz, 1H), 2.63 – 2.57 (m, 2H), 2.31 (d, *J* = 16.5 Hz, 1H), 1.23 (d, *J* = 6.8 Hz, 3H).
^{13}C{^{1}H} NMR (62.5 MHz, $CDCl_3$) δ: 192.1, 174.4, 159.8, 101.9, 45.0, 40.7, 28.1, 23.6, 17.4.
M.P.: 61 – 63 ^{o}C.
IR (neat, ATR): 2976, 1724, 1656, 1431, 1351, 1313, 1158, 1073 cm^{-1}.
HRMS (ESI-Orbitrap) m/z: [M + H]$^+$ Calcd. for $C_9H_{12}NO_2$: 166.0863, found: 166.0861.

1-(2-methyl-5-oxohexan-3-yl)pyrrolidine-2,5-dione (3p)

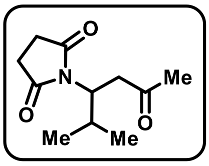

General Procedure A is employed with Na (6 mg, 0.25 mmol, 0.05 equiv.), anhydrous EtOH (1 mL), succinimide **1a** (495 mg, 5 mmol, 1 equiv.) in anhydrous AcOEt (5.5 mL, 0.9 M in relation to **1a**, dried in 3Å MS) and 5-methylhex-3-en-2-one **2f** (75% grade, 970 μL, 5.5 mmol, 1.1 equiv.). Reaction time 12 h. Purification by flash column chromatography (SiO_2, gradient: Hex – 8:2 Hex:AcOEt – 1:1 Hex:AcOEt – 7:3 AcOEt:Hex) affords the title product as a colorless oil: 644 mg, 61%.

^{1}H NMR (250 MHz, $CDCl_3$) δ: 4.17 (td, *J* = 10.5 Hz, *J* = 4.0 Hz, 1H), 3.36 (dd, *J* = 17.3 Hz, *J* = 10.5 Hz, 1H), 2.80 (dd, *J* = 17.3 Hz, *J* = 4.0 Hz, 1H), 2.64 (s, 4H), 2.27 – 2.15 (m, 1H), 2.11 (s, 3H), 0.95 (d, *J* = 6.8 Hz, 3H), 0.80 (d, *J* = 6.8 Hz, 3H).
^{13}C{^{1}H} NMR (62.5 MHz, $CDCl_3$) δ: 206.5, 177.6, 53.6, 42.0, 30.1, 29.8, 27.8, 19.8, 19.5.
IR (neat, ATR): 2934, 2866, 1696, 1404, 1372, 1154 cm^{-1}.
HRMS (ESI-Orbitrap) m/z: $[M + H]^+$ Calcd. for $C_{11}H_{18}NO_3$: 212.1281, found: 212.1278.

5-isopropyl-1,2,5,6-tetrahydroindolizine-3,7-dione (4p)

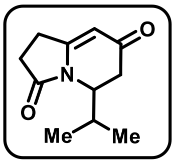

General Procedure B is employed with 1-(2-methyl-5-oxohexan-3-yl)pyrrolidine-2,5-dione **3p** (42 mg, 0.2 mmol, 1 equiv.), 1,2-DCE (5 mL, 0.04 M in relation to **3p**) and TfOH (27 µL, 1.5 mmol, 1.5 equiv.). Reaction time: 16 h. Purification by flash column chromatography (SiO_2, gradient: 8:2 Hex:AcOEt – 1:1 Hex:AcOEt – AcOEt) affords the title compounds as yellowish solid: 20 mg, 52%.

^{1}H NMR (250 MHz, $CDCl_3$) δ: 5.28 (s, 1H), 4.23 (td, *J* = 6.8 Hz, *J* = 2.4 Hz, 1H), 2.93 – 2.86 (m, 2H), 2.70 – 2.54 (m, 4H), 2.16 – 2.02 (m, 1H), 0.91 (d, *J* = 6.8 Hz, 3H), 0.90 (d, *J* = 6.8 Hz, 3H).
^{13}C{^{1}H} NMR (62.5 MHz, $CDCl_3$) δ: 192.6, 174.9, 160.8, 102.4, 54.4, 36.2, 30.6, 28.1, 23.6, 19.4, 18.2.
M.P.: 65 - 66 °C.
IR (neat, ATR): 2962, 2935, 2878, 1729, 1657, 1598, 1419, 1308, 1132 cm^{-1}.
HRMS (ESI-Orbitrap) m/z: $[M + H]^+$ Calcd. for $C_{11}H_{16}NO_2$: 194.1176, found: 194.1173.

2-(2-methyl-5-oxohexan-3-yl)isoindoline-1,3-dione (3q)

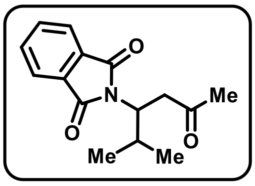

General Procedure A is employed with Na (6 mg, 0.25 mmol, 0.05 equiv.), anhydrous EtOH (1 mL), phthalimide **1e** (735 mg, 5 mmol, 1 equiv.) in anhydrous AcOEt (5.5 mL, 0.9 M in relation to **1e**, dried in 3Å MS) and 5-methylhex-3-en-2-one **2f** (75% grade, 970 μL, 5.5 mmol, 1.1 equiv.). Reaction time: 12h h. Purification by flash column chromatography (SiO_2, gradient: 9:1 Hex:AcOEt – 8:2 Hex:AcOEt – 6:4 AcOEt:Hex) affords the title product as a white solid: 725 mg, 56%.

^{1}H NMR (250 MHz, $CDCl_3$) δ: 7.83 – 7.77 (m, 2H), 7.72 – 7.65 (m, 2H), 4.39 (td, *J* = 9.8 Hz, *J* = 4.0 Hz, 1H), 3.47 (dd, *J* = 17.4 Hz, *J* = 10.5 Hz, 1H), 2.89 (dd, *J* = 17.4Hz, *J* = 4.0 Hz, 1H), 2.36 – 2.19 (m, 1H), 2.11 (s, 3H), 1.00 (d, *J* = 6.8 Hz, 3H), 0.86 (d, *J* = 6.8 Hz, 3H).
^{13}C{^{1}H} NMR (62.5 MHz, $CDCl_3$) δ: 206.3, 168.6, 133.9, 131.7, 123.2, 53.0, 43.1, 30.8, 30.1, 20.0, 19.6.
M.P.: 57 – 59 ^{o}C
IR (neat, ATR): 2968, 1772, 1706, 1468, 1370, 1236, 1043 cm^{-1}.
HRMS (ESI-Orbitrap) m/z: $[M + H]^+$ Calcd. for $C_{15}H_{18}NO_3$: 260.1281, found: 260.1278.

4-isopropyl-3,4-dihydropyrido[2,1-a]isoindole-2,6-dione (4q)

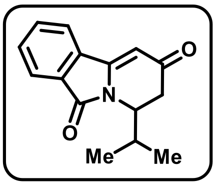

General Procedure B is employed with 2-(2-methyl-5-oxohexan-3-yl)isoindoline-1,3-dione **3q** (52 mg, 0.2 mmol, 1 equiv.), 1,2-DCE (5 mL, 0.04 M in relation to **3q**) and TfOH (27 μL, 0.3 mmol, 1.5 equiv.). Reaction time: 18h. Purification by flash column chromatography (SiO_2, gradient: 9:1 Hex:AcOEt – 8:2 Hex:AcOEt – 7:3 Hex:AcOEt) affords the title compounds as a yellow solid: 36 mg, 75%.

^{1}H NMR (250 MHz, $CDCl_3$) δ: 7.92 – 7.86 (m, 1H), 7.75 - 7.62 (m, 3H), 5.97 (s, 1H), 4.50 (td, *J* = 6.4, *J* = 3.0 Hz, 1H), 2.87 – 2.73 (m, 2H), 2.29 – 2.09 (m, 1H), 0.93 (d, *J* = 6.9 Hz, 3H), 0.92 (d, *J* = 6.9 Hz, 3H) .
^{13}C{^{1}H} NMR (62.5 MHz, $CDCl_3$) δ: 194.0, 166.1, 150.4, 133.9, 132.7, 132.2, 129.6, 124.0, 121.9, 101.5, 54.2, 37.7, 32.0, 19.4, 18.2.
M.P.: 101 – 103 ^{o}C
IR (neat, ATR): 3059, 2958, 2872, 1725, 1619, 1468, 1091, 1008 cm^{-1}.

HRMS (ESI-Orbitrap) m/z: [M + H]+ Calcd. for $C_{15}H_{16}NO_2$: 242.1176, found: 242.1171.

1-(3-oxo-1-phenylbutyl)pyrrolidine-2,5-dione (3r)

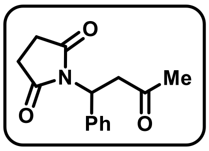

General Procedure A is employed with Na (6 mg, 0.25 mmol, 0.05 equiv.), anhydrous EtOH (1 mL), succinimide **1a** (495 mg, 5 mmol, 1 equiv.) in anhydrous AcOEt (5.5 mL, 0.9 M in relation to **1a**, dried in 3Å MS) and 4-phenylbut-3-en-2-one **2g** (803 mg, 5.5 mmol, 1.1 equiv.). Reaction time 12 h. Purification by flash column chromatography (SiO_2, gradient: Hex – 8:2 Hex:AcOEt – 1:1 Hex:AcOEt – 7:3 AcOEt:Hex) affords the title product as a colorless oil: 895 mg, 73%.

^{1}H NMR (250 MHz, $CDCl_3$) δ: 7.47 – 7.42 (m, 2H), 7.35 – 7.27 (m, 3H), 5.63 (dd, J = 10.2 Hz, J = 5.4 Hz, 1H), 3.95 (dd, J = 18.1 Hz, J = 10.2 Hz, 1H), 3.16 (dd, J = 18.1 Hz, J = 5.4 Hz, 1H), 2.62 (s, 4H), 2.15 (s, 3H).

^{13}C{^{1}H} NMR (62.5 MHz, $CDCl_3$) δ: 205.4, 177.2, 138.6, 128.7, 128.2, 127.8, 50.8, 43.8, 30.0, 27.9.

IR (neat, ATR): 2931, 1692, 1387, 1357, 1166 cm^{-1}.

HRMS (ESI-Orbitrap) m/z: [M + H]+ Calcd. for $C_{14}H_{16}NO_3$: 246.1125, found: 246.1120.

1-(2-(2-methyl-1,3-dioxolan-2-yl)ethyl)pyrrolidine-2,5-dione (5a)

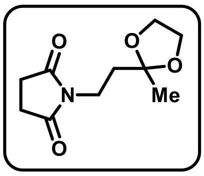

Under air, at room temperature, a round bottom flask equipped with a Dean-Stark aparatus and a reflux condenser is charged with 1-(3-oxobutyl)pyrrolidine-2,5-dione **3a** (169 mg, 1 mmol, 1 equiv.), toluene (0.1 M, 10 mL), ethylene glycol (111 µL, 2 mmol, 2 equiv.) and *p*TSA·H_2O (19 mg, 0.1 mmol, 0.1 equiv.). The resulting mixture is heated to the reflux of toluene (110 ^oC) overnight. Then, the reaction is quenched with an aqueous saturated solution of $NaHCO_3$, extracted with AcOEt (3x), dried ($MgSO_4$) and concentrated under reduced pressure. Purification by flash column chromatography (SiO_2, gradient: Hex – 8:2 Hex:AcOEt – 1:1 Hex:AcOEt) affords the title compound as a white solid: 111 mg, 52%.

^{1}H NMR (250 MHz, $CDCl_3$) δ: 3.92 (s, 4H), 3.62 (t, J = 7.3 Hz, 2H), 2.66 (s, 4H), 1.95 (t, J = 7.3 Hz, 2H), 1.33 (s, 3H).

^{13}C{^{1}H} NMR (62.5 MHz, $CDCl_3$) δ: 177.1, 108.7, 64.6, 35.4, 34.3, 28.1, 23.6.

M.P.: 80 – 82 °C.

IR (neat, ATR): 2995, 2963, 2888, 1694, 1406, 1382, 1339, 1179, 1149, 1057 cm^{-1}.
HRMS (ESI-ToF) m/z: [M + H]$^+$ Calcd. for $C_{10}H_{16}NO_4$: 214.1074, found: 214.1073.

1-(2-(2-methyl-1,3-dioxolan-2-yl)ethyl)piperidine-2,6-dione (5b)

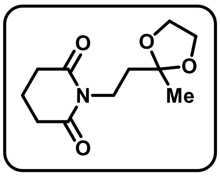

Under air, at room temperature, a round bottom flask equipped with a Dean-Stark aparatus and a reflux condenser is charged with 1-(3-oxobutyl)piperidine-2,6-dione **3b** (183 mg, 1 mmol, 1 equiv.), toluene (0.1 M, 10 mL), ethylene glycol (111 μL, 2 mmol, 2 equiv.) and *p*TSA·H_2O (19 mg, 0.1 mmol, 0.1 equiv.). The resulting mixture is heated to the reflux of toluene (110 ^oC) overnight. Then, the reaction is quenched with an aqueous saturated solution of $NaHCO_3$, extracted with AcOEt (3x), dried ($MgSO_4$) and concentrated under reduced pressure. Purification by flash column chromatography (SiO_2, gradient: Hex – 8:2 Hex:AcOEt – 1:1 Hex:AcOEt) affords the title compound as a colorless oil: 172 mg, 76%.

^{1}H NMR (250 MHz, $CDCl_3$) δ: 3.93 (s, 4H), 3.92 – 3.86 (m, 2H), 2.62 (t, *J* = 6.5 Hz, 4H), 1.96 – 1.83 (m, 4H), 1.37 (s, 3H).
^{13}C{^{1}H} NMR (62.5 MHz, $CDCl_3$) δ: 172.3, 108.9, 64.5, 36.1, 35.3, 32.9, 23.6, 17.1.
IR (neat, ATR): 2960, 2885, 1720, 1666. 1140, 1350 cm^{-1}.
HRMS (ESI-ToF) m/z: [M + H]$^+$ Calcd. for $C_{11}H_{18}NO_4$: 228.1230, found: 228.1234.

1-(hex-5-en-1-yl)pyrrolidine-2,5-dione (7a)

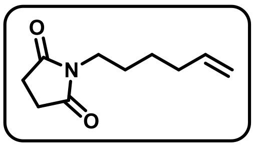

The procedure employed here was originally reported by Martin and co-workers.[8] Under N_2, at room temperature, a round bottom flask is charged with succinimide **1a** (495 mg, 5 mmol, 1 equiv.), dry THF (30 mL, 0.16M, dried from SPS), PPh_3 (1.70 g, 6.5 mmol, 1.3 equiv.) and hex-5-en-1-ol **6** (720 μL, 6 mmol, 1.2 equiv.). The reaction temperature is cooled down to 0 ^oC, and diisopropyl azodicarboxylate (1.28 mL, 6.5 mmol, 1.3 equiv.) is slowly added. The temperature is allowed to warm up to room temperature and the reaction is stirred at this temperature for 24 hours, then being concentrated

[8] ^{1}H and ^{13}C NMRs are in good agreement with the literature. See: Martin, S. F.; Chen, H.-J.; Courtney, A. K.; Liao, Y.; Pätzel, M.; Ramser, M. N.; Wagman, A. S., *Tetrahedron* **1996**, *52*, 7251-7264.

under reduced pressure. Purification by flash column chromatography (SiO_2, gradient: Hex – 95:5 Hex:AcOEt – 9:1 Hex:AcOEt) affords the title compound as a colorless oil: 643 mg, 71%.[6]

^{1}H NMR (250 MHz, $CDCl_3$) δ: 5.83 – 5.67 (m, 1H), 5.03 – 4.90 (m, 2H), 3.49 (t, *J* = 7.4 Hz, 2H), 2.68 (s, 4H), 2.10 – 2.01 (m, 2H), 1.60 – 1.50 (m, 2H), 1.43 – 1.30 (m, 2H).
^{13}C{^{1}H} NMR (62.5 MHz, $CDCl_3$) δ: 177.2, 138.1, 114.8, 38.6, 33.1, 28.1, 27.1, 26.0.
IR (neat, ATR): 3020, 2939, 2862, 1774, 1693, 1639, 1400 cm^{-1}.
HRMS (ESI-ToF) m/z: $[M + H]^+$ Calcd. for $C_{10}H_{16}NO_2$: 182.1176, found: 182.1182.

5-(2,5-dioxopyrrolidin-1-yl)pentanal (8a)

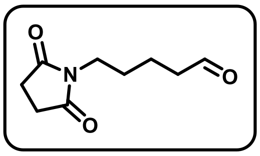

1-(hex-5-en-1-yl)pyrrolidine-2,5-dione **7a** (543 mg, 3 mmol, 1 equiv.) is dissolved in a 7:1 mixture of DCM:MeOH (28 mL:4 mL, 0.09 M in relation to **7a**) and cooled to −78°C. Ozone is bubbled to the solution until a light blue color appeared (in approx. 10 minutes). Then, the solution is purged with oxygen and nitrogen until the excess of ozone is dissipated and the blue color diminishes. Then, PPh_3 (943 mg, 3.6 mmol, 1.2 equiv.) is added and the reaction is allowed to slowly warm up from −78 °C to room temperature over 2 hours. Finally, the solvents are removed under reduced pressure and the reaction mixture is purified by flash column chromatography (SiO_2, DCM) to afford the title compound as a colorless oil: 478 mg, 87% yield.[9]

^{1}H NMR (250 MHz, $CDCl_3$) δ: 9.74 (t, *J* = 1.3 Hz, 1H), 3.51 (t, *J* = 6.4 Hz, 2H), 2.70 (s, 4H), 2.47 (t, *J* = 6.4 Hz, 2H), 1.63 – 1.58 (m, 4H).
^{13}C{^{1}H} NMR (62.5 MHz, $CDCl_3$) δ: 201.8, 177.2, 43.1, 38.2, 28.1, 27.0, 19.1.
IR (neat, ATR): 2943, 2252, 1693, 1404, 1153 cm^{-1}.
HRMS (ESI-ToF) m/z: $[M + H]^+$ Calcd. for $C_9H_{14}NO_3$: 184.0968, found: 184.0968.

1-(4-(1,3-dioxolan-2-yl)butyl)pyrrolidine-2,5-dione (9a)

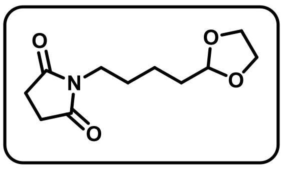

Under air, a stirred solution of 5-(2,5-dioxopyrrolidin-1-yl)pentanal **8a** (366 mg, 2 mmol, 1 equiv.), pTSA·H_2O (38 mg, 0.2 mmol, 0.10 equiv.), ethylene glycol (223 µL, 4 mmol, 2 equiv.) and toluene (20 mL, 0.1 M) is

[9] ^{1}H NMR is in good agreement with the literature. See: Sui, G.; Lv, Q.; Song, X.; Guo, H.; Dai, J.; Ren, L.; Lee, C.-S.; Zhou, W.; Hao, H.-D.; *New J. Chem.*, **2019**, *43*, 15793-15796.

heated to reflux (110 ºC) for 24 hours using a Dean-Stark apparatus. The reaction is quenched with a saturated aqueous solution of $NaHCO_3$, extracted with AcOEt (3x), dried ($MgSO_4$) and concentrated under reduced pressure. Purification by flash column chromatography (SiO_2, gradient: Hex – 9:1 Hex:AcOEt – 8:2 Hex:AcOEt – 1:1 Hex:AcOEt) affords the title product as an yellowish oil: 287 mg, 63%.

^{1}H NMR (250 MHz, $CDCl_3$) δ: 4.83 (t, *J* = 4.7 Hz, 1H), 3.96 – 3.90 (m, 2H), 3.87 – 3.81 (m, 2H), 3.50 (t, *J* = 7.4 Hz, 2H), 2.68 (s, 4H), 1.68 – 1.57 (m, 4H), 1.45 – 1.41 (m, 2H).
^{13}C{^{1}H} NMR (62.5 MHz, $CDCl_3$) δ: 177.2, 104.2, 64.8, 38.7, 33.3, 28.1, 27.6, 21.3.
IR (neat, ATR): 2935, 2615, 1766, 1708, 1627, 1392, 1253, 1006 cm^{-1}.
HRMS (ESI-ToF) m/z: [M + H]$^+$ Calcd. for $C_{11}H_{18}NO_4$: 228.1230, found: 228.1231.

1-(hex-5-en-1-yl)piperidine-2,6-dione (7b)

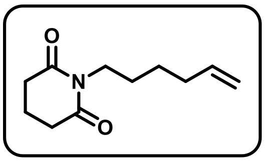

The procedure employed here was originally reported by Martin and coworkers.[8] Under N_2, at room temperature, a round bottom flask is charged with glutarimide **1b** (565 mg, 5 mmol, 1 equiv.), dry THF (30 mL, 0.16 M, dried from SPS), PPh_3 (1.70 g, 6.5 mmol, 1.3 equiv.) and hex-5-en-1-ol **6** (720 µL, 6 mmol, 1.2 equiv.). The reaction temperature is cooled down to 0 ºC, and diisopropyl azodicarboxylate (1.28 mL, 6.5 mmol, 1.3 equiv.) is slowly added. The temperature is allowed to warm up to room temperature and the reaction is stirred at this temperature for 24 hours, then being concentrated under reduced pressure. Purification by flash column chromatography (SiO_2, gradient: Hex – 95:5 Hex:AcOEt – 9:1 Hex:AcOEt) affords the title compound as a yellowish oil: 634 mg, 65%.[10]

^{1}H NMR (250 MHz, $CDCl_3$) δ: 5.85 – 5.69 (m, 1H), 5.02 – 4.90 (m, 2H), 3.74 (t, *J* = 7.5 Hz, 2H), 2.63 (t, *J* = 6.6 Hz, 4H), 2.07 – 2.01 (m, 2H), 1.97 – 1.86 (m, 2H), 1.57 – 1.45 (m, 2H), 1.43 – 1.36 (m, 2H).
^{13}C{^{1}H} NMR (62.5 MHz, $CDCl_3$) δ: 172.4, 138.5, 114.6, 39.4, 33.3, 32.9, 27.5, 26.2, 17.2.
IR (neat, ATR): 3062, 2985, 2939, 1735, 1674, 1442, 1373, 1238, 1091, 1045 cm^{-1}.
HRMS (ESI-ToF) m/z: [M + H]$^+$ Calcd. for $C_{11}H_{18}NO_2$: 196.1332, found: 196.1328.

5-(2,6-dioxopiperidin-1-yl)pentanal (8b)

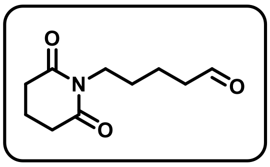

1-(hex-5-en-1-yl)piperidine-2,6-dione **7b** (585 mg, 3 mmol, 1 equiv.) is dissolved in a 7:1 mixture DCM:MeOH (28 mL:4 mL, 0.09M in relation to **7b**) and cooled to −78°C. Ozone is bubbled to the solution until a light blue color appeared (approx. In 10 minutes). Then, the solution is purged with oxygen and nitrogen until the excess ozone is dissipated and the blue color fades. Then, PPh_3 (943 mg, 3.6 mmol, 1.2 equiv.) is added, and the reaction is allowed to slowly warm up from −78°C to room temperature over 2 hours. Finally, the solvents are removed under reduced pressure and the residue is purified by flash column chromatography (SiO_2, DCM) to afford the title compound as a colorless oil: 284 mg, 48% yield.

^{1}H NMR (250 MHz, $CDCl_3$) δ: 9.73 (t, *J* = 1.7Hz, 1H), 3.75 (t, *J* = 7.2 Hz, 2H), 2.62 (t, *J* = 6.6 Hz, 4H), 2.44 (t, *J* = 7.2 Hz, 2H), 1.97 – 1.86 (m, 2H), 1.63 – 1.50 (m, 4H).
^{13}C{^{1}H} NMR (62.5 MHz, $CDCl_3$) δ: 202.1, 172.5, 43.3, 38.9, 32.8, 27.4, 19.2, 17.1.
IR (neat, ATR): 1728, 1670, 1045 cm^{-1}.
HRMS (ESI-ToF) m/z: [M + H]$^+$ Calcd. for $C_{10}H_{16}NO_3$: 198.1125, found: 198.1117.

1-(4-(1,3-dioxolan-2-yl)butyl)piperidine-2,6-dione (9b)

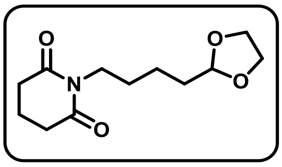

Under air, a stirred solution of 5-(2,6-dioxopiperidin-1-yl)pentanal **8b** (268 mg, 1.36 mmol, 1 equiv.), *p*TSA·H_2O (27 mg, 0.14 mmol, 0.10 equiv.), ethylene glycol (152 µL, 2.72 mmol, 2 equiv.) and toluene (14 mL, 0.1 M) is heated to reflux (110 ºC) for 24 hours using a Dean-Stark apparatus. The reaction is quenched with a saturated aqueous solution of $NaHCO_3$, extracted with AcOEt (3x), dried ($MgSO_4$) and concentrated under reduced pressure. Purification by flash column chromatography (SiO_2, gradient: Hex – 9:1 Hex:AcOEt – 8:2 Hex:AcOEt – 1:1 Hex:AcOEt) affords the title compound as an yellowish oil: 236 mg, 66%.

^{1}H NMR (250 MHz, $CDCl_3$) δ: 4.82 (t, *J* = 4.8 Hz, 1H), 3.94 – 3.87 (m, 2H), 3.84 – 3.78 (m, 2H), 3.77 – 3.71 (m, 2H), 2.62 (t, *J* = 6.6 Hz, 4H), 1.93 – 1.85 (m, 2H), 1.69 – 1.59 (m, 2H), 1.57 – 1.48 (m, 2H), 1.45 – 1.34 (m, 2H).
^{13}C{^{1}H} NMR (62.5 MHz, $CDCl_3$) δ: 172.4, 104.3, 64.8, 39.4, 33.4, 32.8, 27.8, 21.4, 17.1.
IR (neat, ATR): 2966, 2885, 1720, 1666, 1442, 1354, 1114, 1049 cm^{-1}.
HRMS (ESI-ToF) m/z: [M + Na]$^+$ Calcd. for $C_{12}H_{19}NO_4Na$: 264.1206, found: 264.1208.

Hexahydroindolizin-3(2H)-one (12a)

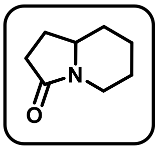

At room temperature, under air, a round bottom flask is charged with 1,2,5,6-tetrahydroindolizine-3,7-dione **4a** (76 mg, 0.5 mmol, 1 equiv.) and anhydrous AcOEt (5 mL, 0.1 M in relation to **4a**, dried in 3Å MS). Then, 10% Pd/C (50 mg, 100 mg/ mmol of **4a**) is added. The flask is closed with a rubber septum and a balloon filled with H_2 is inserted. The reaction is allowed to stir at room temperature overnight. Then, the reaction mixture is filtered through a pad of celite while eluting with AcOEt; and the resulting solution is concentrated under reduced pressure. Purification by flash column chromatography (SiO_2, gradient: 8:2 Hex:AcOEt – 1:1 Hex:AcOEt – AcOEt) affords the title compound as a colorless oil: 49 mg, 70%.[10]

^{1}H NMR (250 MHz, $CDCl_3$) δ: 4.13 – 4.06 (m, 1H), 3.39 (dtd, *J* = 10.7 Hz, *J* = 7.4 Hz, *J* = 3.2 Hz, 1H), 2.61 (td, *J* = 13.0 Hz, *J* = 3.2 Hz, 1H), 2.38 – 2.31 (m, 2H), 2.19 (dt, *J* = 13.0 Hz, *J* = 7.4 Hz, 1H), 1.91 – 1.82 (m, 2H), 1.74 – 1.64 (m, 1H), 1.62 – 1.51 (m, 1H), 1.44 – 1.26 (m, 2H), 1.23 – 1.09 (m, 1H).

^{13}C{^{1}H} NMR (62.5 MHz, $CDCl_3$) δ: 173.7, 57.3, 40.2, 33.5, 30.3, 25.3, 24.4, 23.6.

IR (neat, ATR): 2933, 2853, 1657,1445, 1423,1674, 1372, 1313, 1272 cm^{-1}.

HRMS (ESI-ToF) m/z: [M + H]$^+$ Calcd. for $C_8H_{14}NO$: 140.1070, found: 140.1070.

(±)-Coniceine (13a)

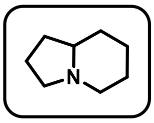

At room temperature, under N_2, a round bottom flask is charged with hexahydroindolizin-3(2H)-one **12a** (21 mg, 0.15 mmol, 1 equiv.) and dry Et_2O (1.5 mL, 0.1M, dried from SPS). Then, the reaction mixture is cooled down to 0 ºC and a solution of $LiAlH_4$ (1M in THF, 300 μL, 2 equiv.) is slowly added. Then, the temperature of the reaction mixture is allowed to warm up to room temperature and the resulting reaction mixture is stirred at this temperature overnight. Then, the reaction is cooled to 0°C, and the reaction is quenched following the Fieser protocol: sequential addition of H_2O (15 µL), 15% w/w $NaOH_{(aq)}$ (15 µL), H_2O (45 µL). At this point, the reaction mixture is allowed to stir at room temperature for additional 30 min and $MgSO_4$ is added. The mixture is filtered through a pad of celite, while eluting with Et_2O and carefully concentrated under vacuum to cleanly afford the title compound

[10] ^{1}H and ^{13}C NMRs are in good agreement with the literature. See: Santiago, J. V.; Burtoloso, A. C. B., *Eur. J. Org. Chem.* **2018,** *2018*, 2822-2830.

as a pale yellow oil: 15 mg, 79%. (*Note:* the reaction seems to be quantitative, but product is volatile).[11]

^{1}H NMR (250 MHz, $CDCl_3$) δ: 3.10 – 2.99 (m, 2H), 2.05 (app q, *J* = 9.0 Hz, 1H), 1.95 (dt, *J* = 11.4 Hz, *J* = 3.3 Hz, 1H), 1.82 – 1.74 (m, 3H), 1.73 – 1.68 (m, 2H), 1.67 – 1.60 (m, 2H), 1.59 – 1.51 (m, 1H), 1.42 – 1.32 (m, 1H), 1.26 – 1.19 (m, 2H).
^{13}C{^{1}H} NMR (62.5 MHz, $CDCl_3$) δ: 64.4, 54.3, 53.1, 31.1, 30.5, 25.5, 24.5, 20.6.
IR (neat, ATR): 2952, 2920, 1461, 1454, 1320, 1255, 1096 cm^{-1}.
HRMS (ESI-ToF) m/z: $[M + H]^+$ Calcd. for $C_8H_{16}N$: 126.1277, found: 126.1276.

Octahydro-4H-quinolizin-4-one (12b) and Hexahydro-2H-quinolizine-2,6(1H)-dione (12b')
At room temperature, under air, a round bottom flask is charged with 3,4,8,9-tetrahydro-2H-quinolizine-2,6(7H)-dione **4b** (83 mg, 0.5 mmol, 1 equiv.) and anhydrous AcOEt (5 mL, 0.1 M in relation to **4b**, dried in 3Å MS). Then, 10% Pd/C (50 mg, 100 mg/ mmol of **4b**) is added. The flask is closed with a rubber septum and a balloon filled with H_2 is inserted. The reaction is allowed to stir at room temperature overnight. Then, the reaction mixture is filtered through a pad of celite while eluting with AcOEt; and the resulting solution is concentrated under reduced pressure. Purification by flash column chromatography (SiO_2, gradient: 8:2 Hex:AcOEt – 1:1 Hex:AcOEt – AcOEt) affords amide **12b** as a colorless oil: 54 mg, 70%;[12] and the ketone **12b'** as a colorless oil: 8 mg, 10% yield.[13]

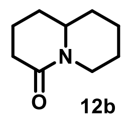
12b

^{1}H NMR (400 MHz, $CDCl_3$) δ: 4.80 – 4.73 (m, 1H), 3.24 – 3.17 (m, 1H), 2.43 – 2.30 (m, 3H), 1.98 – 1.93 (m, 1H), 1.86 – 1.75 (m, 2H), 1.73 – 1.62 (m, 3H), 1.53 – 1.18 (m, 4H).
^{13}C{^{1}H} NMR (100 MHz, $CDCl_3$) δ: 169.3, 56.9, 42.4, 34.0, 33.0, 30.5, 25.3, 24.5, 19.2.
IR (neat, ATR): 3010, 2945, 2860, 1620, 1479, 1452, 1283 cm^{-1}.
HRMS (ESI-ToF) m/z: $[M + H]^+$ Calcd. for $C_9H_{16}NO$: 154.1226, found: 154.1231.

---

[11] ^{1}H and ^{13}C NMRs are in good agreement with the literature. See: Sibi, M. P.; Christensen, J. W., *J. Org. Chem.* **1999,** *64*, 6434-6442.
[12] ^{1}H and ^{13}C NMRs are in good agreement with the literature. See: Yu, H.; Zhang, G.; Huang, H., *Angew. Chem. Int. Ed.* **2015,** *54*, 10912-10916.
[13] ^{1}H and ^{13}C NMRs are in good agreement with the literature. See: Gade, A. B.; Patil, N. T., *Org. Lett.* **2016,** *18*, 1844-1847.

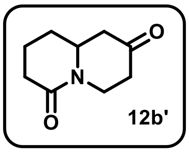

^{1}H NMR (250 MHz, CDCl$_3$) δ: 4.95 – 4.86 (m, 1H), 3.75 – 3.64 (m, 1H), 2.96 – 2.85 (m, 1H), 2.50 – 2.40 (m, 6H), 2.13 – 2.04 (m, 1H), 1.93 – 1.84 (m, 1H), 1.82 – 1.73 (m, 1H), 1.63 – 1.56 (m, 1H).

^{13}C{^{1}H} NMR (62.5 MHz, CDCl$_3$) δ: 206.9, 169.5, 54.9, 48.1, 41.0, 40.6, 32.7, 29.7, 18.8.

IR (neat, ATR): 2951, 1712, 1616, 1469, 1446, 1334 cm^{-1}.

HRMS (ESI-ToF) m/z: [M + H]$^+$ Calcd. for $C_9H_{14}NO_2$: 168.1019, found: 168.1017.

Quinolizidine (13b)

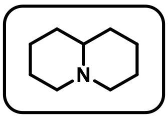

At room temperature, under N_2, a round bottom flask is charged with octahydro-4H-quinolizin-4-one **12b** (23 mg, 0.15 mmol, 1 equiv.) and dry Et_2O (1.5 mL, 0.1M, dried from SPS). Then, the reaction mixture is cooled down to 0 ^{o}C and a solution of $LiAlH_4$ (1M in THF, 300 μL, 2 equiv.) is slowly added. Then, the temperature of the reaction mixture is allowed to warm up to room temperature and the resulting reaction mixture is stirred at this temperature overnight. Then, the reaction is cooled to 0°C, and the reaction is treated following the Fieser work-up: sequential addition of H_2O (15 µL), 15% w/w $NaOH_{(aq)}$ (15 µL), H_2O (45 µL). At this point, the reaction mixture is allowed to stir at room temperature for additional 30 min and $MgSO_4$ is added. The mixture is filtered through a pad of celite, while eluting with Et_2O and is carefully concentrated under vacuum to cleanly afford the title compound as a colorless oil: 20 mg, 96%.[14]

^{1}H NMR (250 MHz, CDCl$_3$) δ: 2.79 – 2.74 (m, 2H), 2.02 – 1.90 (m, 2H), 1.74 – 1.44 (m, 9H), 1.33 – 1.19 (m, 4H).

^{13}C{^{1}H} NMR (62.5 MHz, CDCl$_3$) δ: 63.0, 56.6, 33.4, 25.8, 24.6.

IR (neat, ATR): 2931, 2854, 1450, 1242, 1226, 1165, 1099, 1029 cm^{-1}.

HRMS (ESI-ToF) m/z: [M + H]$^+$ Calcd. for $C_9H_{18}N$: 140.1434, found: 140.1438.

1,3,4,10b-tetrahydropyrido[2,1-a]isoindol-6(2H)-one (12c)

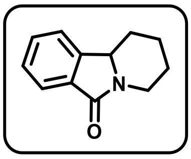

At room temperature, under air, a round bottom flask is charged with 3,4-dihydropyrido[2,1-a]isoindole-2,6-dione **4e** (80 mg, 0.4 mmol, 1 equiv.) and

[14] ^{1}H and ^{13}C NMRs are in good agreement with the literature. See: Tehrani, K. A.; D'hooghe, M.; De Kimpe, N.; *Tetrahedron*, **2003**, *59*, 3099-3108.

anhydrous AcOEt (4 mL, 0.1 M in relation to **4e**, dried in 3Å MS). Then, 10% Pd/C (40 mg, 100 mg/ mmol of **4e**) is added. The flask is closed with a rubber septum and a balloon filled with H_2 is inserted. The reaction is allowed to stir at room temperature overnight. Then, the reaction mixture is filtered through a pad of celite while eluting with AcOEt; and the resulting solution is concentrated under reduced pressure. Then, a procedure reported by Routier and coworkers is employed.[15] At room temperature, under N_2, a round bottom flask is charged with Na (25 mg, 1.1 mmol, 2.75 equiv.) and ethylene glycol (3 mL, 0.13M in relation to **4e**). Then, under N_2, at room temperature, another round bottom flask is charged with the previously obtained crude reaction mixture and $H_2NNH_2 \cdot H_2O$ 55% (131 μL, 1.48 mmol, 3.7 equiv.) and is transferred using a syringe to the first solution. The resulting mixture is refluxed at 140°C for 4 h, then being allowed to cool down to room temperature. At this point, the reaction mixture is diluted in DCM and washed with 1M aqueous solution of NaOH, dried ($MgSO_4$) and concentrated under reduced pressure. Purification by flash column chromatography (SiO_2, gradient: DCM – 9:1 DCM:AcOEt) affords the title compound as a colorless oil: 29 mg, 39%.[16]

^{1}H NMR (400 MHz, $CDCl_3$) δ: 7.85 (d, *J* = 7.4 Hz, 1H), 7.51 (t, *J* = 7.4 Hz, 1H), 7.44 (t, *J* = 7.4 Hz, 1H), 7.41 (d, *J* = 7.4 Hz, 1H), 4.48 (dd, *J* = 13.2 Hz, *J* = 5.0 Hz, 1H), 4.26 (dd, *J* = 11.8 Hz, *J* = 3.7 Hz, 1H), 2.97 (td, *J* = 13.2 Hz, *J* = 3.7 Hz, 1H), 2.38 – 2.32 (m, 1H), 2.02 – 1.97 (m, 1H), 1.85 – 1.78 (m, 1H), 1.66 (qt, *J* = 13.2 Hz, *J* = 3.3 Hz, 1H), 1.45 – 1.33 (m, 1H), 1.17 – 1.07 (m, 1H).

^{13}C{^{1}H} NMR (100 MHz, $CDCl_3$) δ: 166.1, 145.7, 132.4, 131.0, 128.0, 123.6, 121.6, 58.9, 39.6, 31.7, 25.2, 23.6.

IR (neat, ATR): 2927, 1666, 1616, 1269, 1284 cm^{-1}.

HRMS (ESI-ToF) m/z: [M + Na]$^+$ Calcd. for $C_{12}H_{13}NONa$: 210.0889, found: 210.0892.

---

[15] Boulahjar, R.; Ouach, A.; Matteo, C.; Bourg, S.; Ravache, M.; Guével, R. l.; Marionneau, S.; Oullier, T.; Lozach, O.; Meijer, L.; Guguen-Guillouzo, C.; Lazar, S.; Akssira, M.; Troin, Y.; Guillaumet, G.; Routier, S., *J. Med. Chem.* **2012,** *55*, 9589-9606.

[16] ^{1}H and ^{13}C NMRs are in good agreement with the literature. See: Ali, I. A. I.; *Monatsch. Chem.*, **2014**, *145*, 803-810.

4. NMR Spectra

Molecule **3a**: ^{1}H NMR (250 MHz, $CDCl_3$)

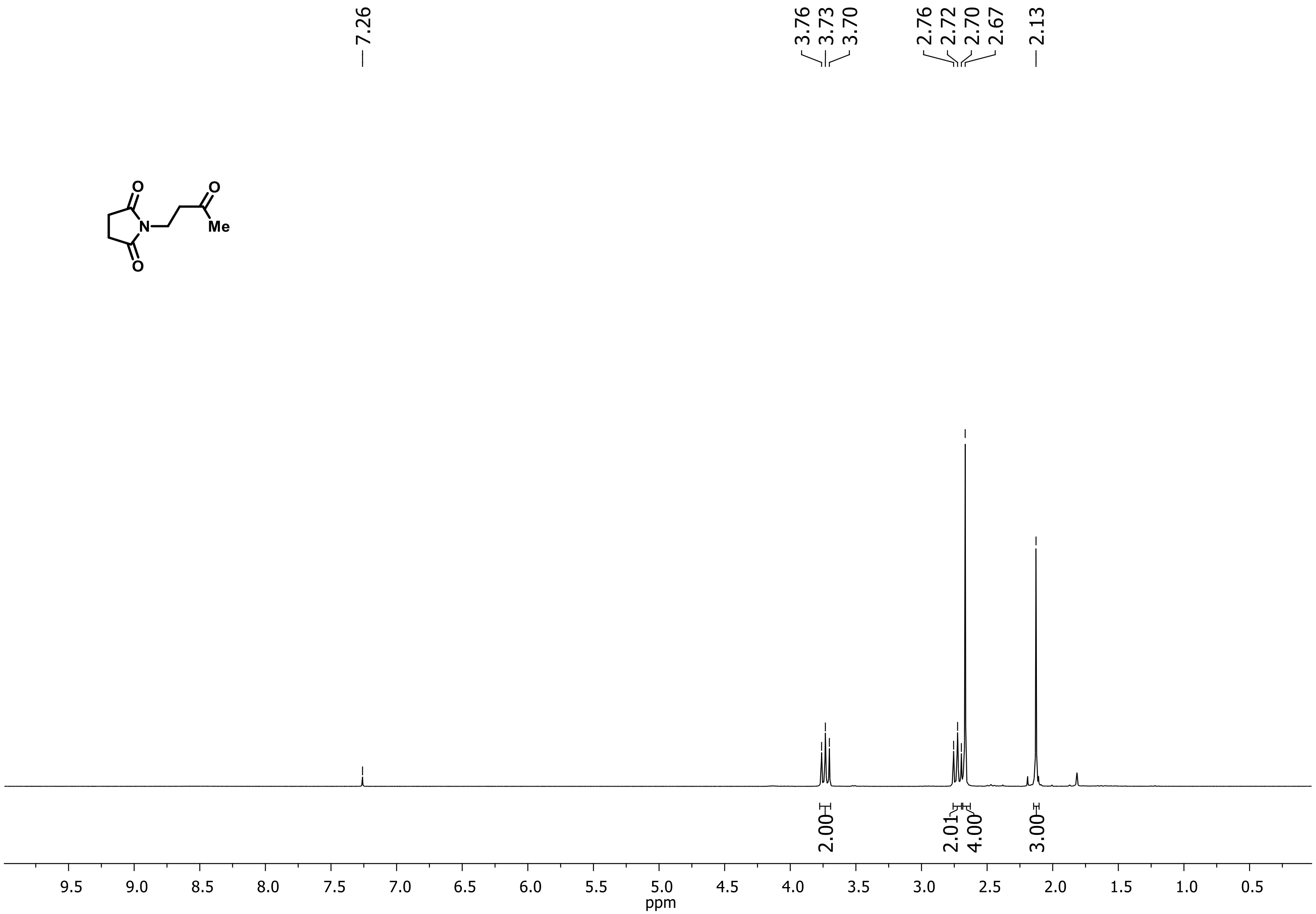

Molecule **3a**: $^{13}C\{^{1}H\}$ NMR (62.5 MHz, $CDCl_3$)

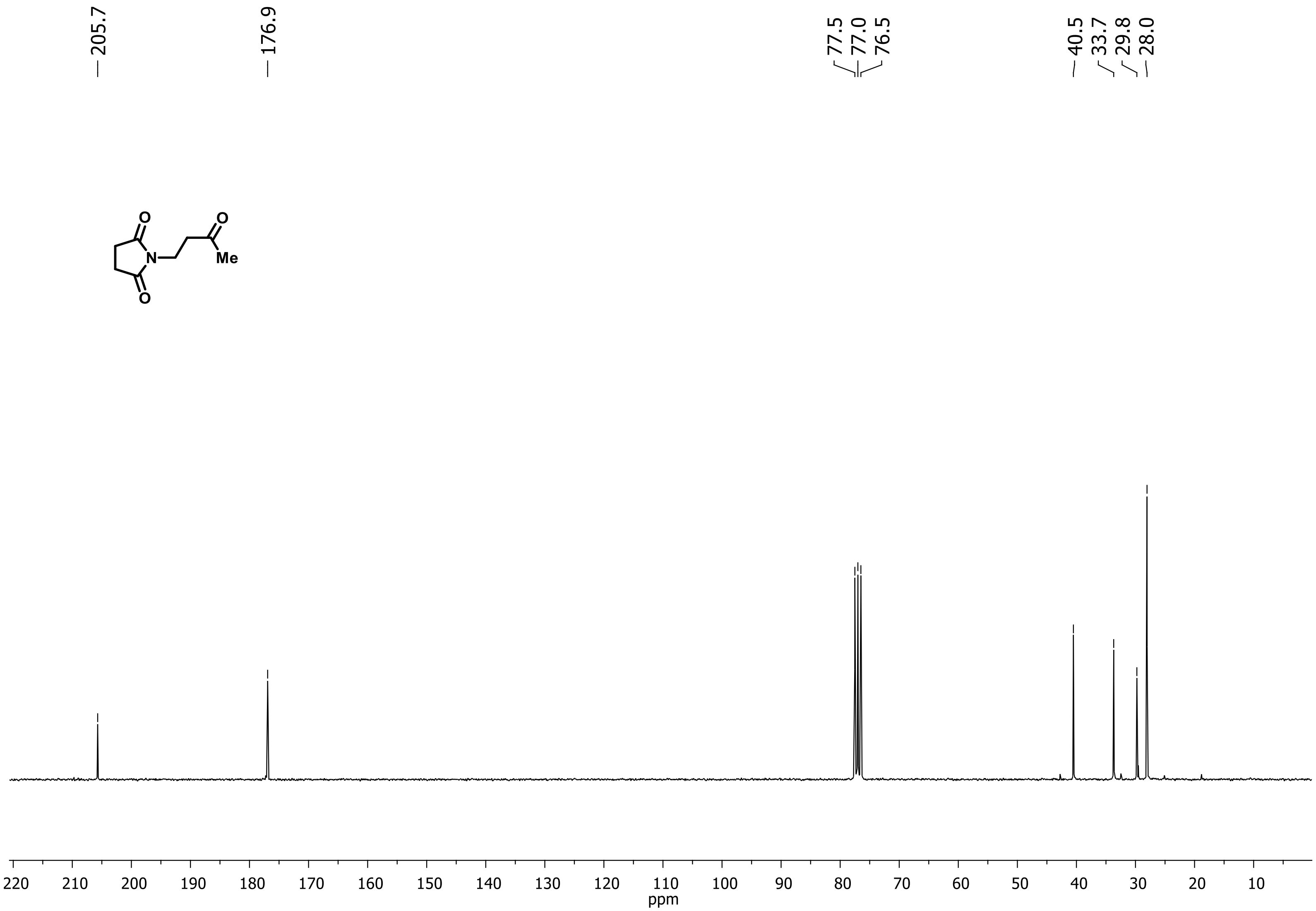

Molecule **4a**: ^{1}H NMR (250MHz, $CDCl_3$)

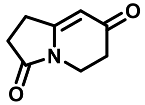

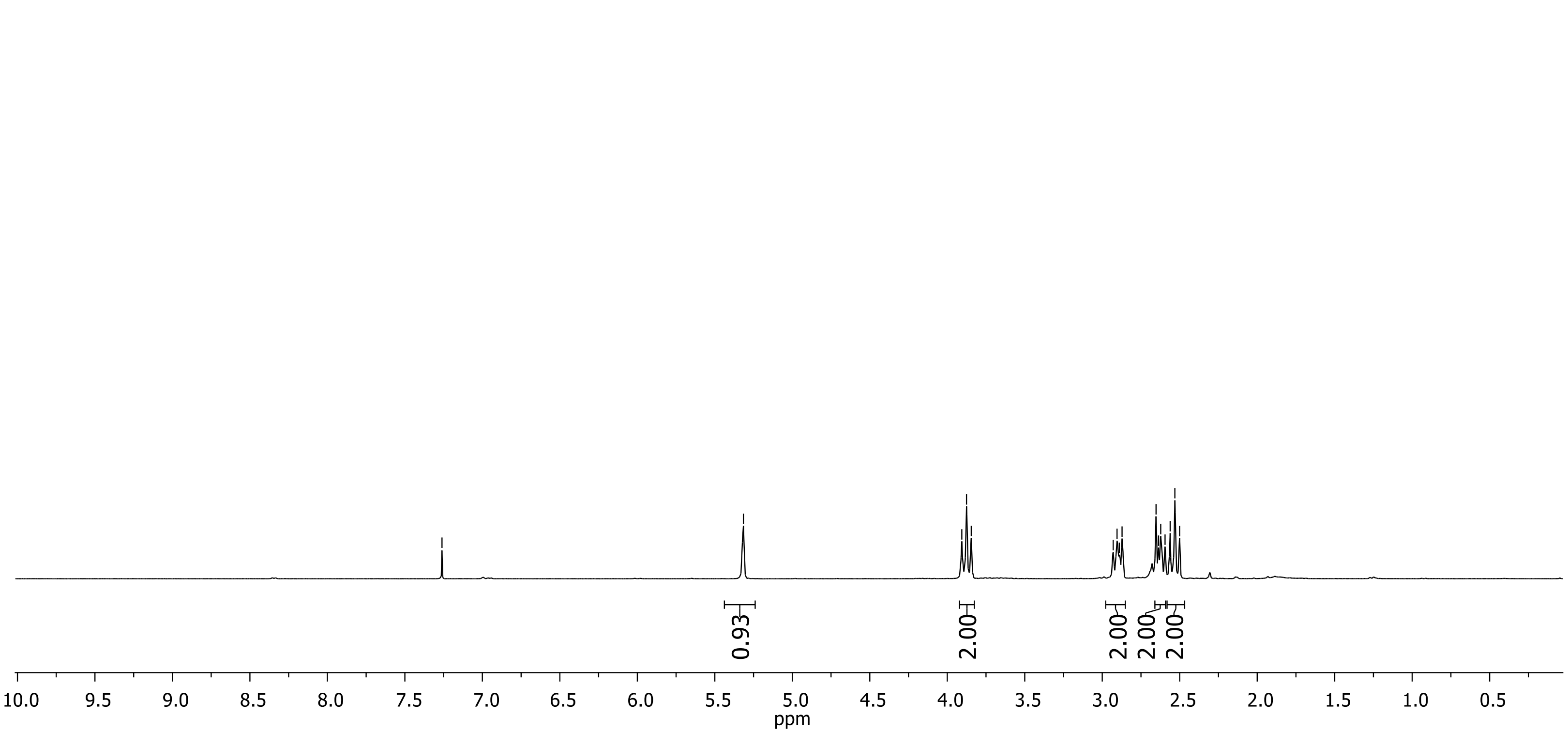

Molecule **4a**: $^{13}C\{^{1}H\}$ NMR (62.5 MHz, $CDCl_3$)

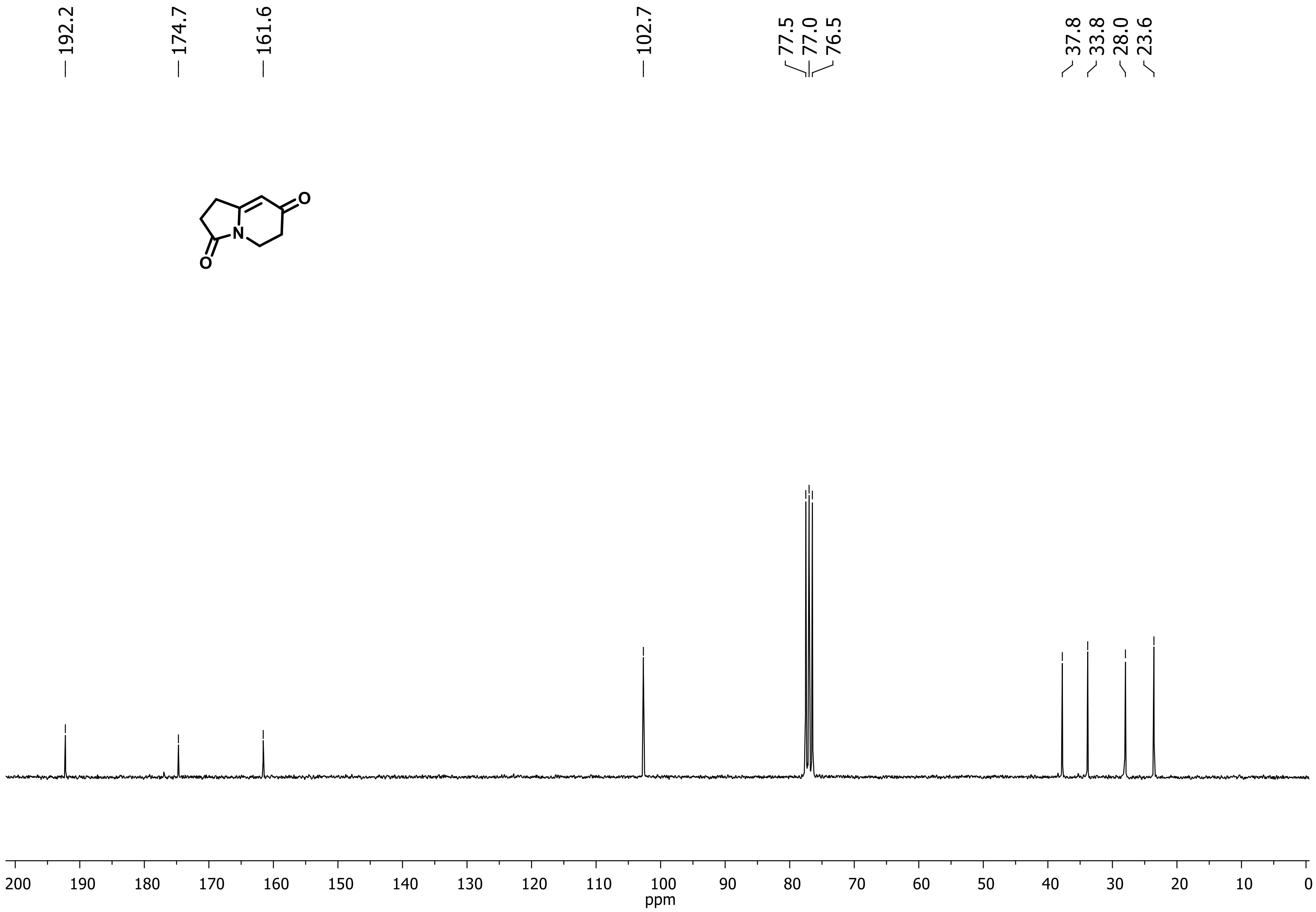

Molecule **3b**: ^{1}H NMR (250 MHz, $CDCl_3$)

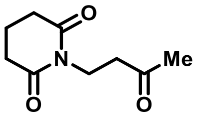

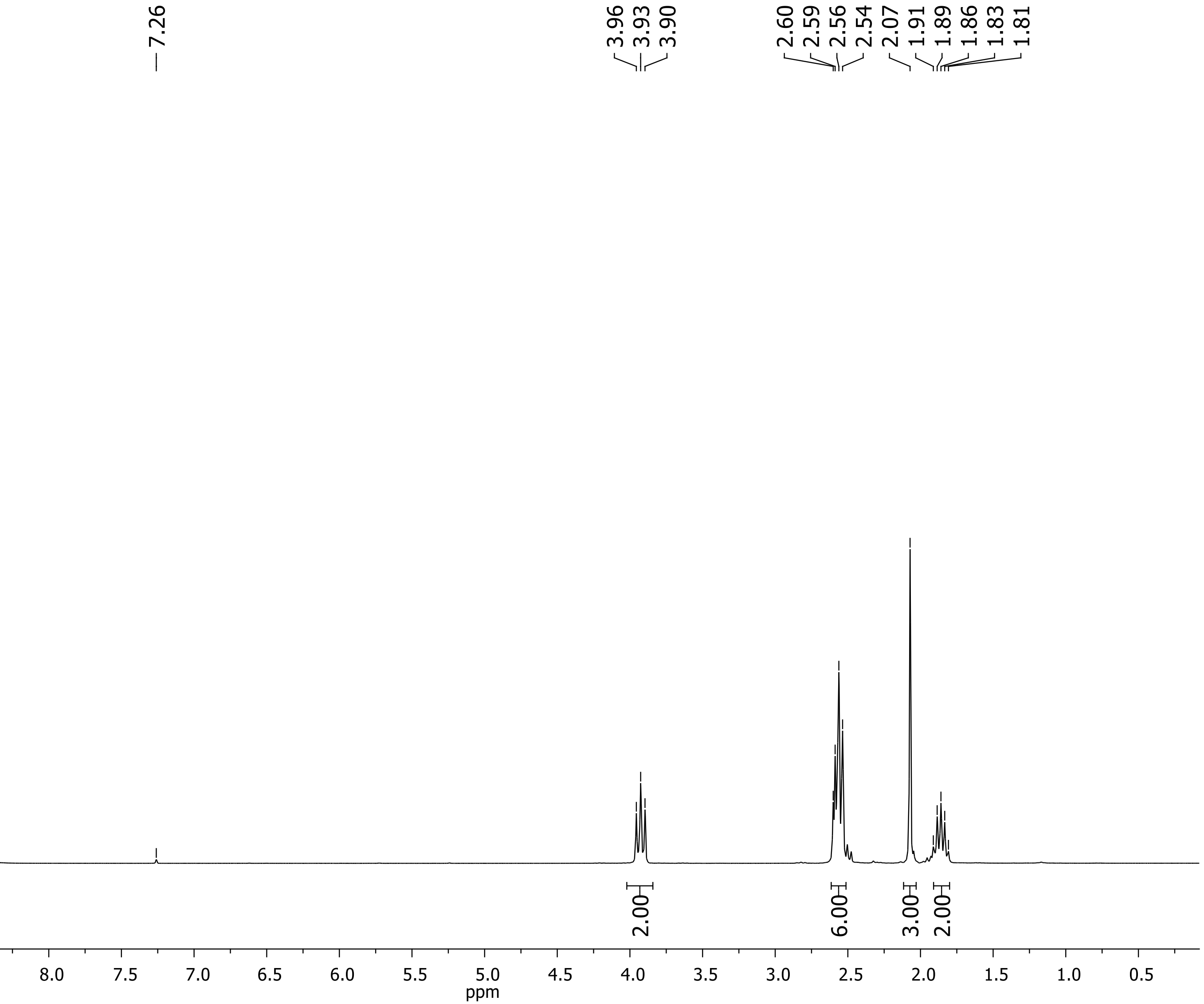

Molecule **3b**: $^{13}C\{^1H\}$ NMR (62.5 MHz, $CDCl_3$)

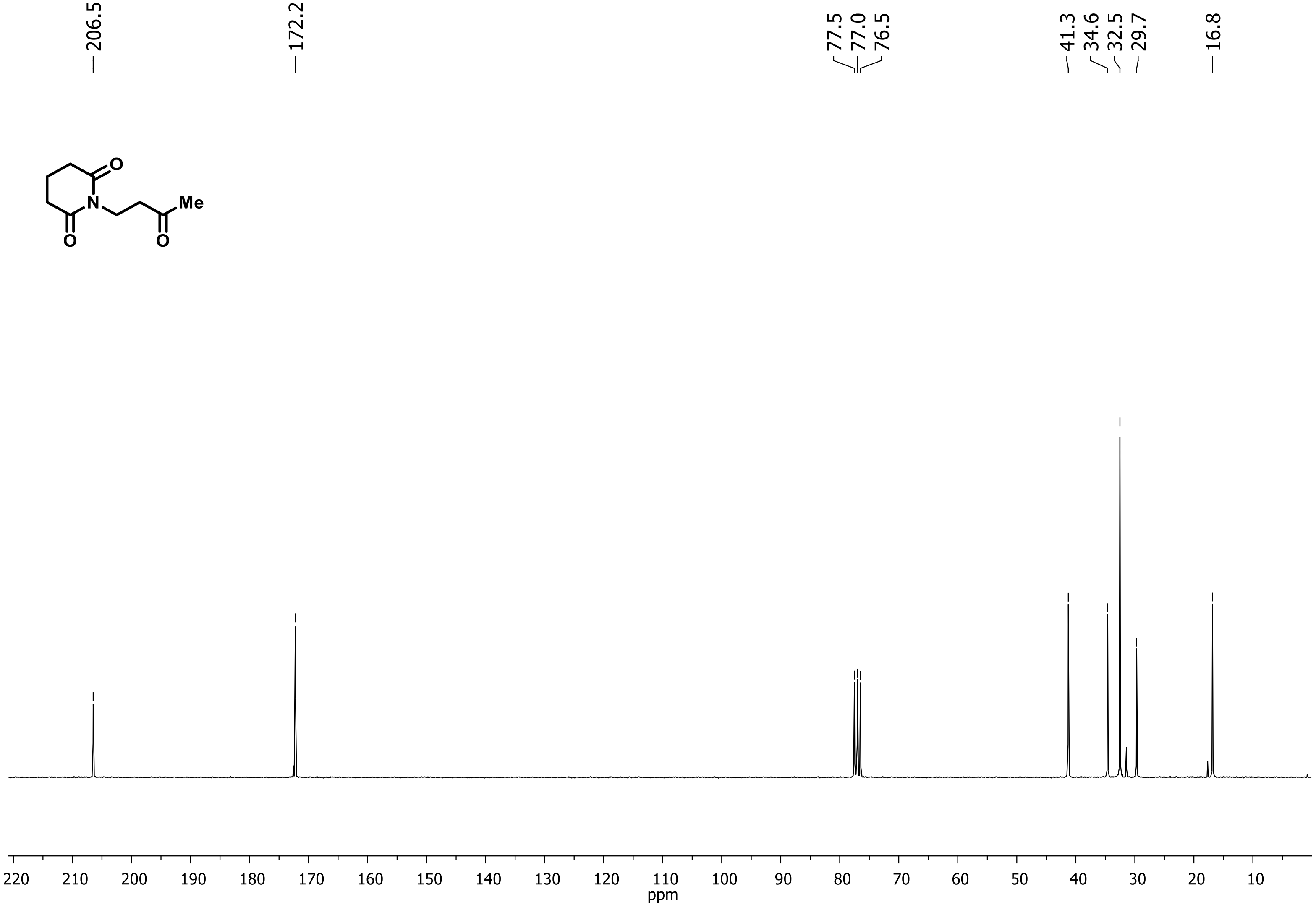

Molecule **4b**: ^{1}H NMR (250 MHz, $CDCl_3$)

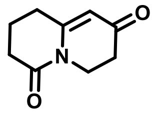

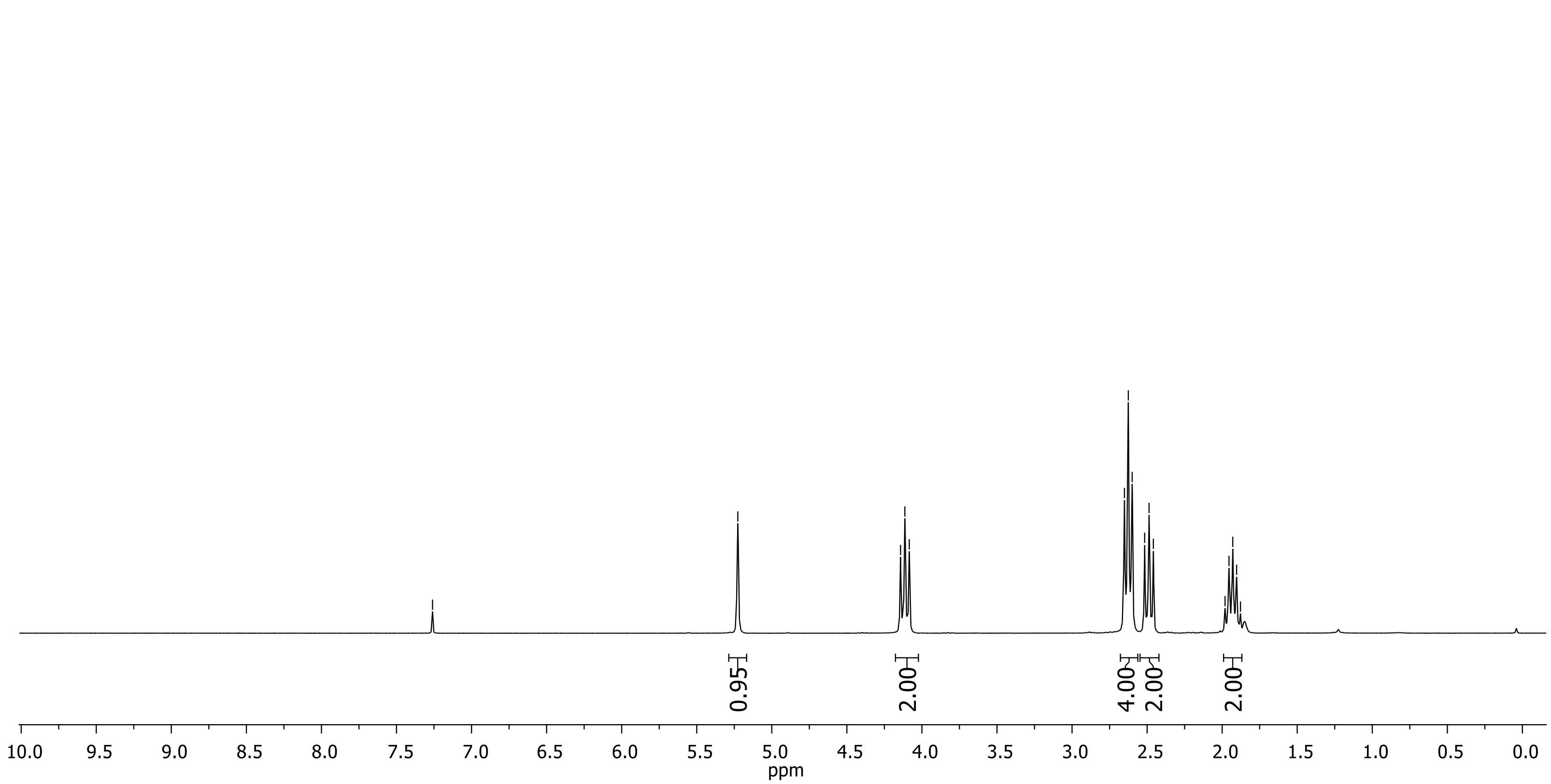

Molecule **4b**: $^{13}C\{^1H\}$ NMR (62.5 MHz, $CDCl_3$)

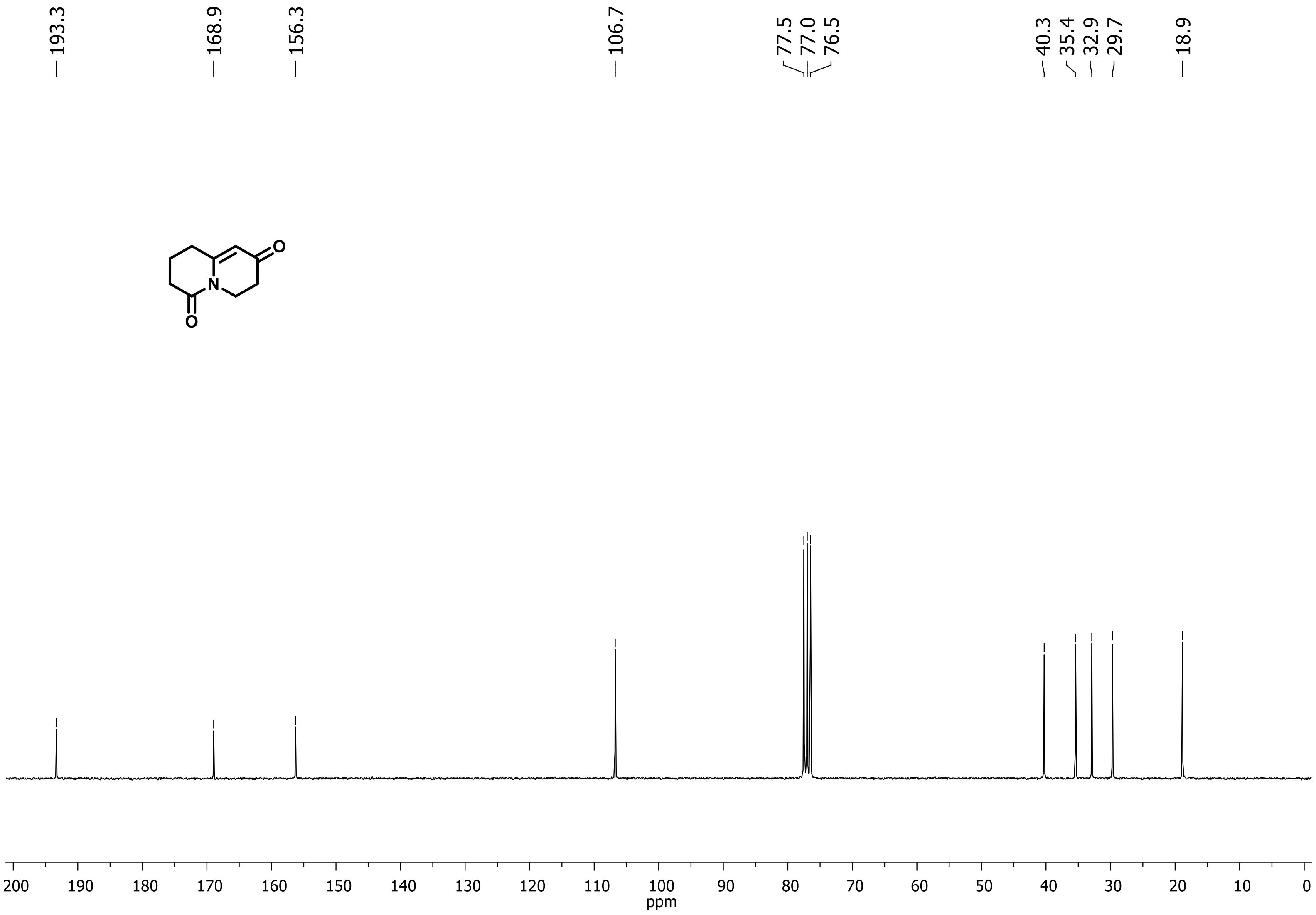

Molecule **3c**: ^{1}H NMR (400 MHz, $CDCl_3$)

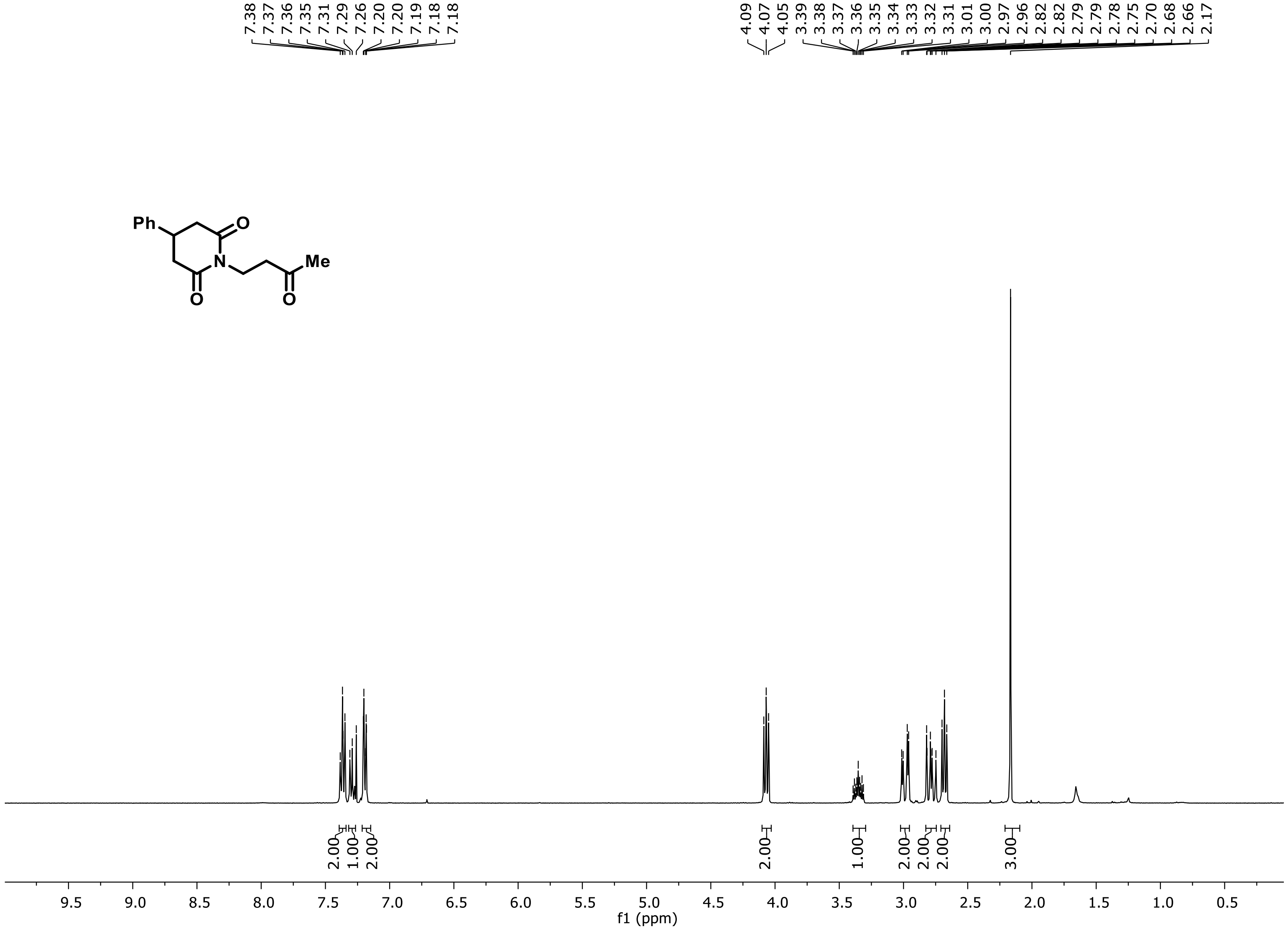

Molecule **3c**: $^{13}C\{^1H\}$ NMR (100 MHz, $CDCl_3$)

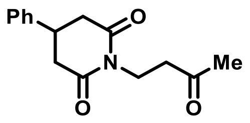

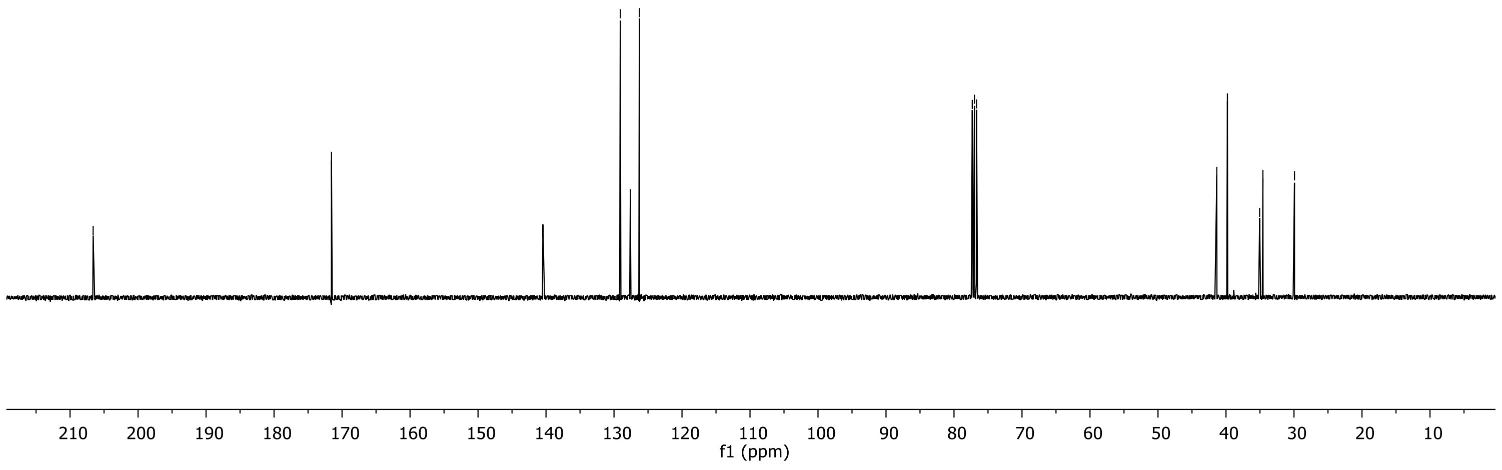

Molecule **4c**: ^{1}H NMR (400 MHz, $CDCl_3$)

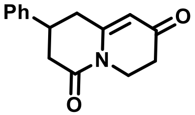

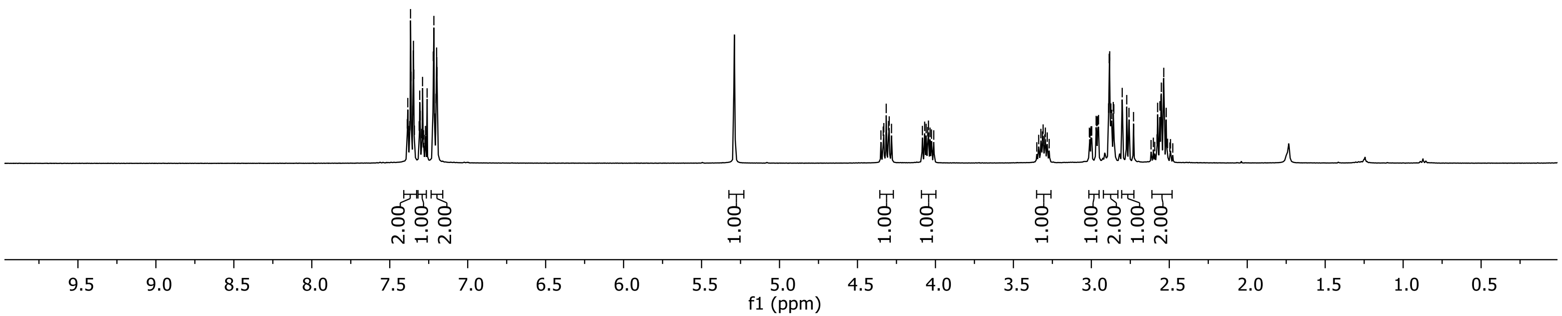

Molecule **4c**: $^{13}C\{^{1}H\}$ NMR (100 MHz, $CDCl_3$)

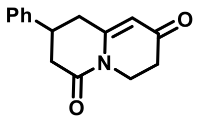

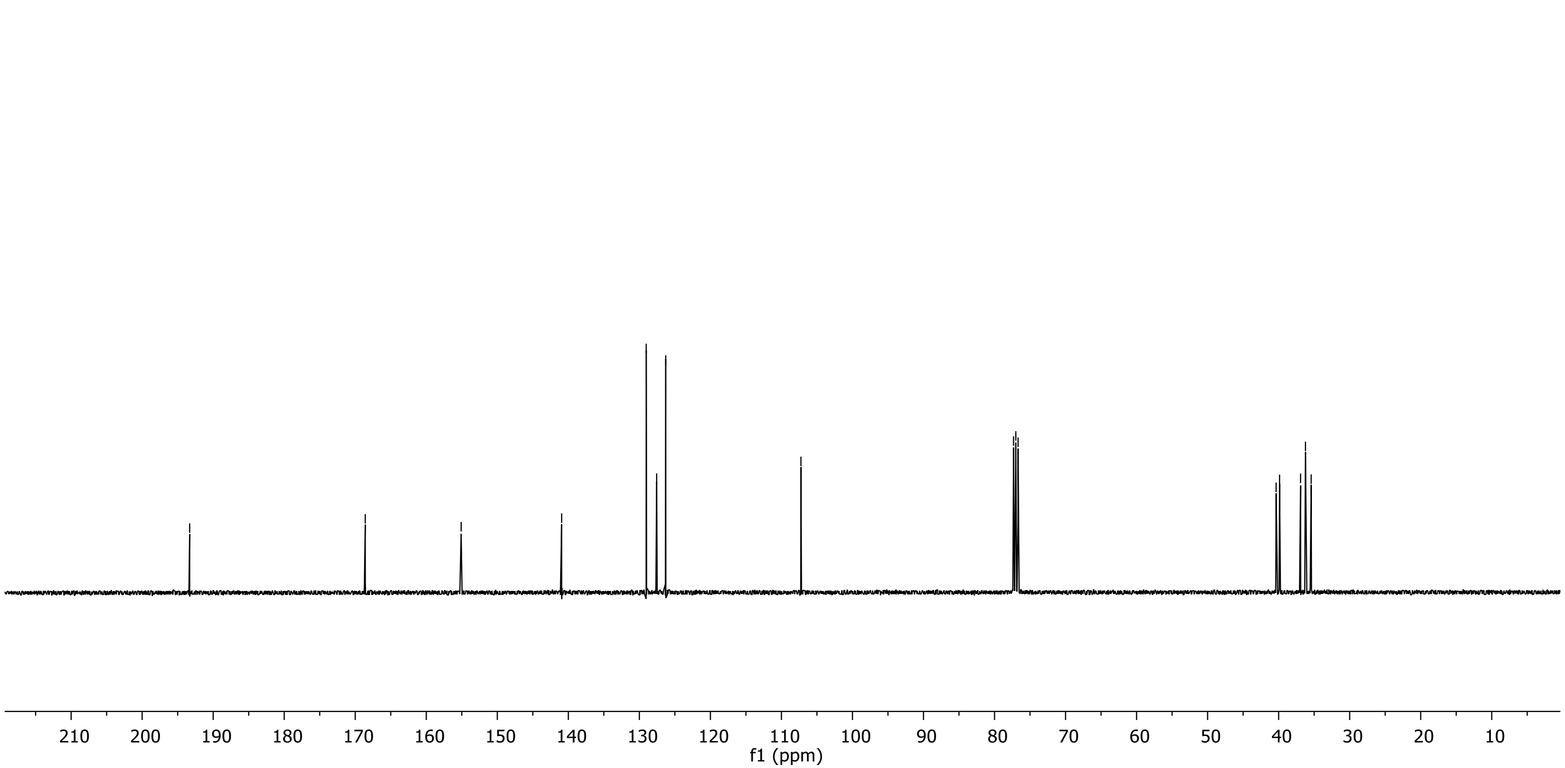

Molecule **3d**: ^{1}H NMR (400 MHz, $CDCl_3$)

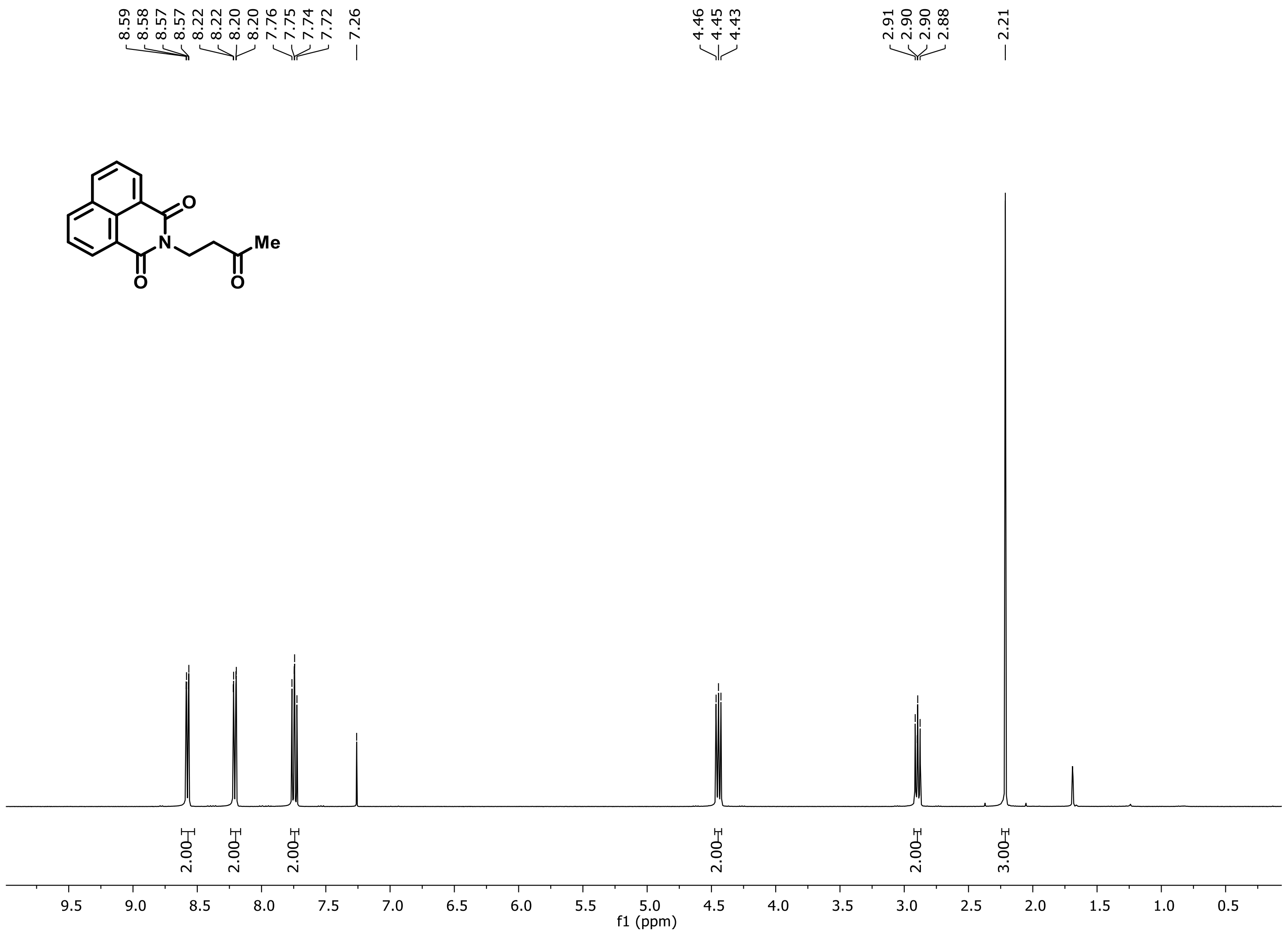

Molecule **3d**: $^{13}C\{^1H\}$ NMR (100 MHz, $CDCl_3$)

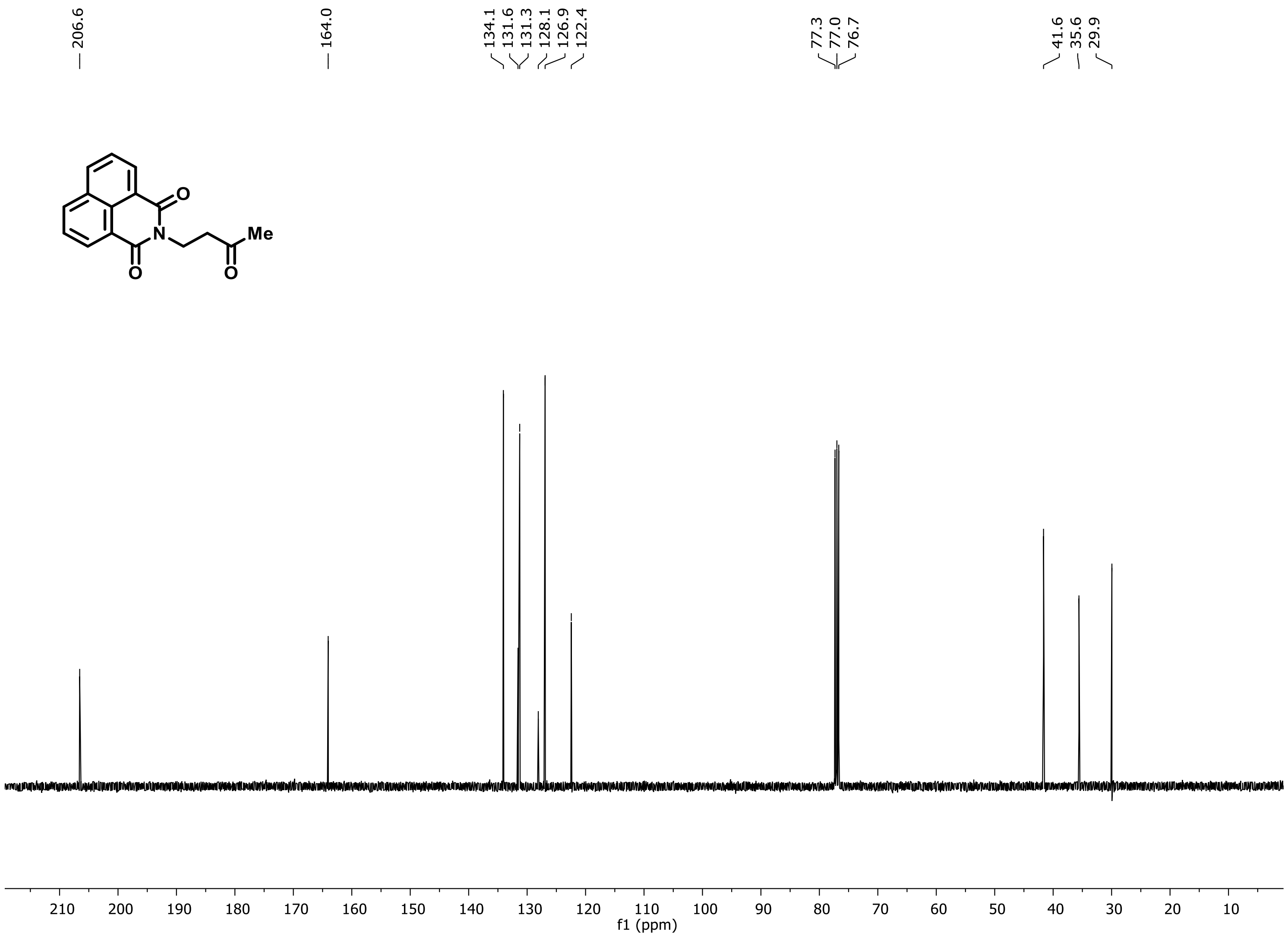

Molecule **4d**: ^{1}H NMR (400 MHz, $CDCl_3$)

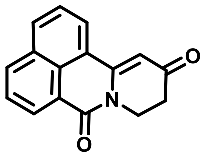

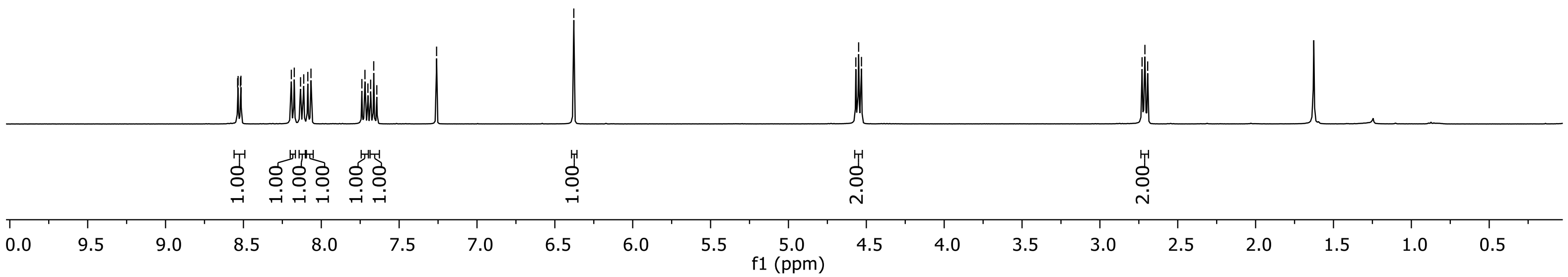

Molecule **4d**: $^{13}C\{^{1}H\}$ NMR (100 MHz, $CDCl_3$)

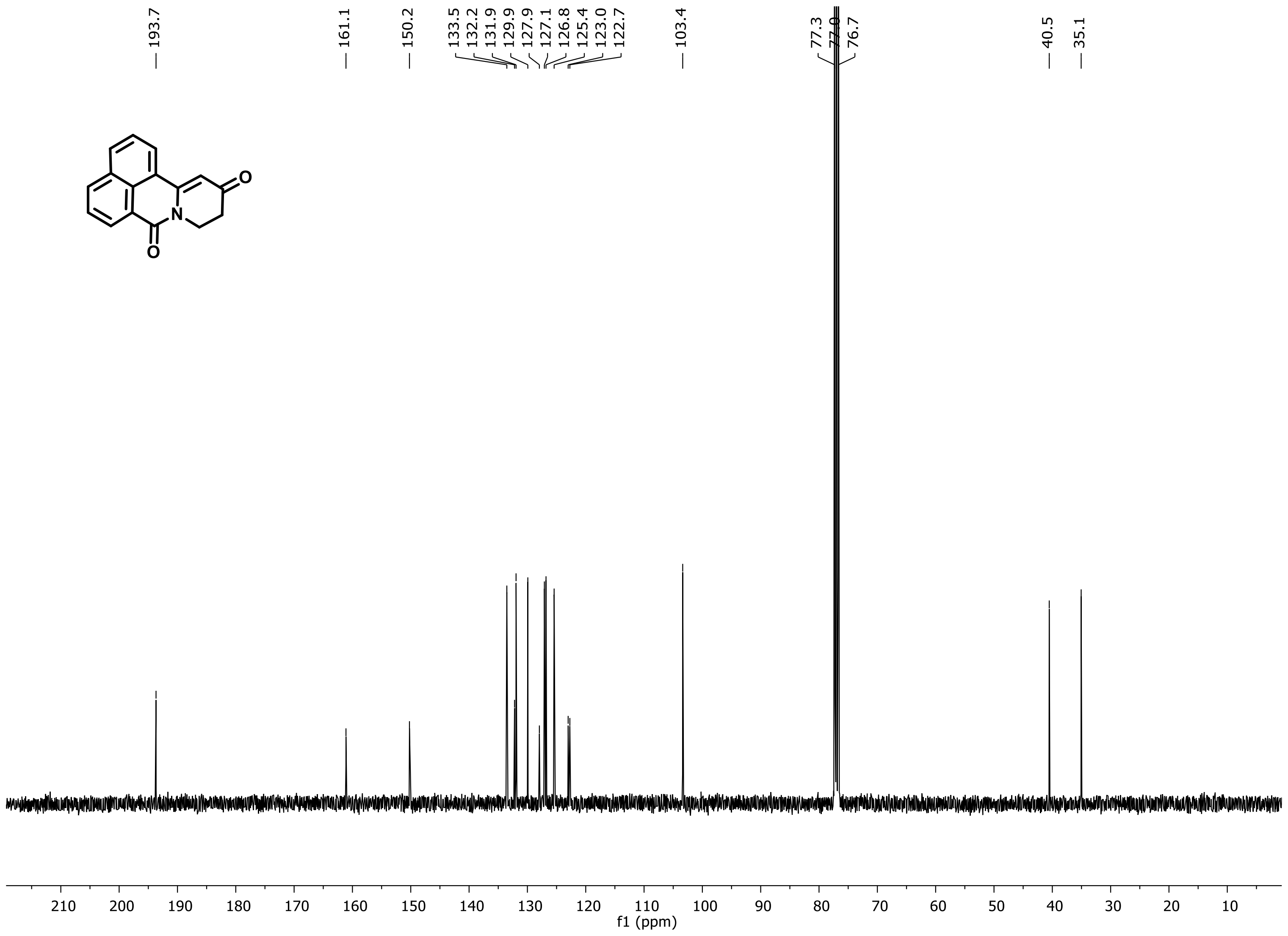

Molecule **3e:** ^{1}H NMR (250 MHz, $CDCl_3$)

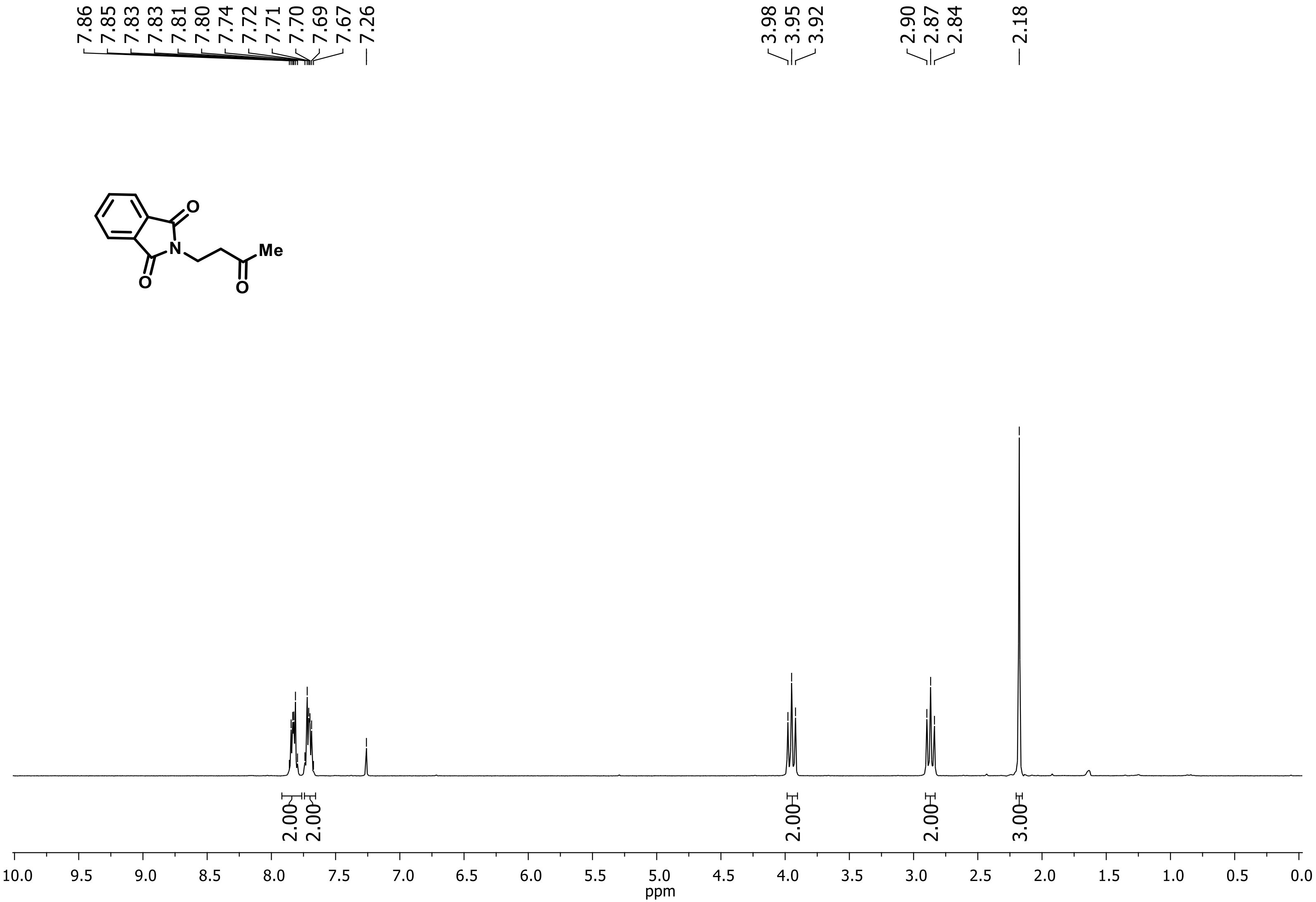

Molecule 3e: $^{13}C\{^1H\}$ NMR (62.5 MHz, $CDCl_3$)

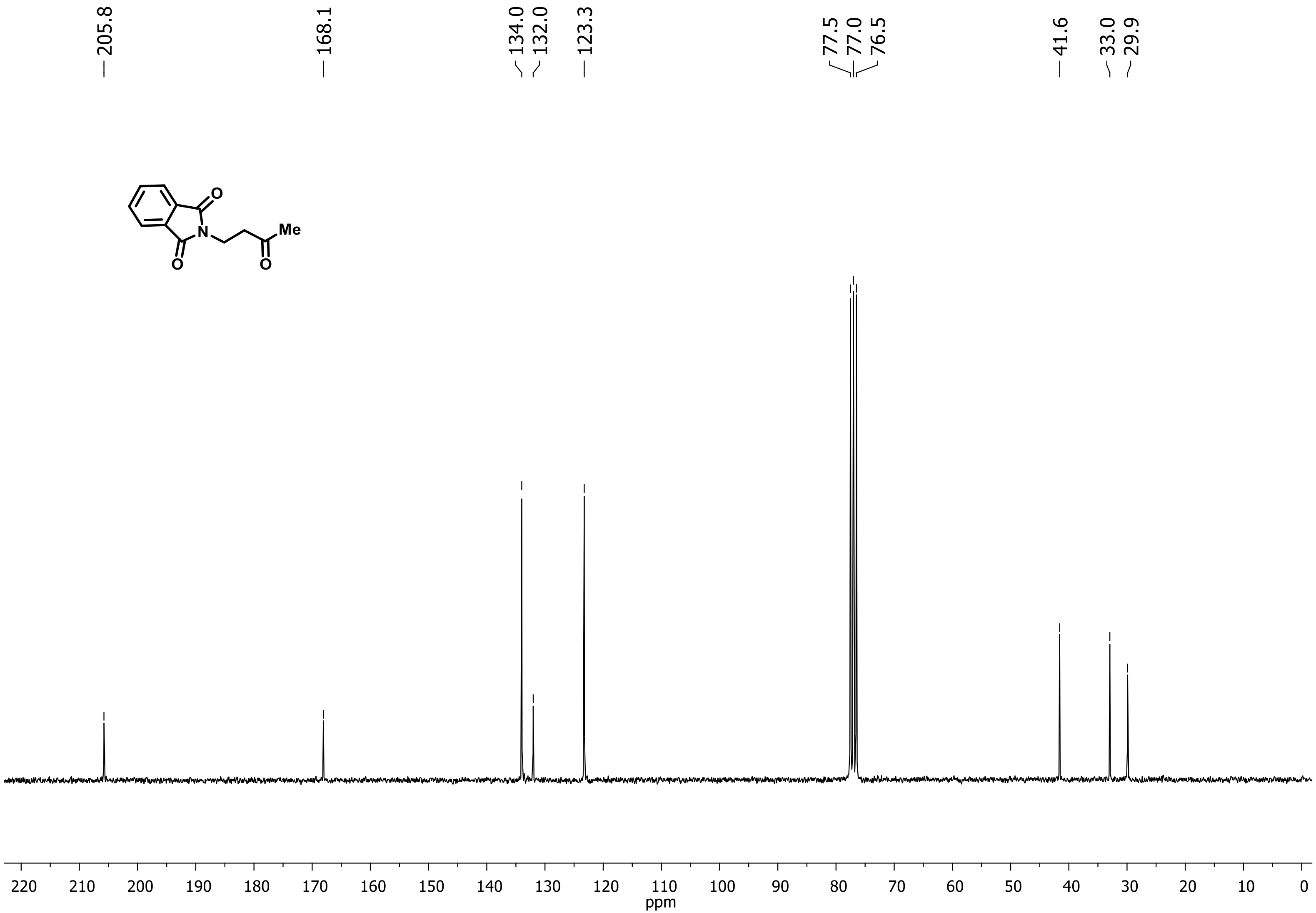

Molecule 4e: ^{1}H NMR (400 MHz, $CDCl_3$)

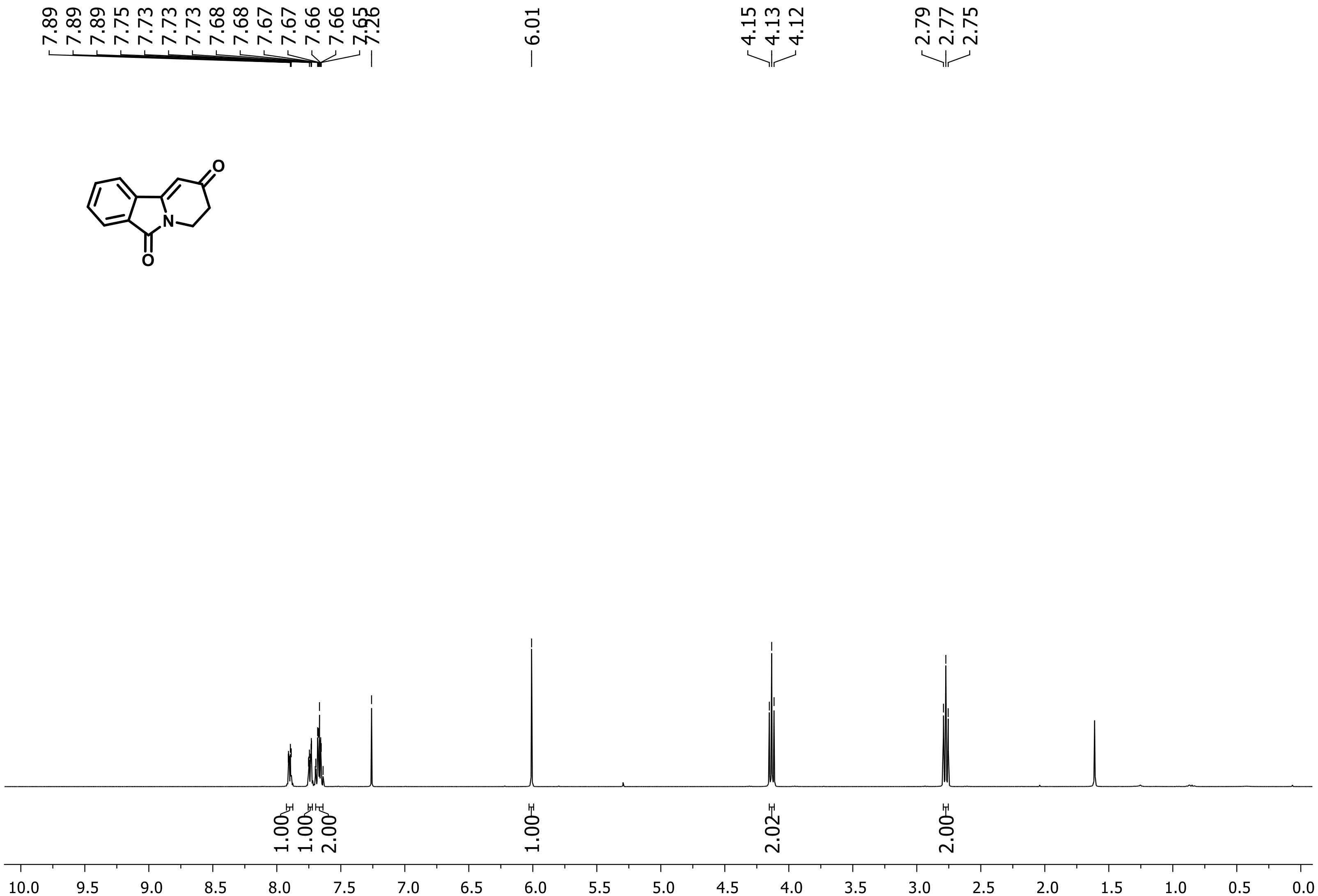

Molecule 4e: $^{13}C\{^1H\}$ NMR (100 MHz, $CDCl_3$)

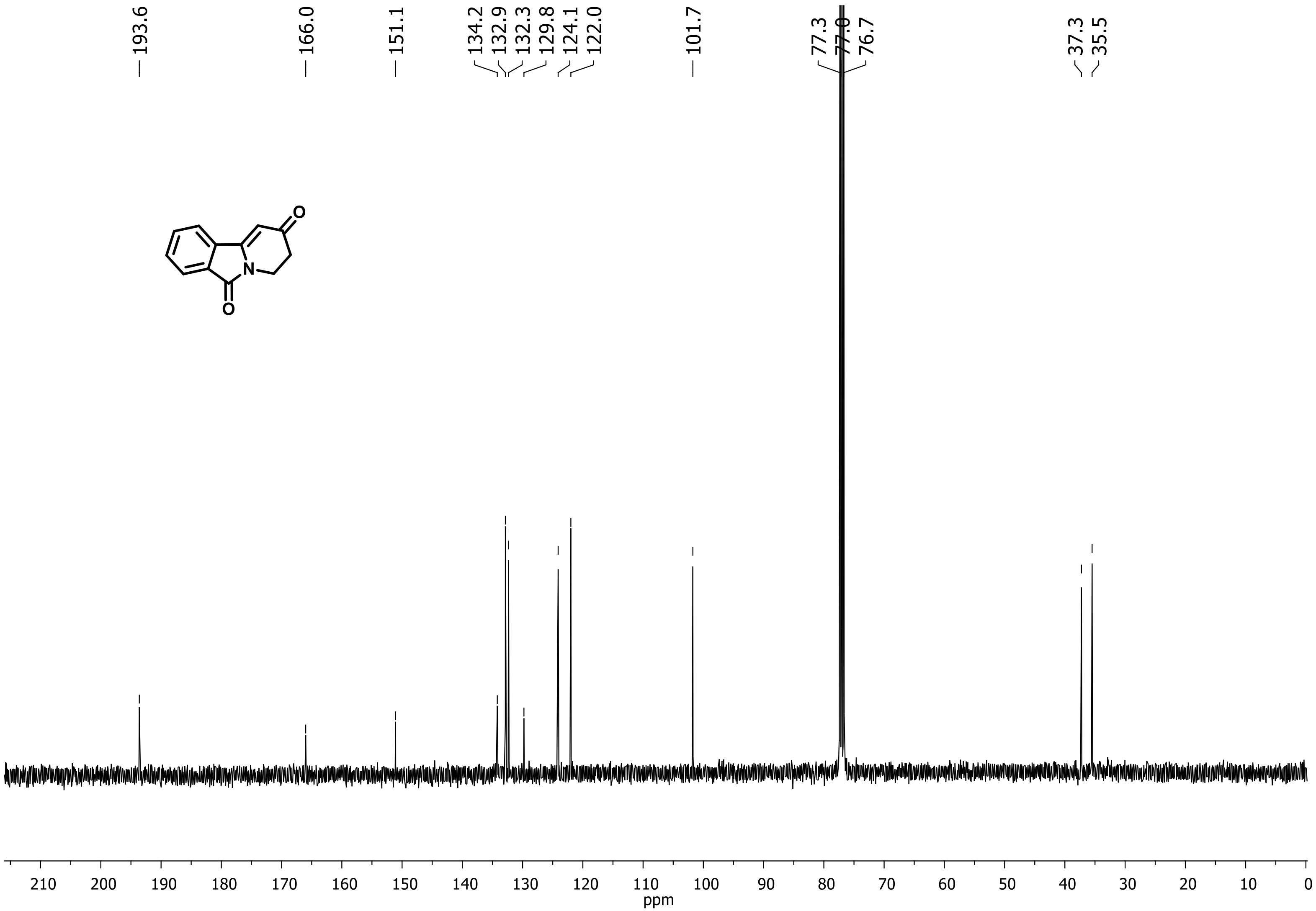

Molecule **3f**: ^{1}H NMR (400 MHz, $CDCl_3$)

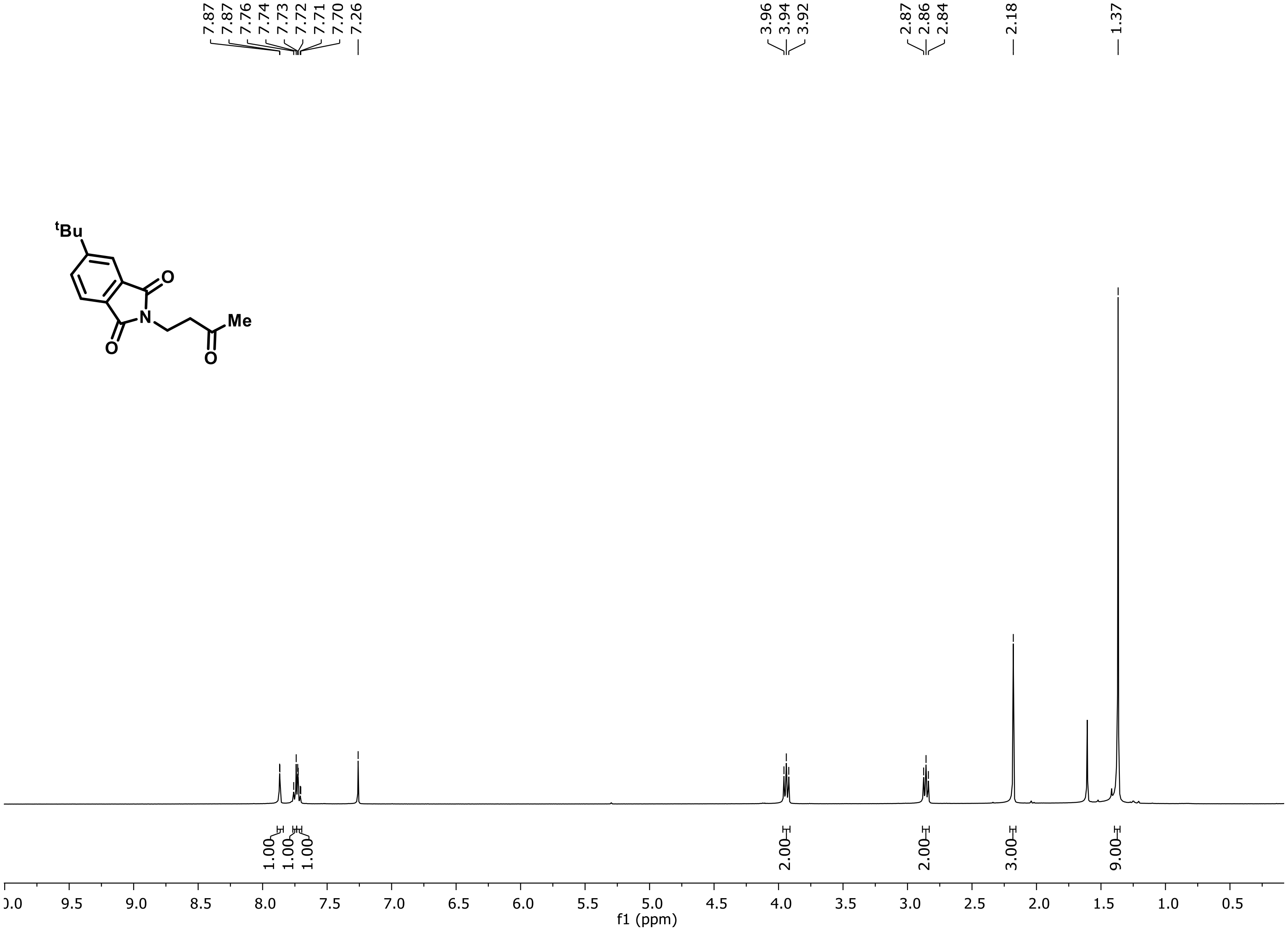

Molecule **3f**: $^{13}C\{^1H\}$ NMR (100 MHz, $CDCl_3$)

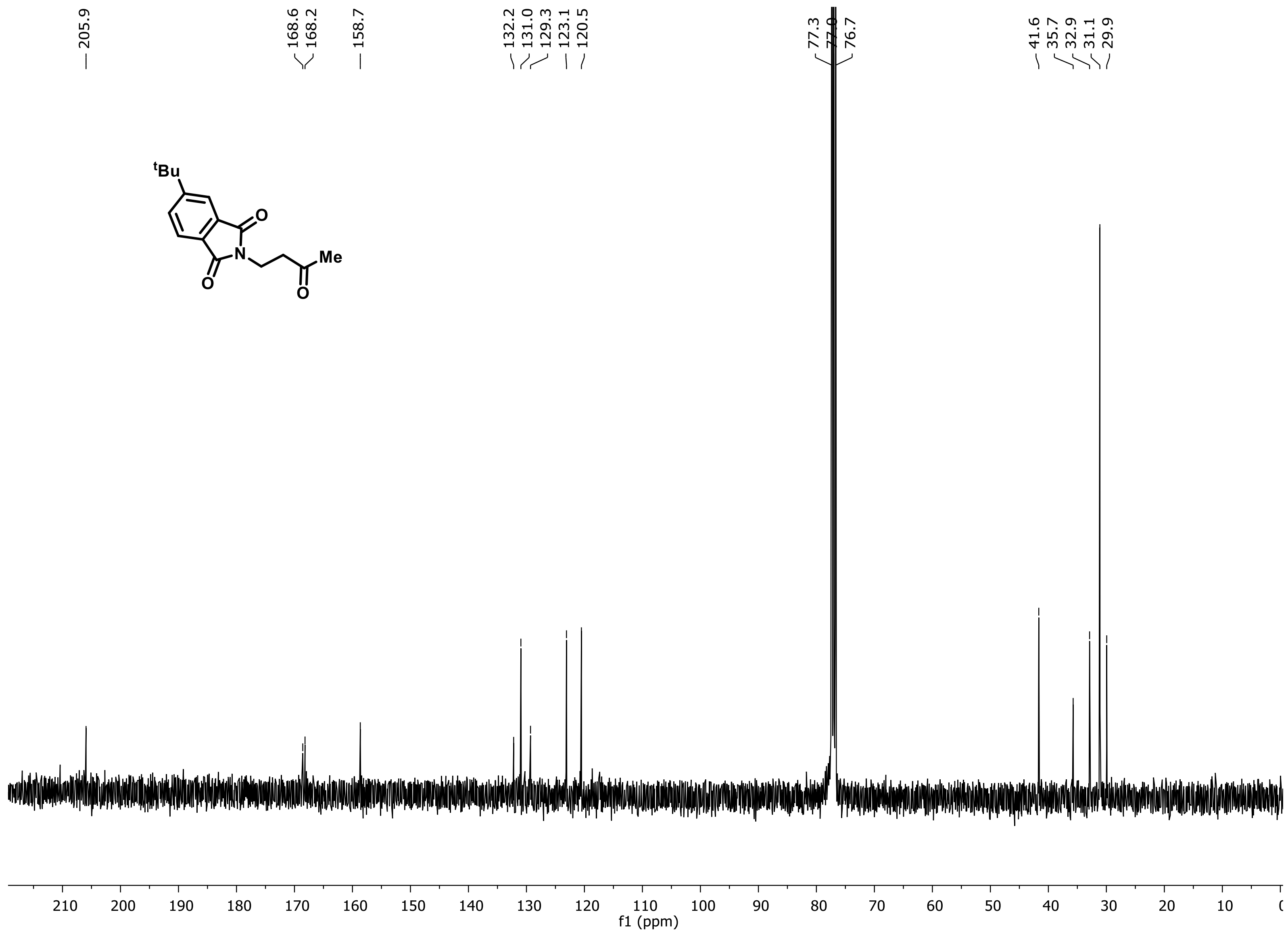

Molecules **4f** + **4f’** (1:1 r.r.): ^{1}H NMR (400 MHz, $CDCl_3$)

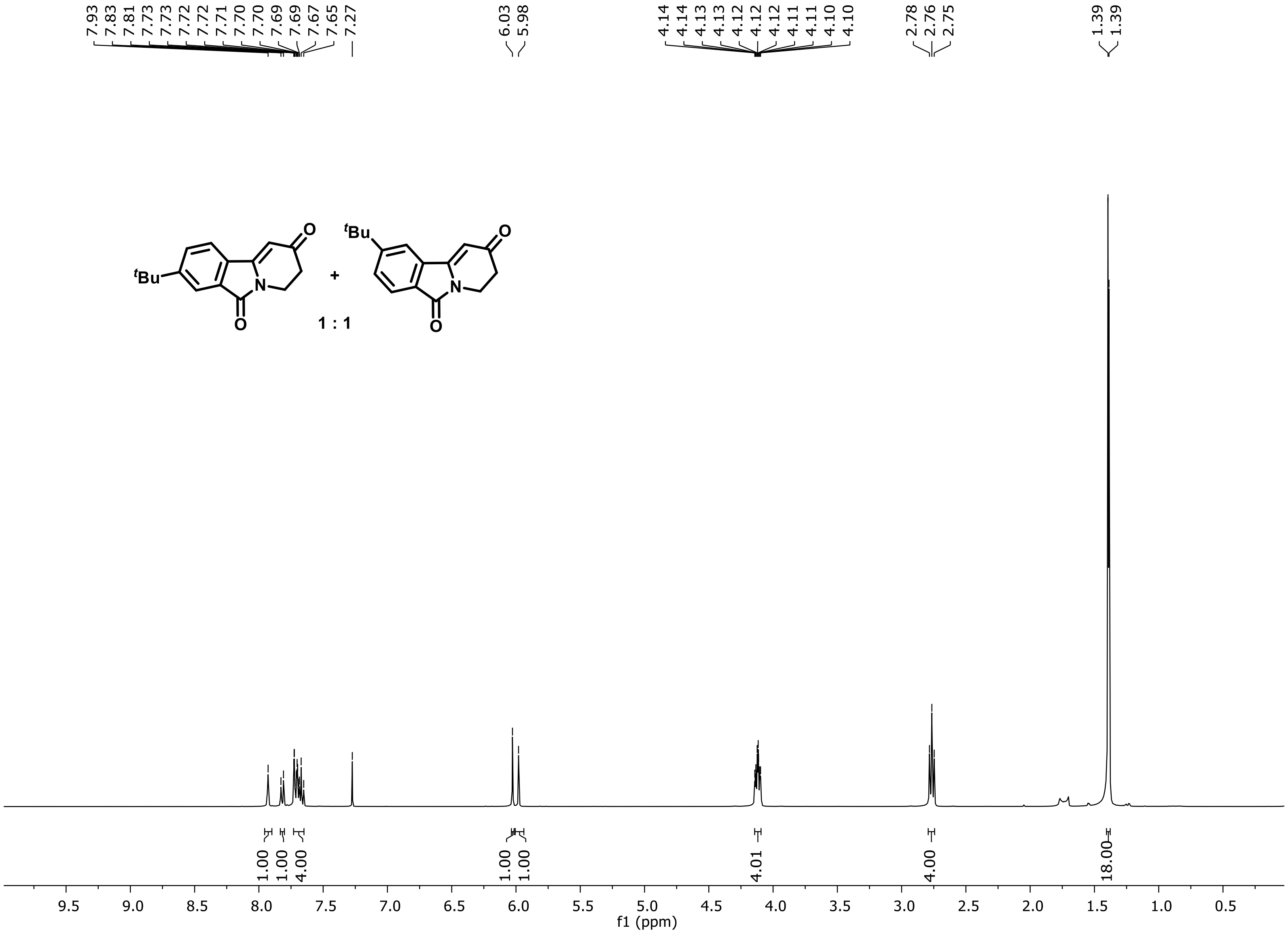

Molecules **4f** + **4f'** (1:1 r.r.): $^{13}C\{^1H\}$ NMR (100 MHz, $CDCl_3$)

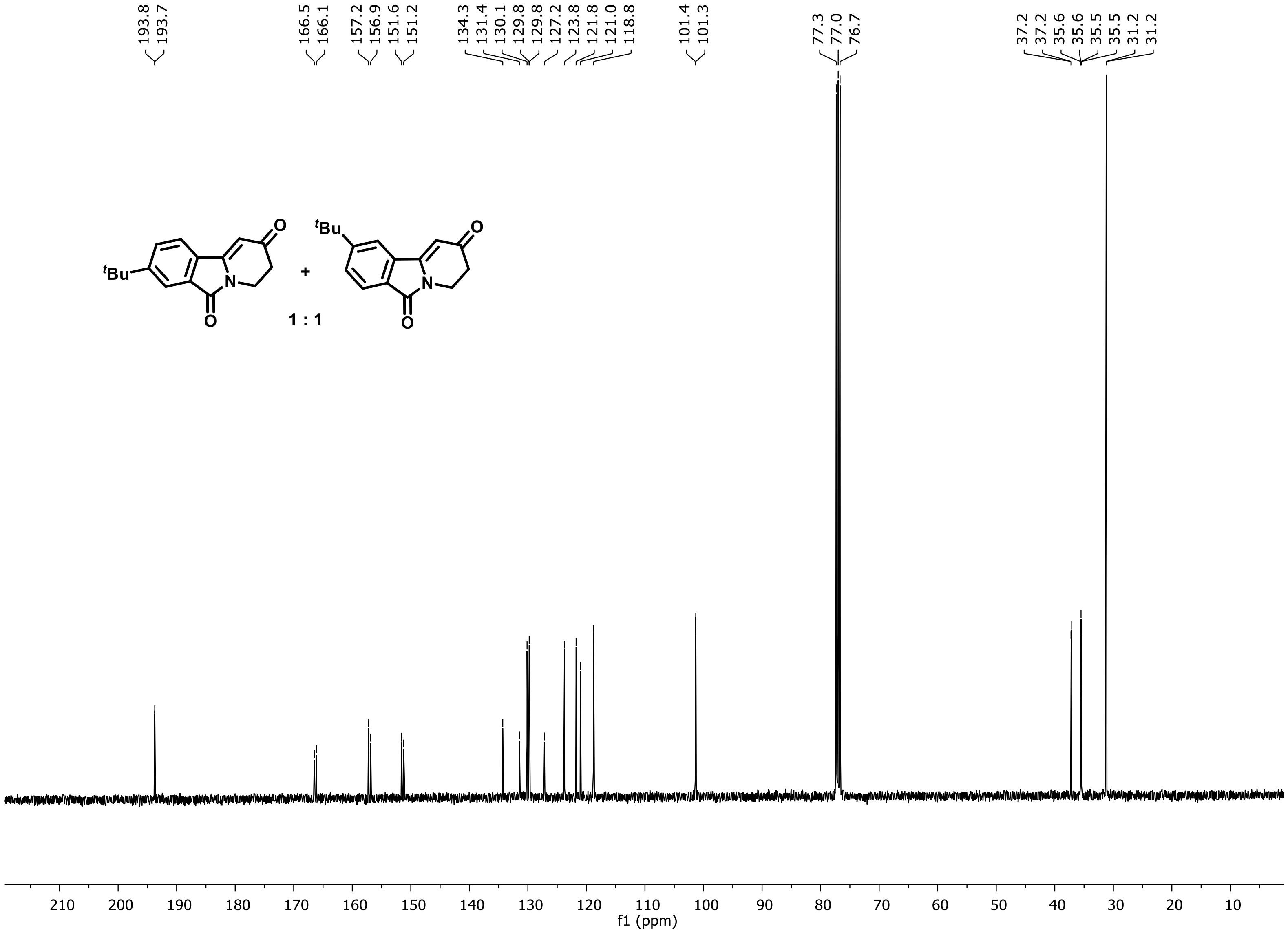

Molecule **3g**: ^{1}H NMR (400 MHz, $CDCl_3$)

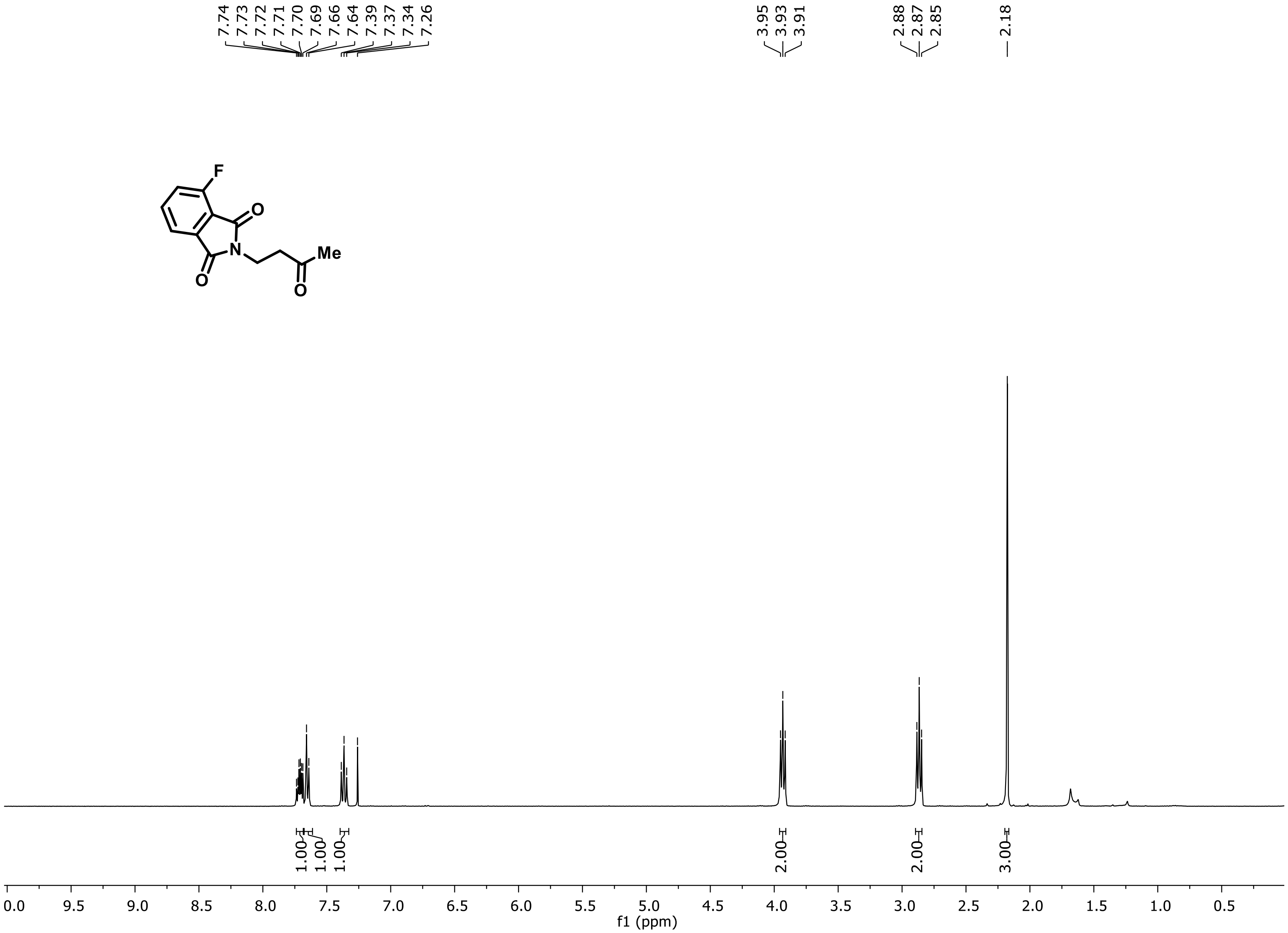

Molecule **3g**: $^{13}C\{^{1}H\}$ NMR (100 MHz, $CDCl_3$)

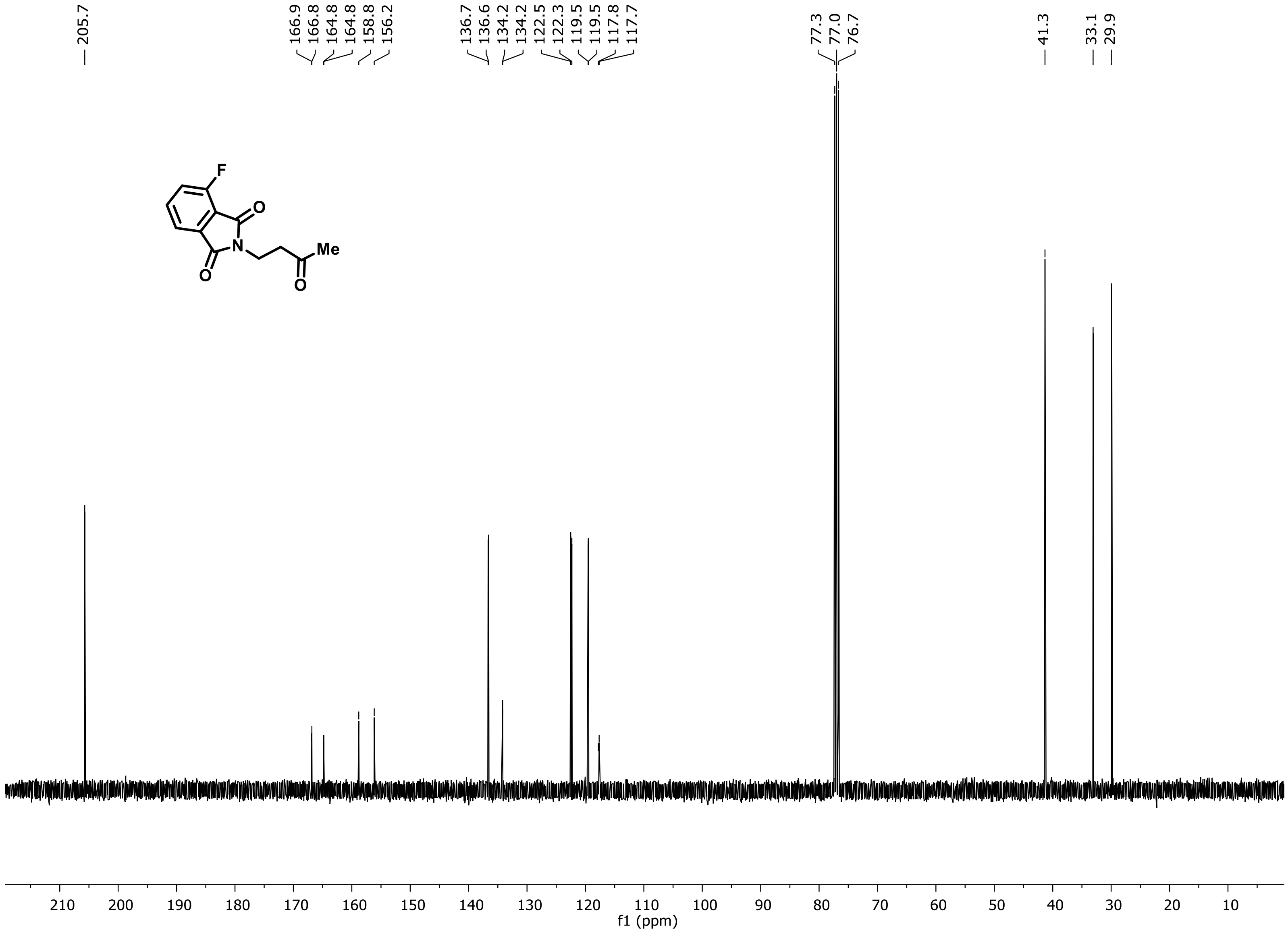

Molecule **3g**: ^{19}F NMR (376.5 MHz, $CDCl_3$) [TFA used as internal reference, δ = -75.39 ppm][17]

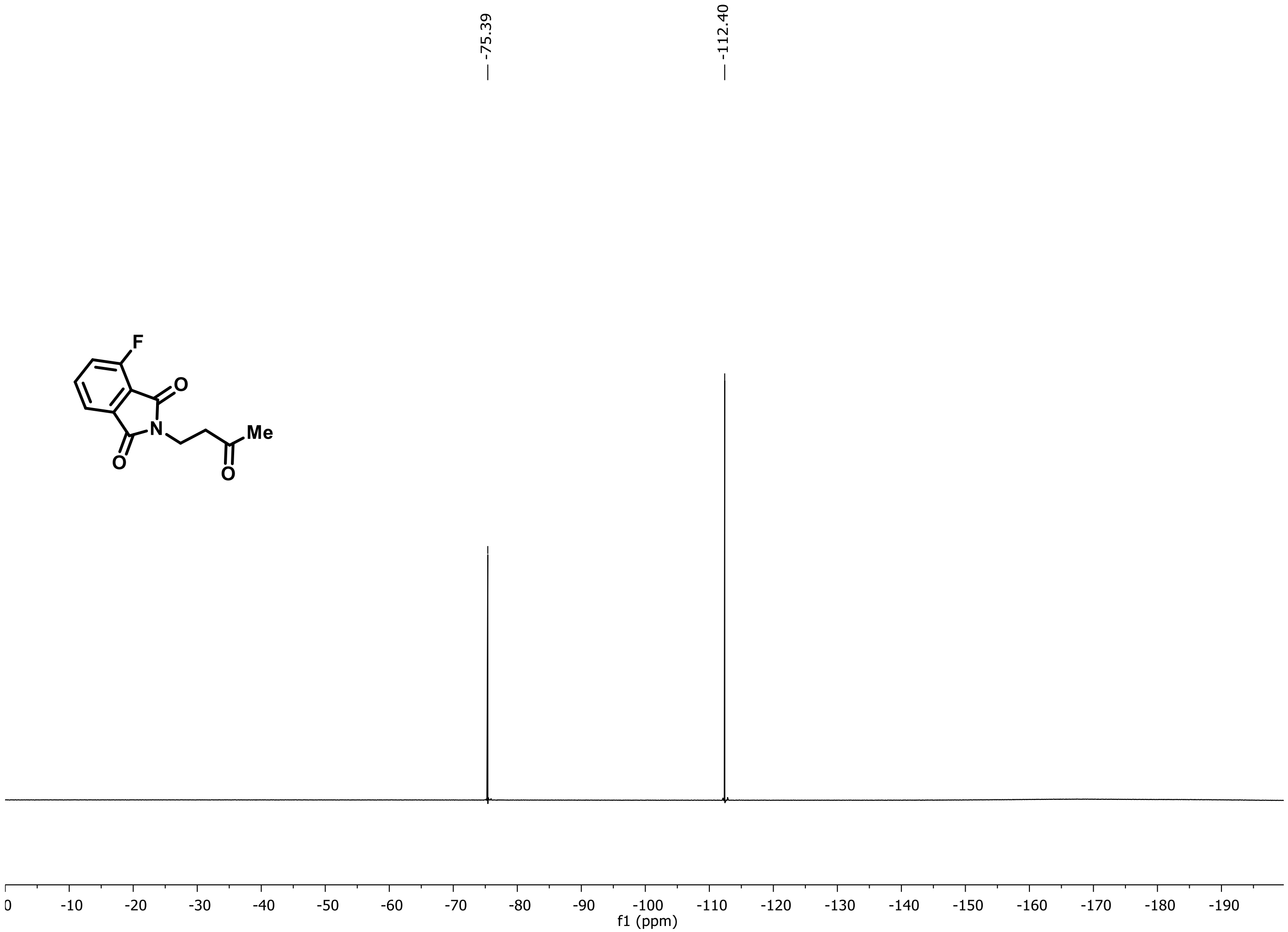

[17] Rosenau, C. P.; Jelier, B. J.; Gossert, A. D.; Togni, A.; *Angew. Chem. Int. Ed.*, **2018**, *57*, 9528-9533.

Molecules **4g**: ^{1}H NMR (400 MHz, $CDCl_3$)

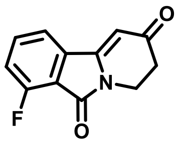

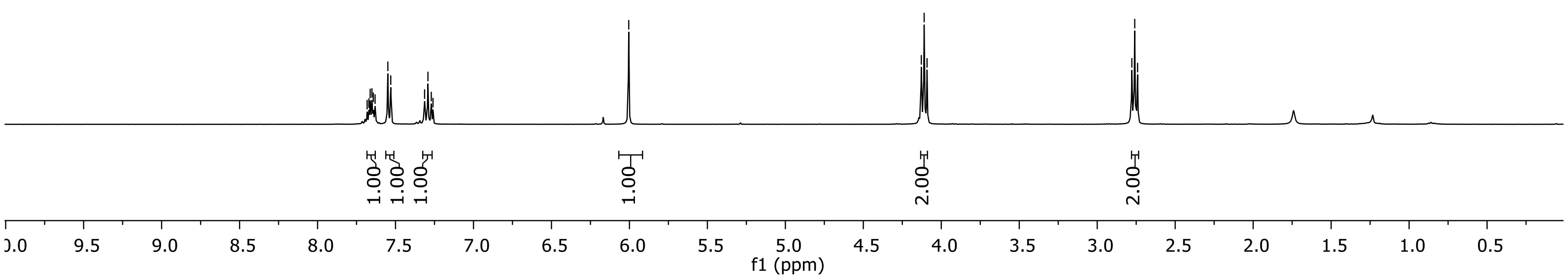

Molecules **4g**: $^{13}C\{^{1}H\}$ NMR (100 MHz, $CDCl_3$)

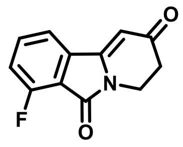

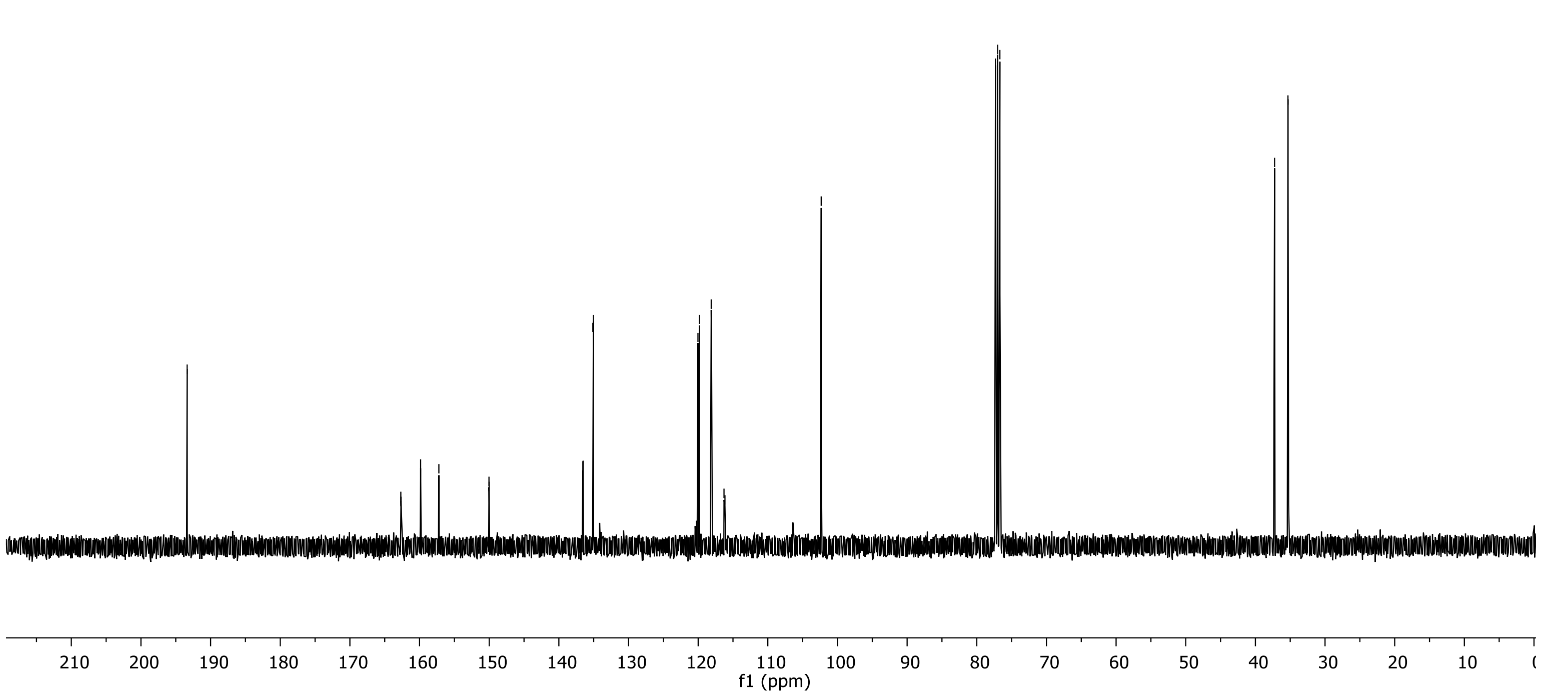

Molecules **4g**: ^{19}F NMR (376.5 MHz, $CDCl_3$) [TFA used as internal reference, δ = -75.39 ppm][17]

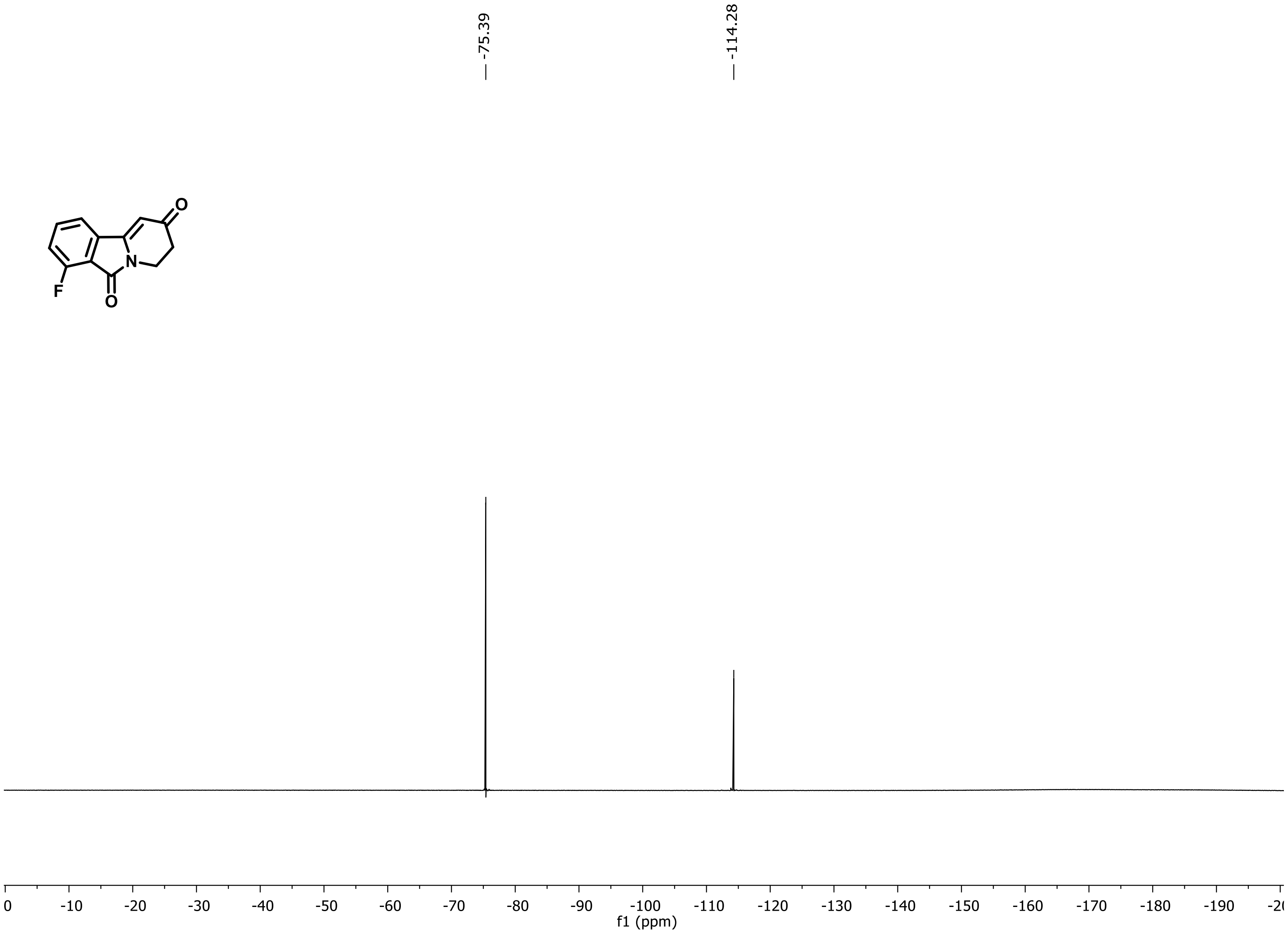

Molecules **4g**: NOESY (400 MHz, $CDCl_3$)

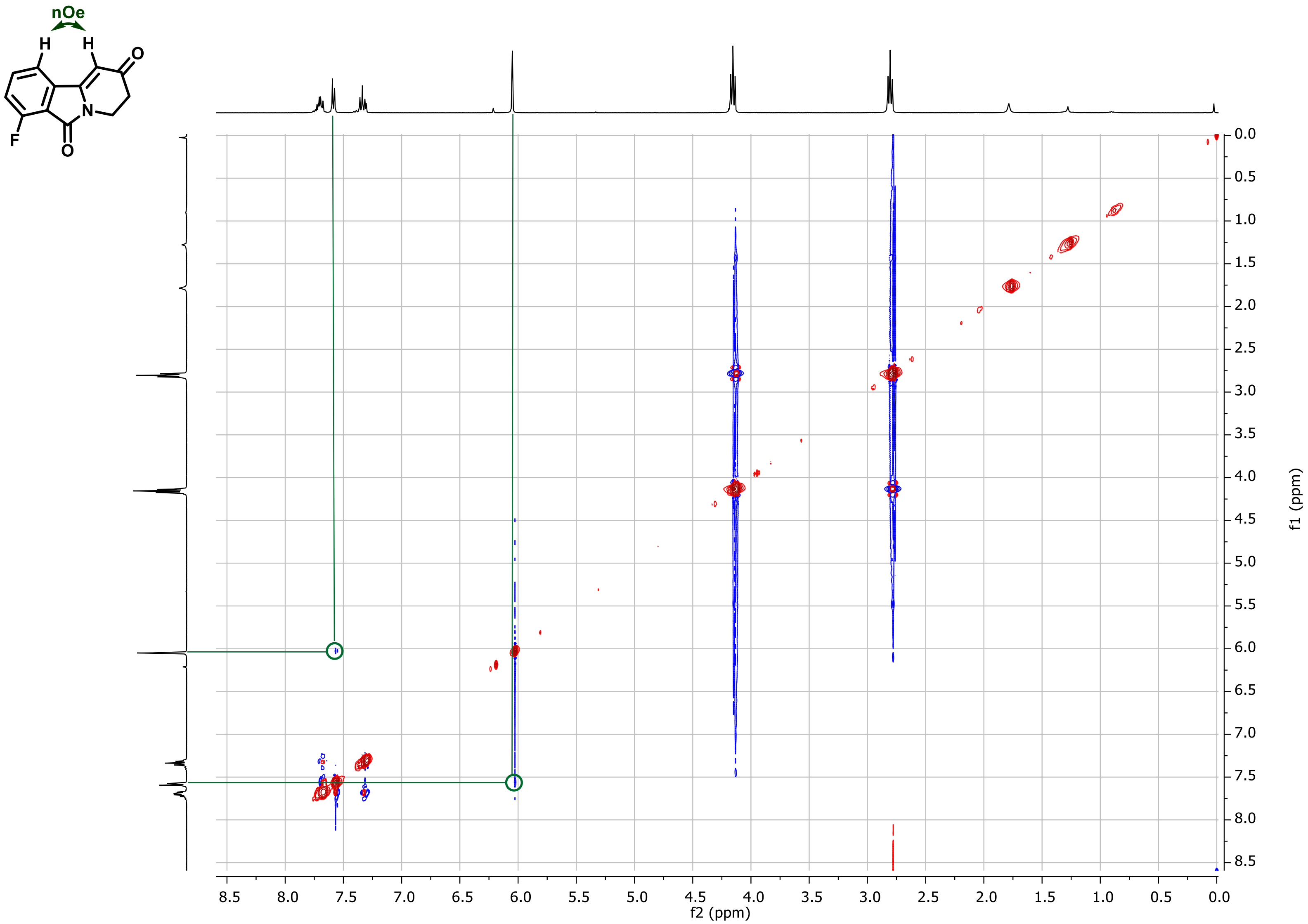

Molecule **3h**: ^{1}H NMR (400 MHz, $CDCl_3$)

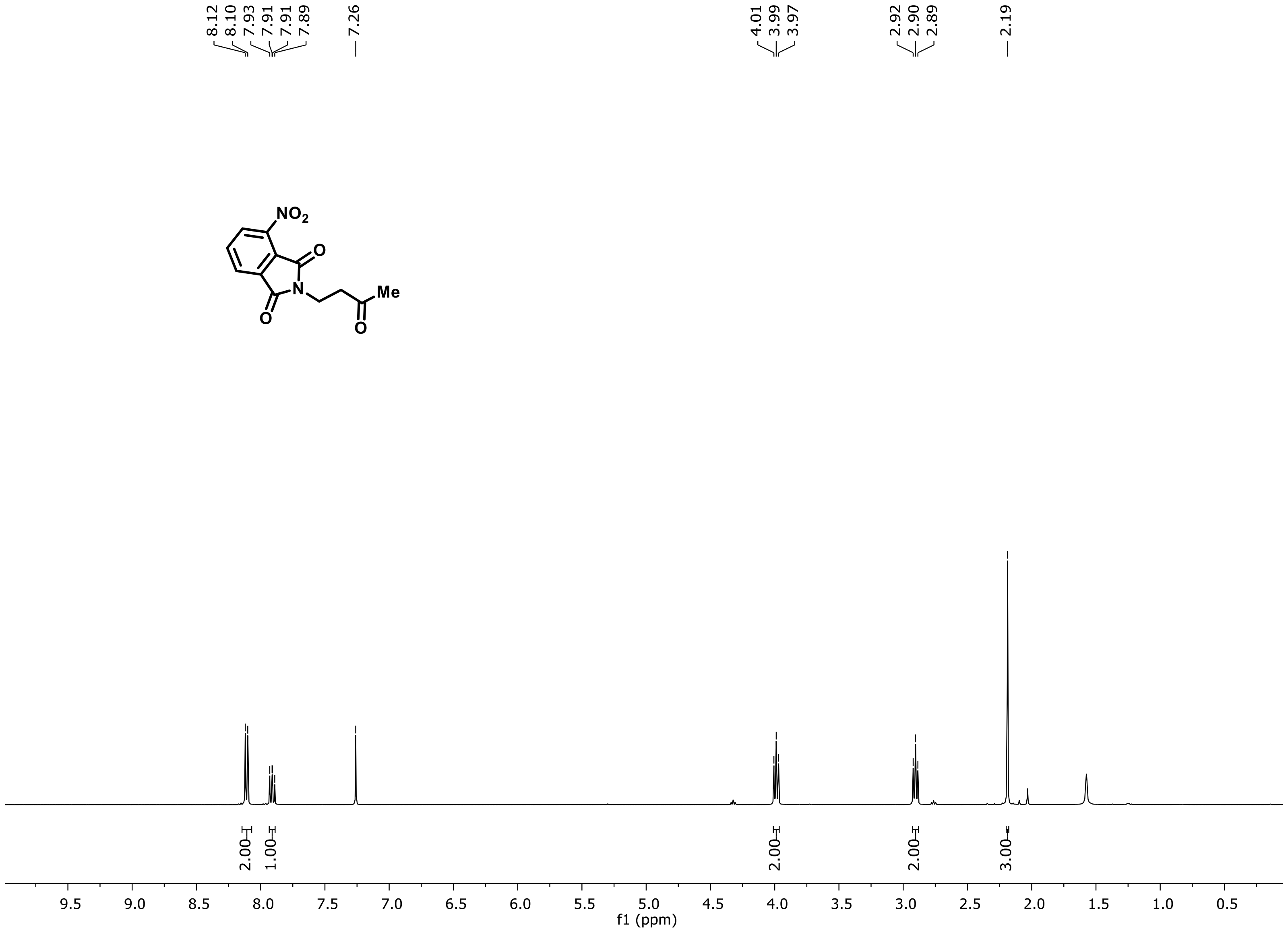

Molecule **3h**: $^{13}C\{^1H\}$ NMR (100 MHz, $CDCl_3$)

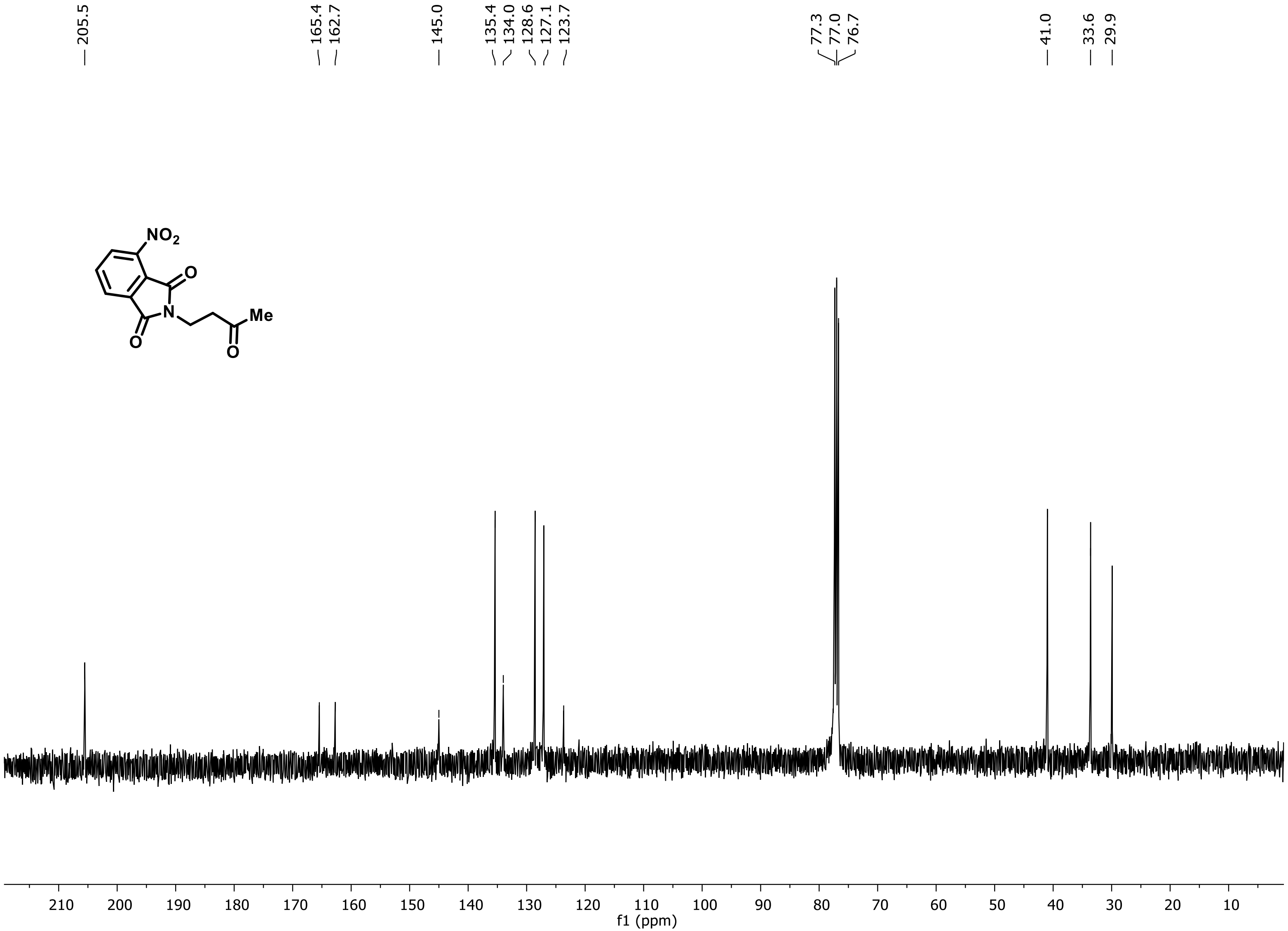

Molecules **4h** + **4h'** (2.6:1 r.r.): ^{1}H NMR (400 MHz, $CDCl_3$)

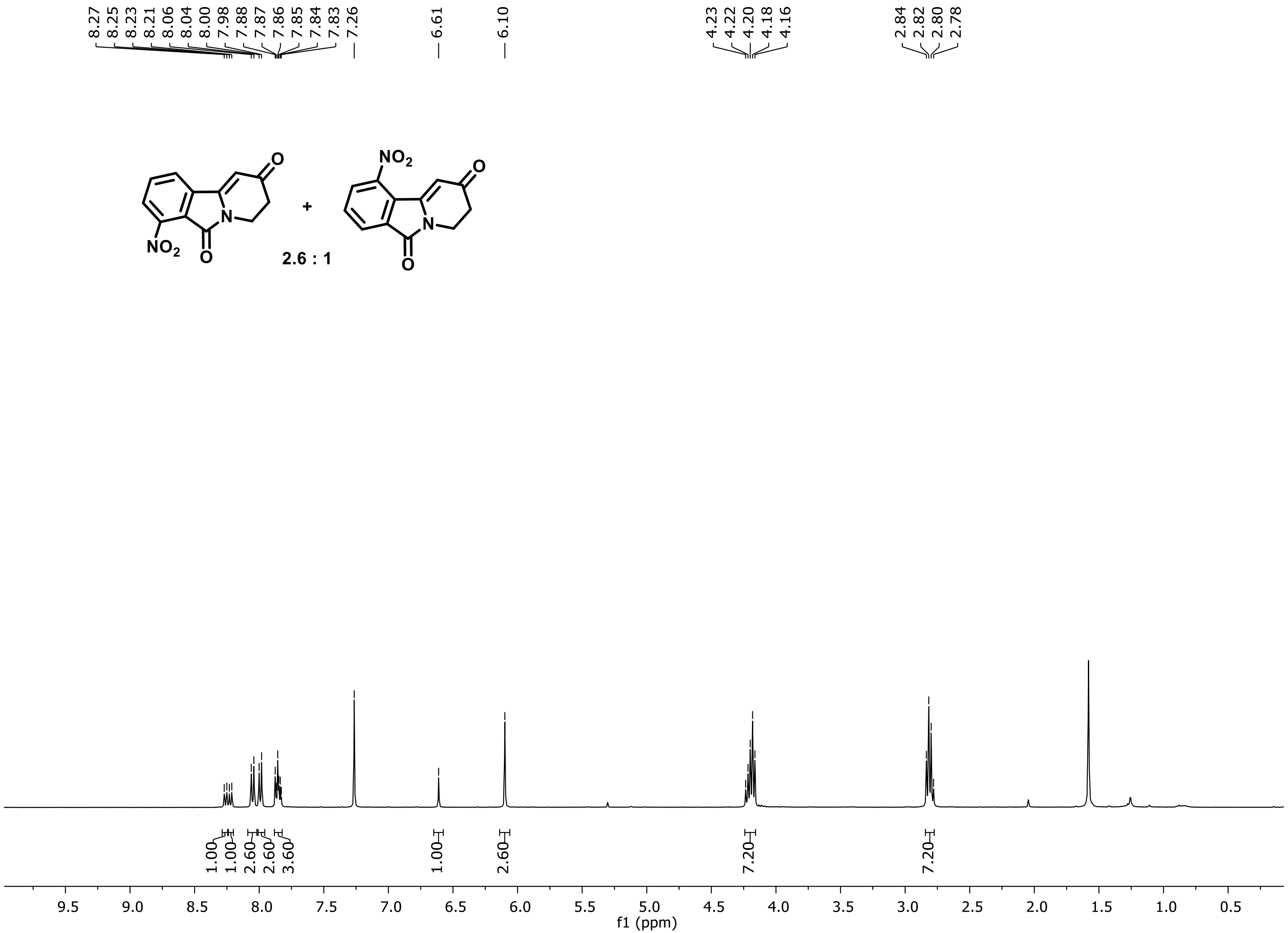

Molecules **4h** + **4h'** (2.6:1 r.r.): $^{13}C\{^{1}H\}$ NMR (100 MHz, $CDCl_3$)

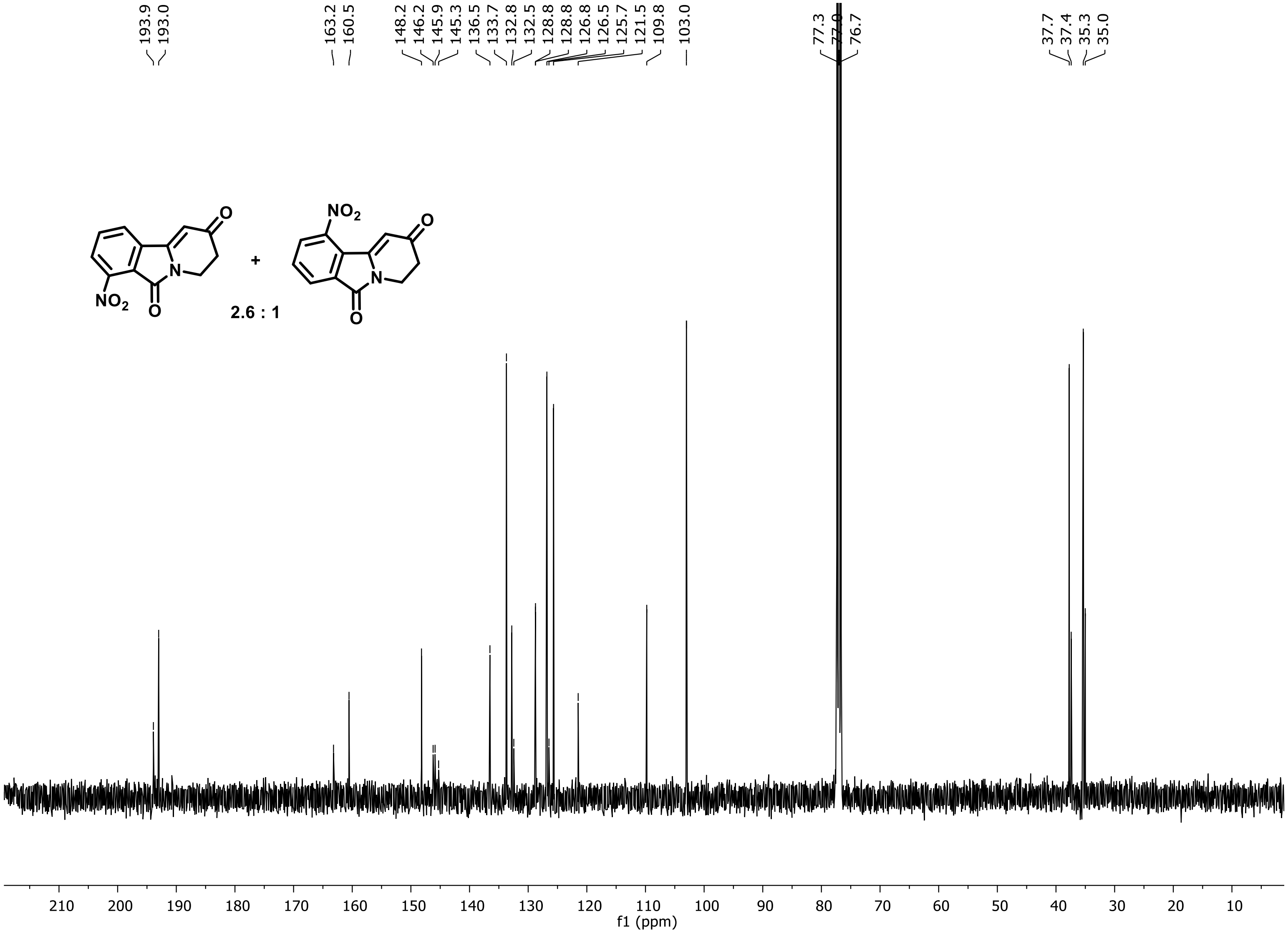

Molecules **4h** + **4h’** (2.6:1 r.r.): NOESY (400 MHz, $CDCl_3$)

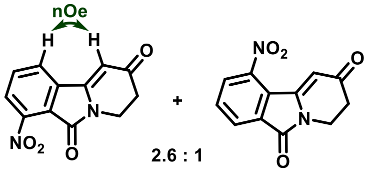

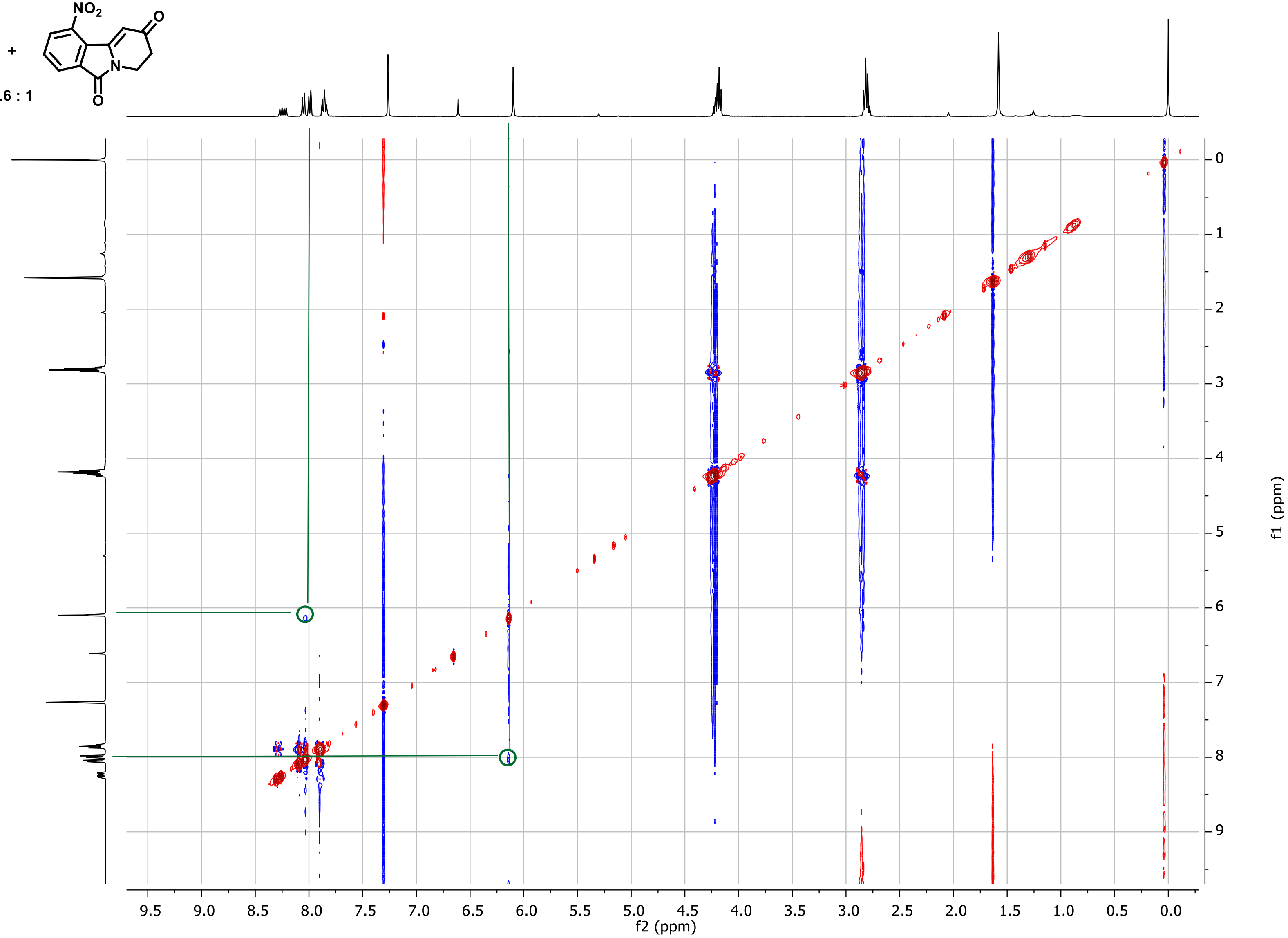

Molecule **3i**: ^{1}H NMR (400 MHz, $CDCl_3$)

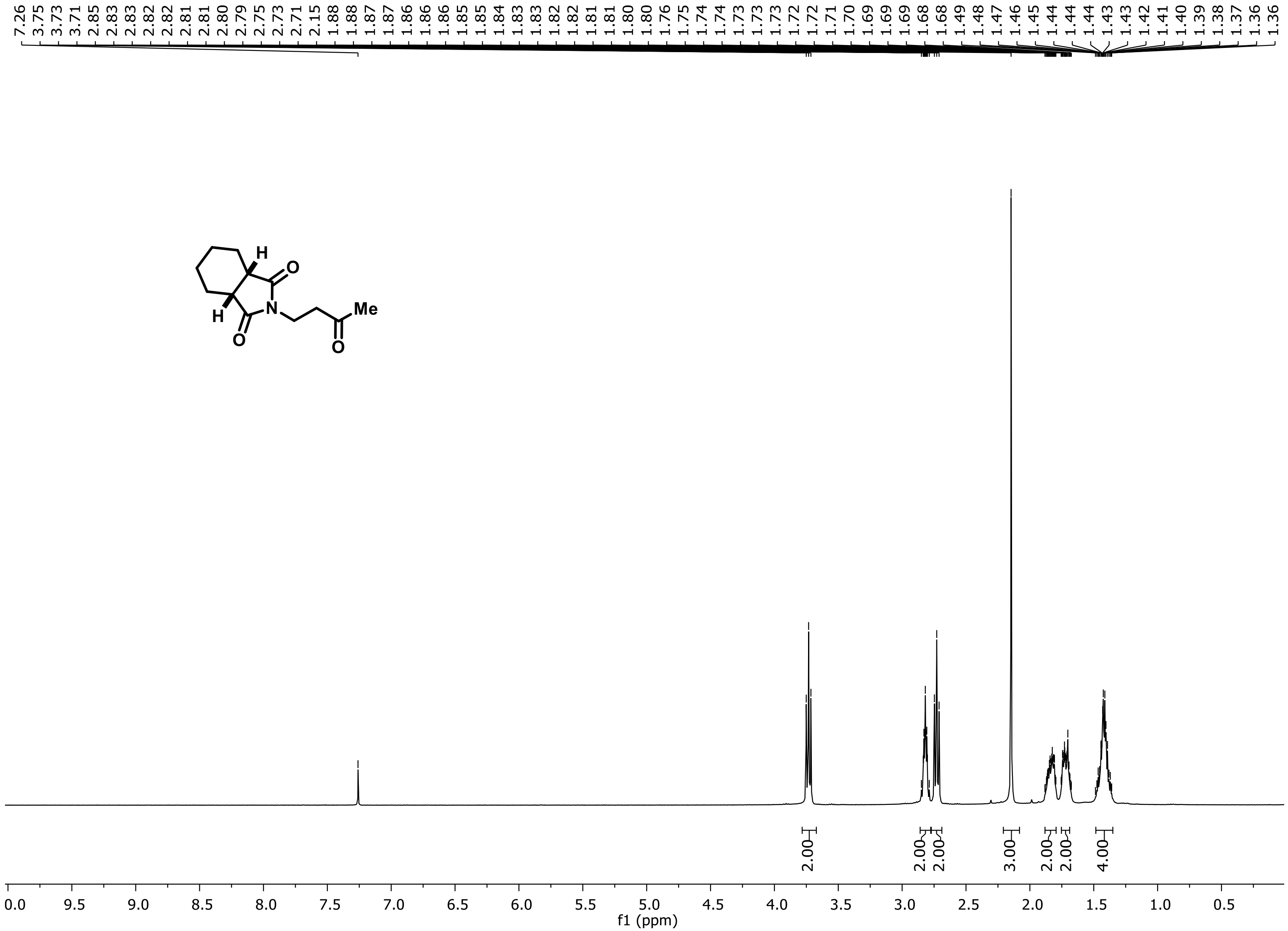

Molecule **3i**: $^{13}C\{^{1}H\}$ NMR (100 MHz, $CDCl_3$)

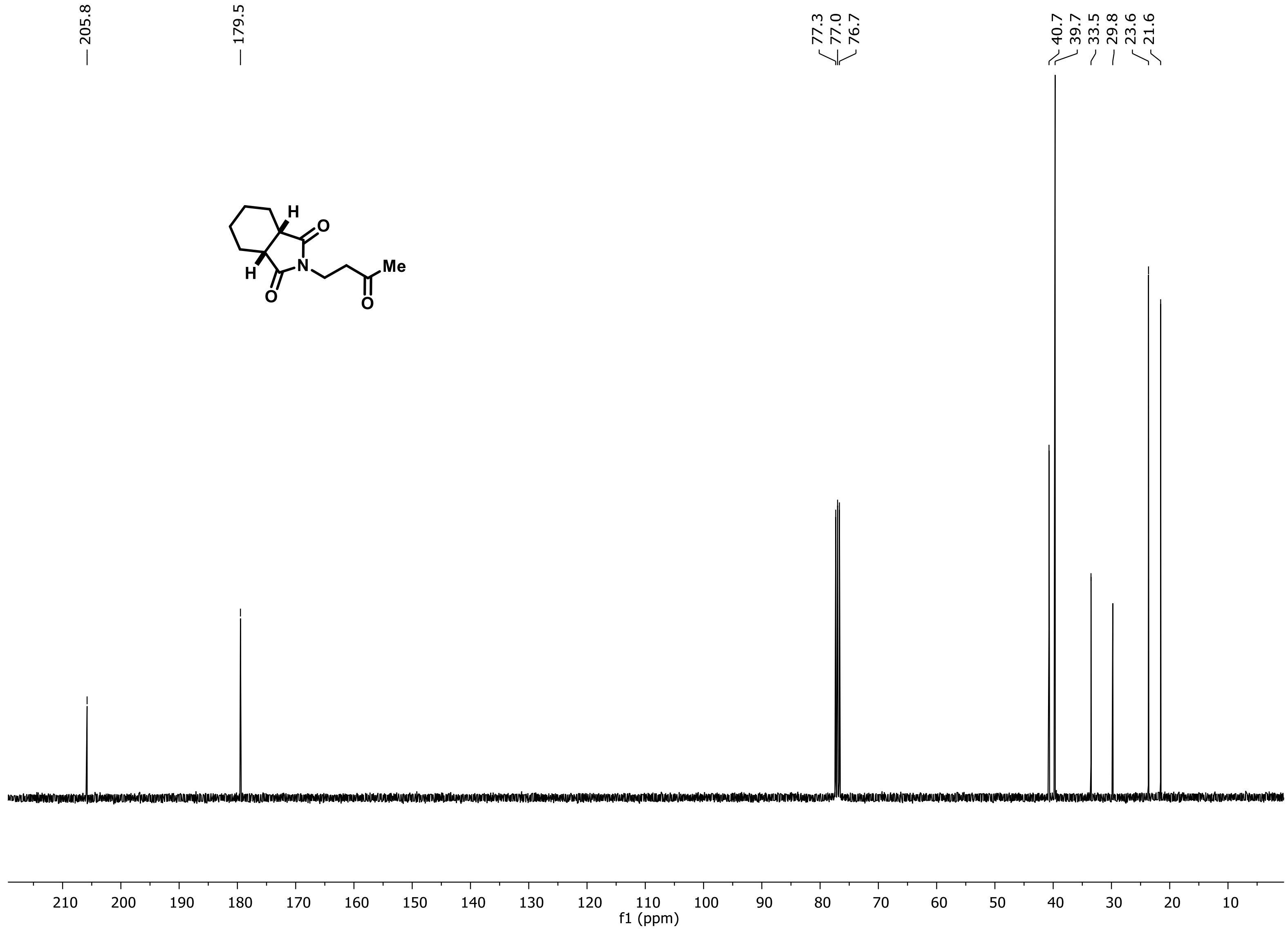

Molecule **4i**: ^{1}H NMR (400 MHz, $CDCl_3$)

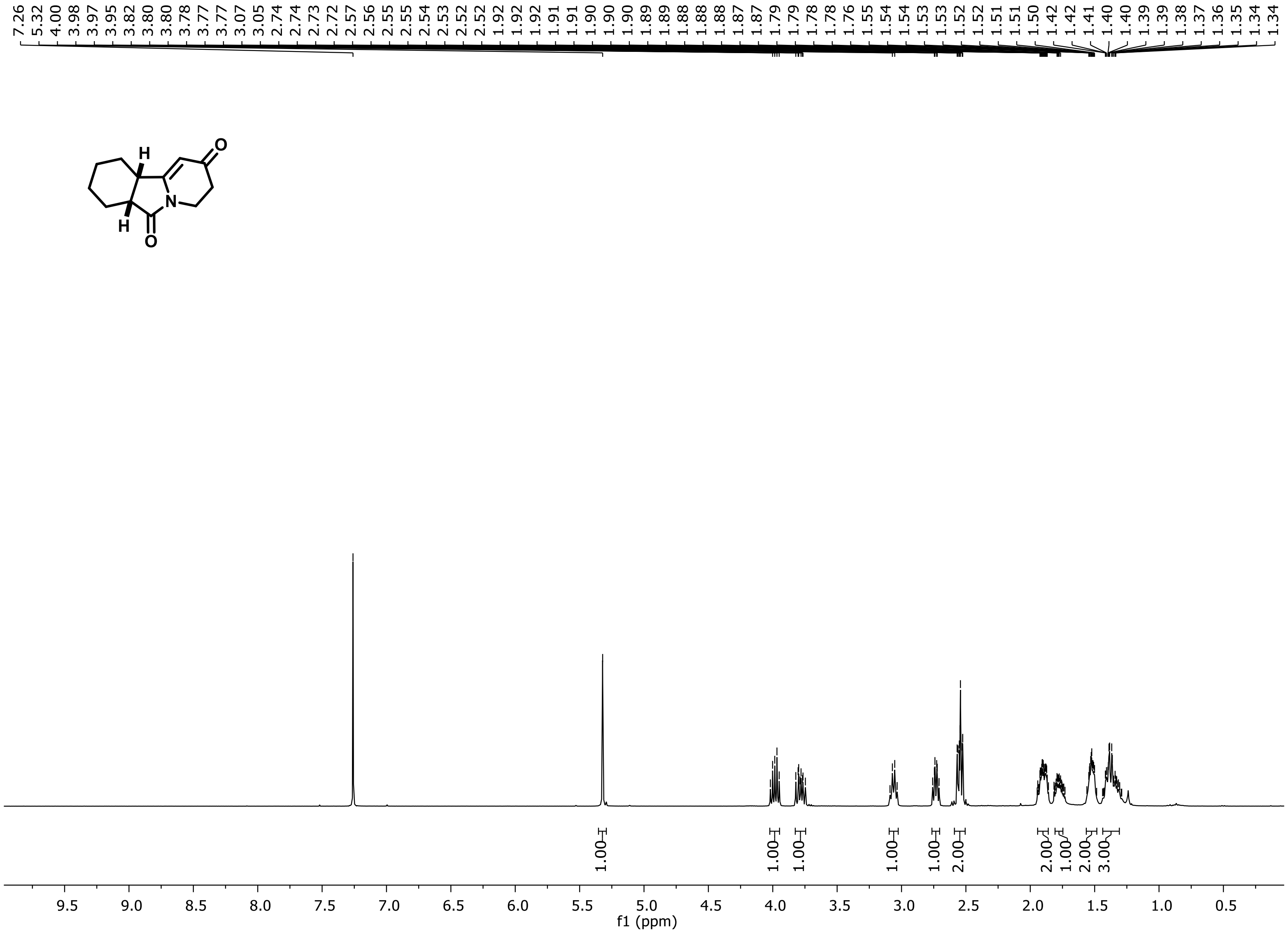

Molecule **4i**: $^{13}C\{^{1}H\}$ NMR (100 MHz, $CDCl_3$)

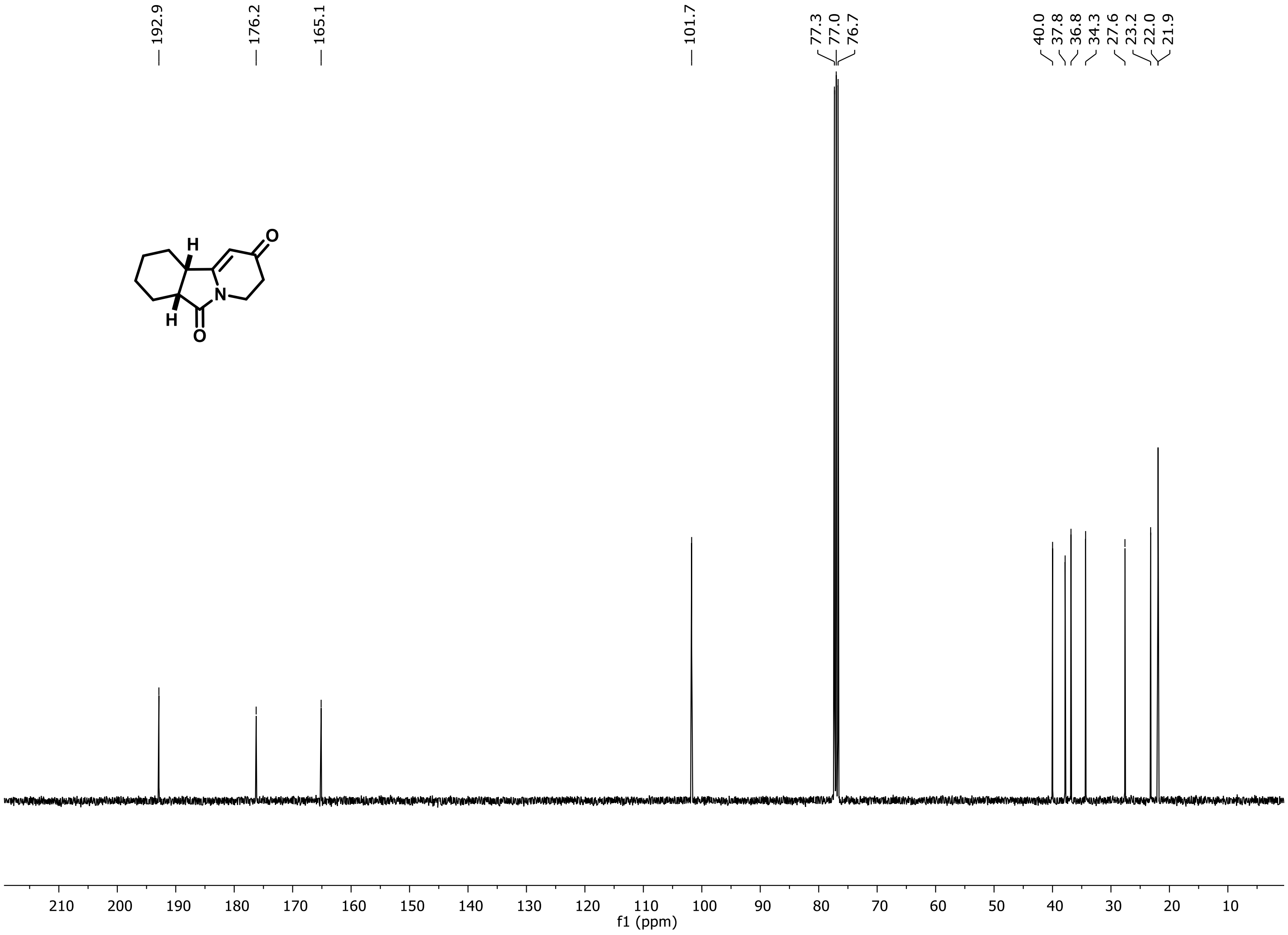

Molecule **3j**: ^{1}H NMR (250 MHz, $CDCl_3$)

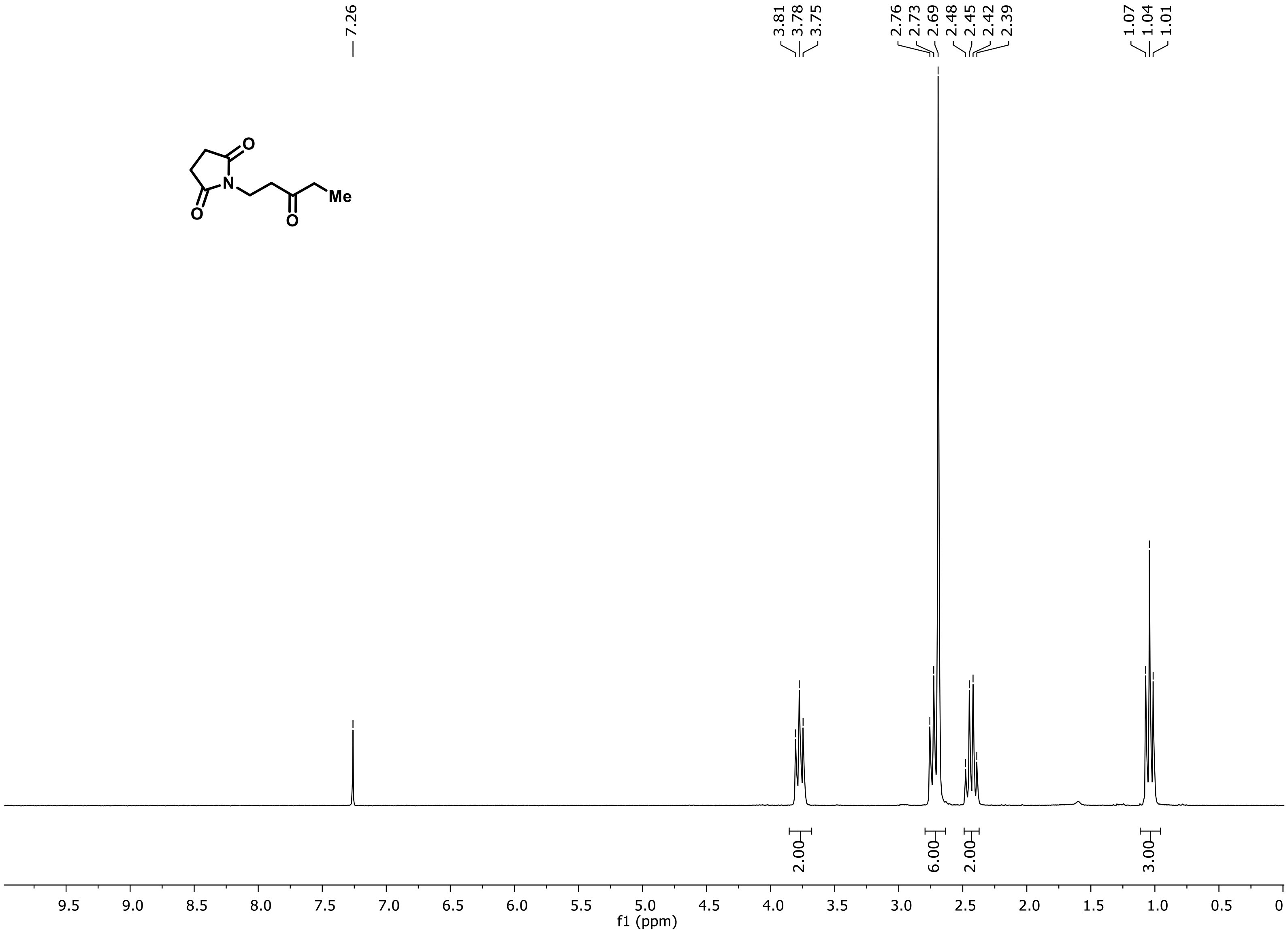

Molecule **3j**: $^{13}C\{^{1}H\}$ NMR (62.5 MHz, $CDCl_3$)

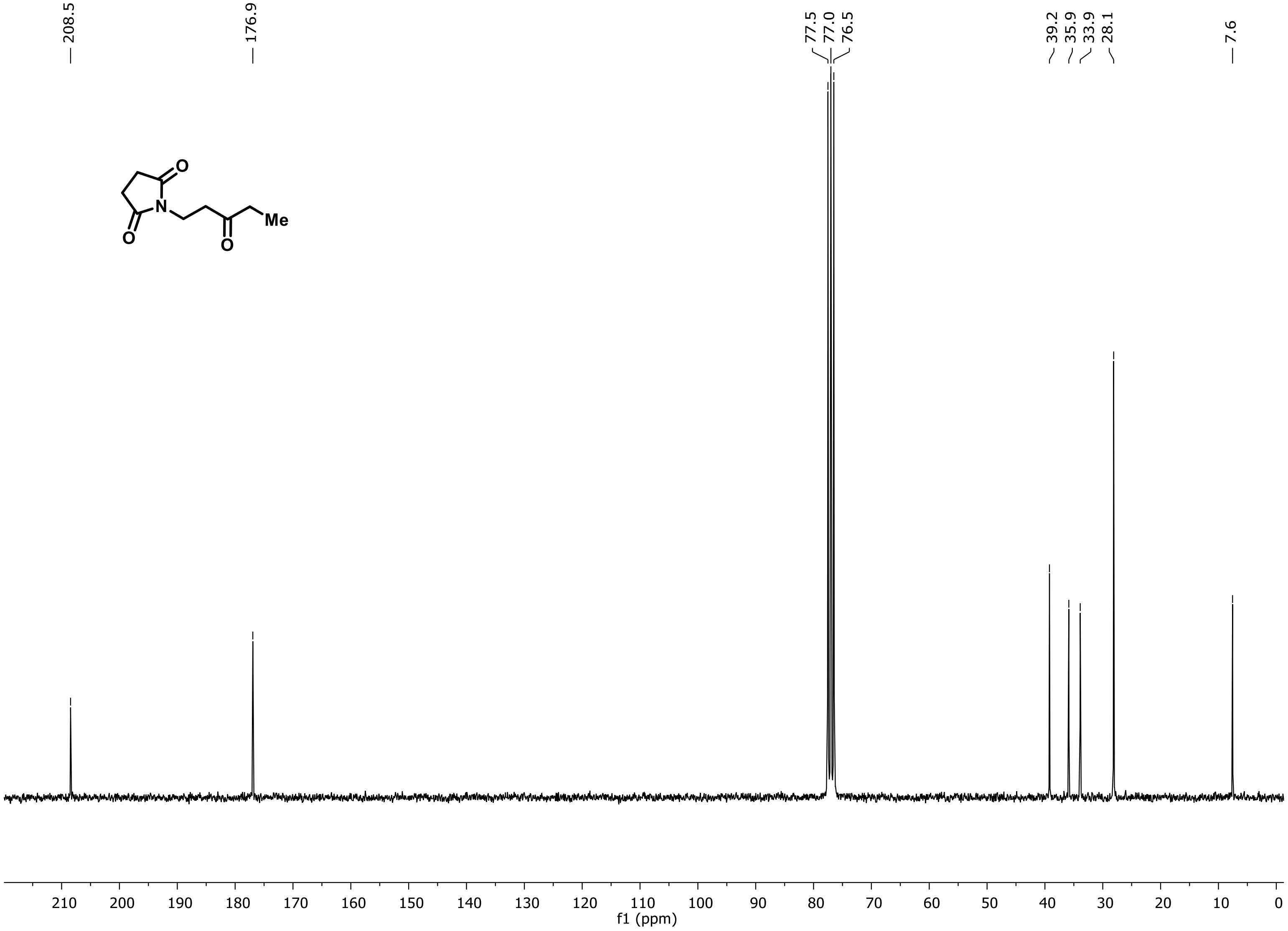

Molecule **4j**: ^{1}H NMR (250 MHz, $CDCl_3$)

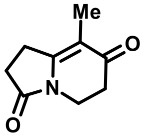

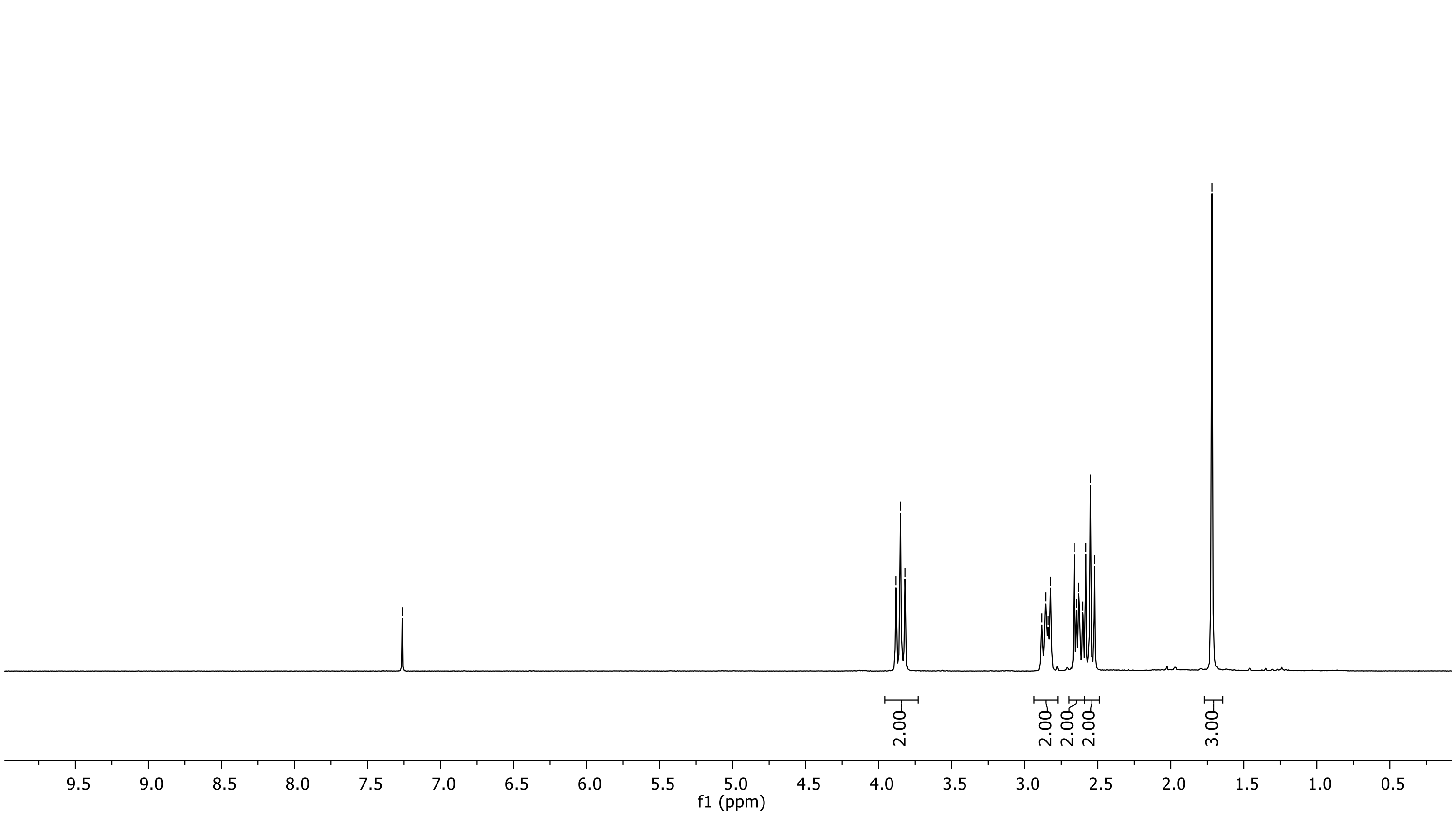

Molecule **4j**: $^{13}C\{^1H\}$ NMR (62.5 MHz, $CDCl_3$)

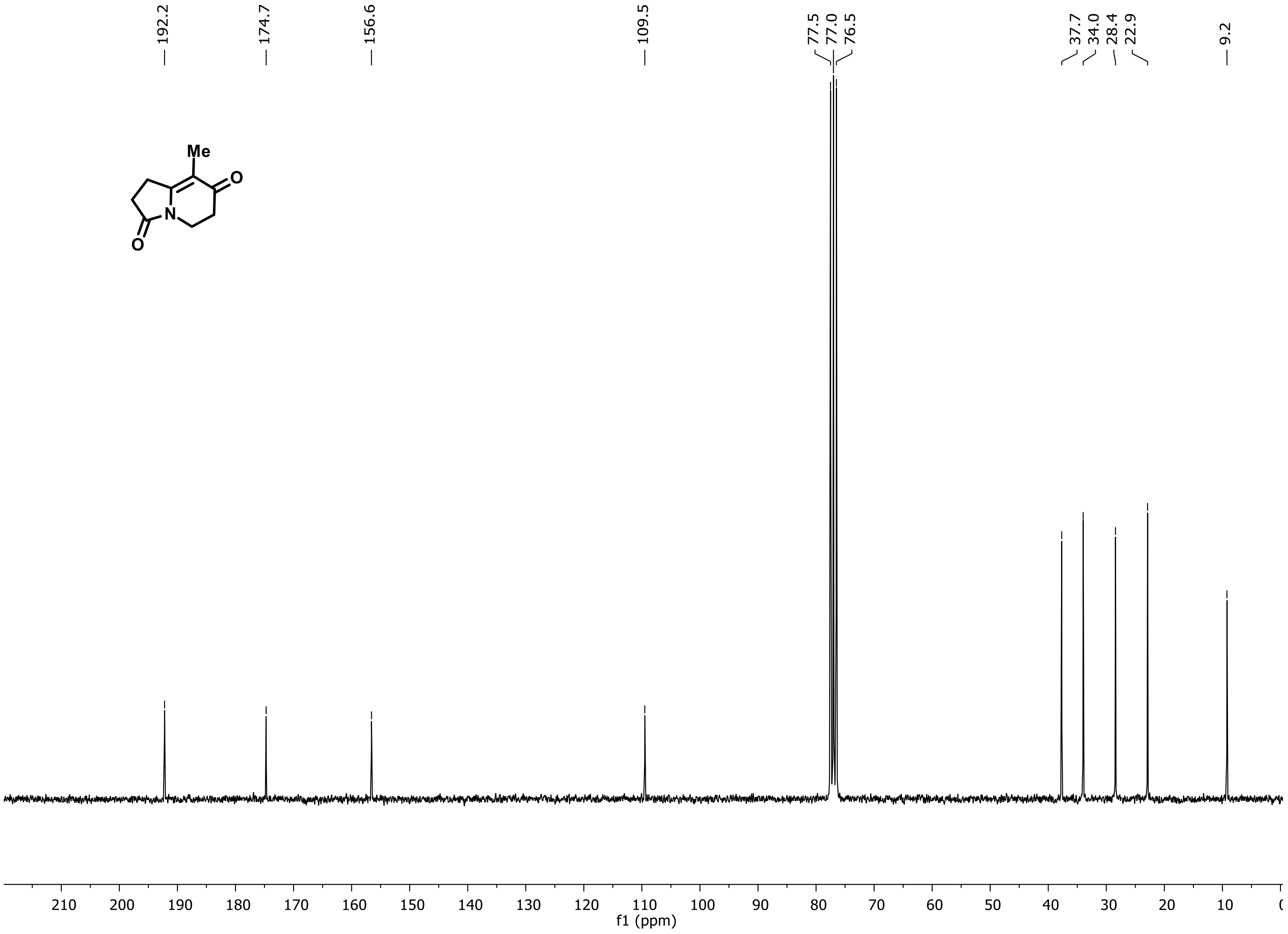

Molecule **3k**: ^{1}H NMR (250 MHz, $CDCl_3$)

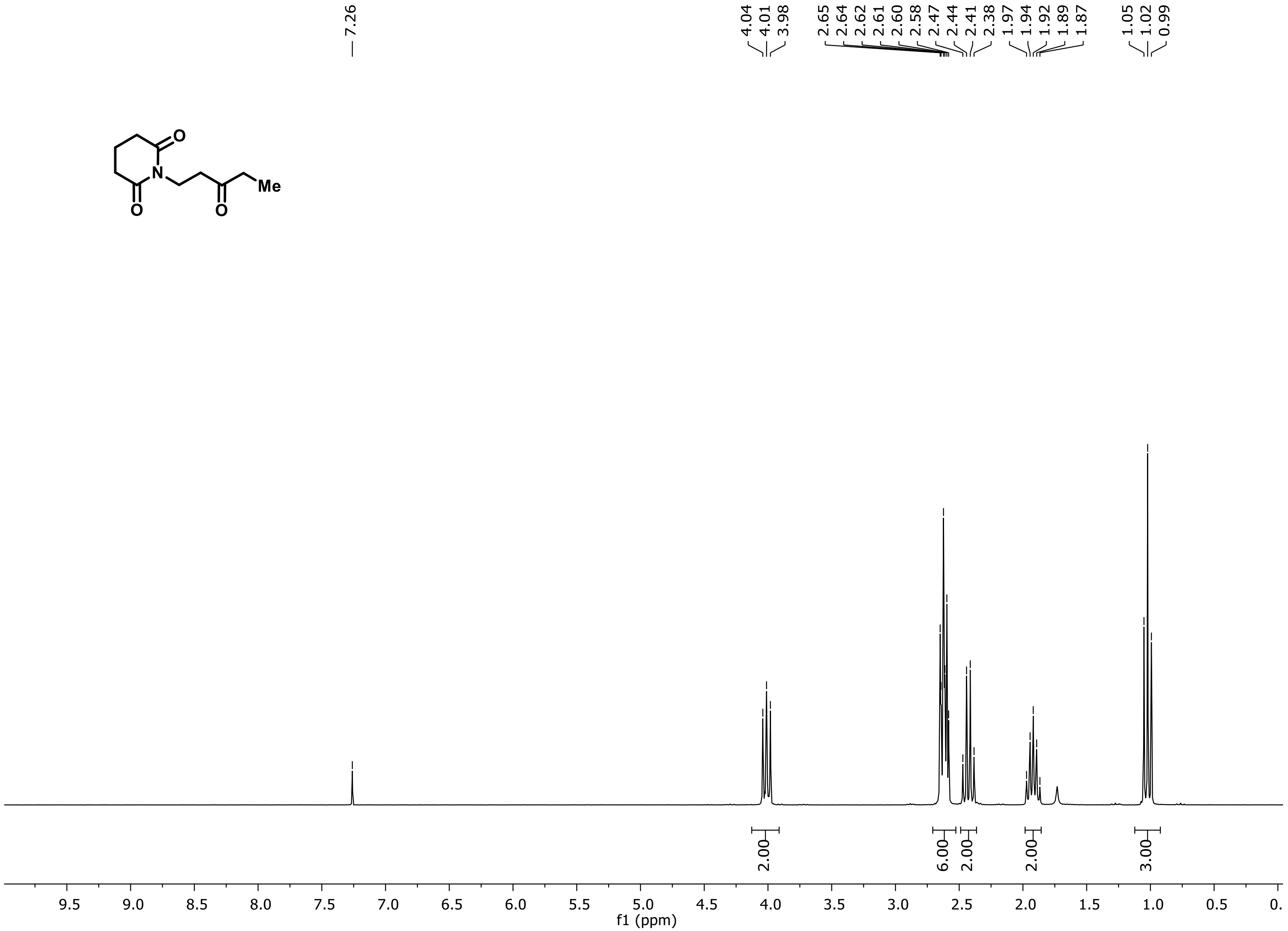

Molecule **3k**: $^{13}C\{^{1}H\}$ NMR (62.5 MHz, $CDCl_3$)

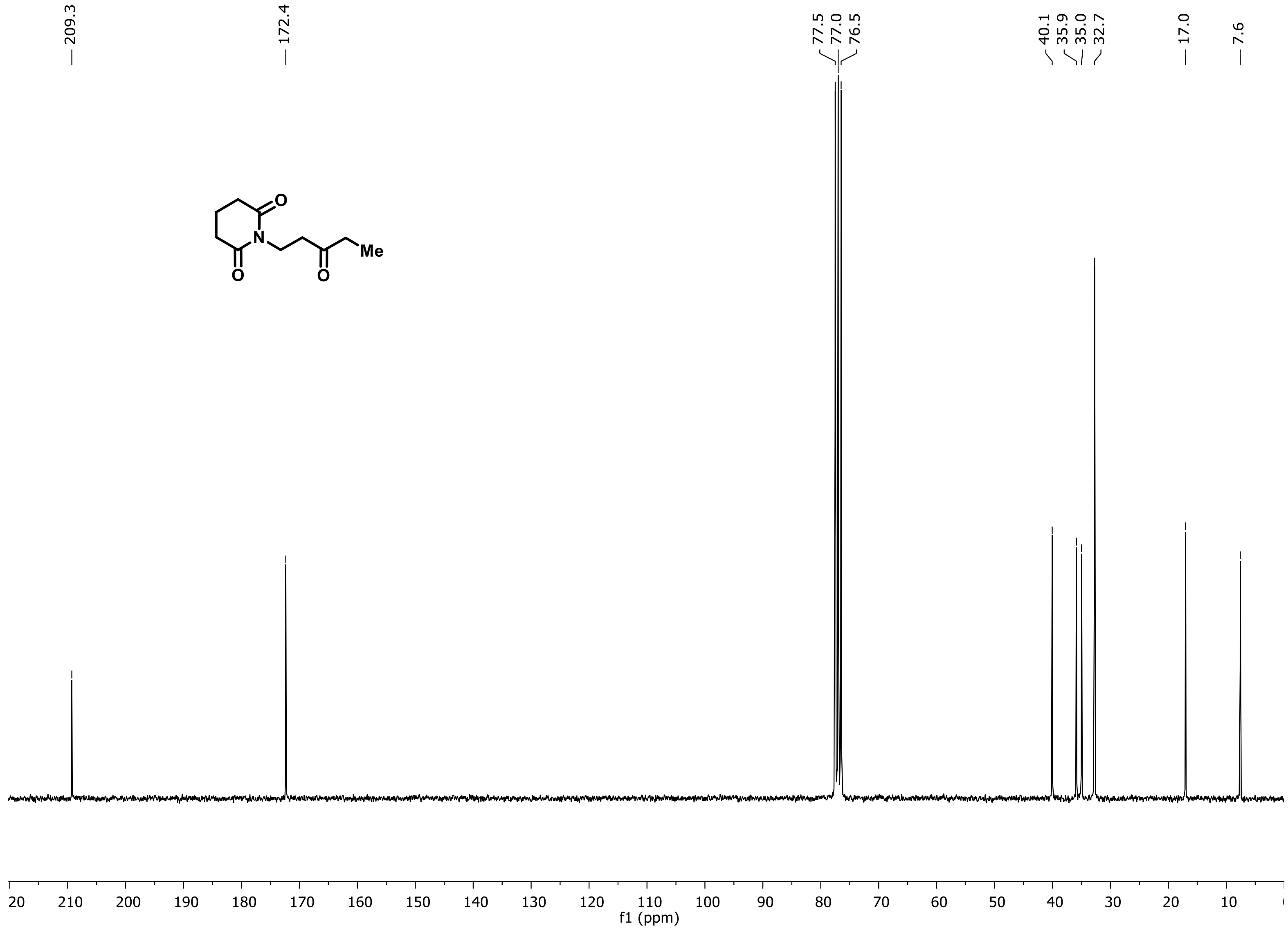

Molecule **4k**: ^{1}H NMR (250 MHz, $CDCl_3$)

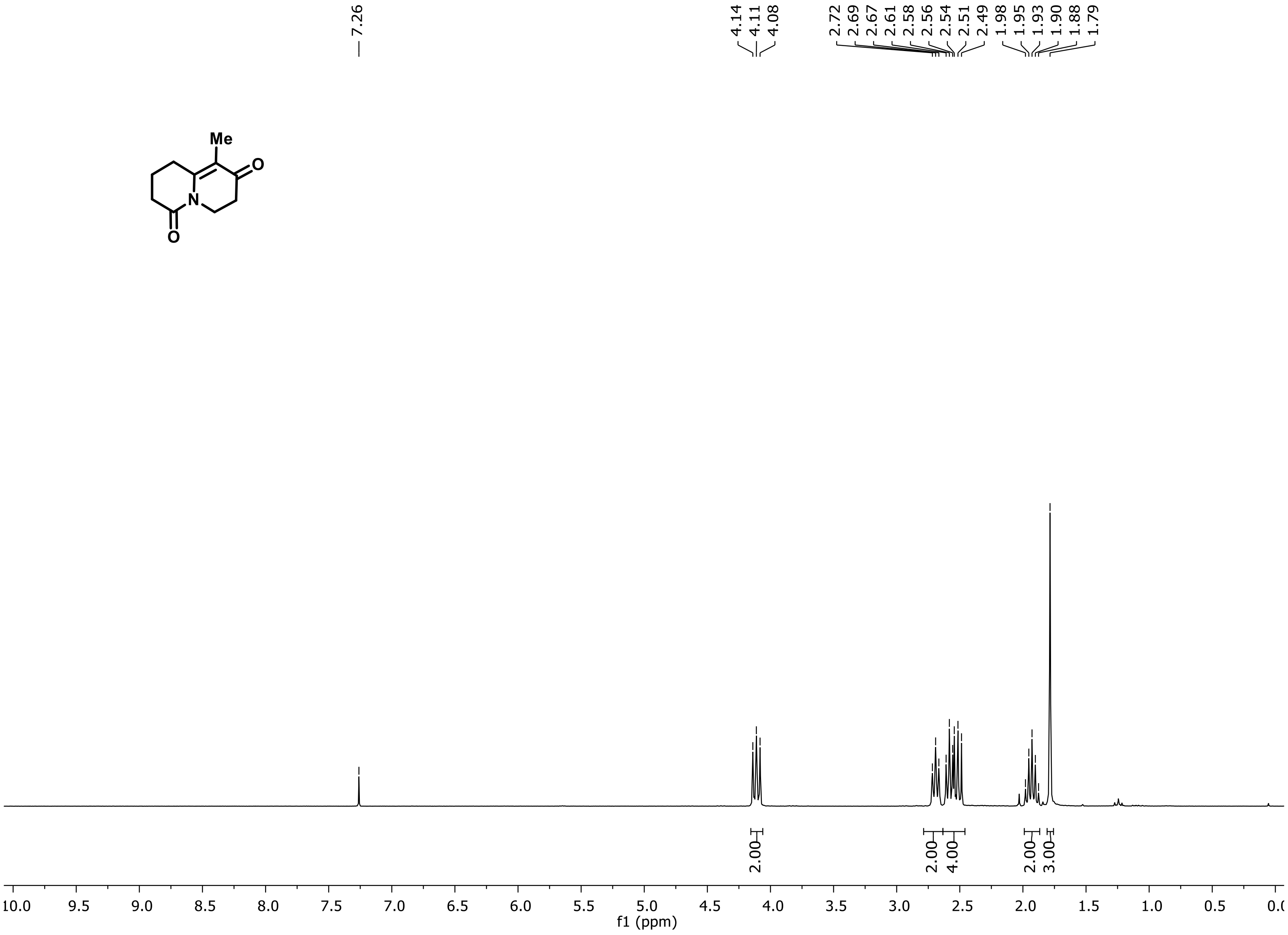

Molecule **4k**: $^{13}C\{^{1}H\}$ NMR (62.5 MHz, $CDCl_3$)

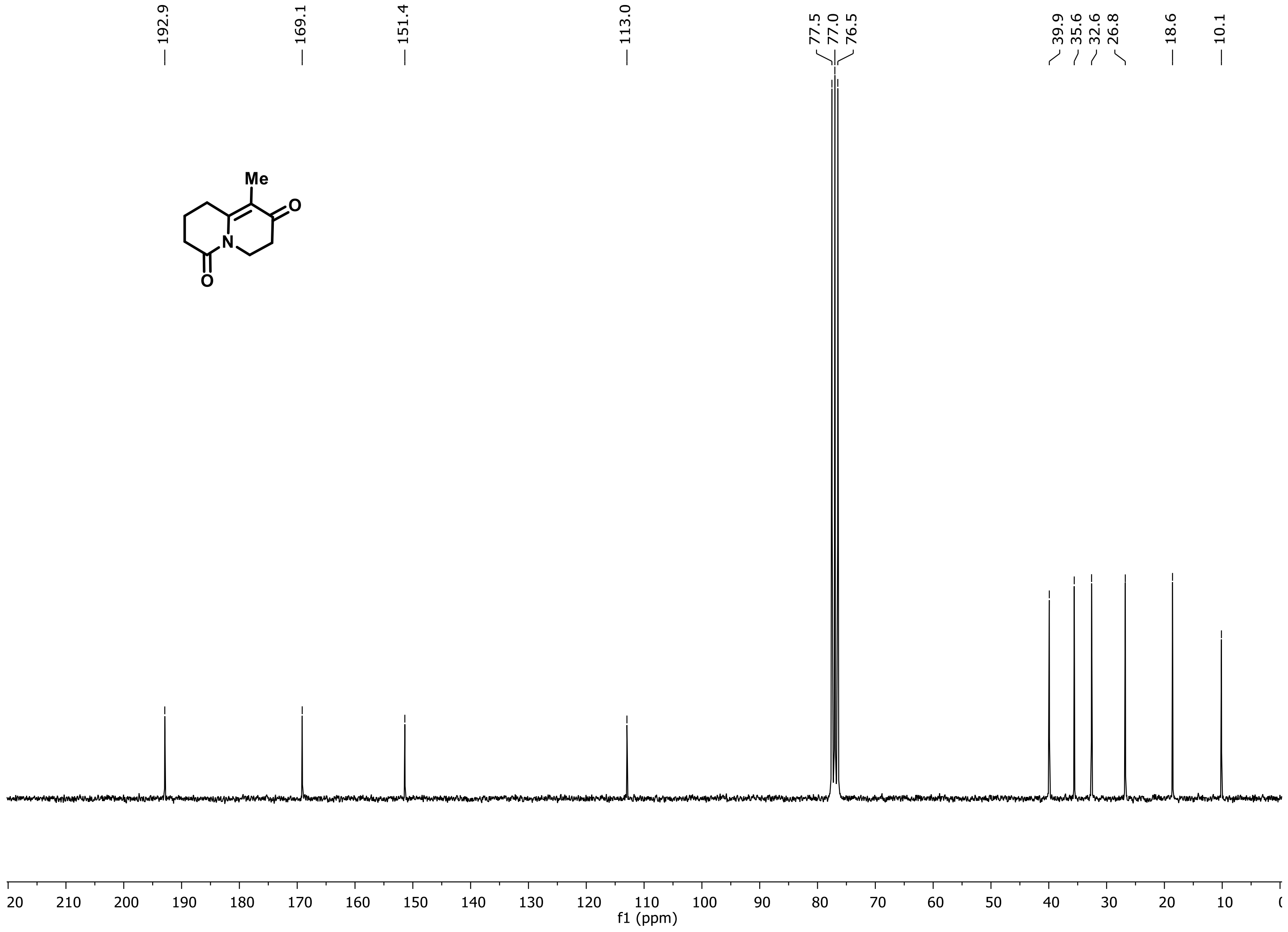

Molecule **3l**: ^{1}H NMR (250 MHz, $CDCl_3$)

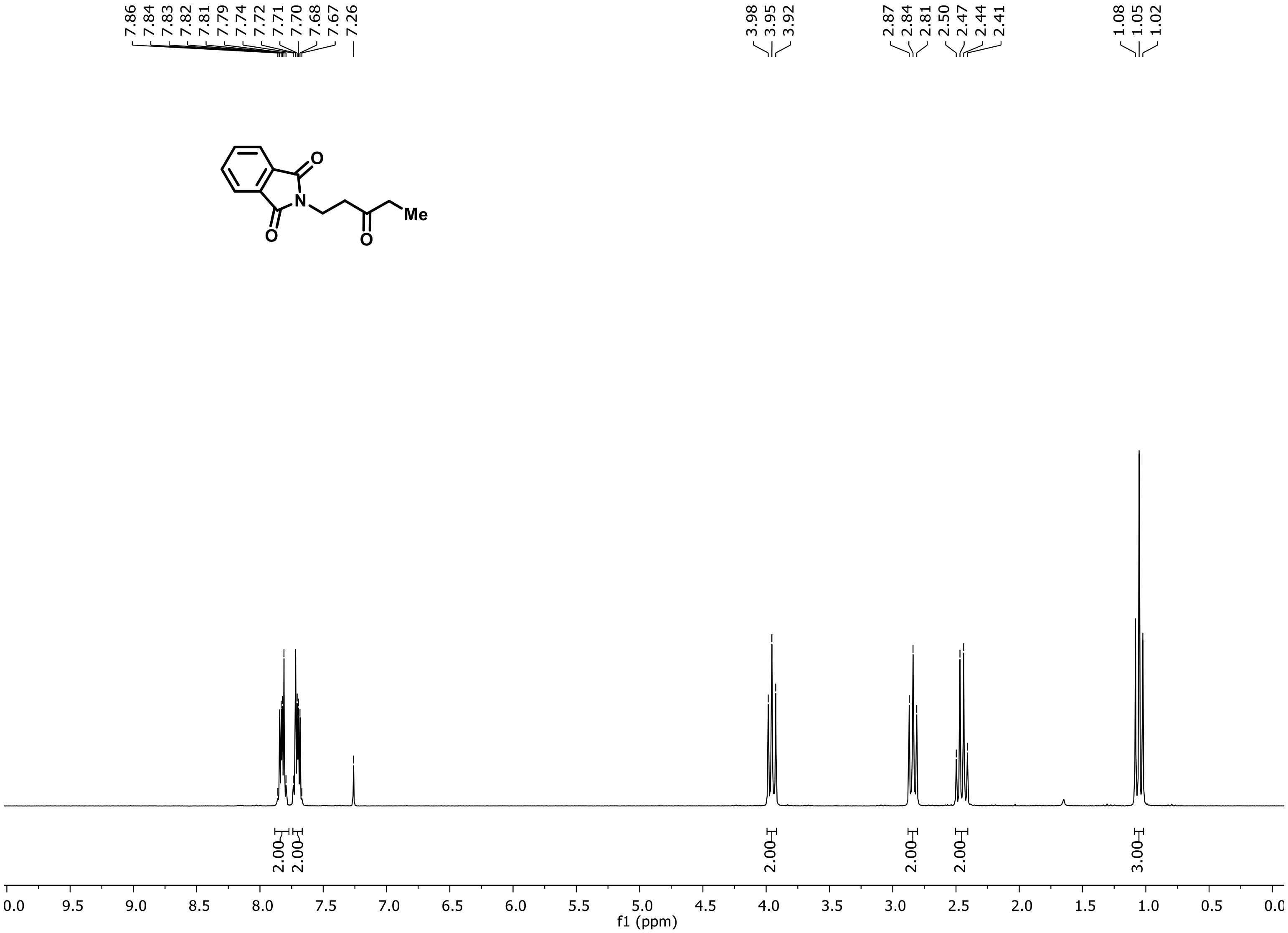

Molecule **3l**: $^{13}C\{^{1}H\}$ NMR (62.5 MHz, $CDCl_3$)

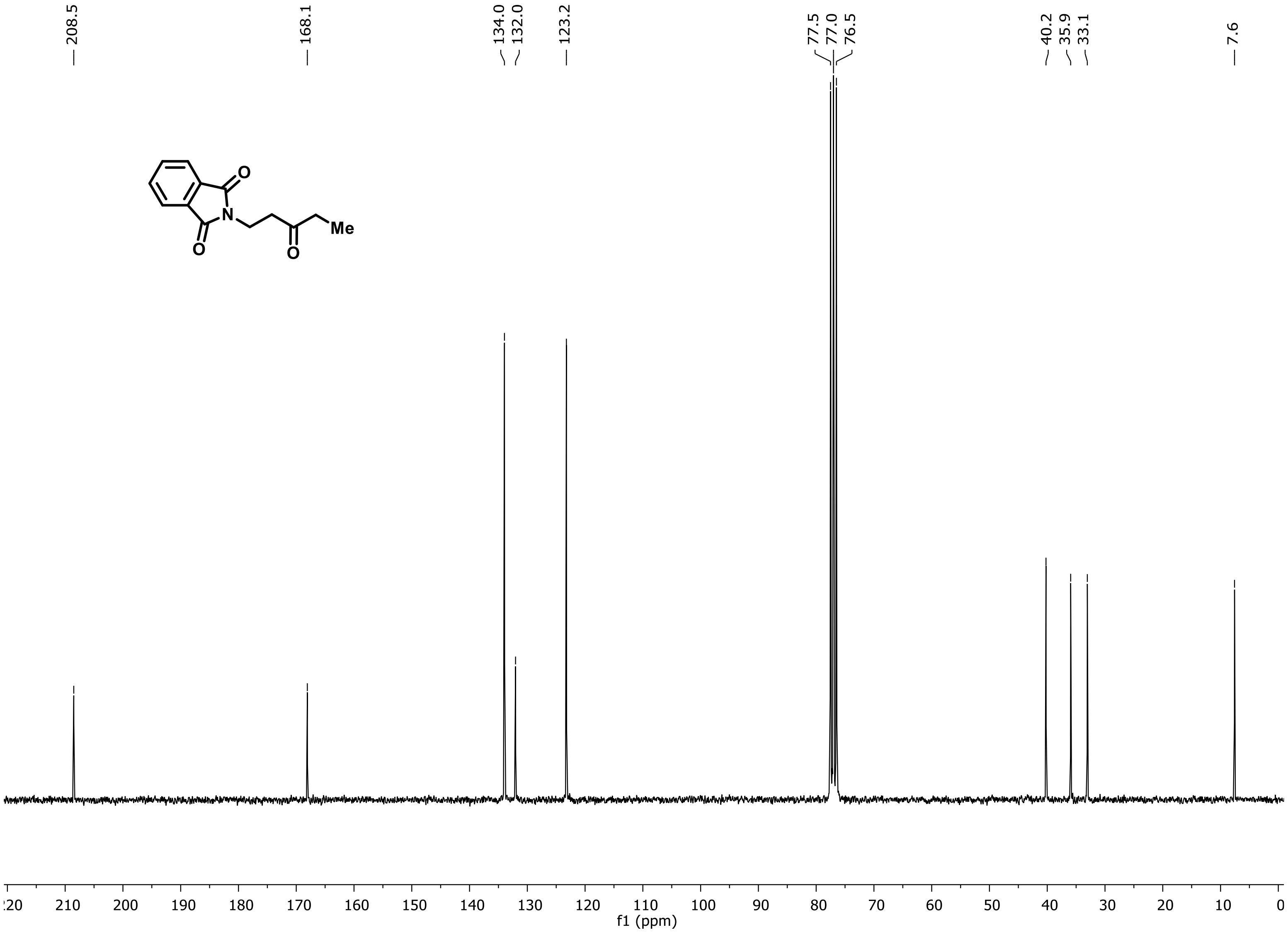

Molecule **4l**: ^{1}H NMR (250 MHz, $CDCl_3$)

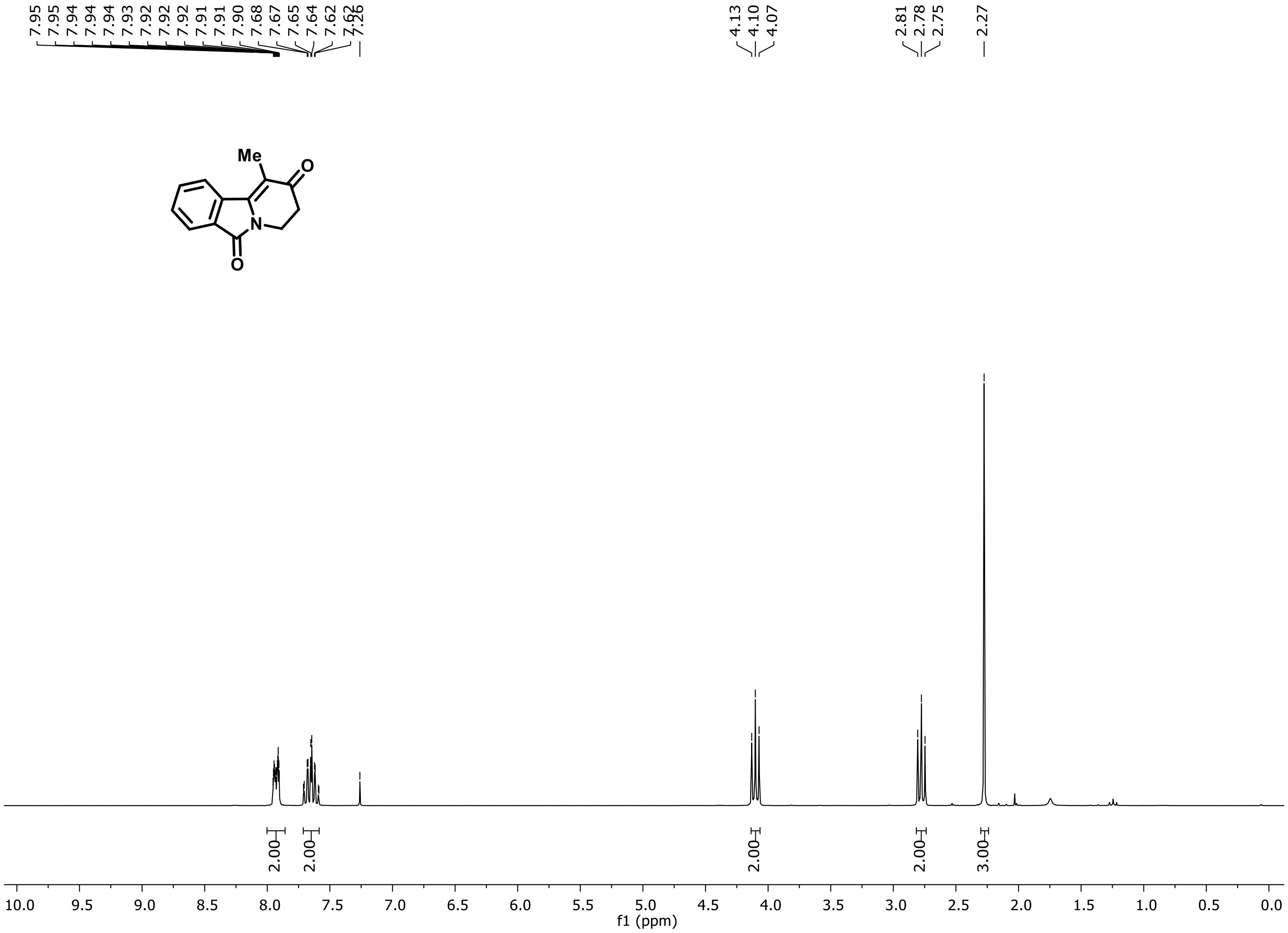

Molecule **4l**: $^{13}C\{^{1}H\}$ NMR (62.5 MHz, $CDCl_3$)

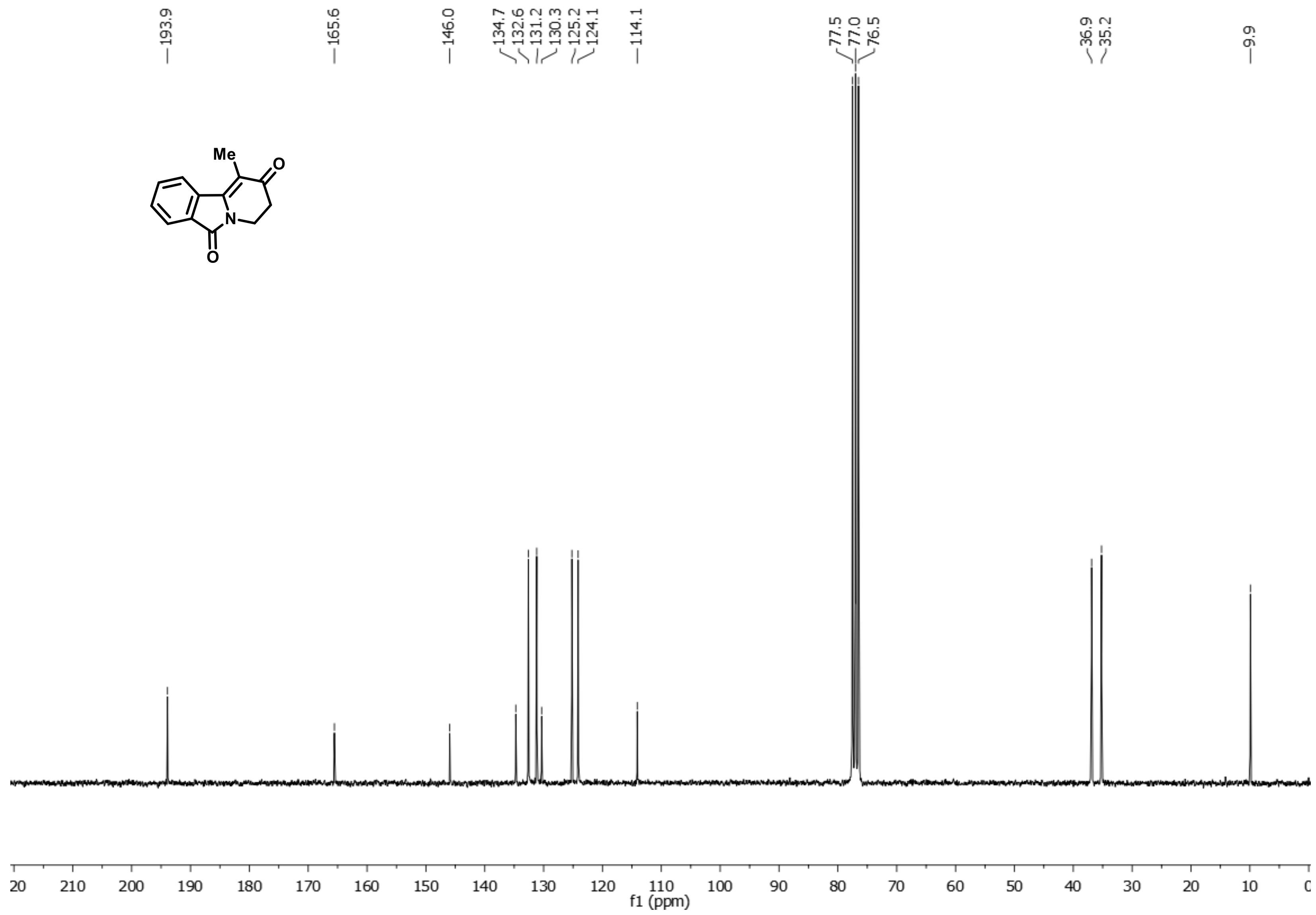

Molecule **3m**: ^{1}H NMR (250 MHz, $CDCl_3$)

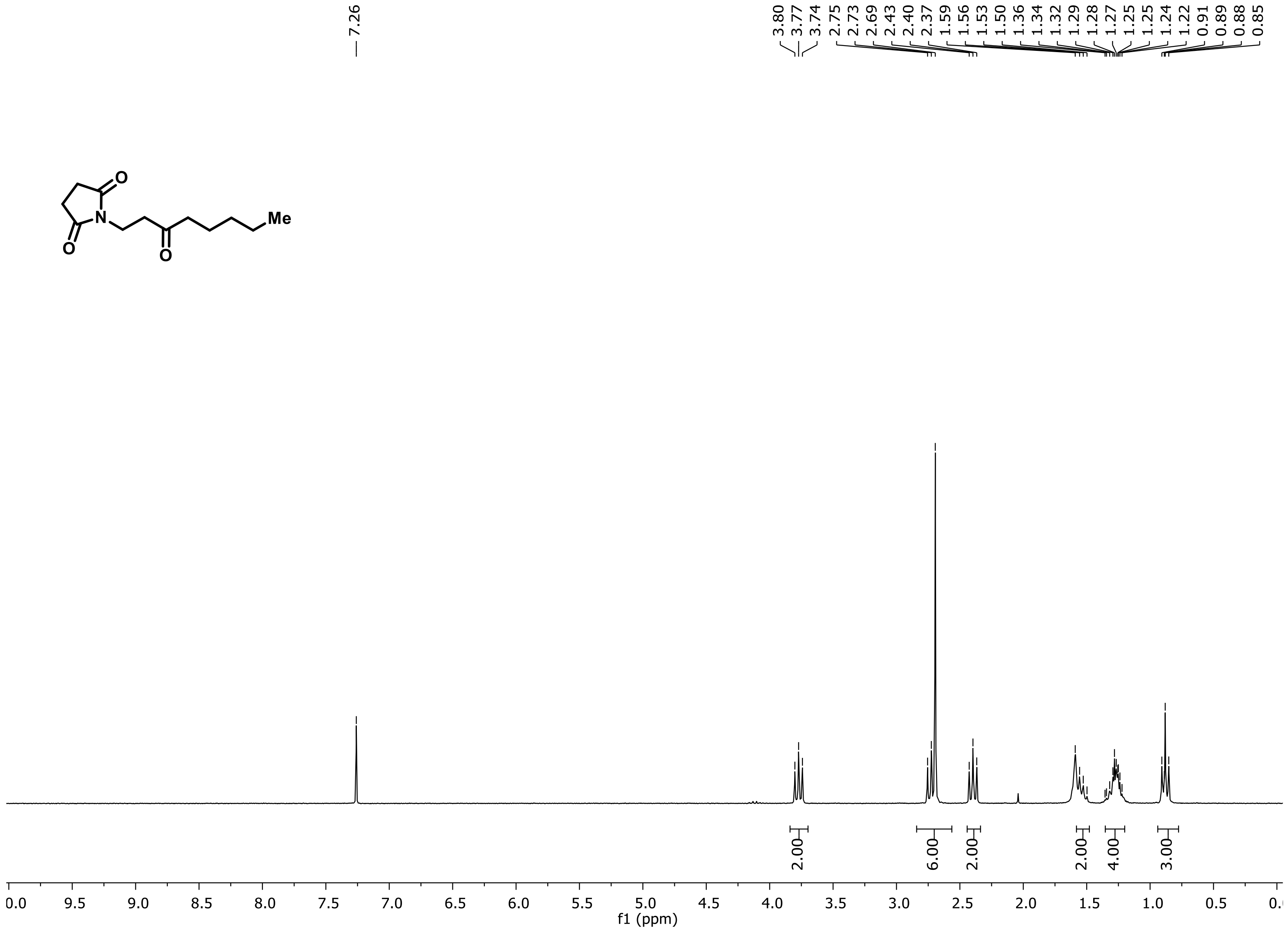

Molecule **3m**: $^{13}C\{^{1}H\}$ NMR (62.5 MHz, $CDCl_3$)

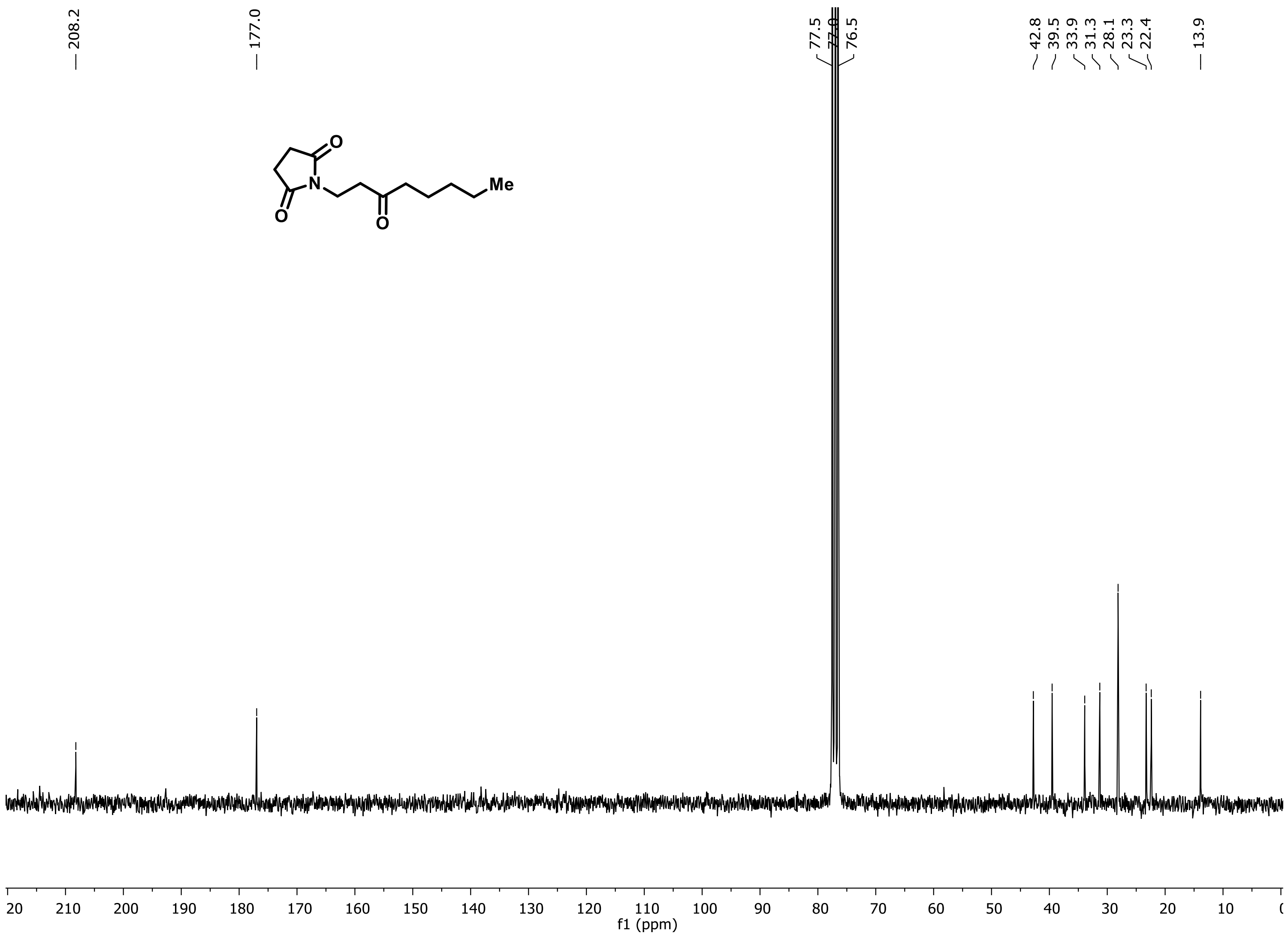

Molecule **4m**: ^{1}H NMR (250 MHz, $CDCl_3$)

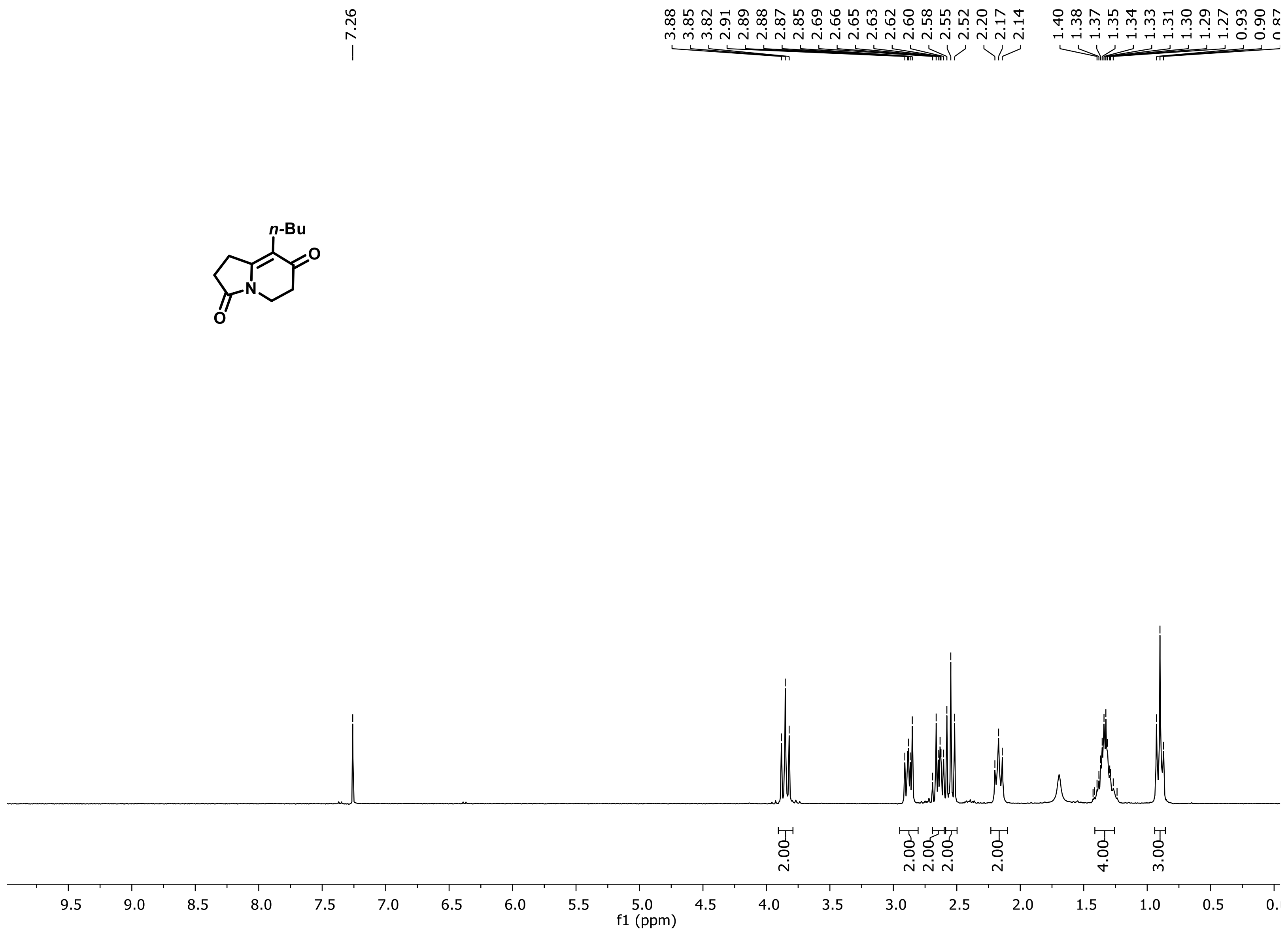

Molecule **4m**: $^{13}C\{^{1}H\}$ NMR (62.5 MHz, $CDCl_3$)

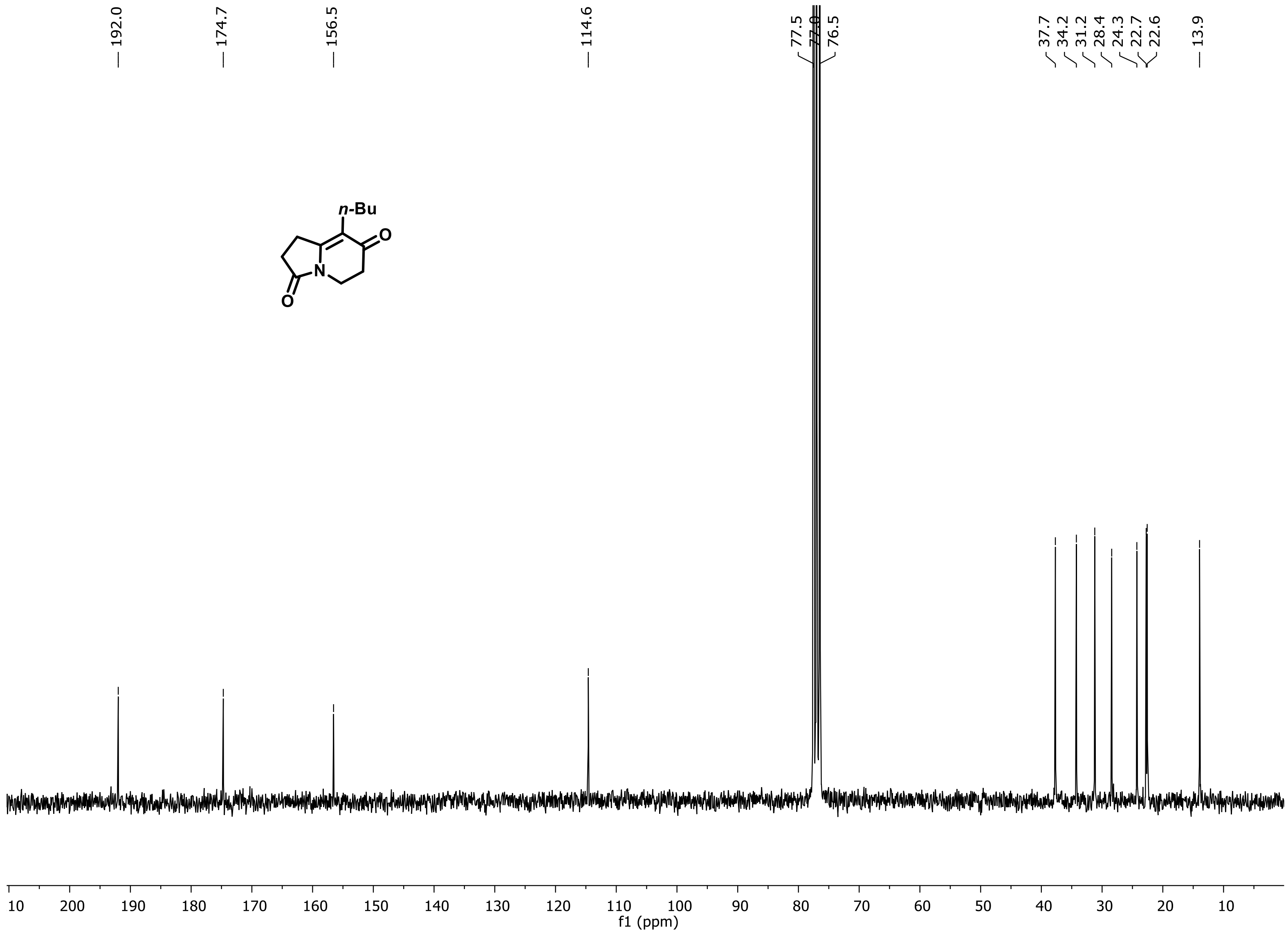

Molecule **3n**: ^{1}H NMR (250 MHz, $CDCl_3$)

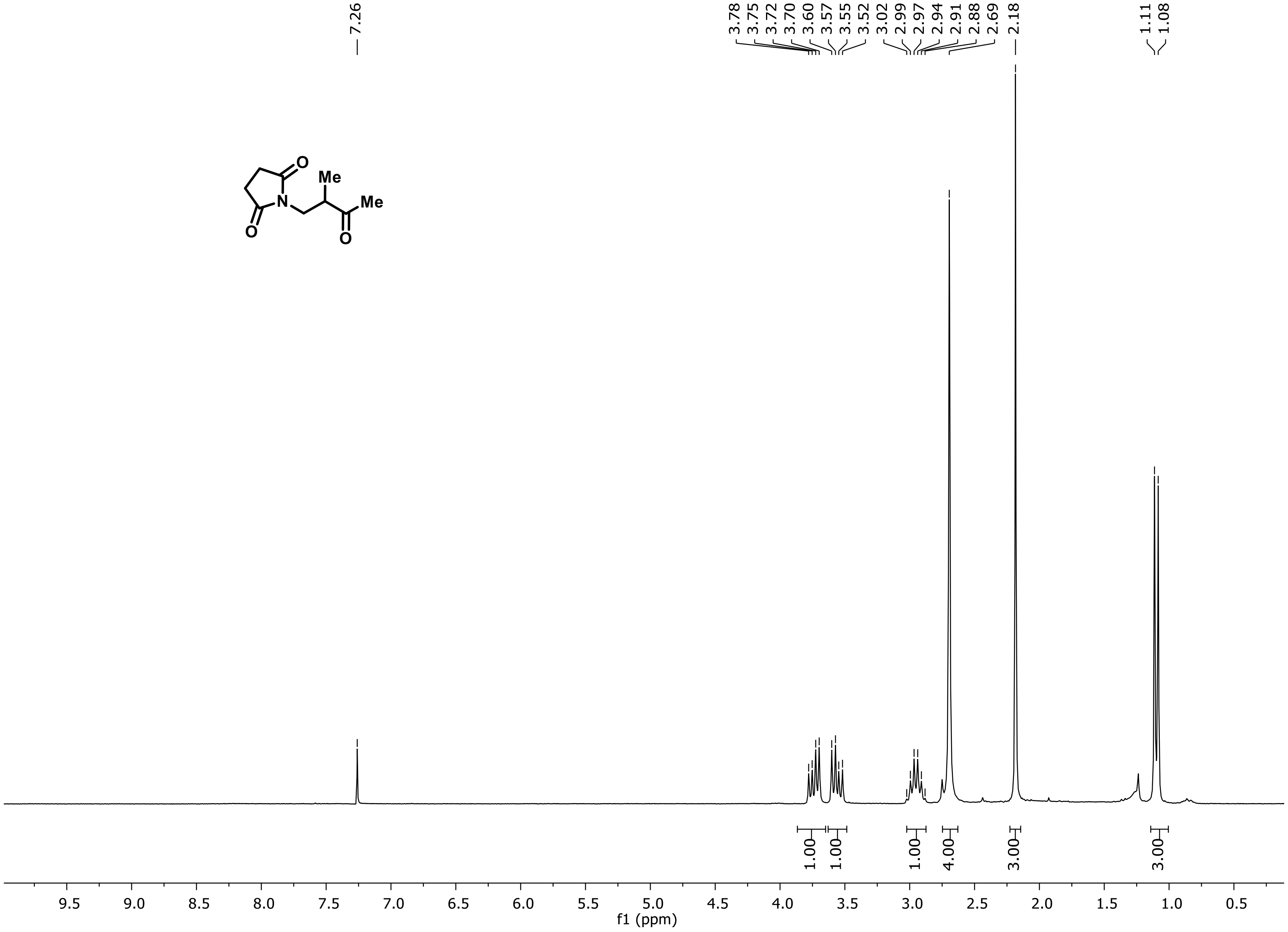

Molecule **3n**: $^{13}C\{^{1}H\}$ NMR (62.5 MHz, $CDCl_3$)

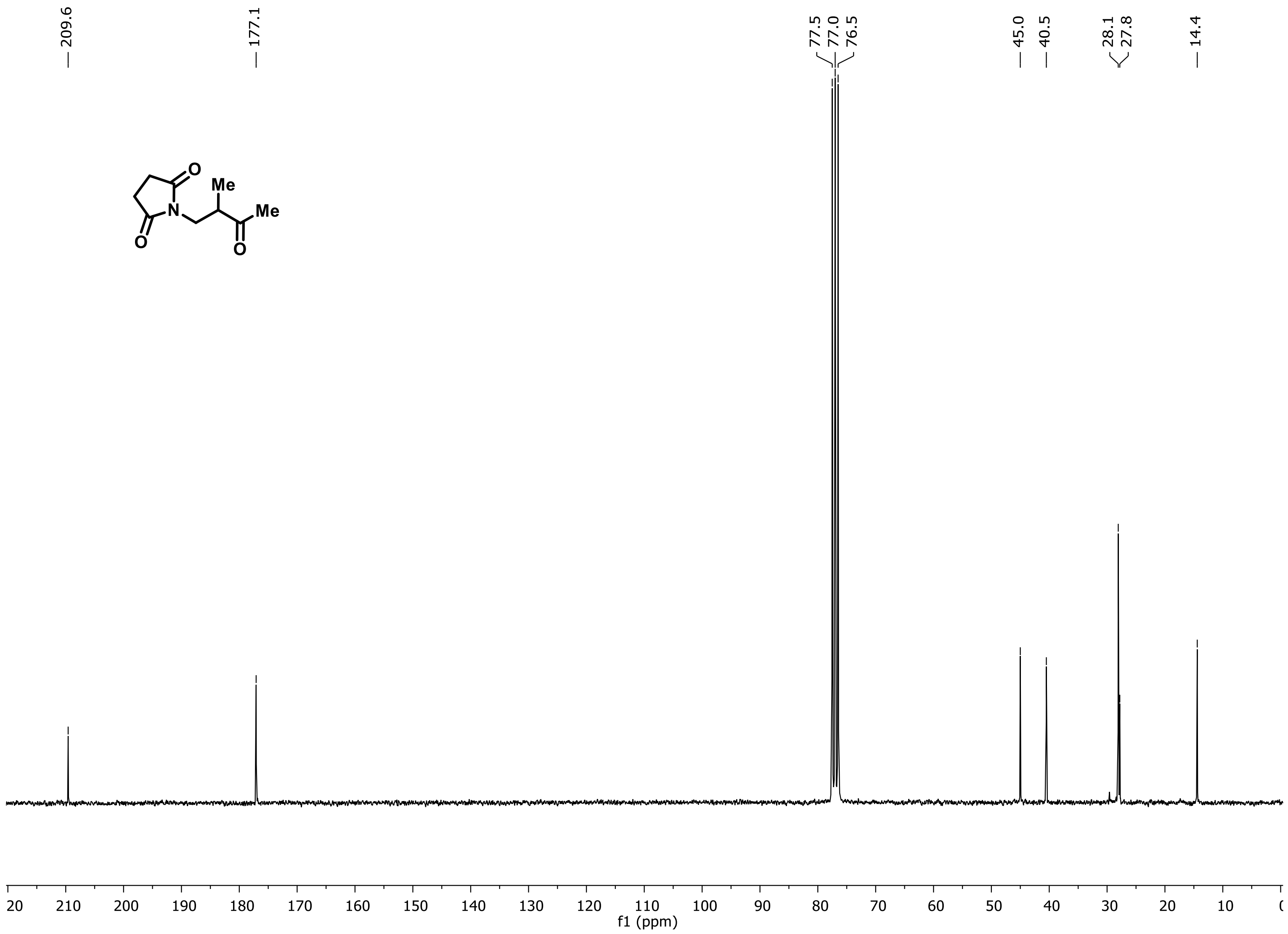

Molecule **4n**: ^{1}H NMR (250 MHz, $CDCl_3$)

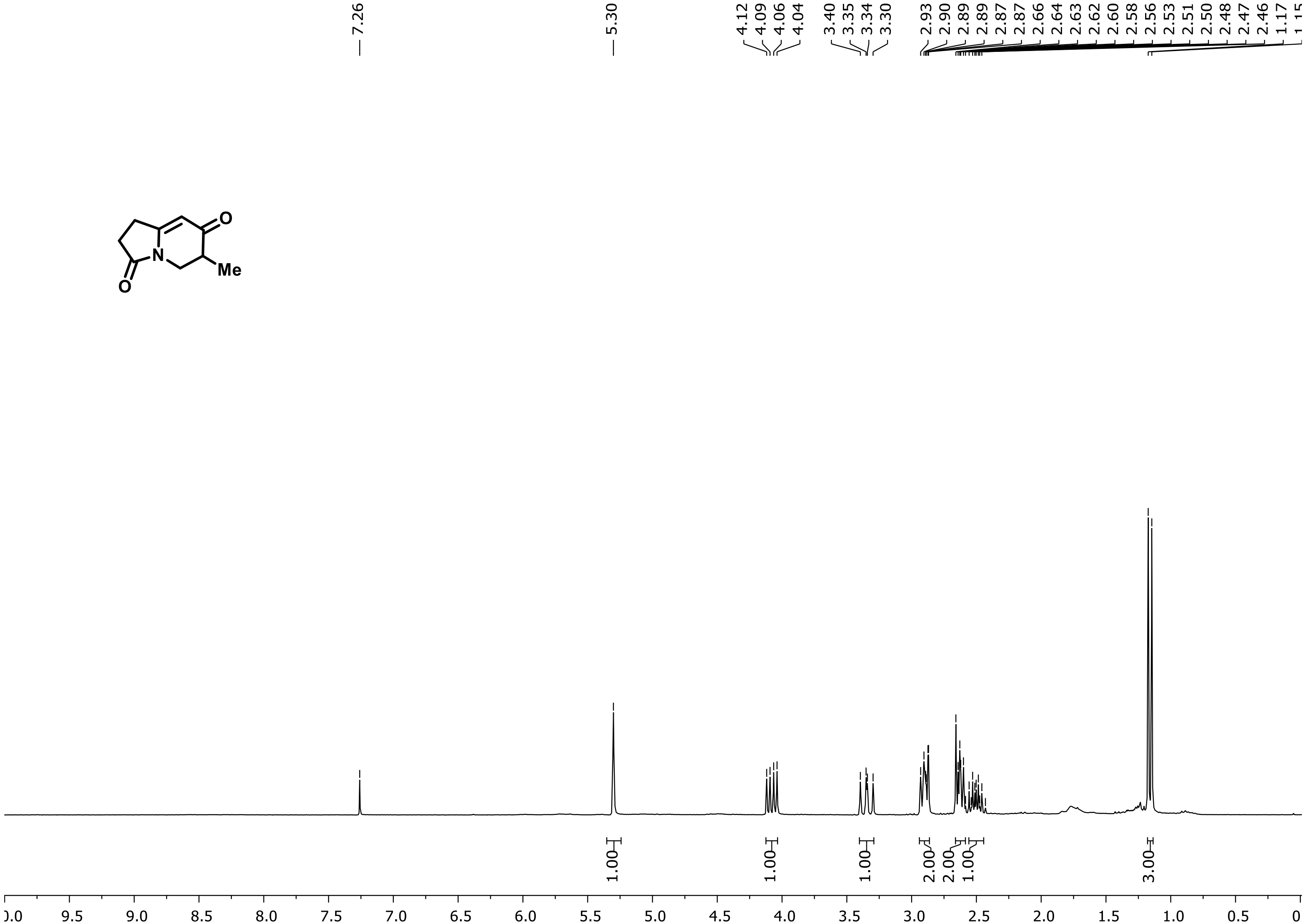

Molecule **4n**: $^{13}C\{^{1}H\}$ NMR (62.5 MHz, $CDCl_3$)

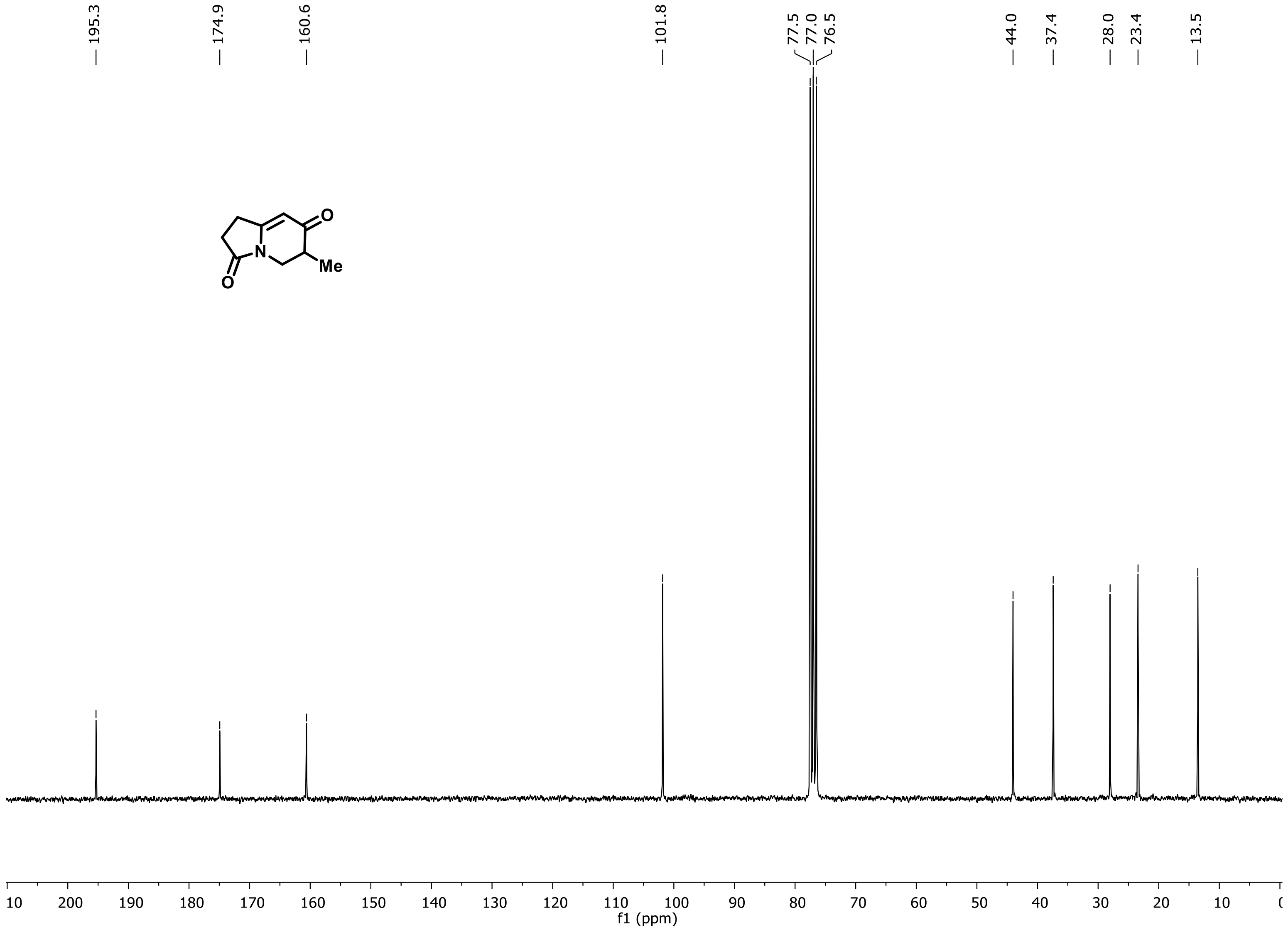

Molecule **3o**: ^{1}H NMR (250 MHz, $CDCl_3$)

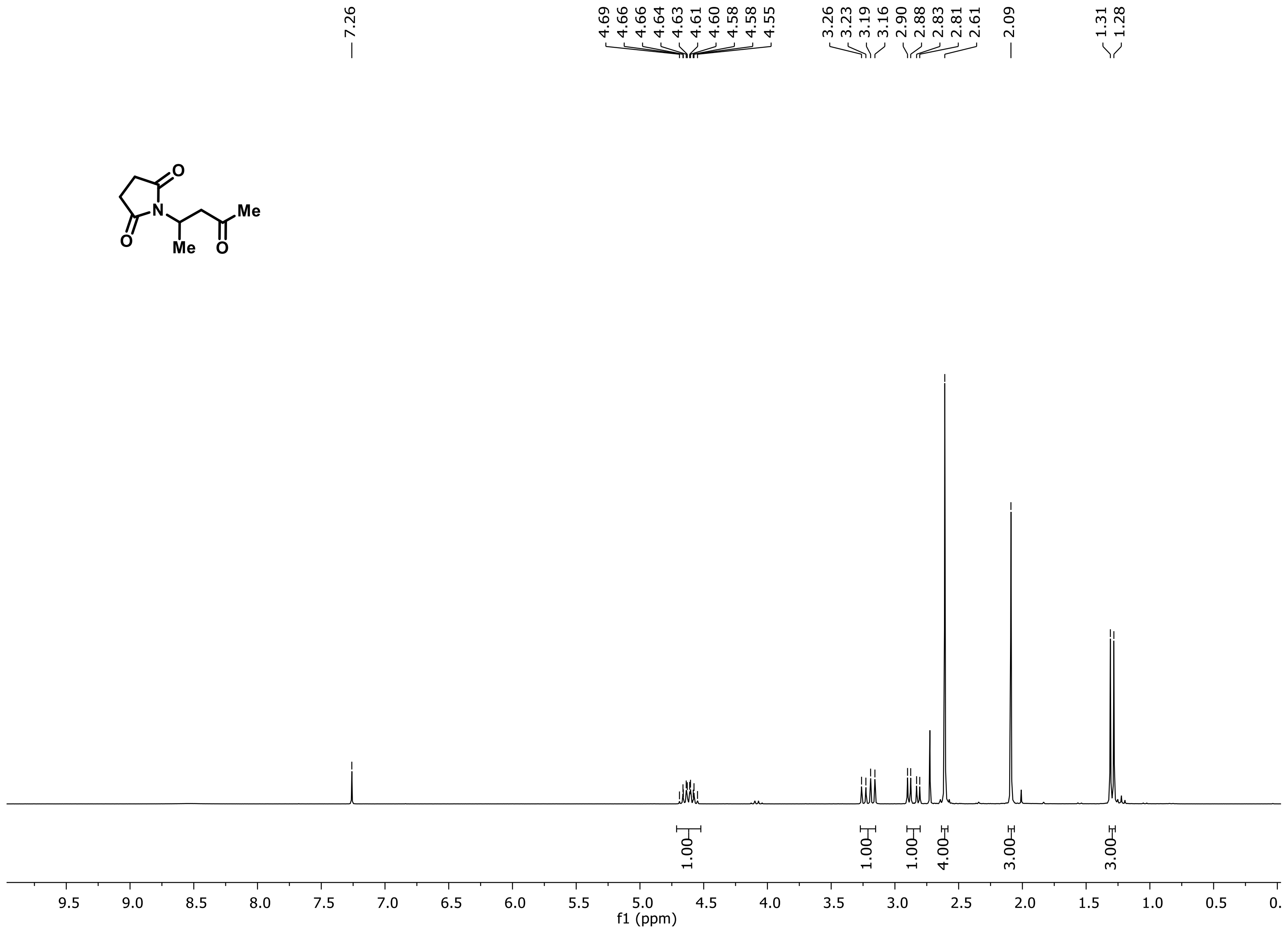

Molecule **3o**: $^{13}C\{^{1}H\}$ NMR (62.5 MHz, $CDCl_3$)

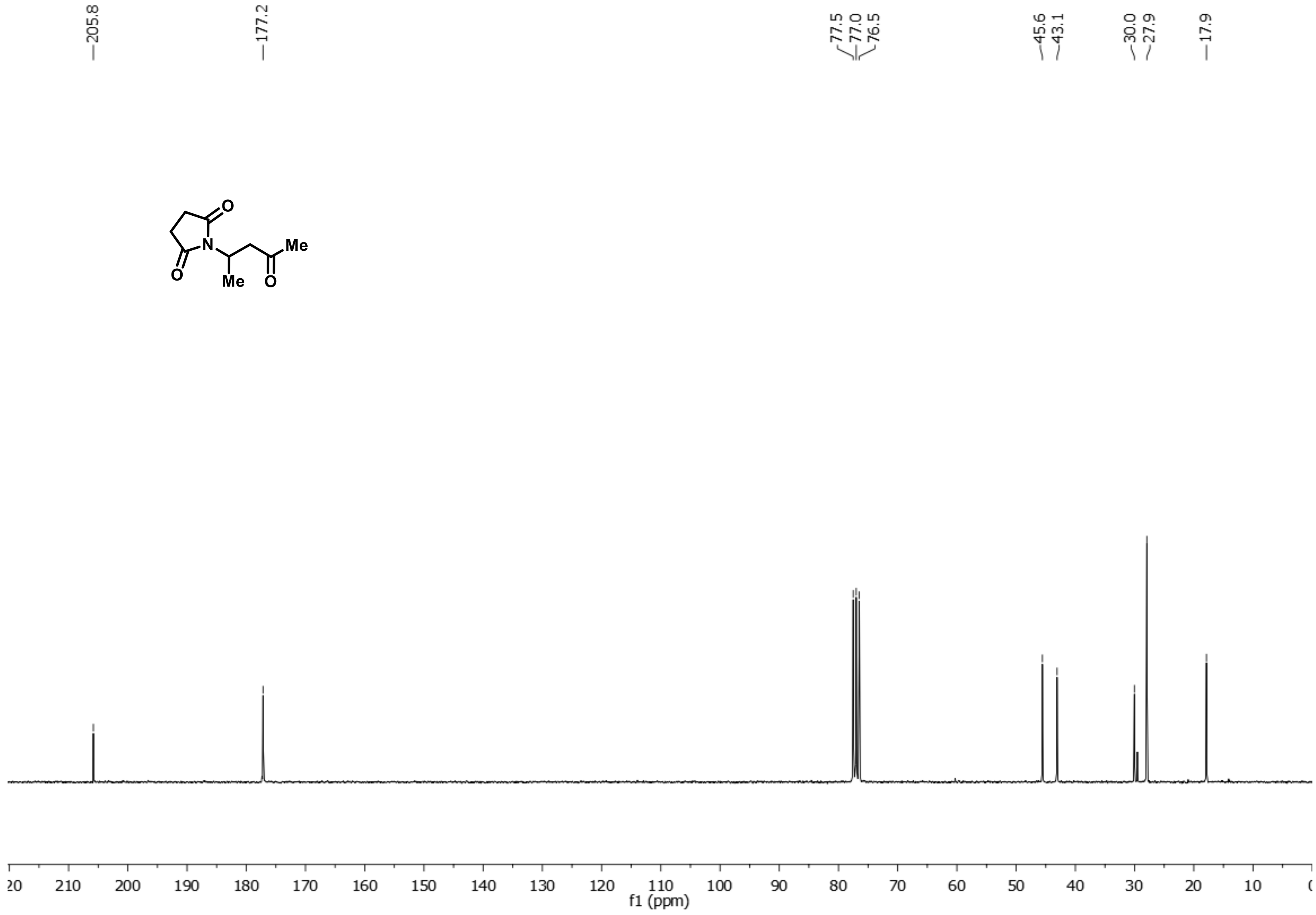

Molecule **4o**: ^{1}H NMR (250 MHz, $CDCl_3$)

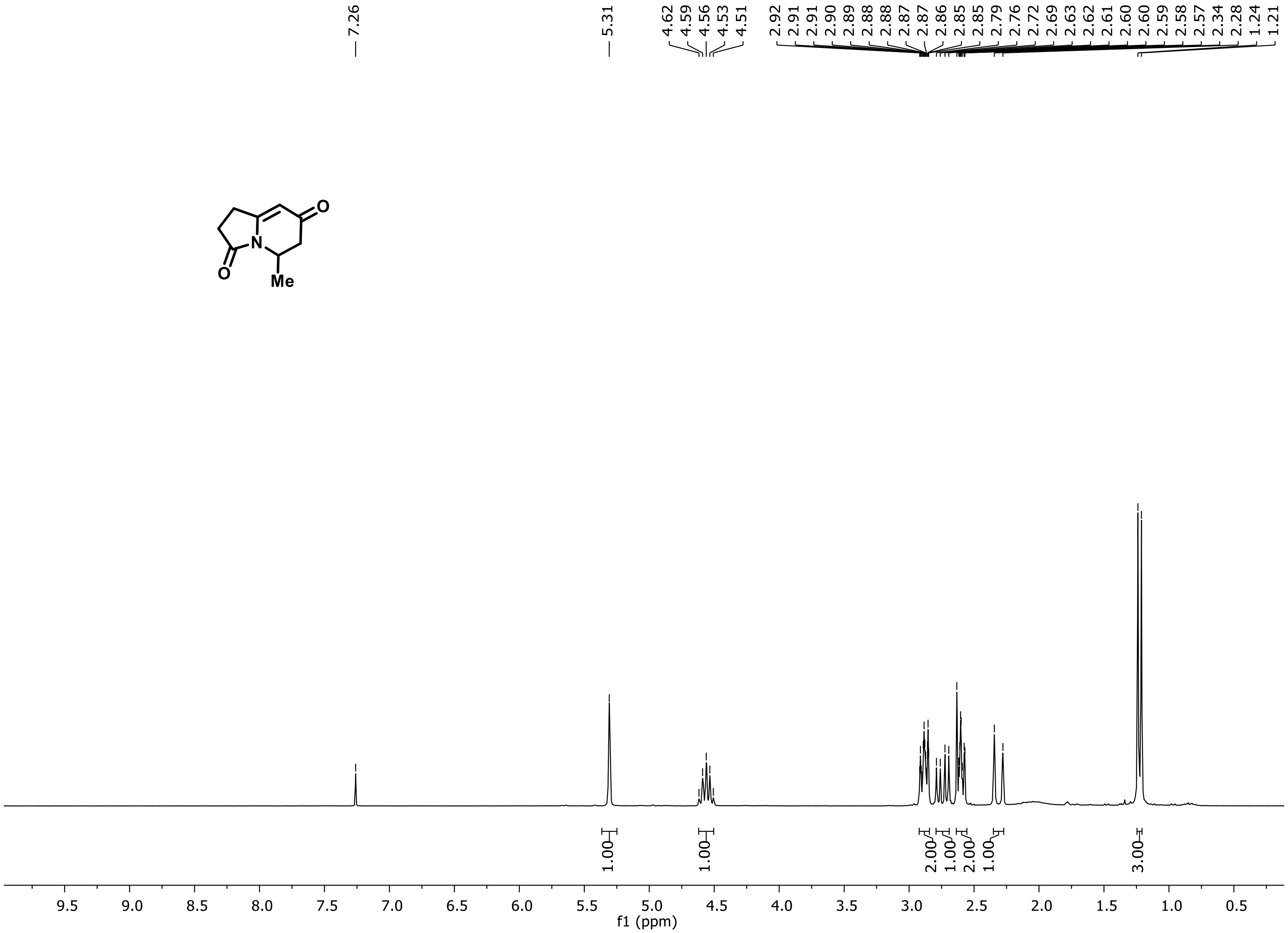

Molecule **4o**: $^{13}C\{^{1}H\}$ NMR (62.5 MHz, $CDCl_3$)

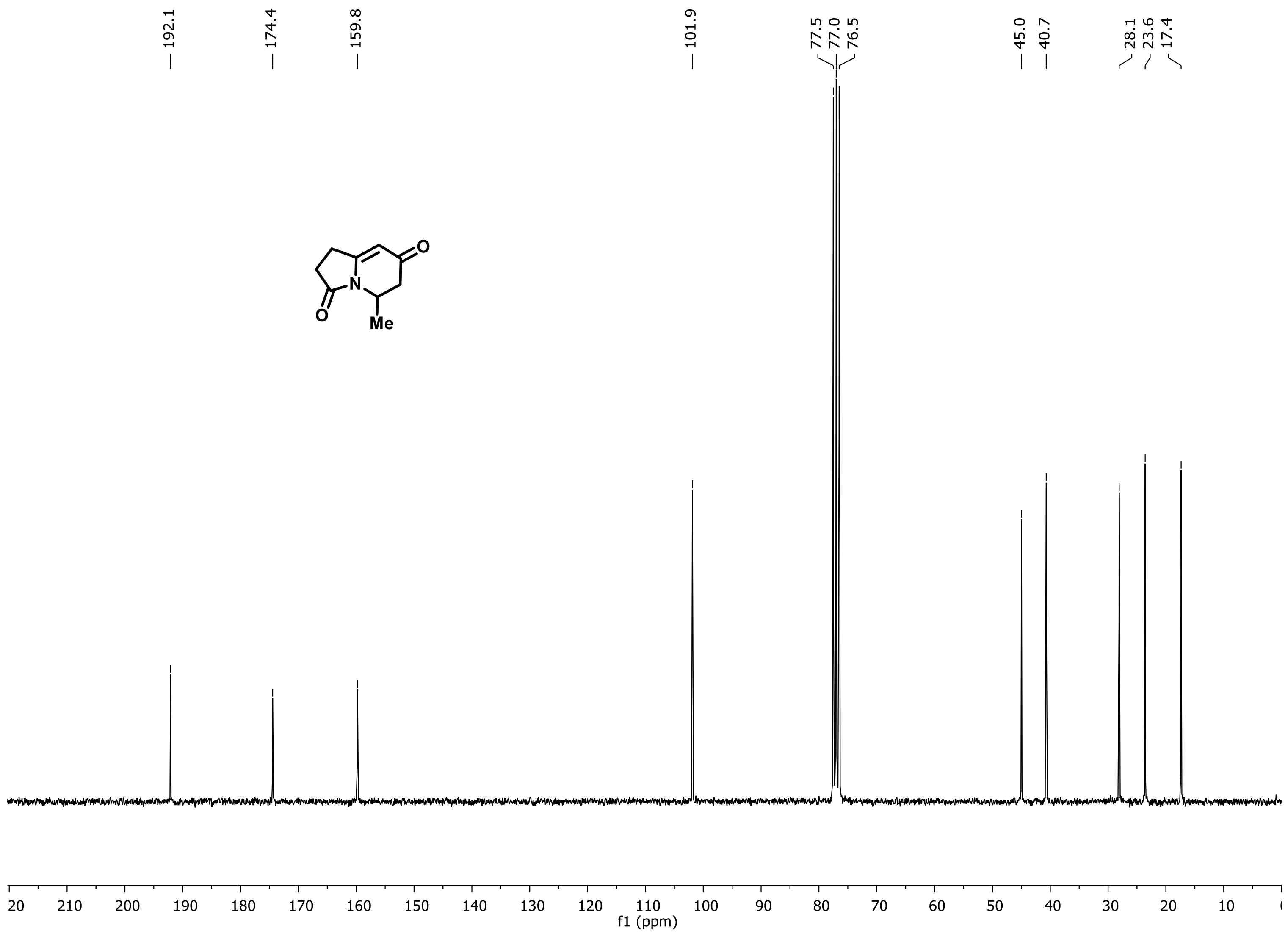

Molecule **3p**: ^1H NMR (250 MHz, $CDCl_3$)

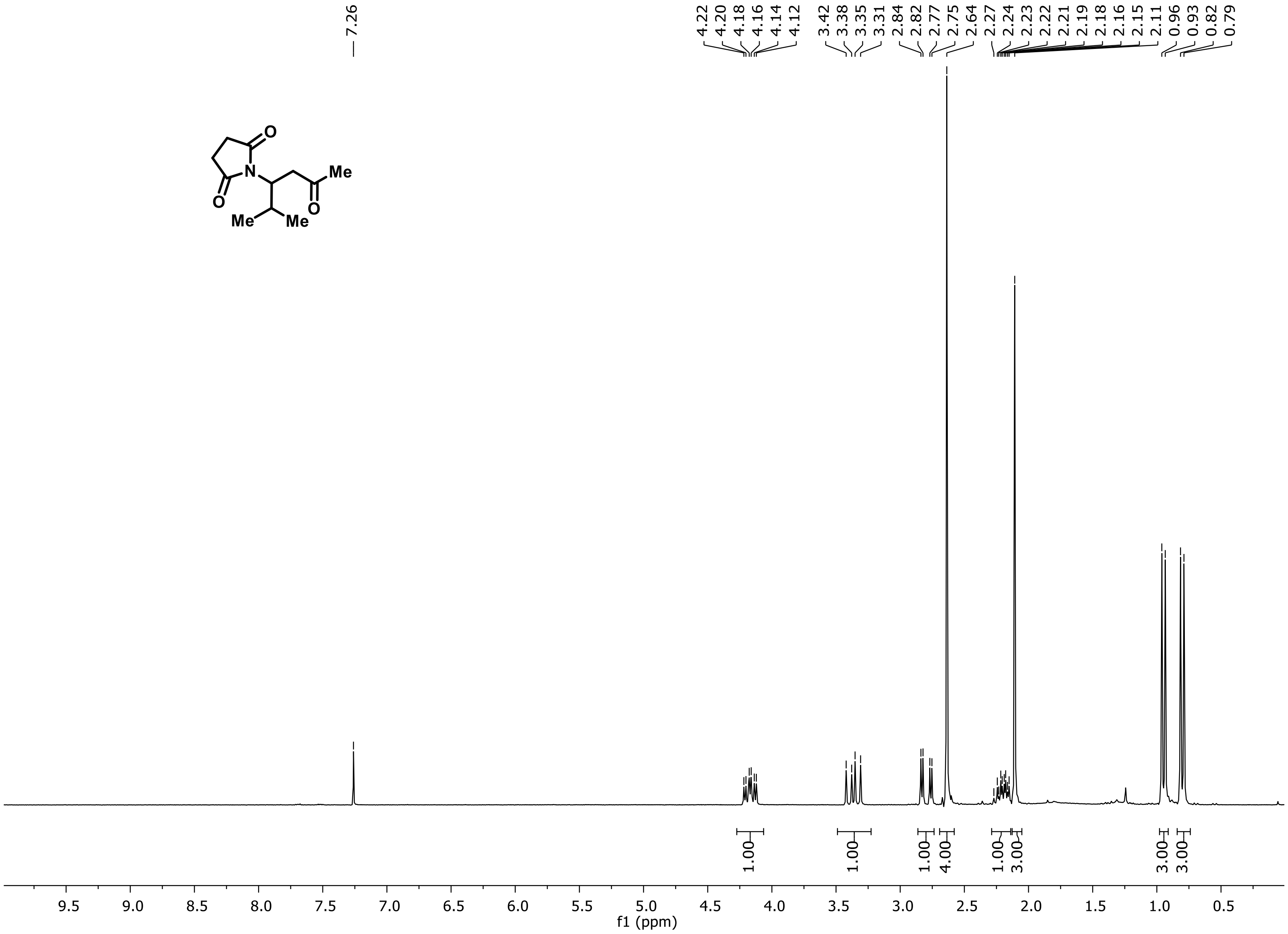

Molecule **3p**: $^{13}C\{^{1}H\}$ NMR (62.5 MHz, $CDCl_3$)

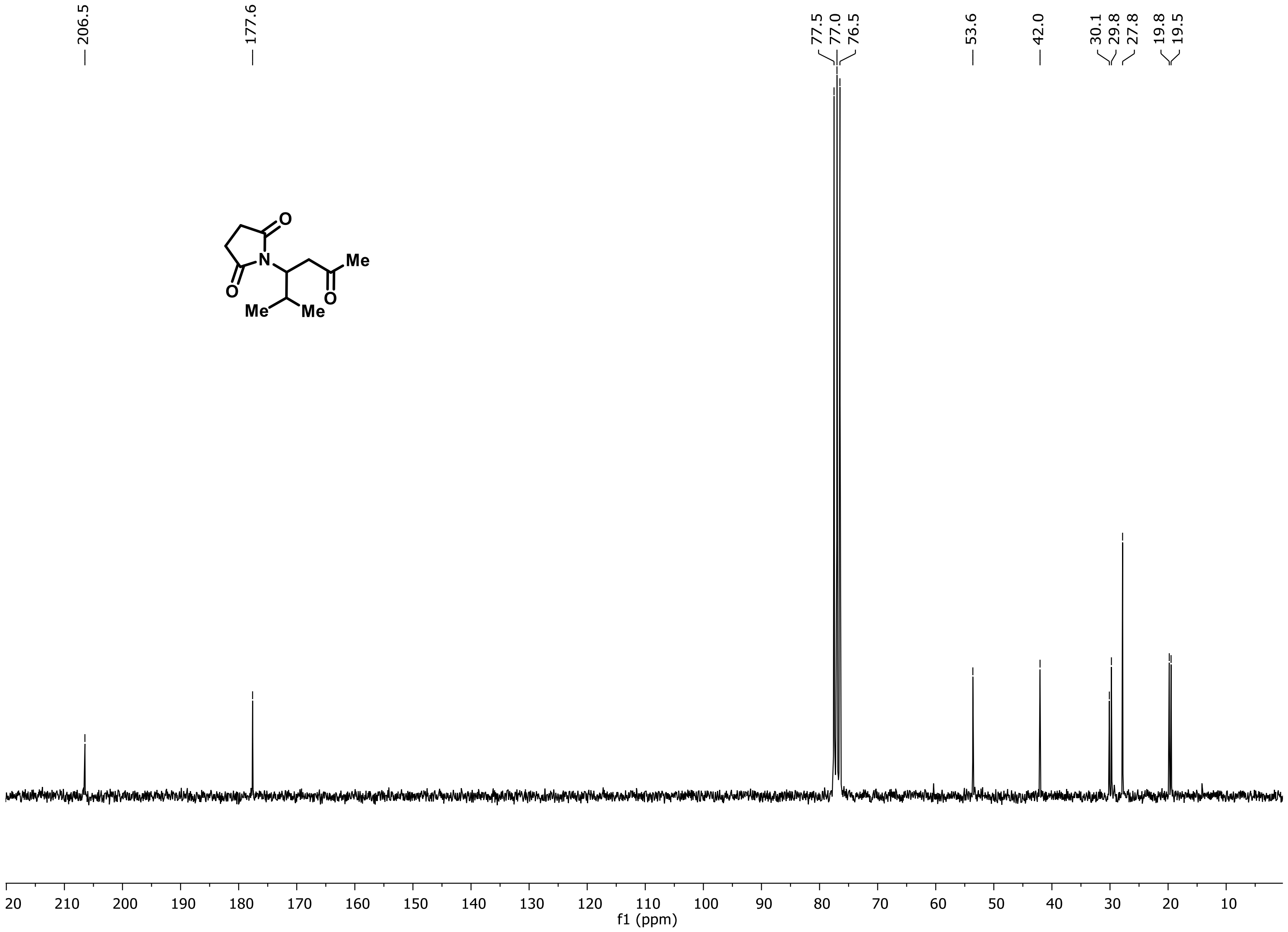

Molecule **4p**: ^{1}H NMR (250 MHz, $CDCl_3$)

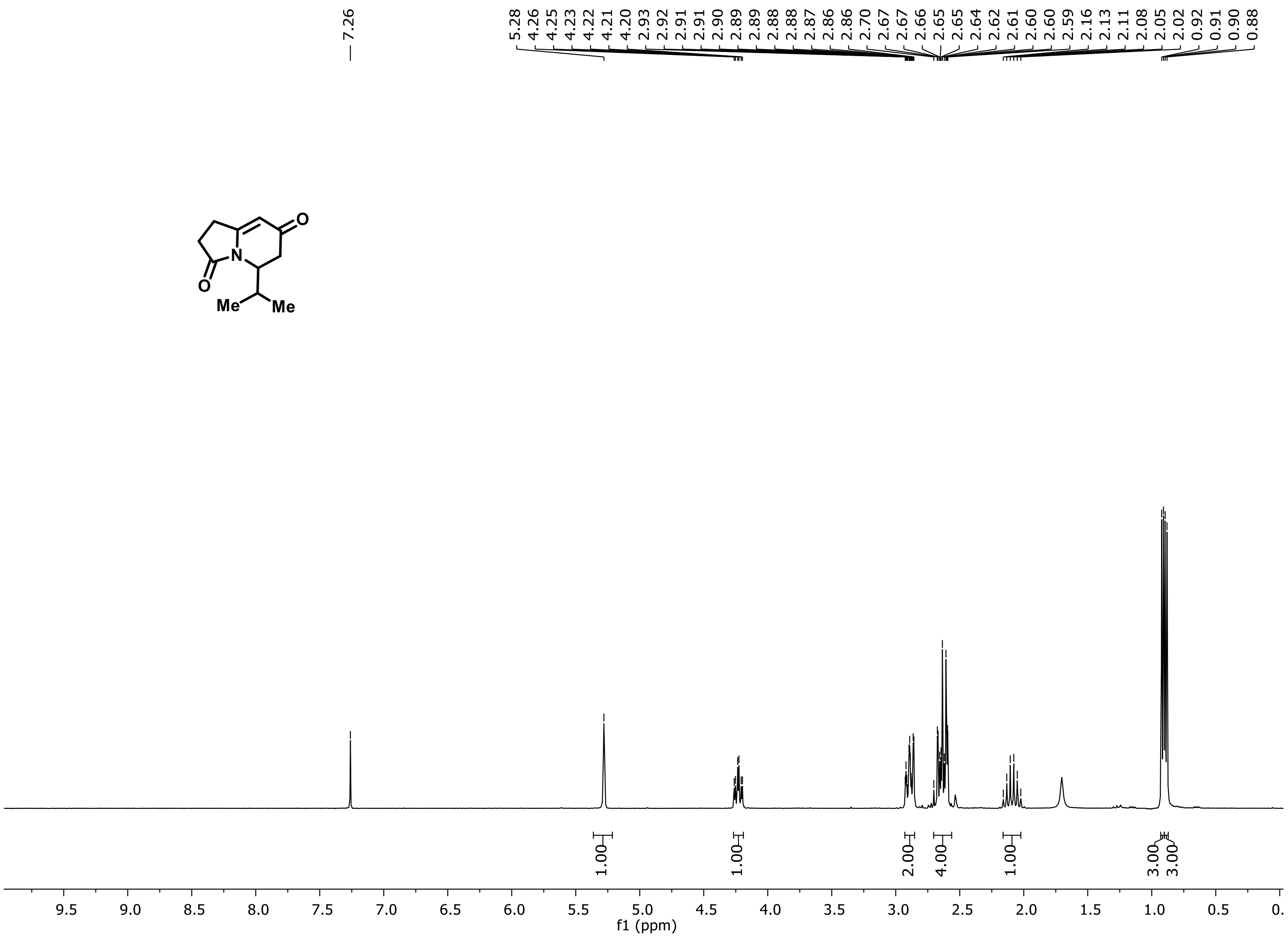

Molecule **4p**: $^{13}C\{^{1}H\}$ NMR (62.5 MHz, $CDCl_3$)

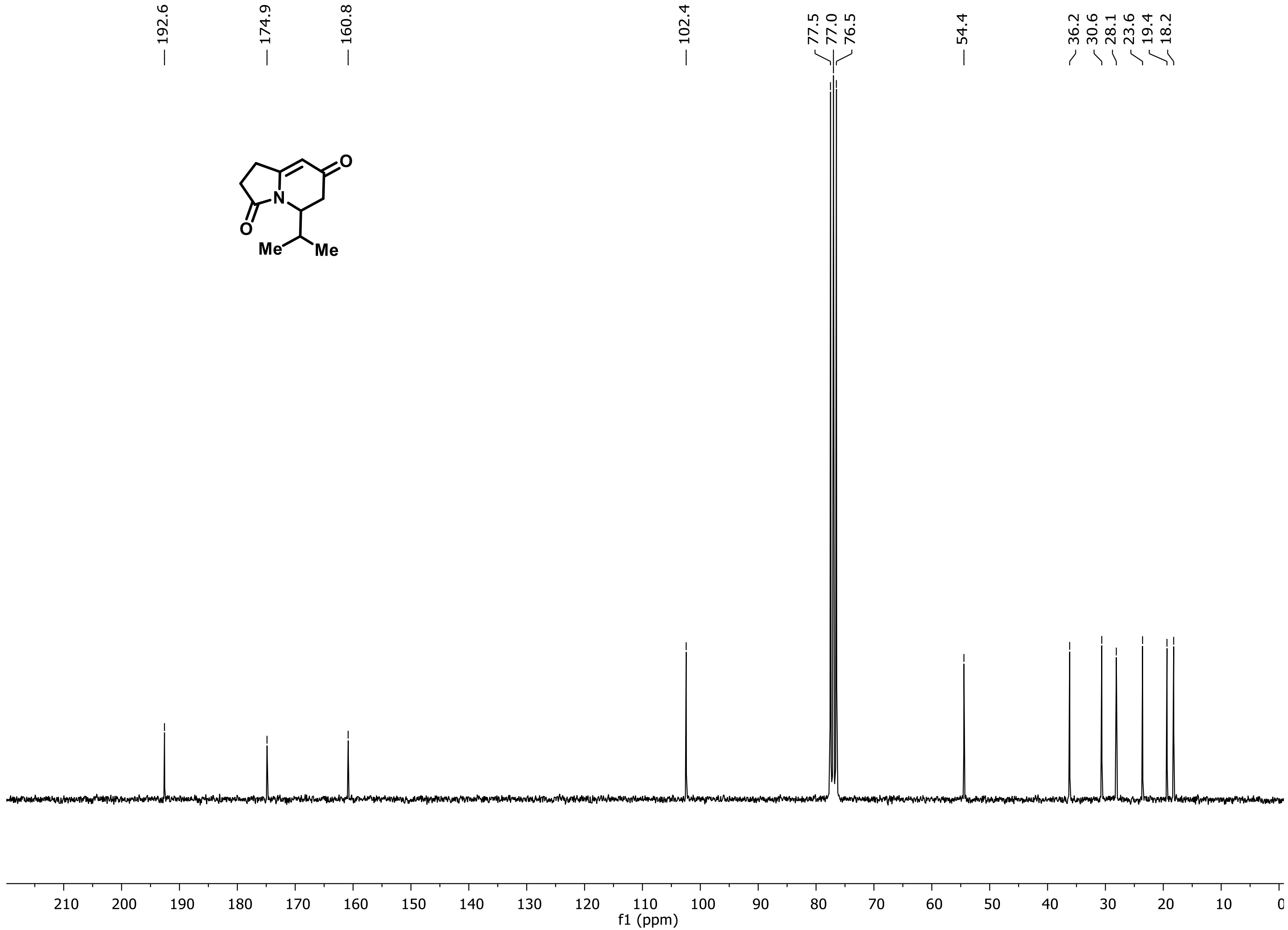

Molecule **3q**: ^{1}H NMR (250 MHz, $CDCl_3$)

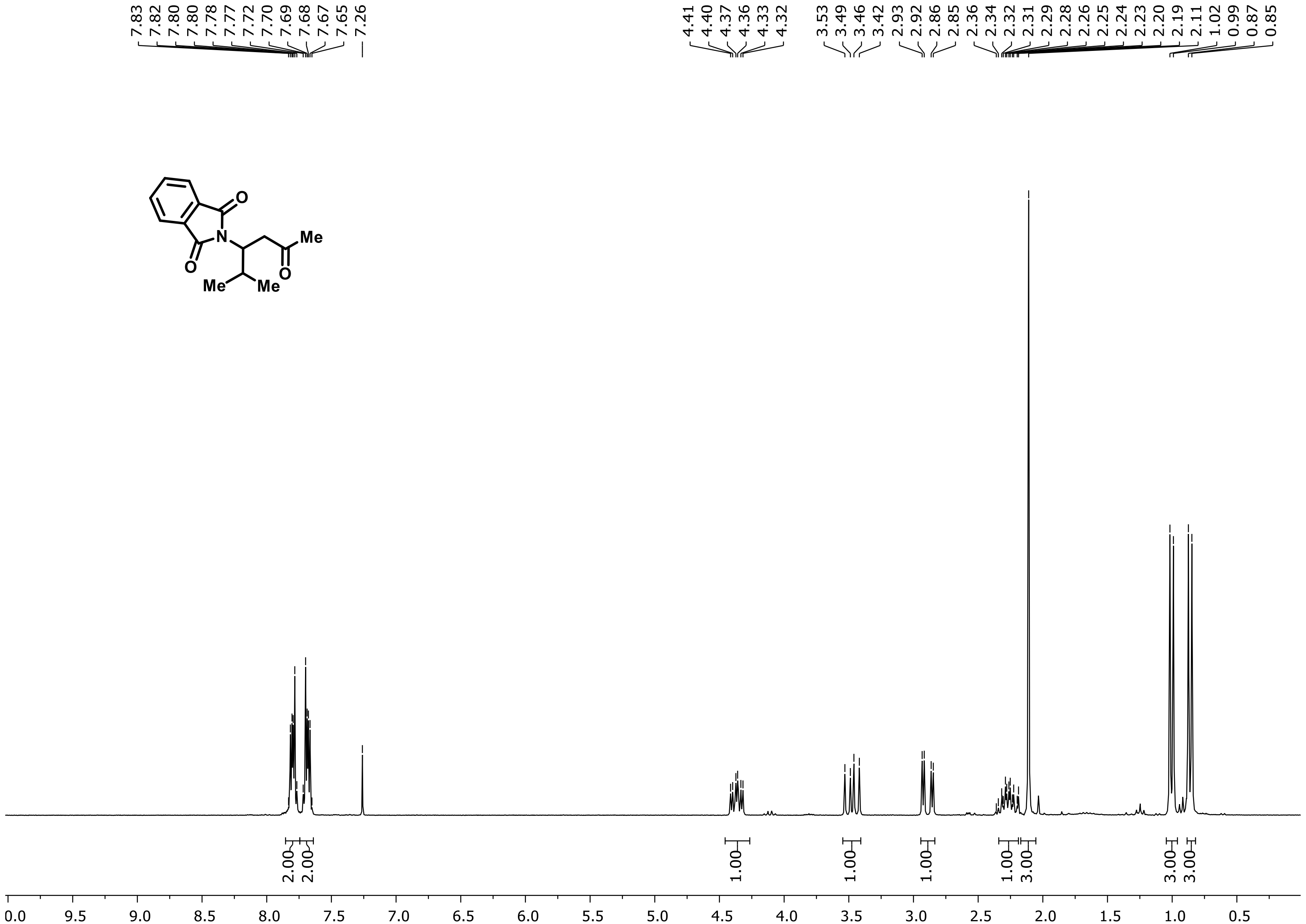

Molecule **3q**: $^{13}C\{^{1}H\}$ NMR (62.5 MHz, $CDCl_3$)

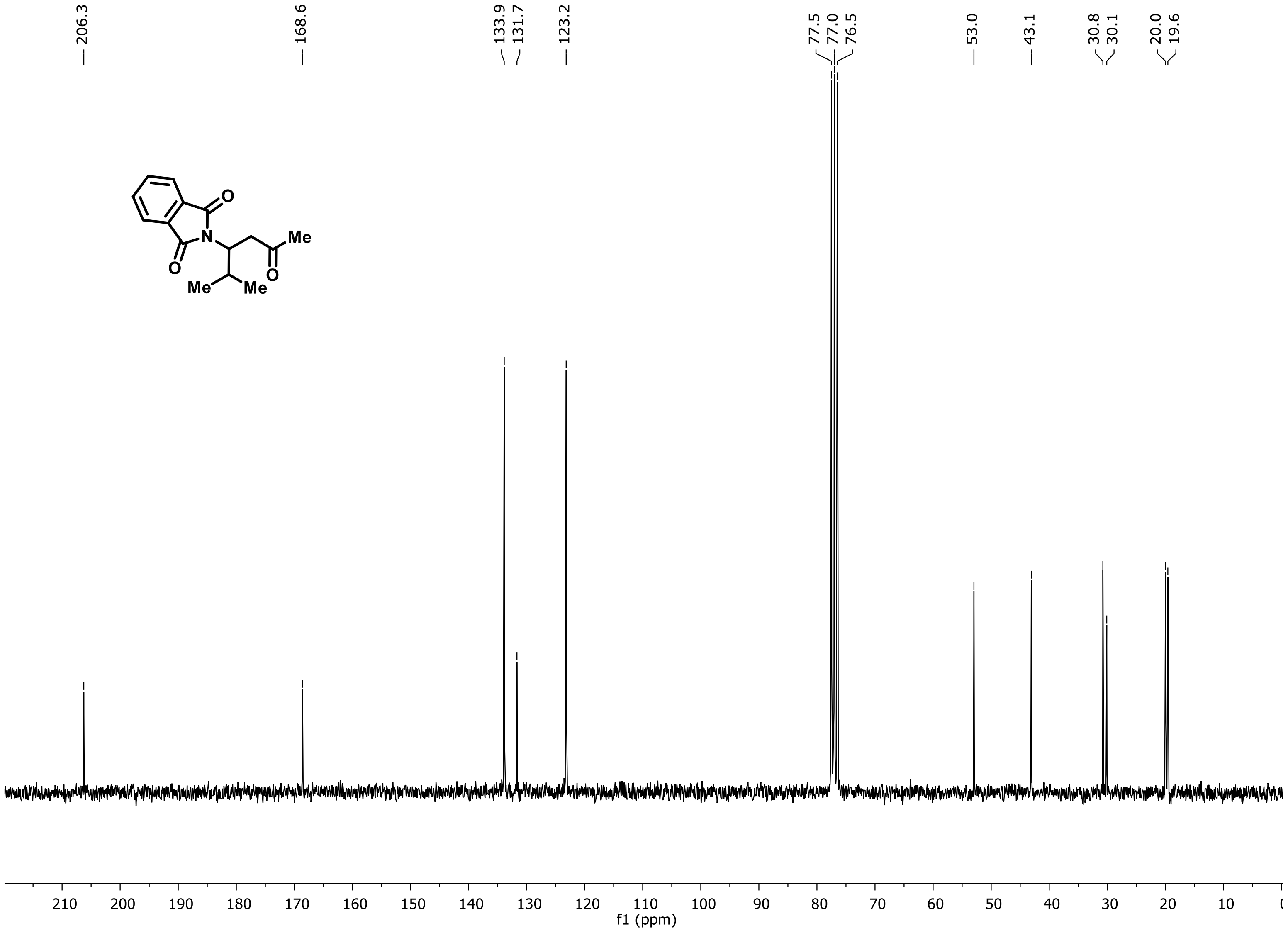

Molecule **4q**: ^{1}H NMR (250 MHz, $CDCl_3$)

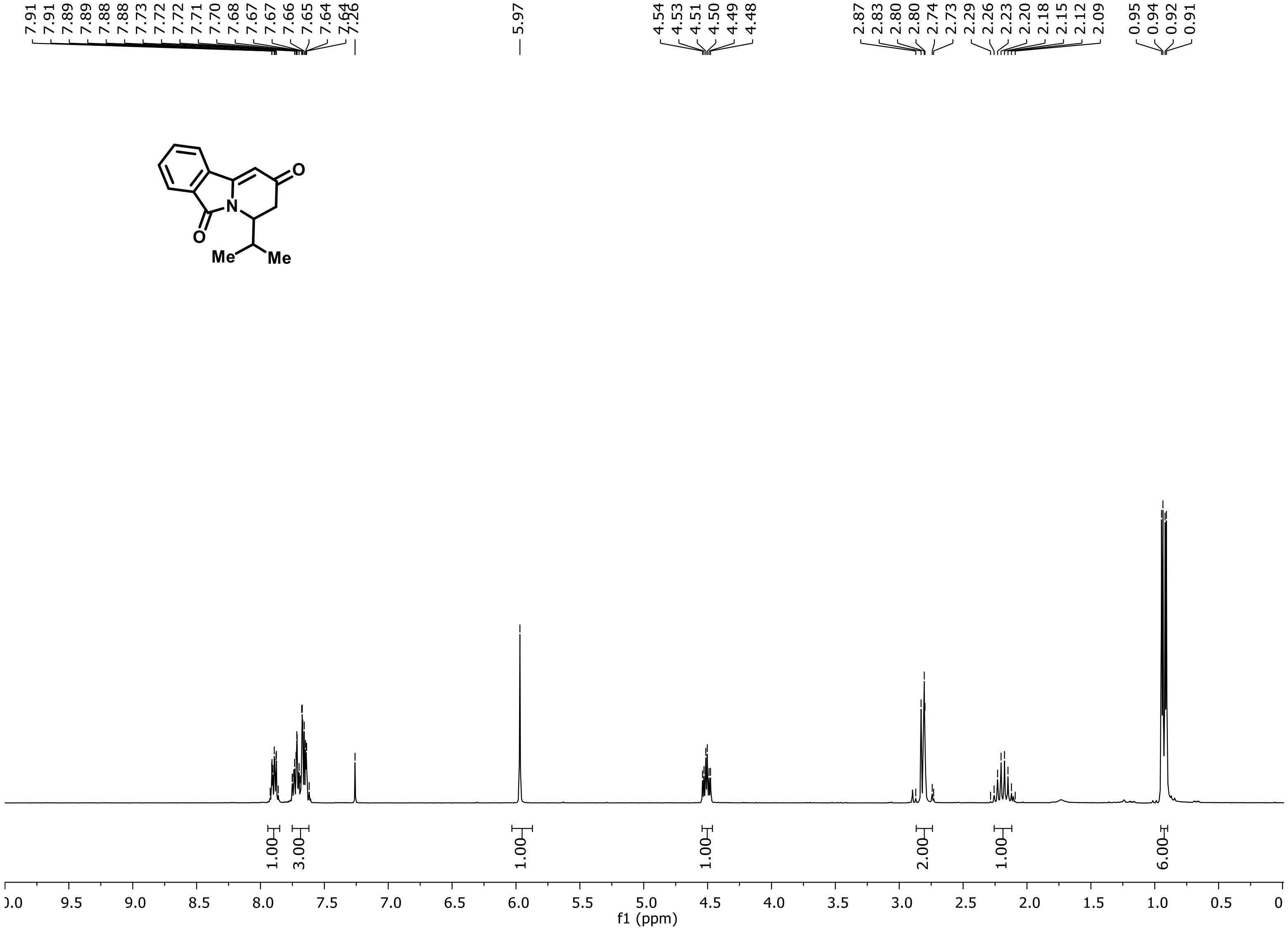

Molecule **4q**: $^{13}C\{^{1}H\}$ NMR (62.5 MHz, $CDCl_3$)

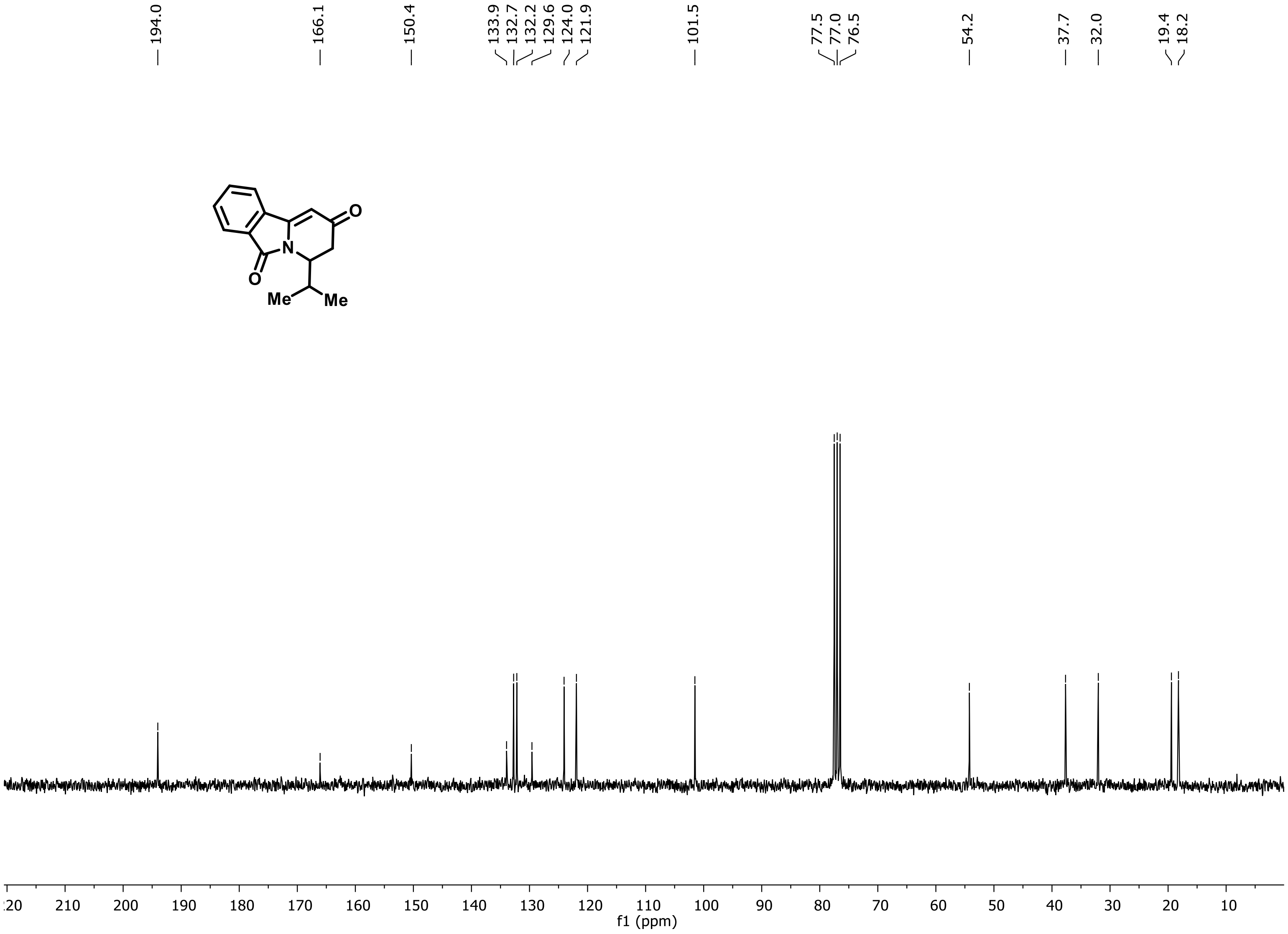

Molecule **3r**: ^{1}H NMR (250 MHz, $CDCl_3$)

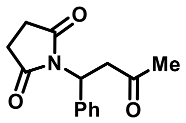

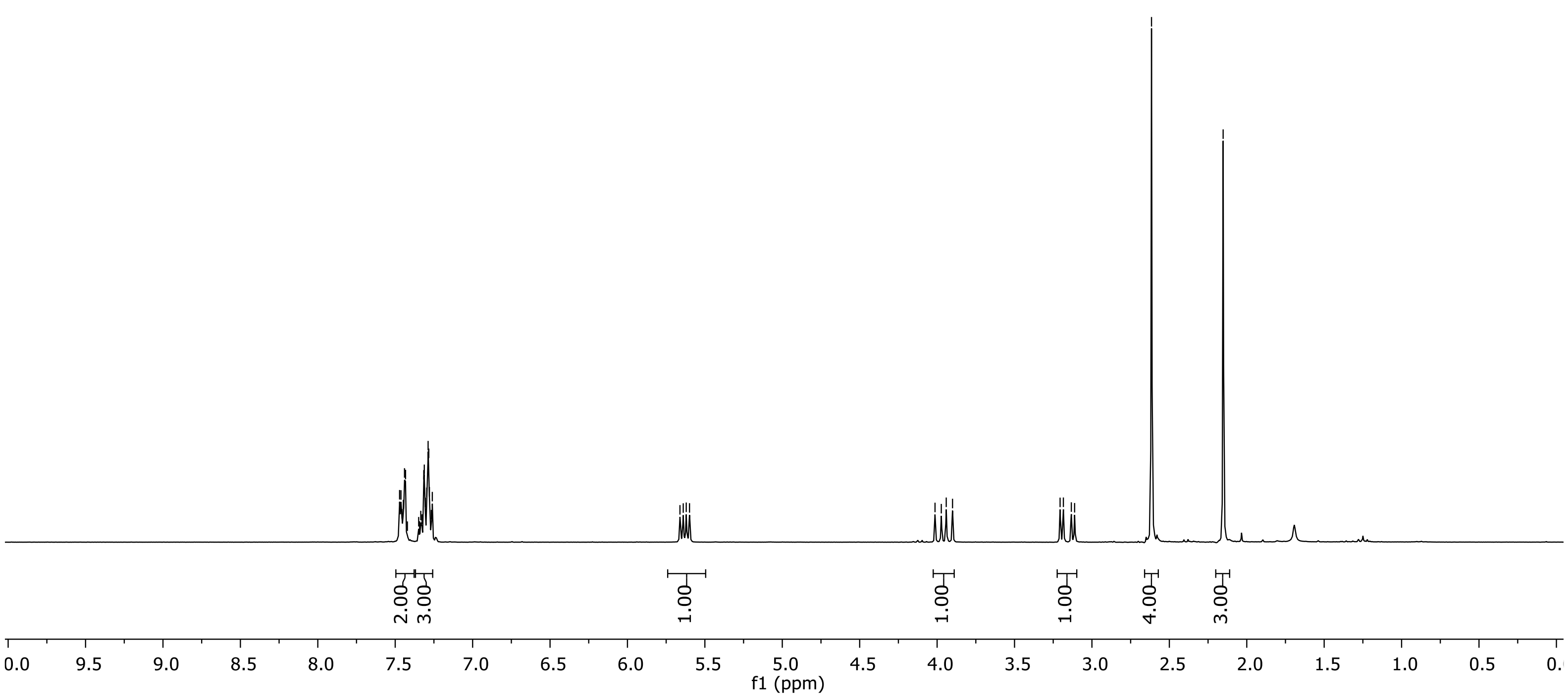

Molecule **3r**: $^{13}C\{^1H\}$ NMR (62.5 MHz, $CDCl_3$)

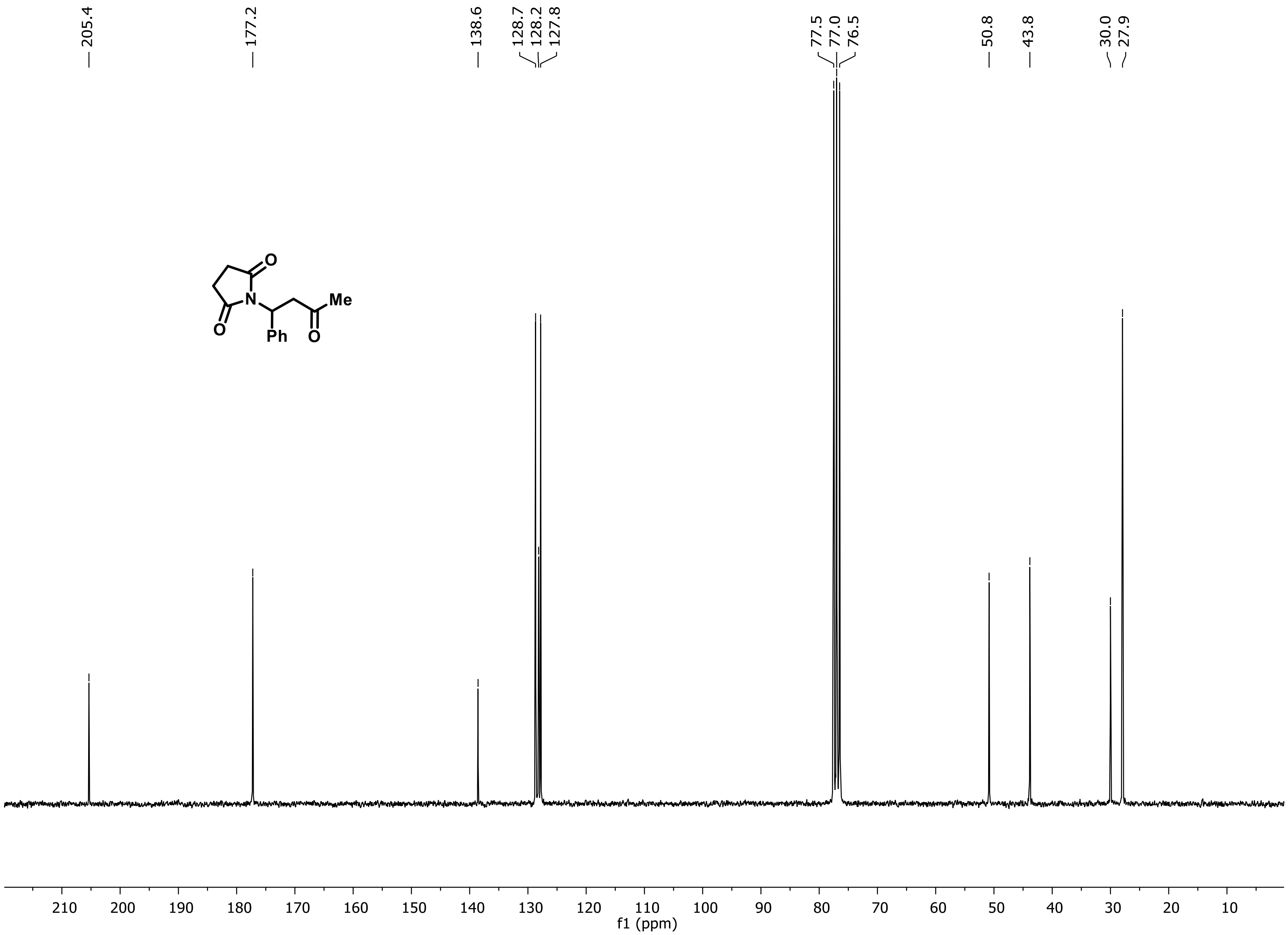

Molecule **5a**: ^{1}H NMR (250 MHz, $CDCl_3$)

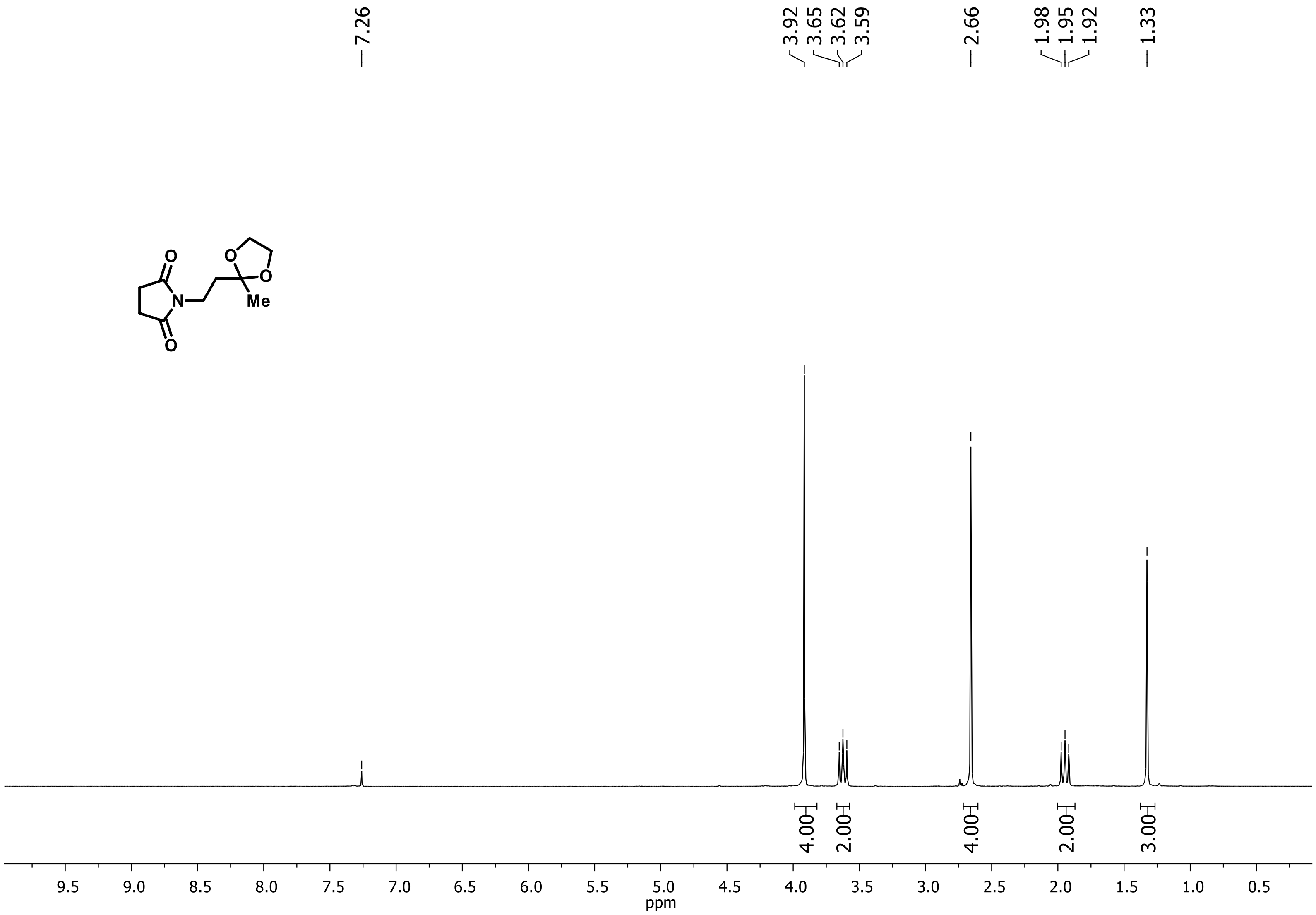

Molecule **5a**: $^{13}C\{^{1}H\}$ NMR (62.5 MHz, $CDCl_3$)

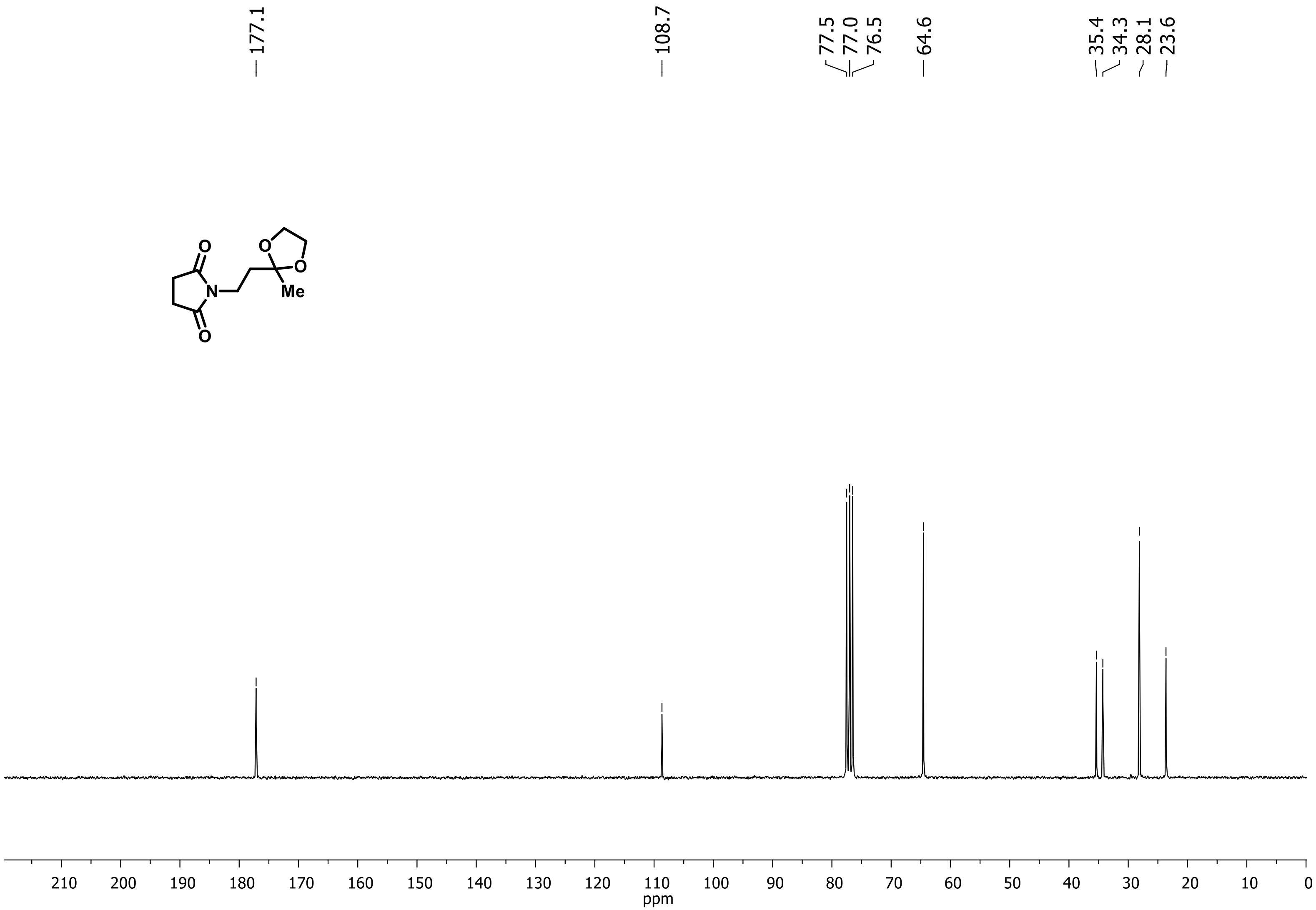

Conversion from ketal **5a** to bicycle **4a**: ^{1}H NMR crude with internal standard (250 MHz, $CDCl_3$):

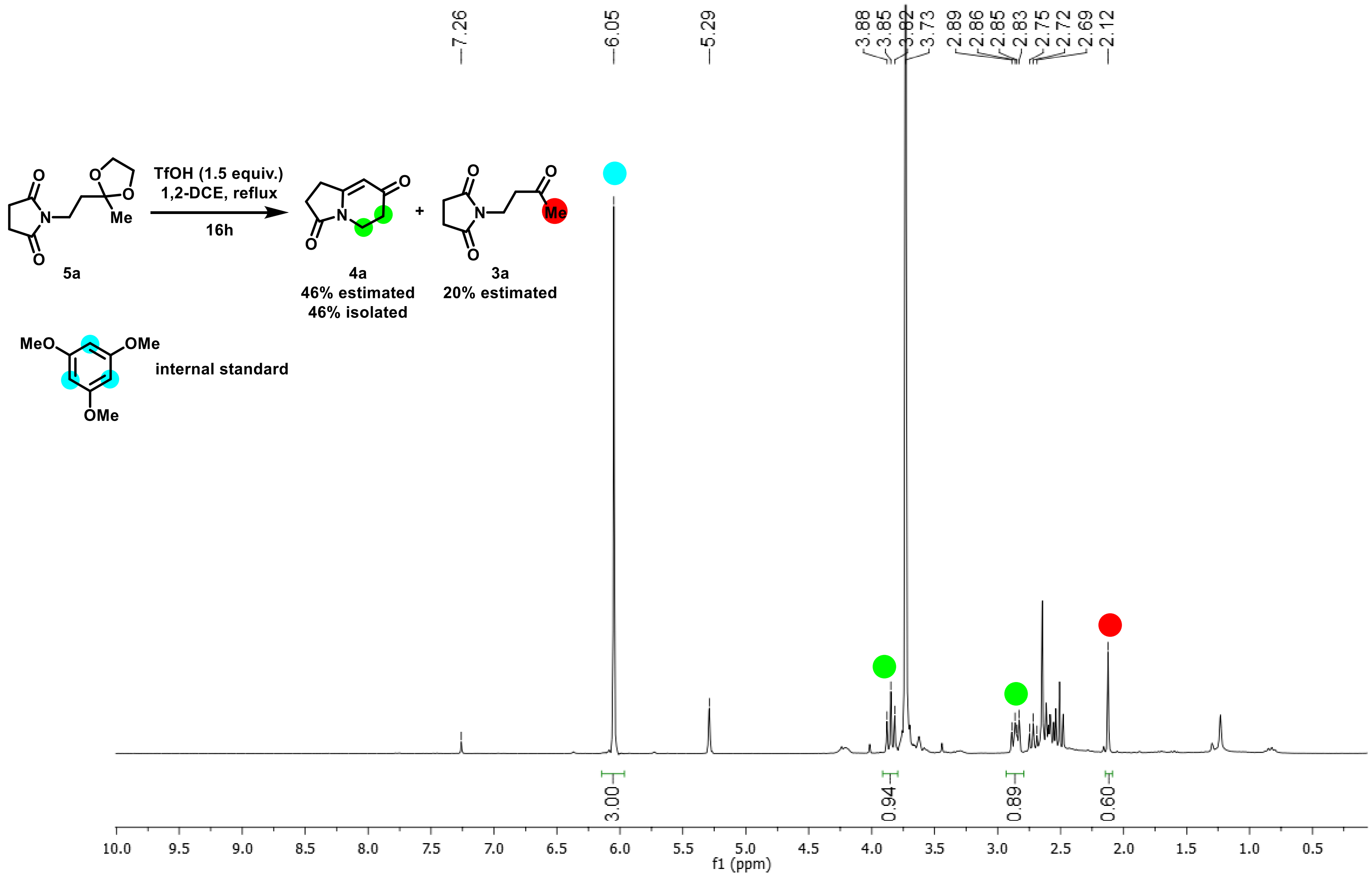

Molecule **5b**: ^{1}H NMR (250 MHz, $CDCl_3$)

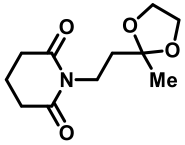

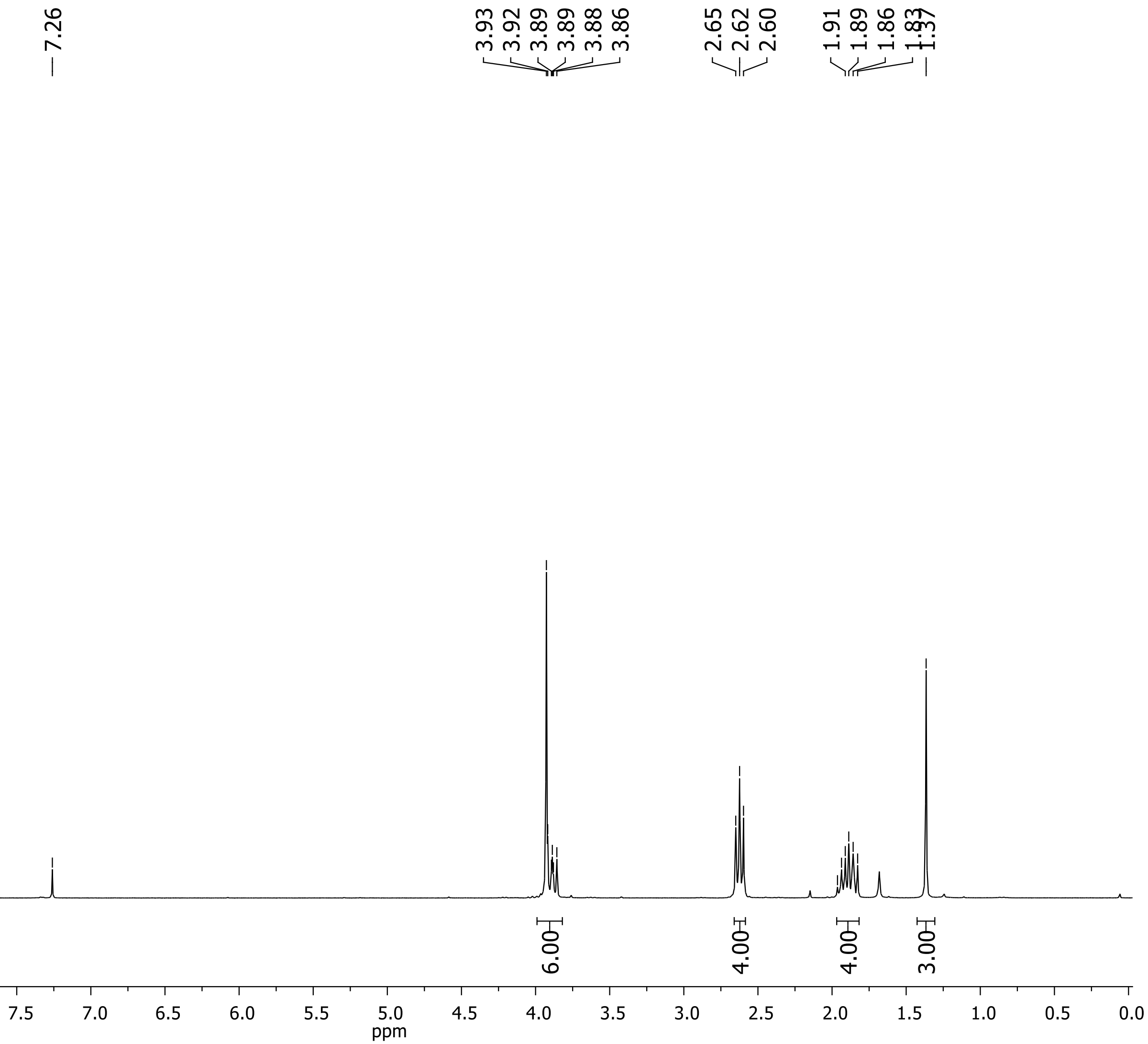

Molecule **5b**: $^{13}C\{^{1}H\}$ NMR (62.5 MHz, $CDCl_3$)

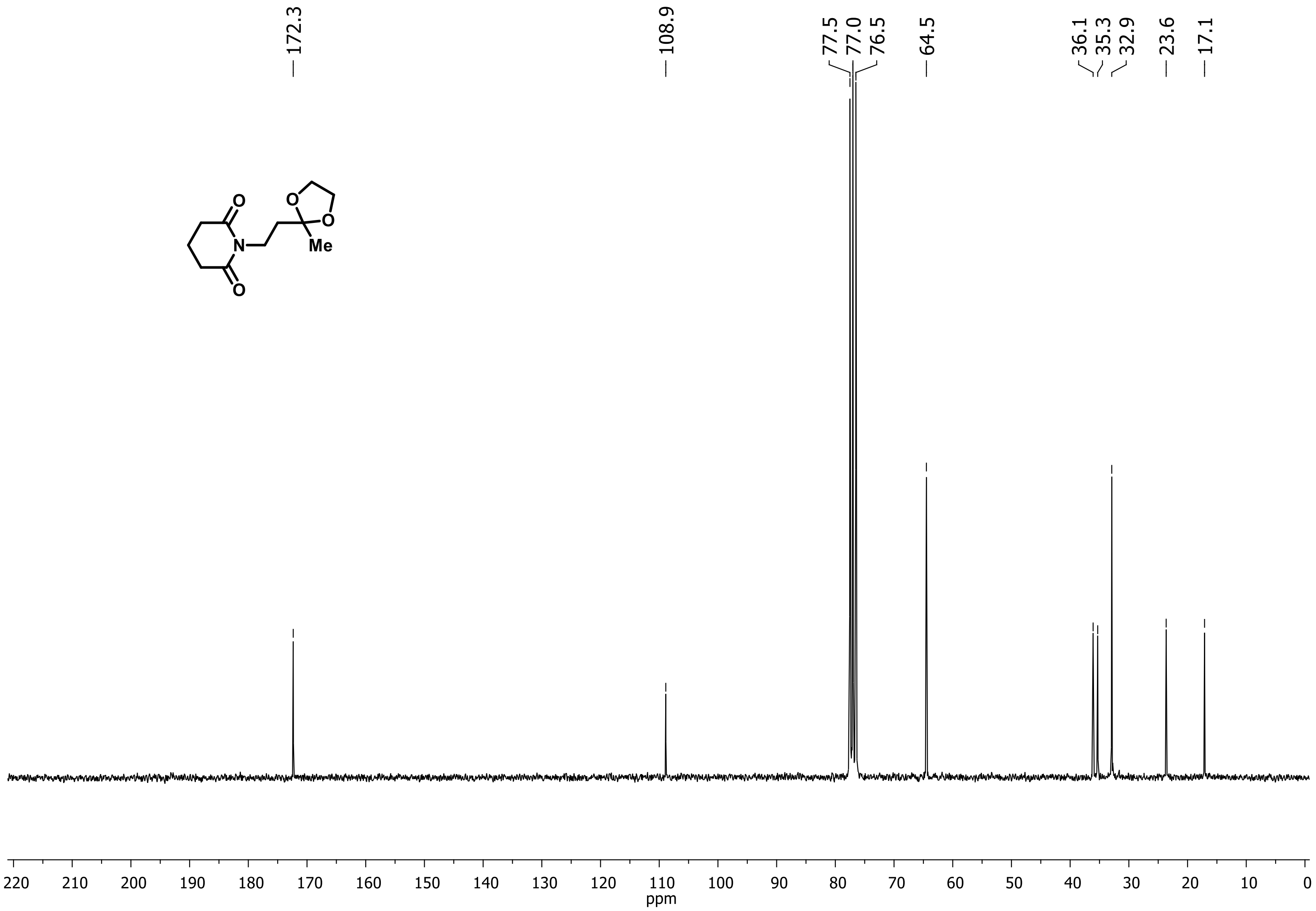

Conversion from ketal **5b** to bicycle **4b**: ^{1}H NMR crude with internal standard (250 MHz, $CDCl_3$):

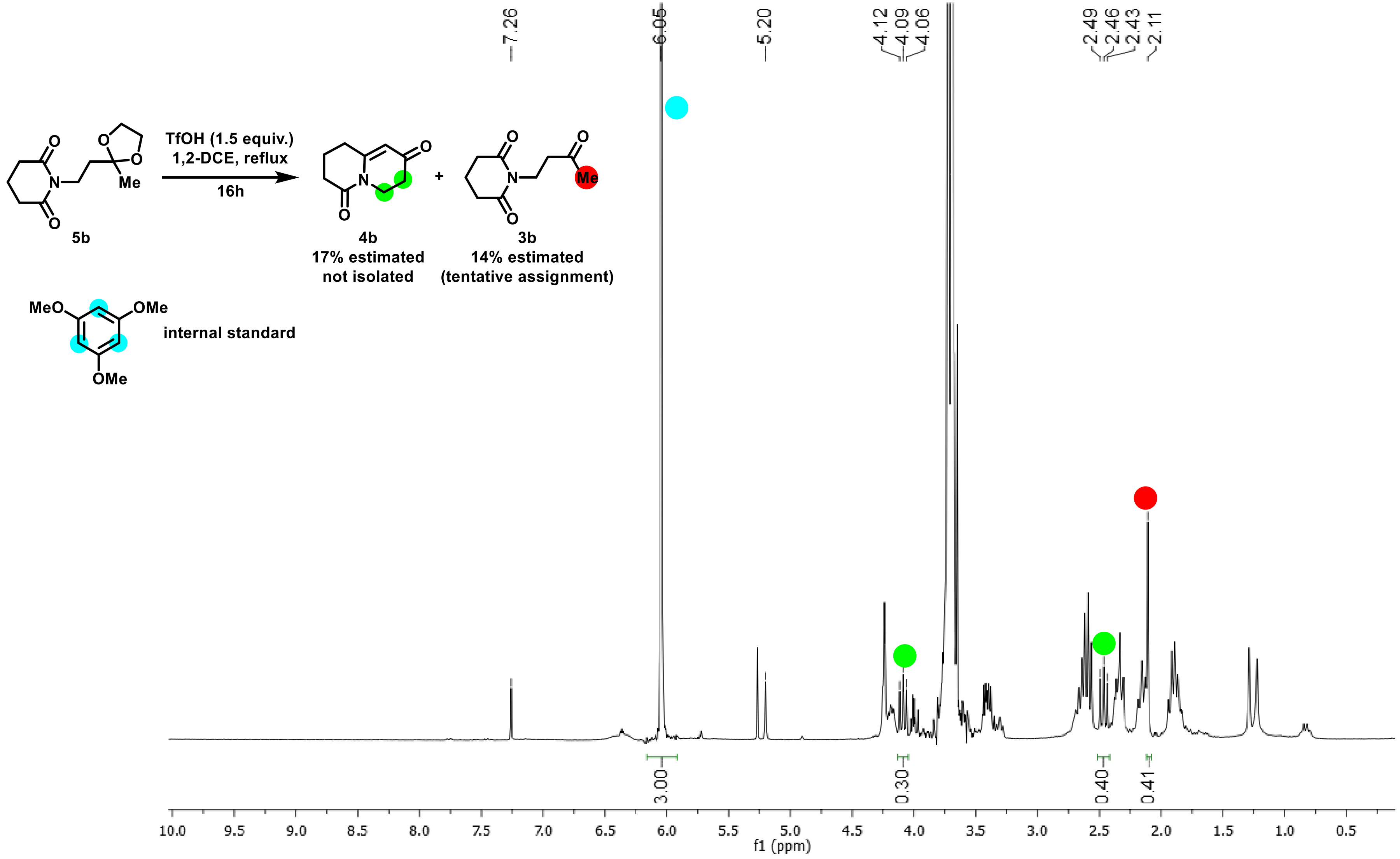

Molecule 7a: ^{1}H NMR (250 MHz, $CDCl_3$)

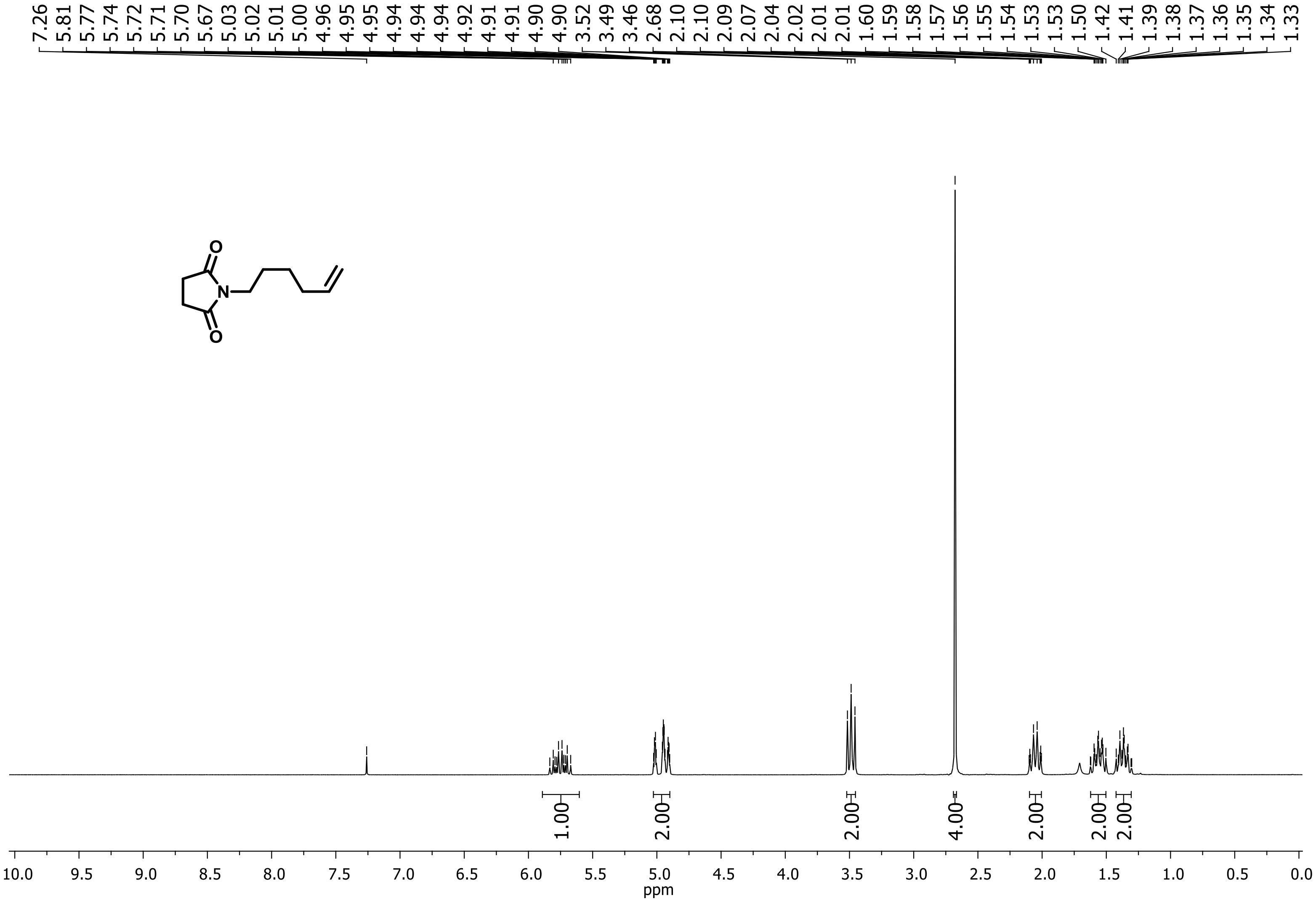

Molecule 7a: $^{13}C\{^1H\}$ NMR (62.5 MHz, $CDCl_3$)

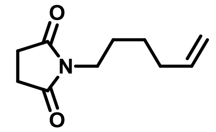

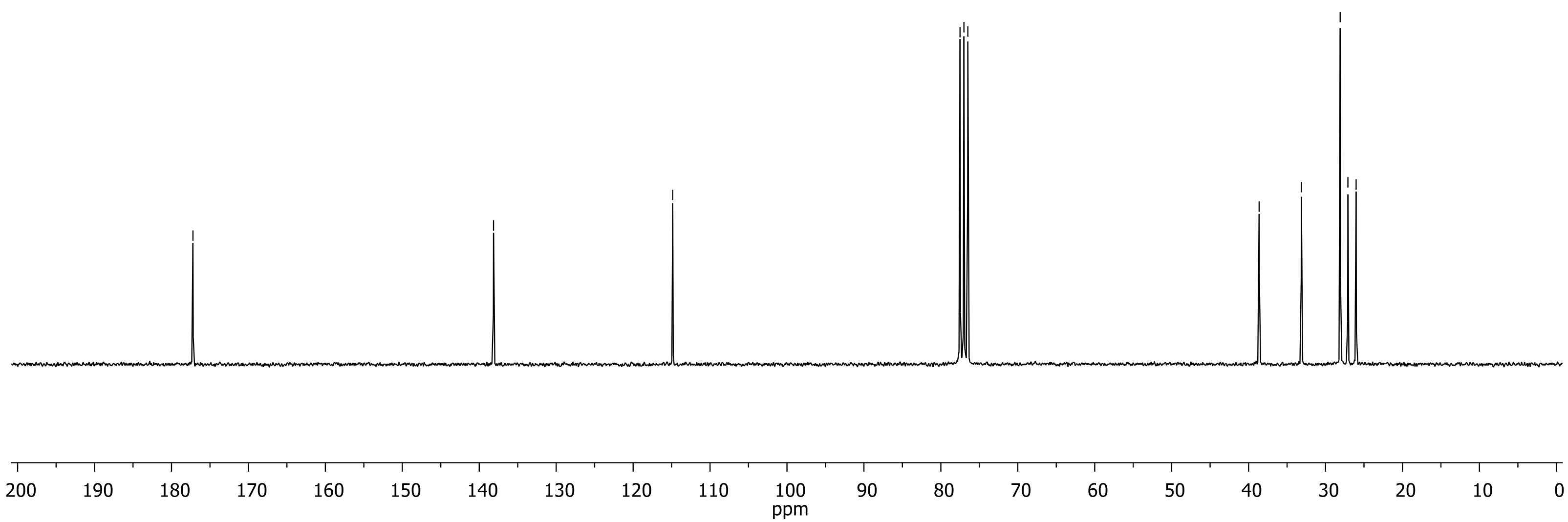

Molecule 8a: ^{1}H NMR (250 MHz, $CDCl_3$)

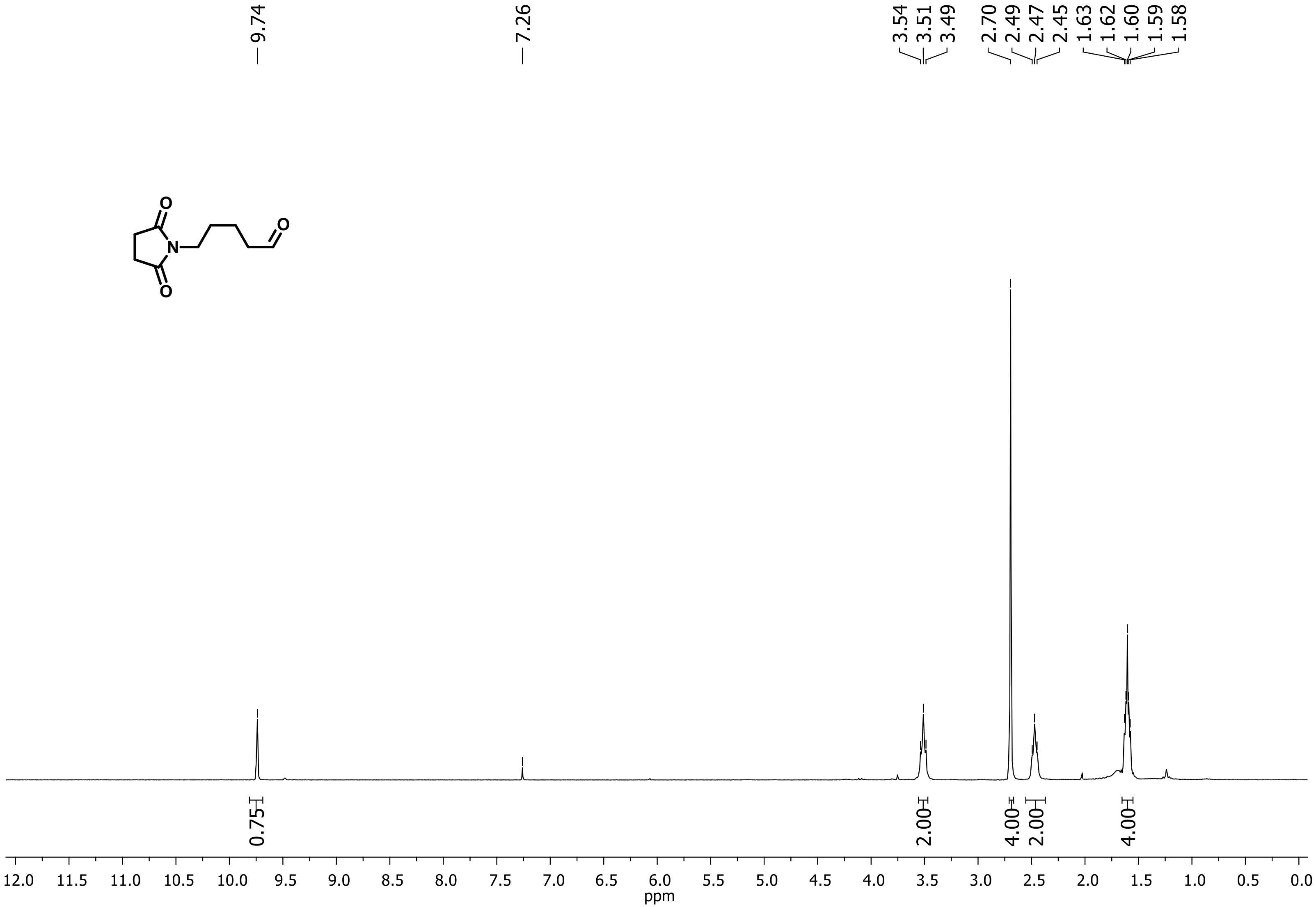

Molecule 8a: $^{13}C\{^{1}H\}$ NMR (62.5 MHz, $CDCl_3$)

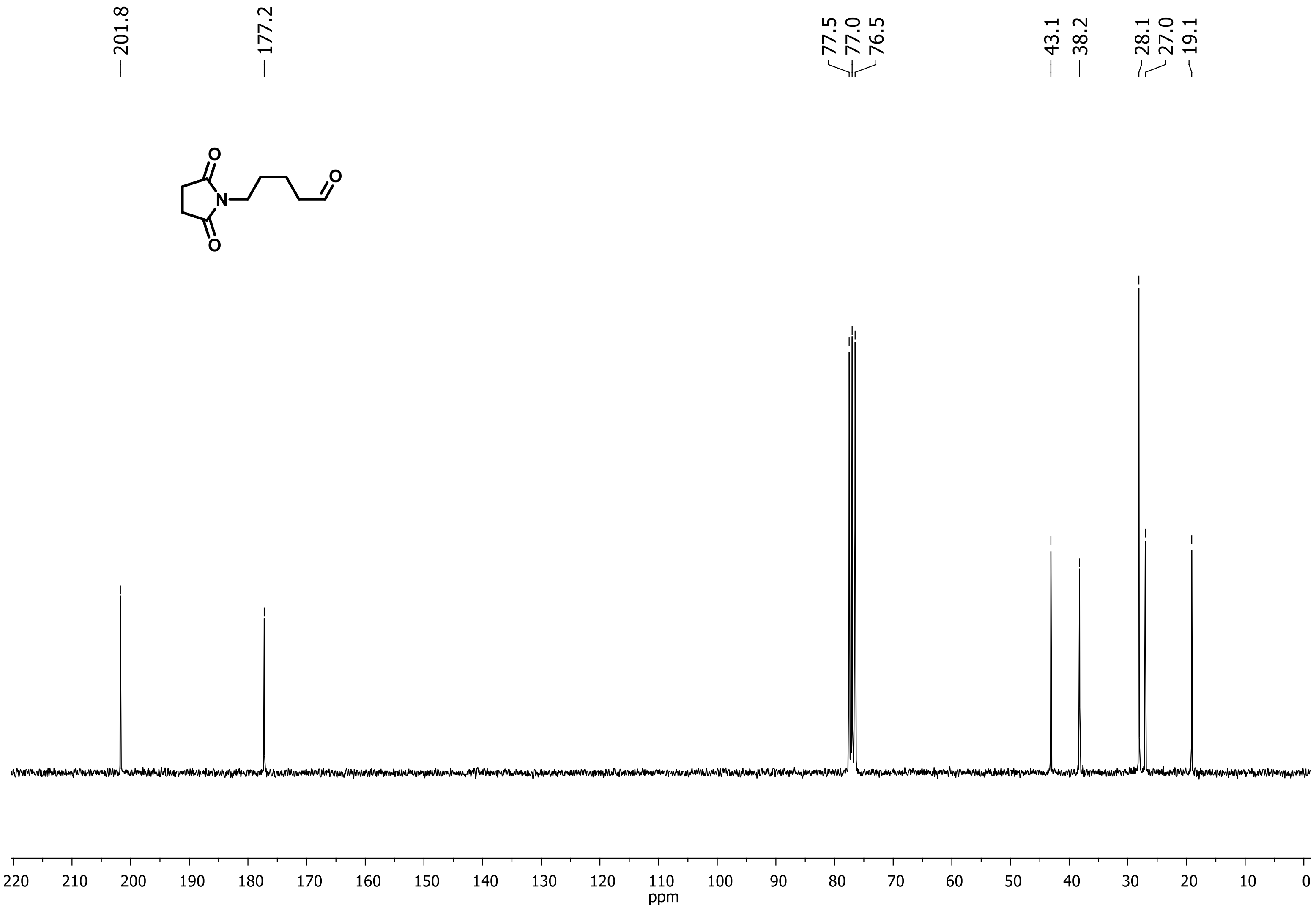

Molecule 9a: ^{1}H NMR (250 MHz, $CDCl_3$)

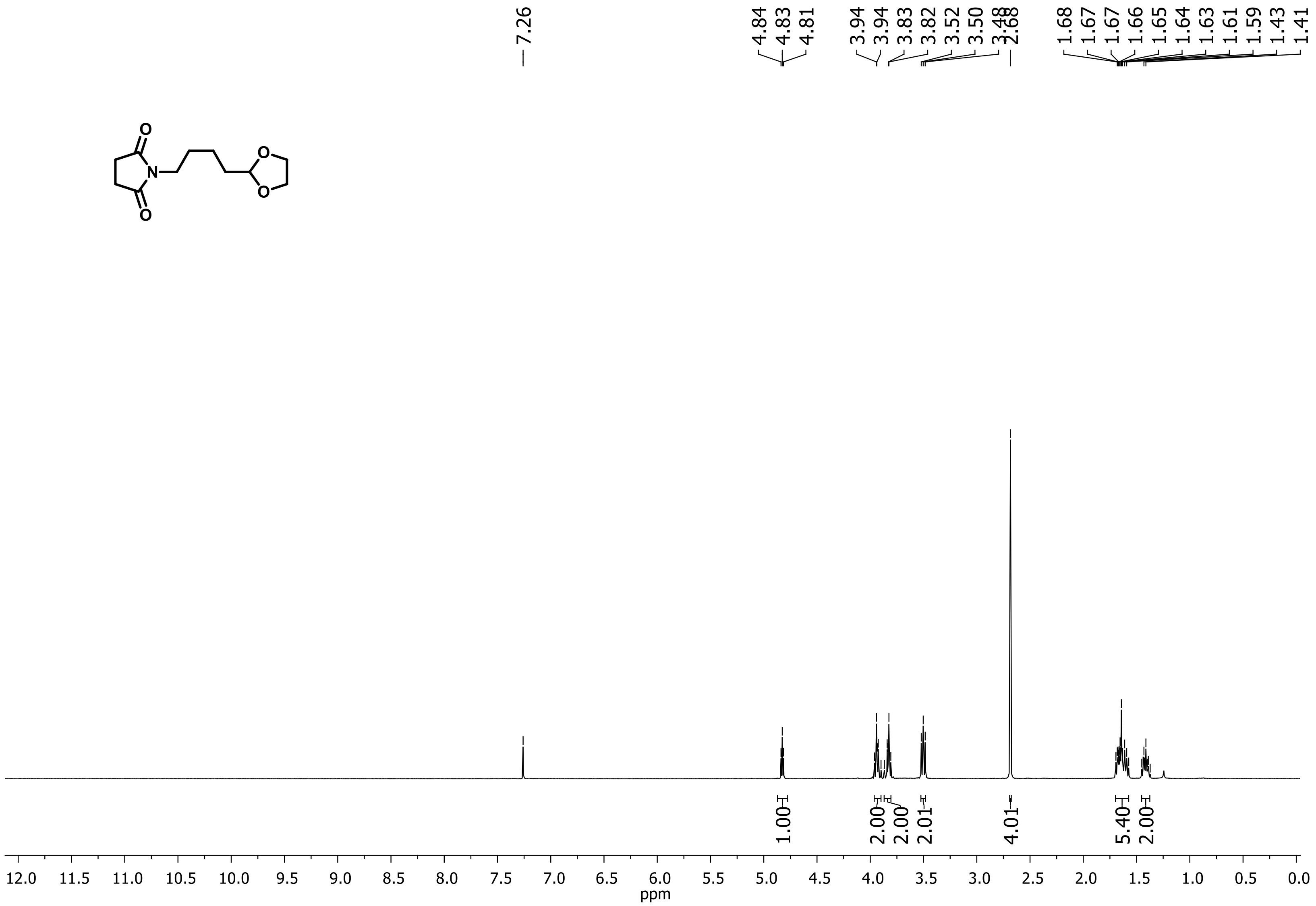

Molecule 9a: $^{13}C\{^{1}H\}$ NMR (62.5 MHz, $CDCl_3$)

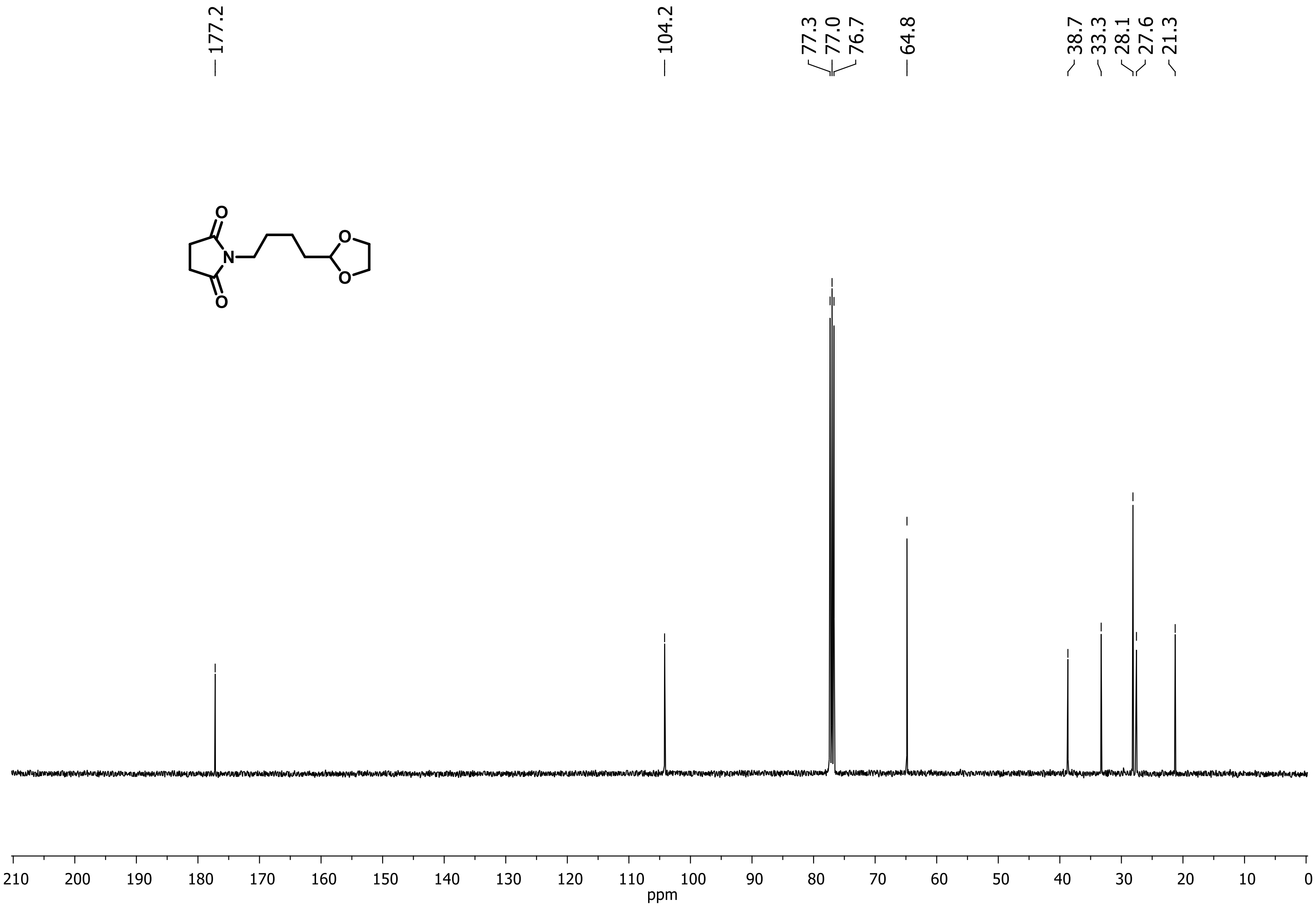

Molecule 7b: ^{1}H NMR (250 MHz, $CDCl_3$)

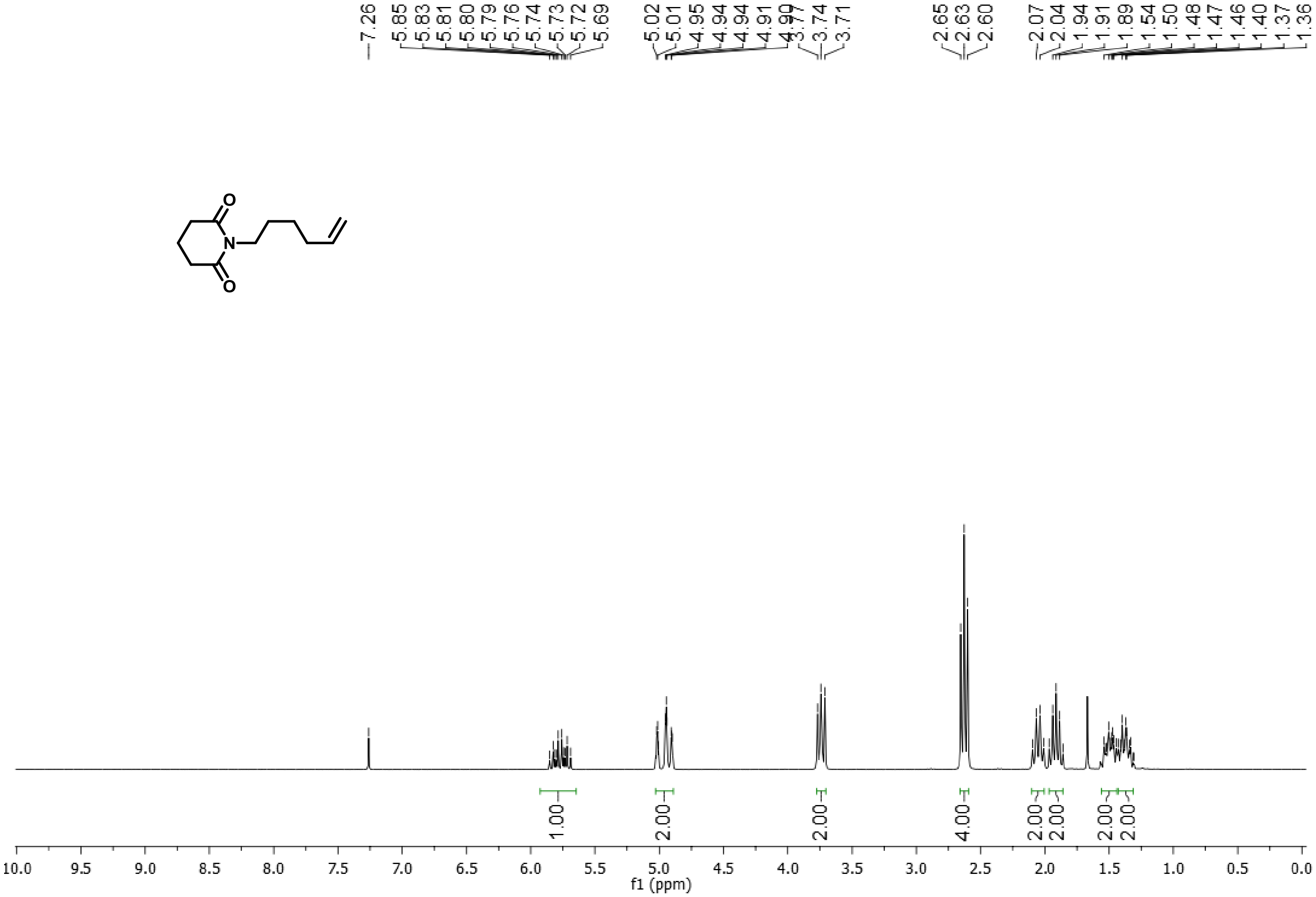

Molecule 7b: $^{13}C\{^1H\}$ NMR (62.5 MHz, $CDCl_3$)

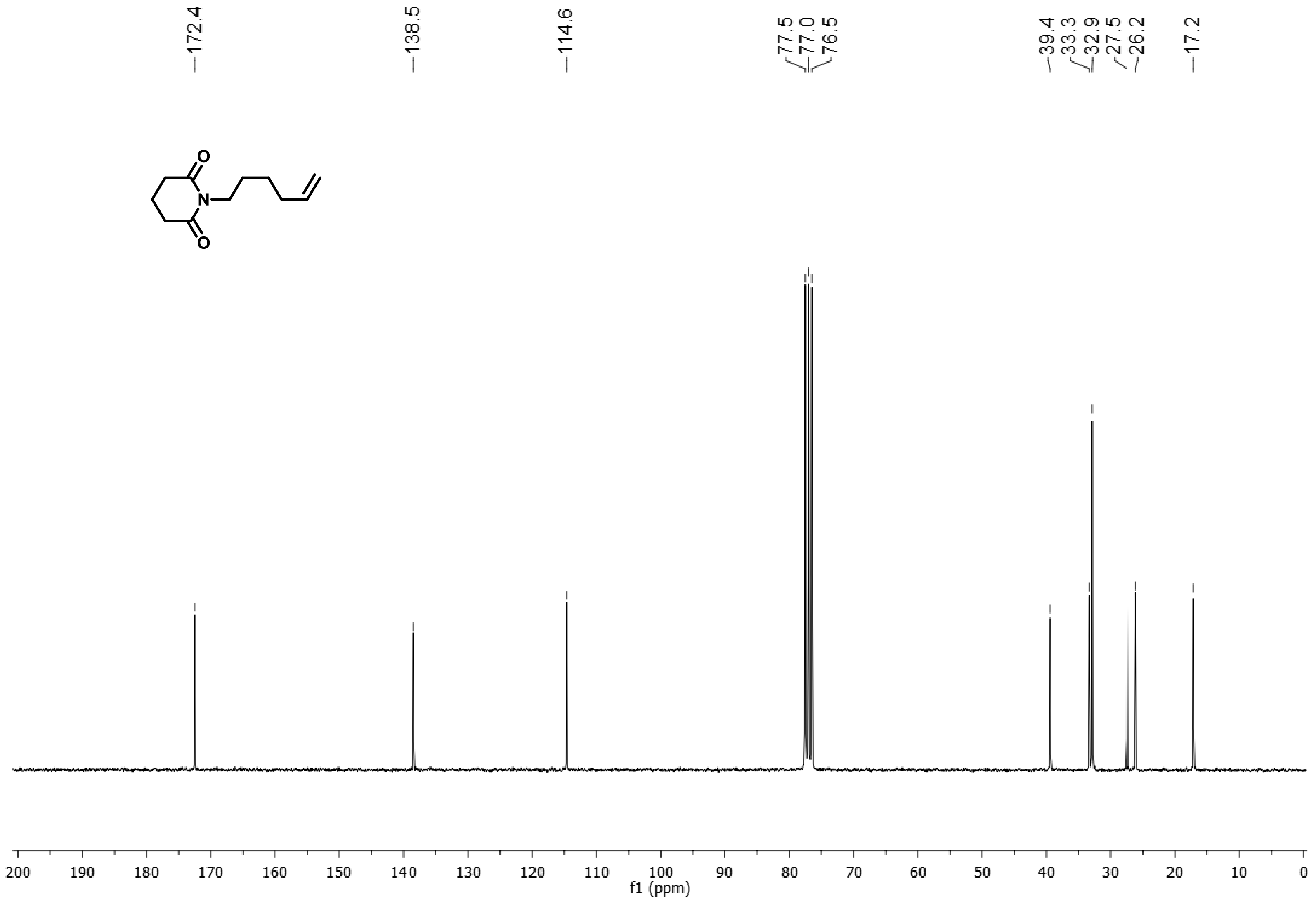

Molecule 8b: ^{1}H NMR (250 MHz, $CDCl_3$)

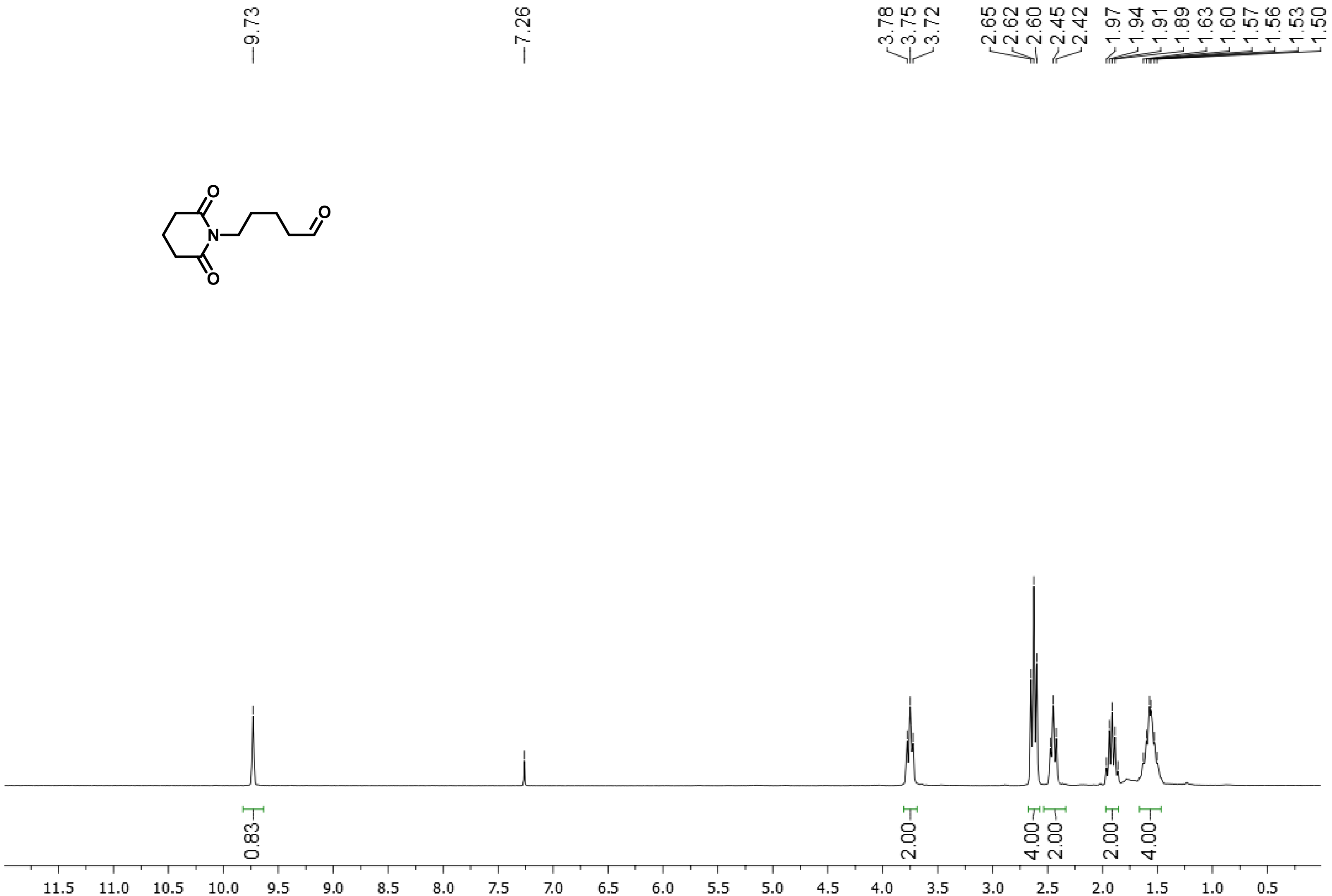

Molecule 8b: $^{13}C\{^{1}H\}$ NMR (62.5 MHz, $CDCl_3$)

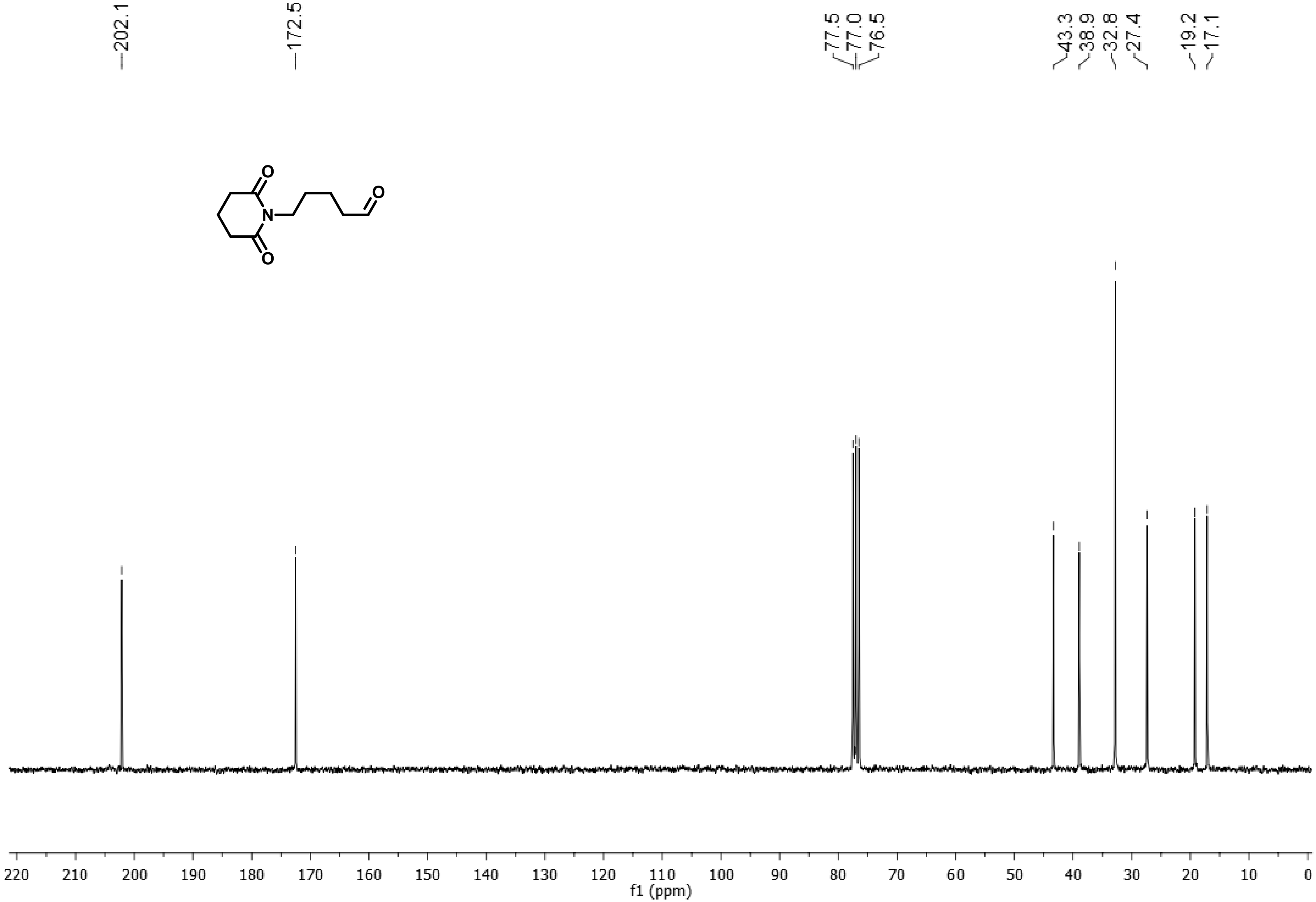

Molecule 9b: ^{1}H NMR (250 MHz, $CDCl_3$)

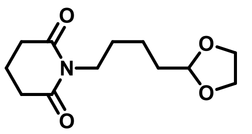

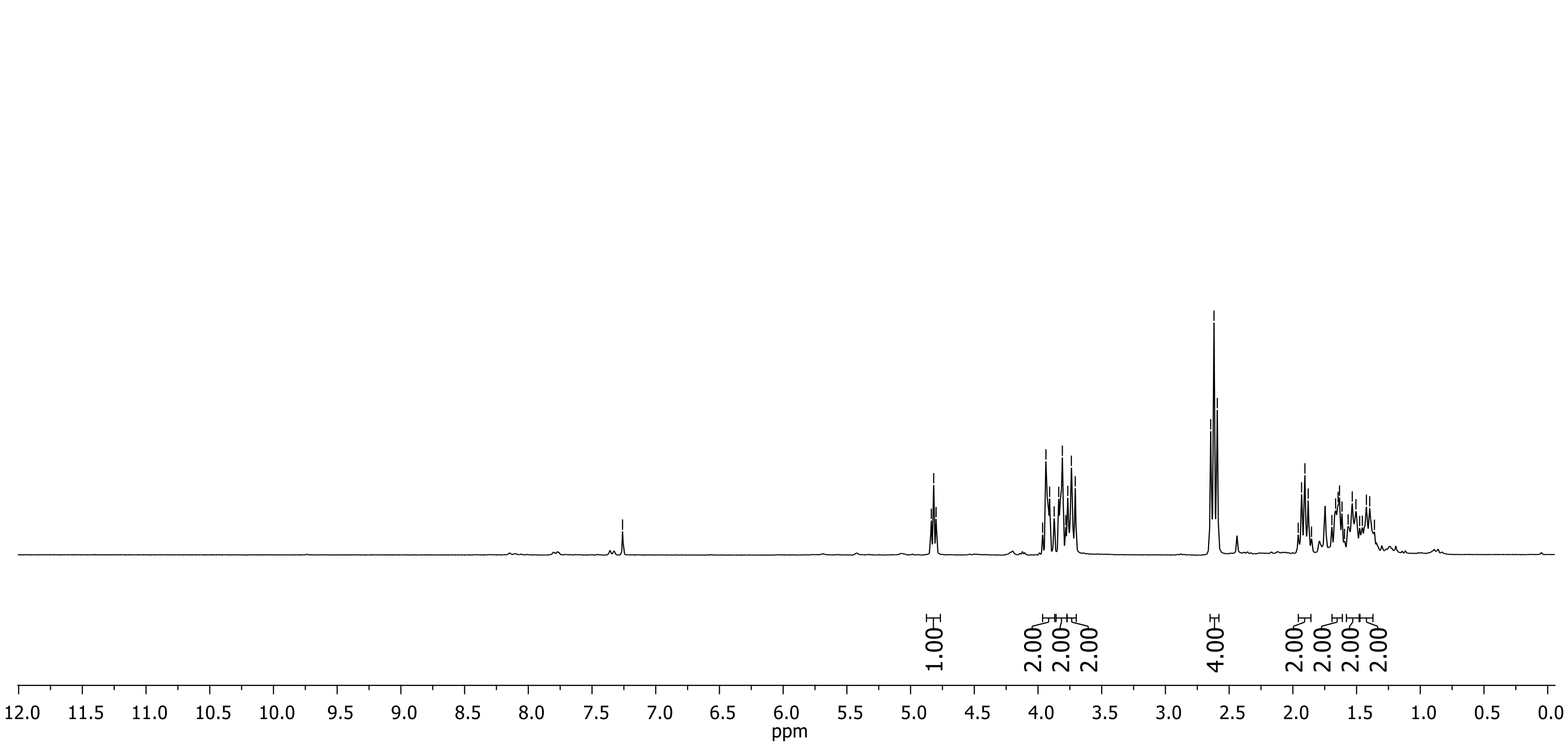

Molecule 9b: $^{13}C\{^{1}H\}$ NMR (62.5 MHz, $CDCl_3$)

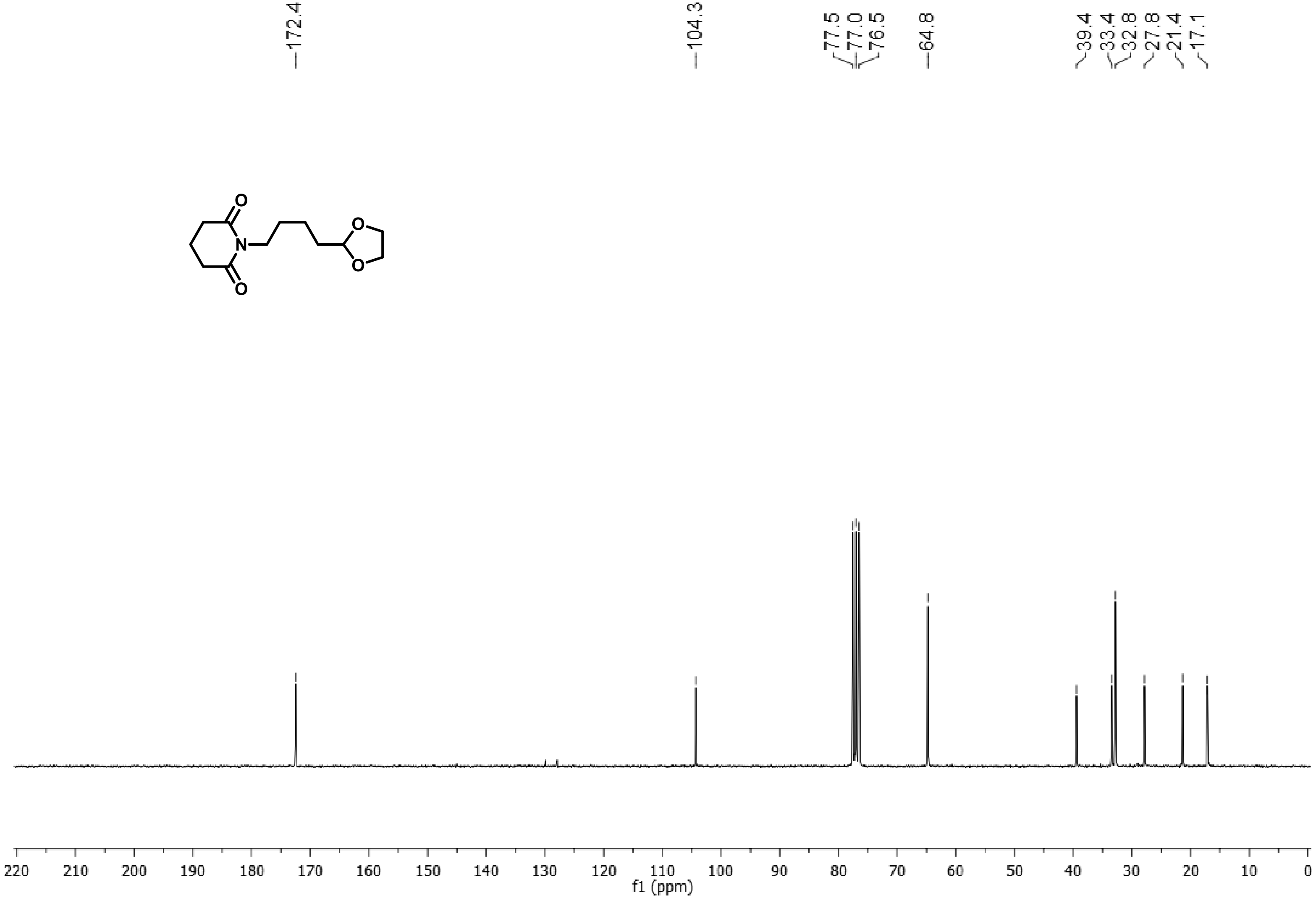

Molecule **12a**: ^{1}H NMR (250 MHz, $CDCl_3$)

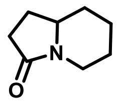

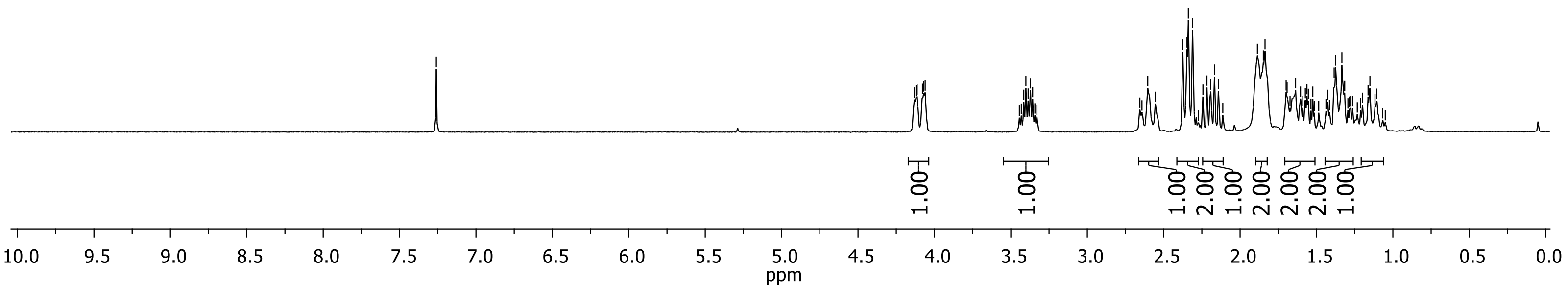

Molecule **12a**: $^{13}C\{^{1}H\}$ NMR (62.5 MHz, $CDCl_3$)

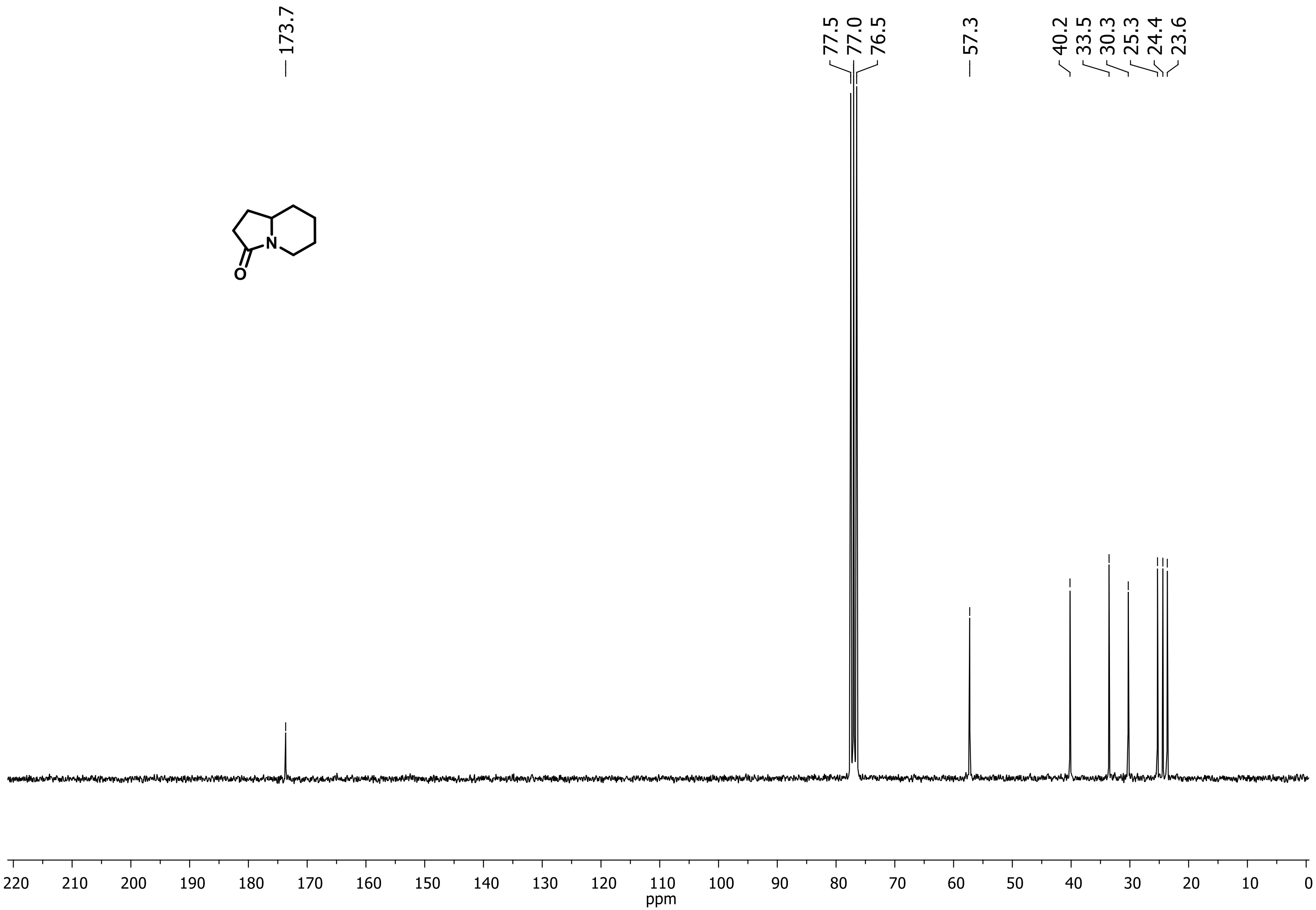

Molecule **13a**: ^{1}H NMR (250 MHz, $CDCl_3$)

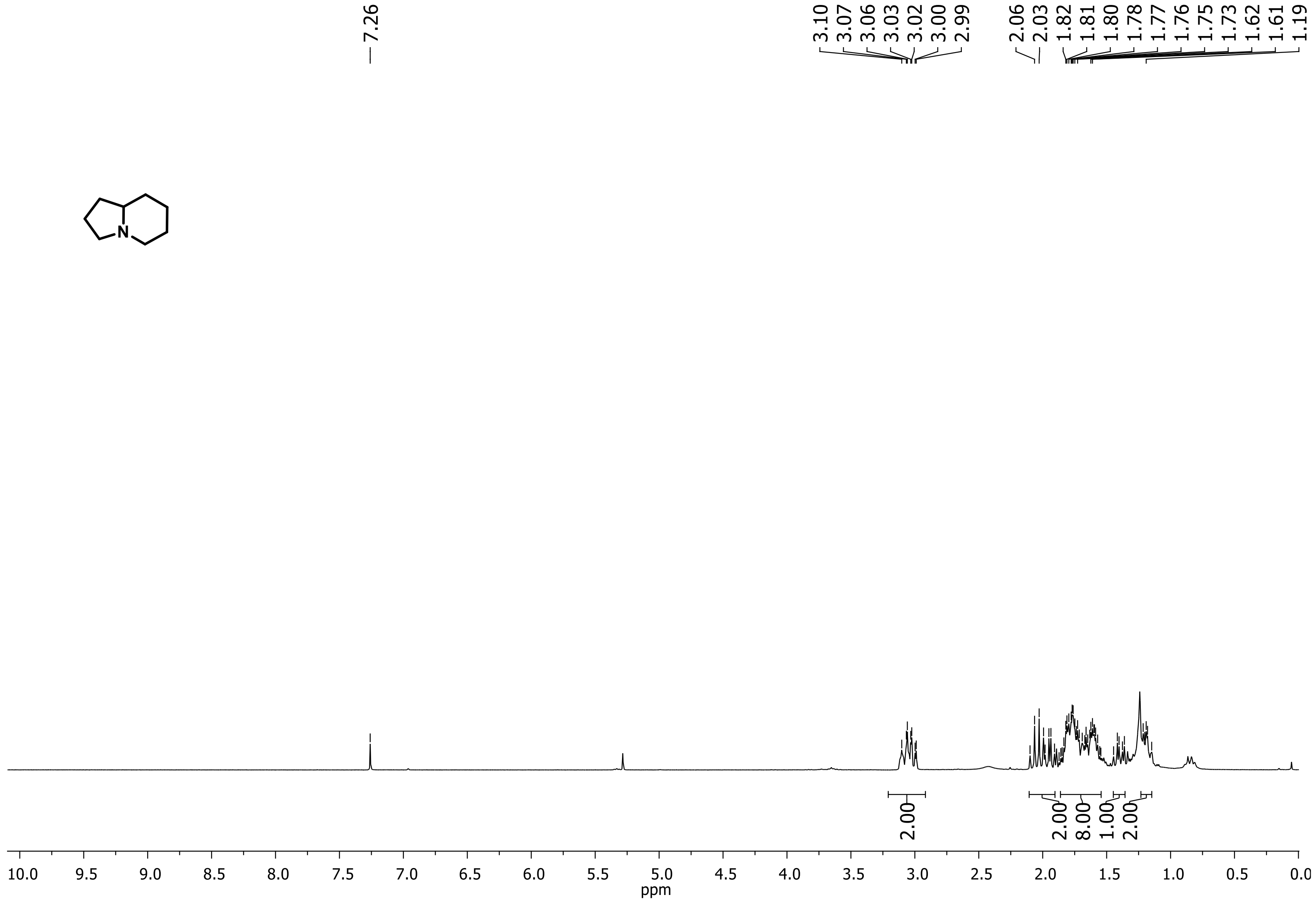

Molecule **13a**: $^{13}C\{^{1}H\}$ NMR (62.5 MHz, $CDCl_3$)

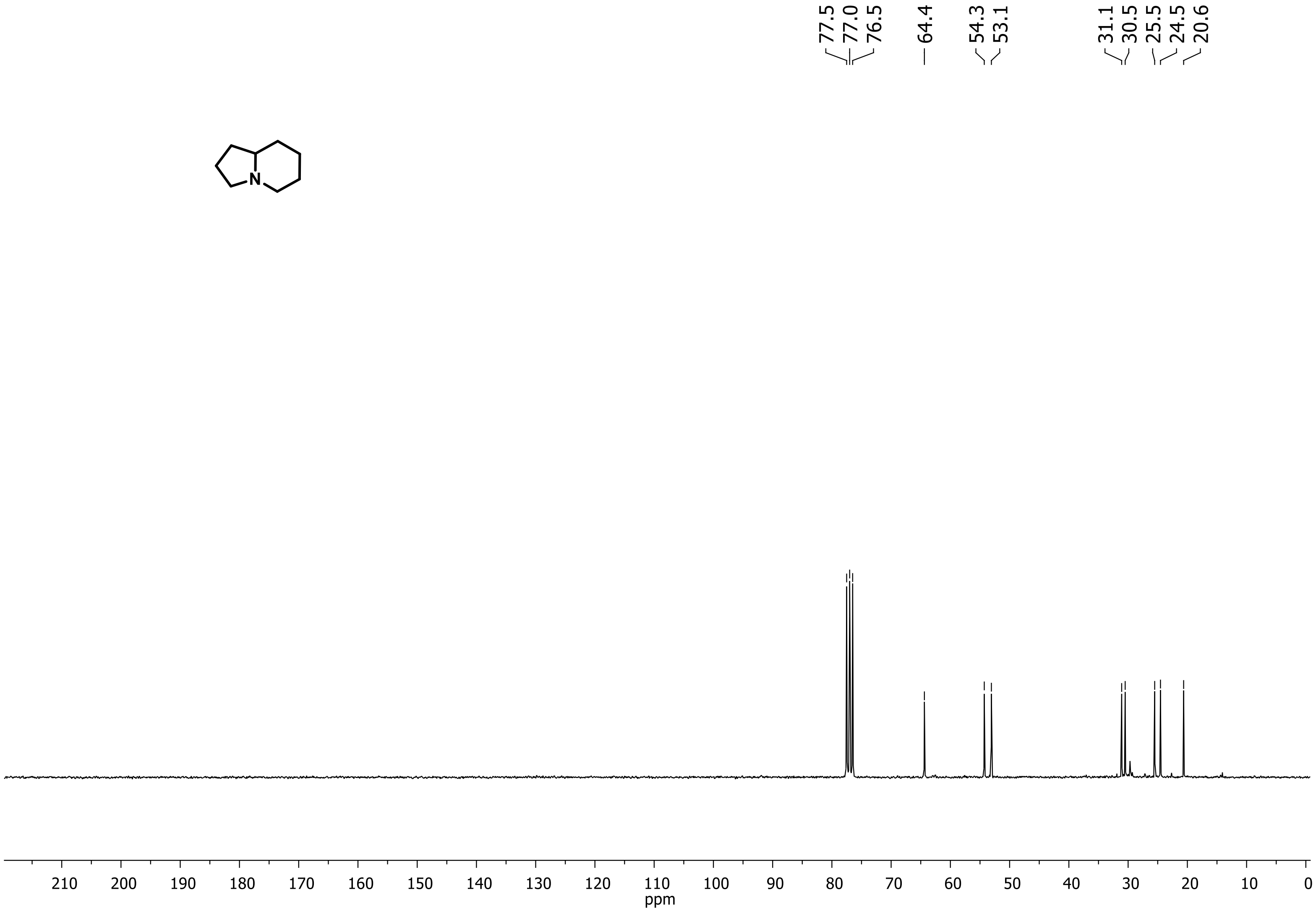

Molecule **12b**: ^{1}H NMR (400 MHz, $CDCl_3$)

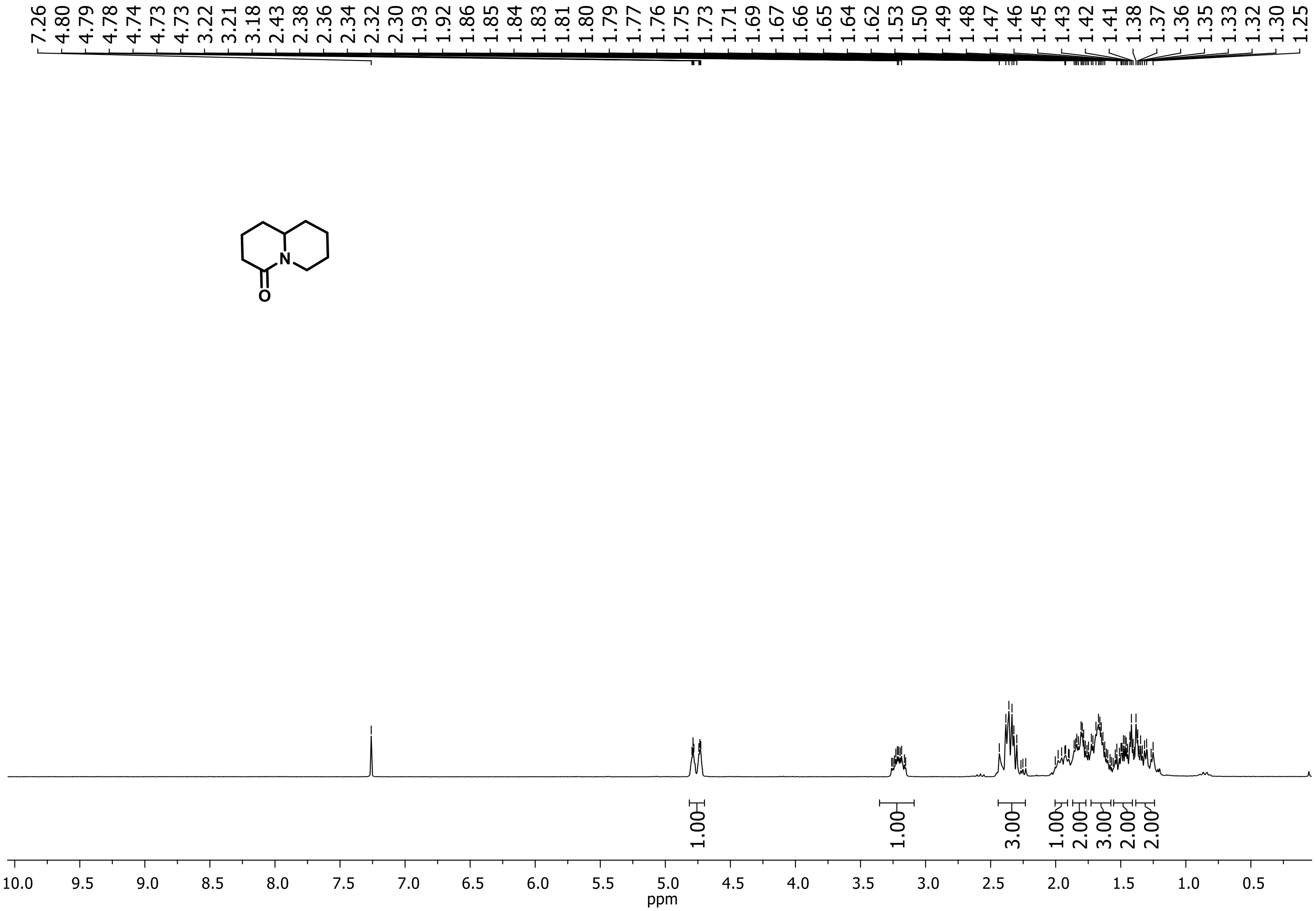

Molecule **12b**: $^{13}C\{^{1}H\}$ NMR (100 MHz, $CDCl_3$)

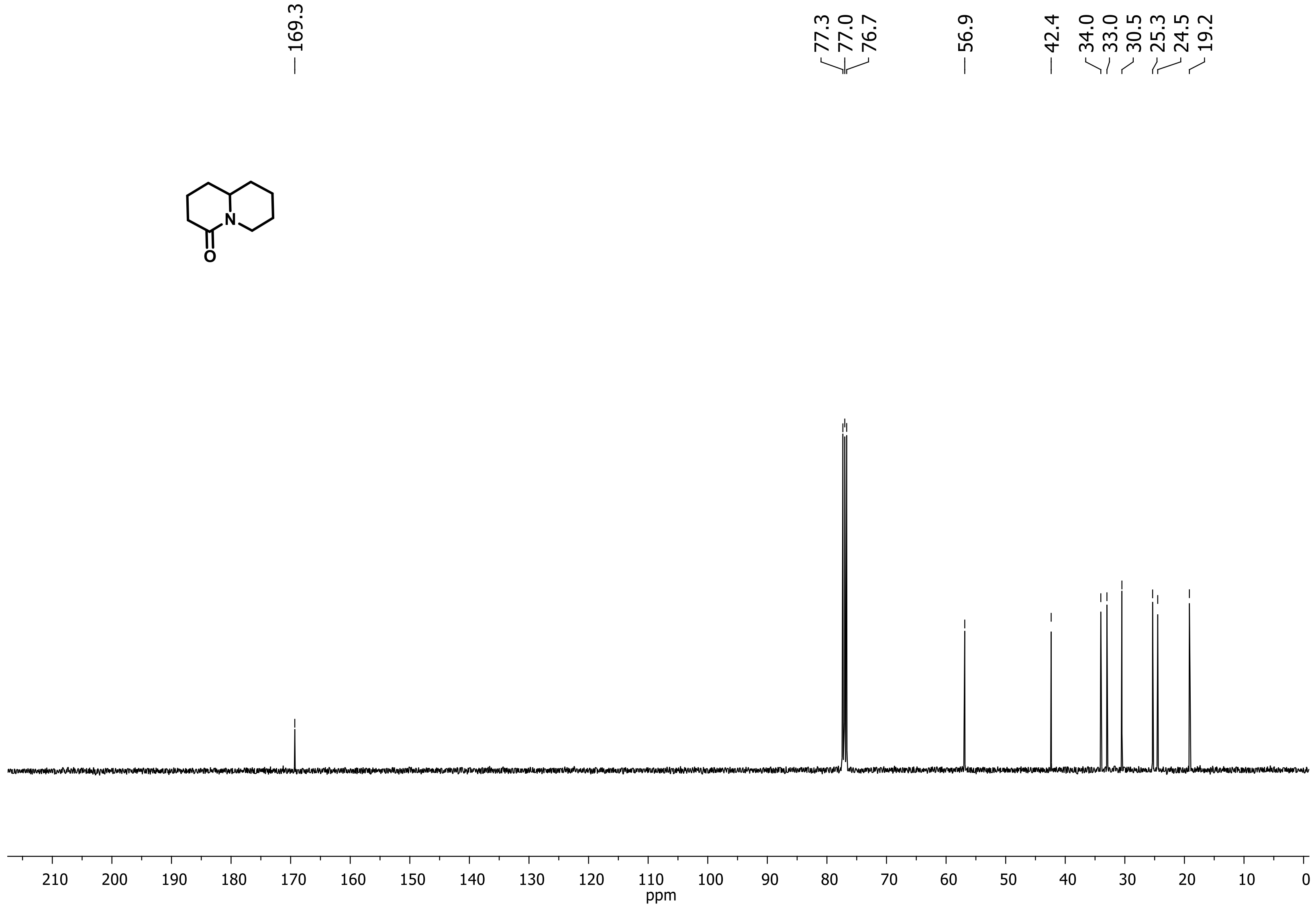

Molecule **12b'**: ^{1}H NMR (250 MHz, $CDCl_3$)

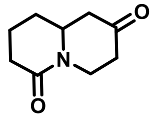

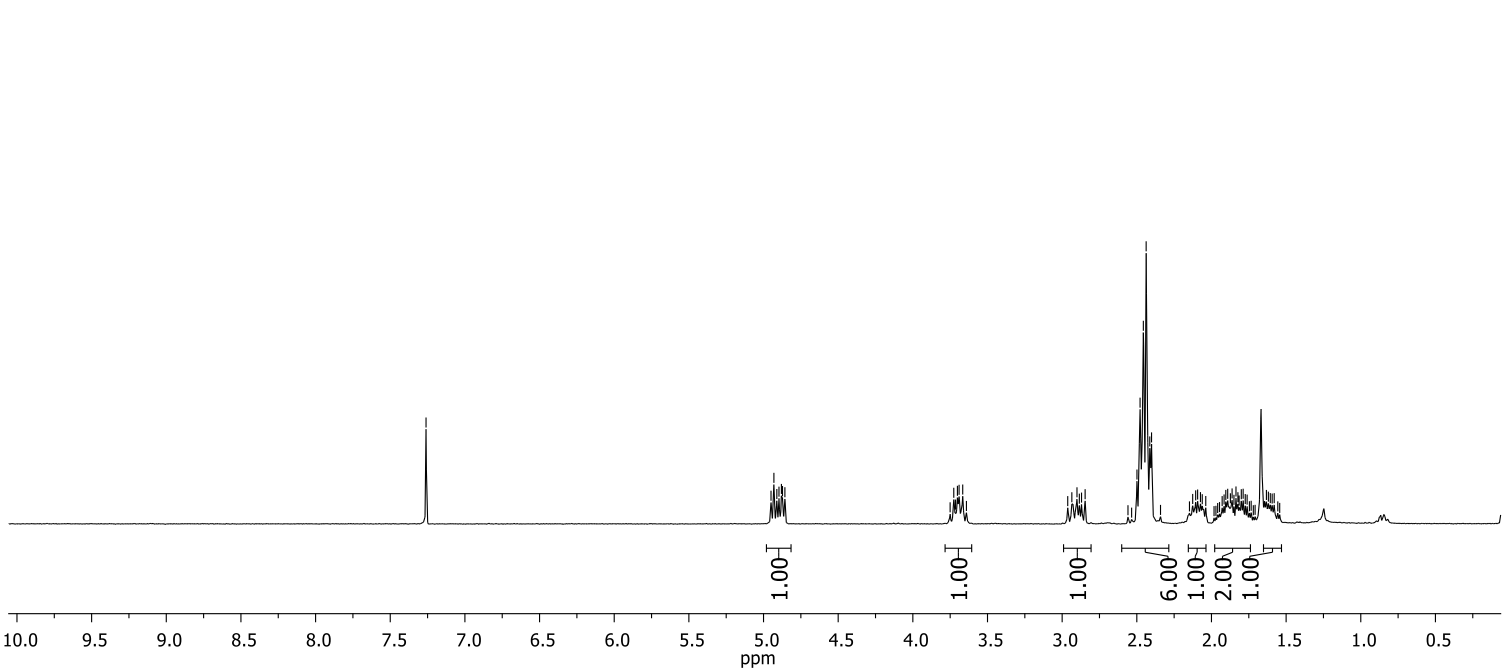

Molecule **12b'**: $^{13}C\{^1H\}$ NMR (62.5 MHz, $CDCl_3$)

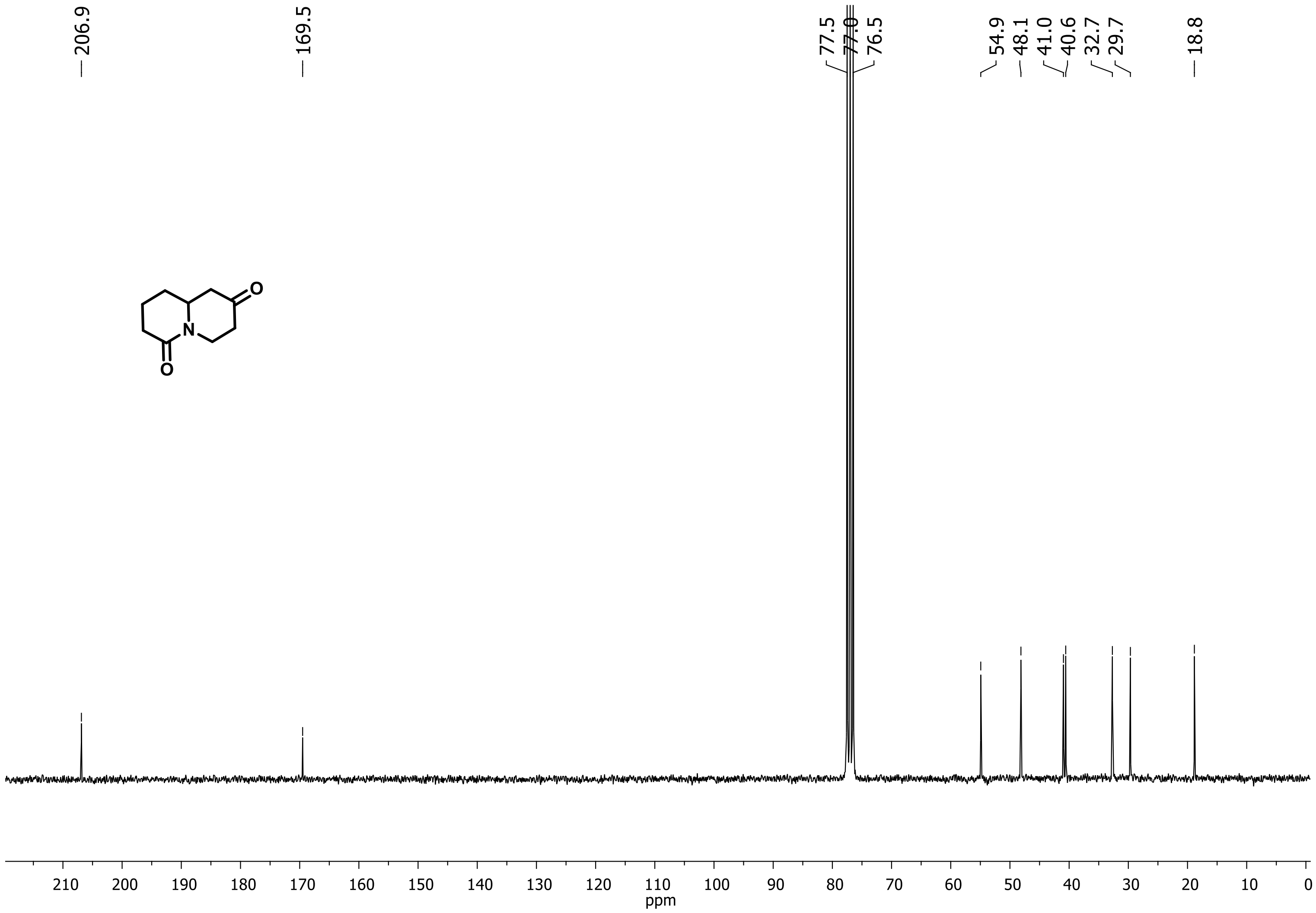

Molecule **13b**: ^{1}H NMR (250 MHz, $CDCl_3$)

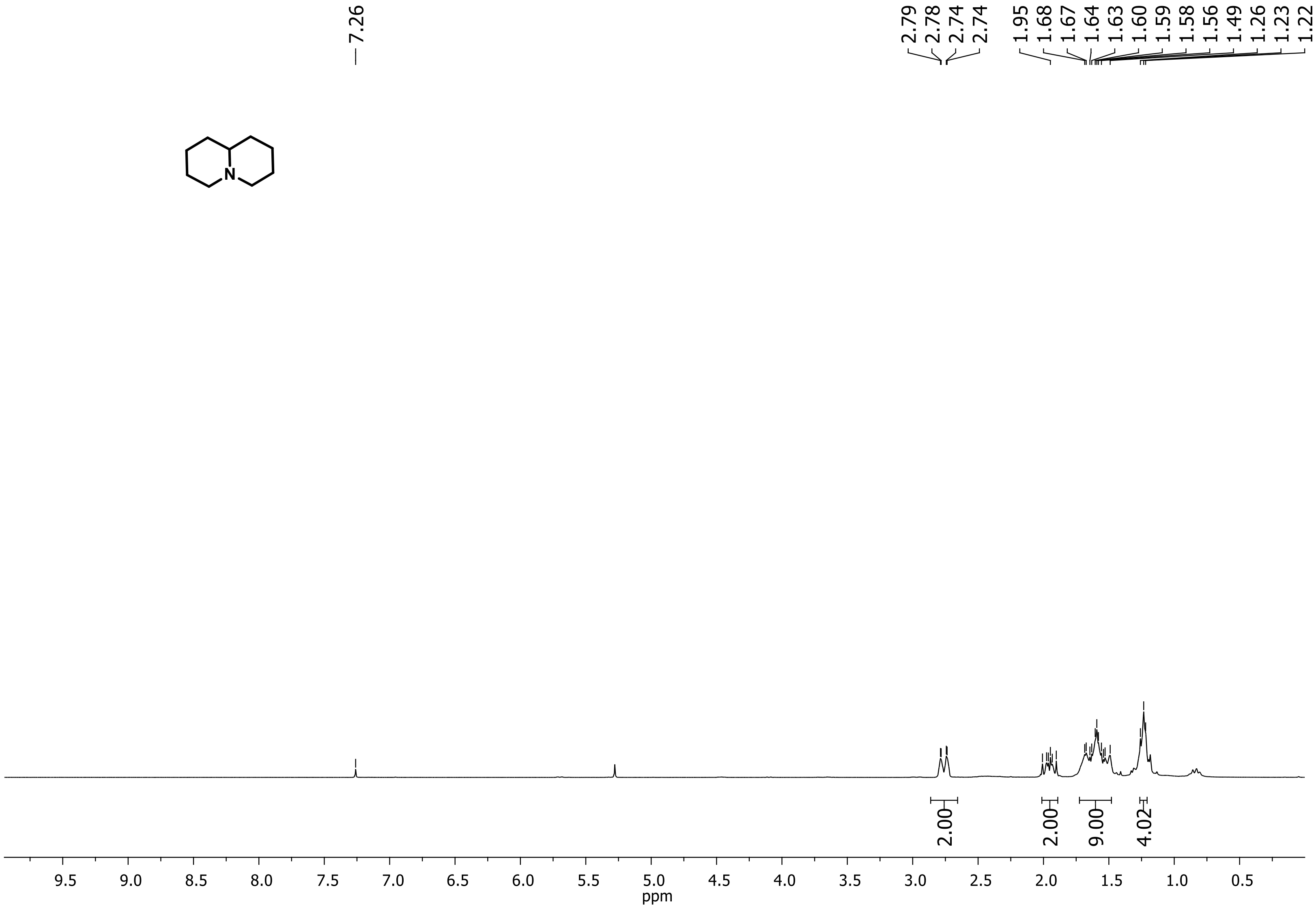

Molecule **13b**: $^{13}C\{^{1}H\}$ NMR (62.5 MHz, $CDCl_3$)

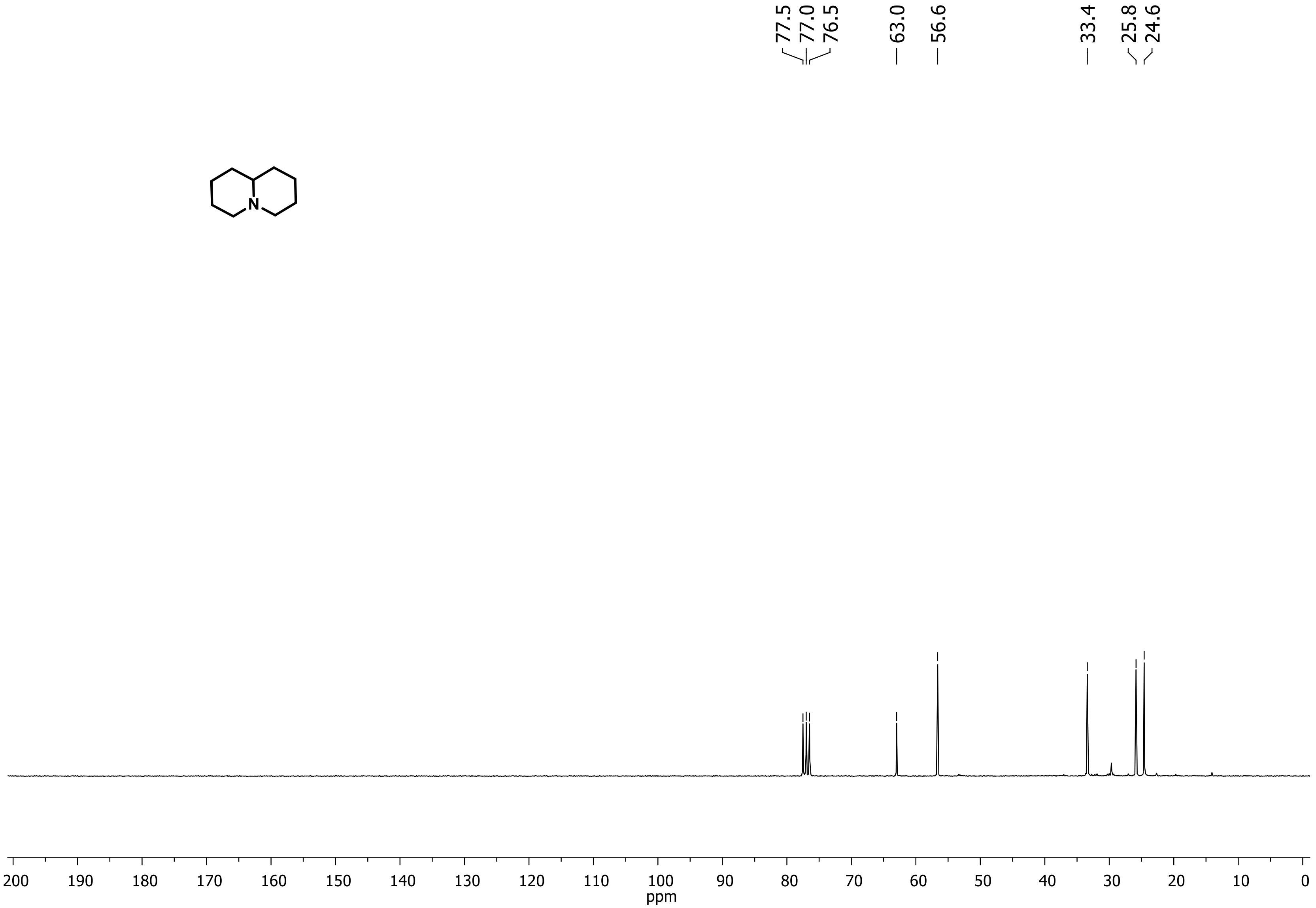

Molecule 12c: ^{1}H NMR (400 MHz, $CDCl_3$)

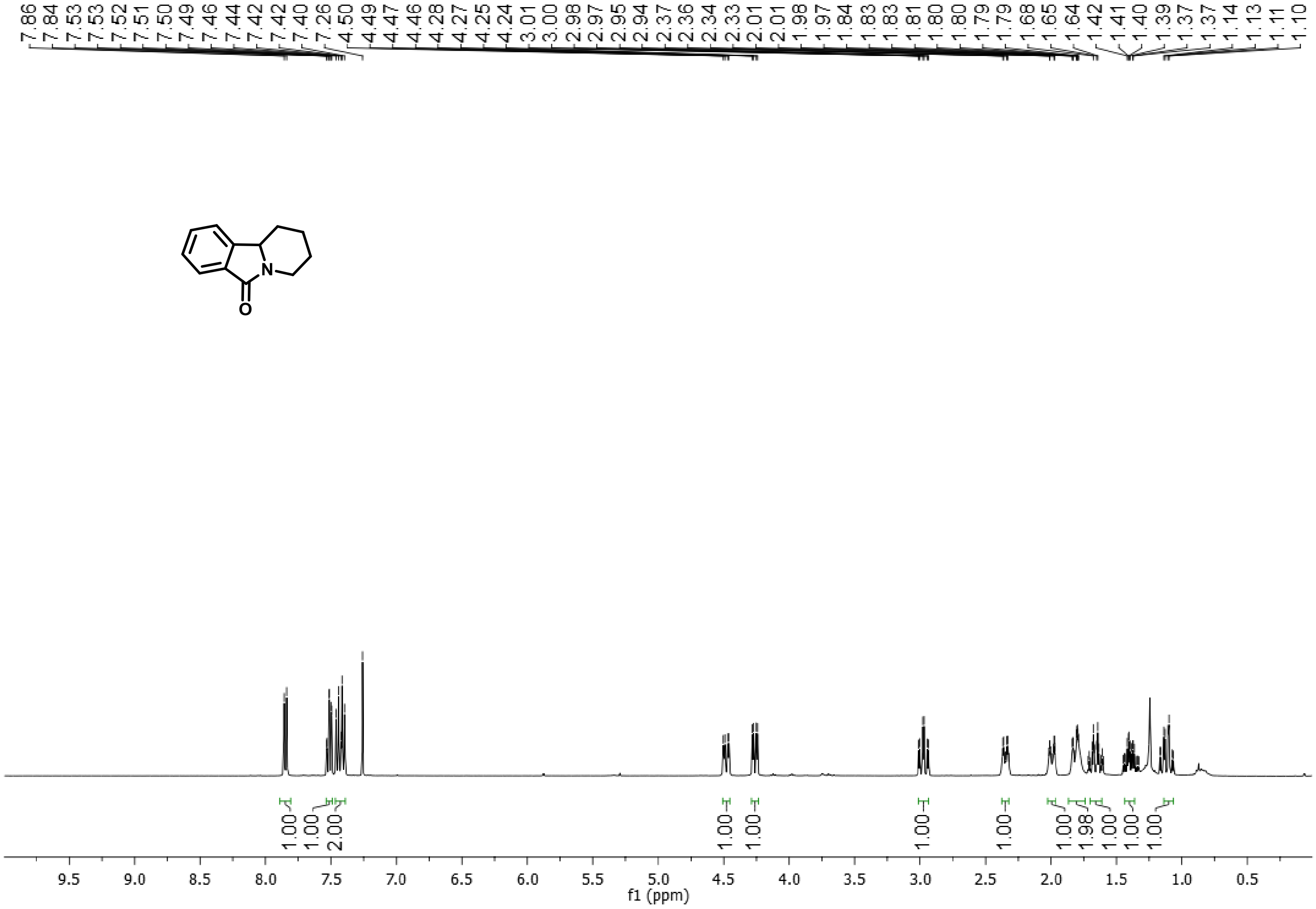

Molecule 12c: $^{13}C\{^{1}H\}$ NMR (100 MHz, $CDCl_3$)

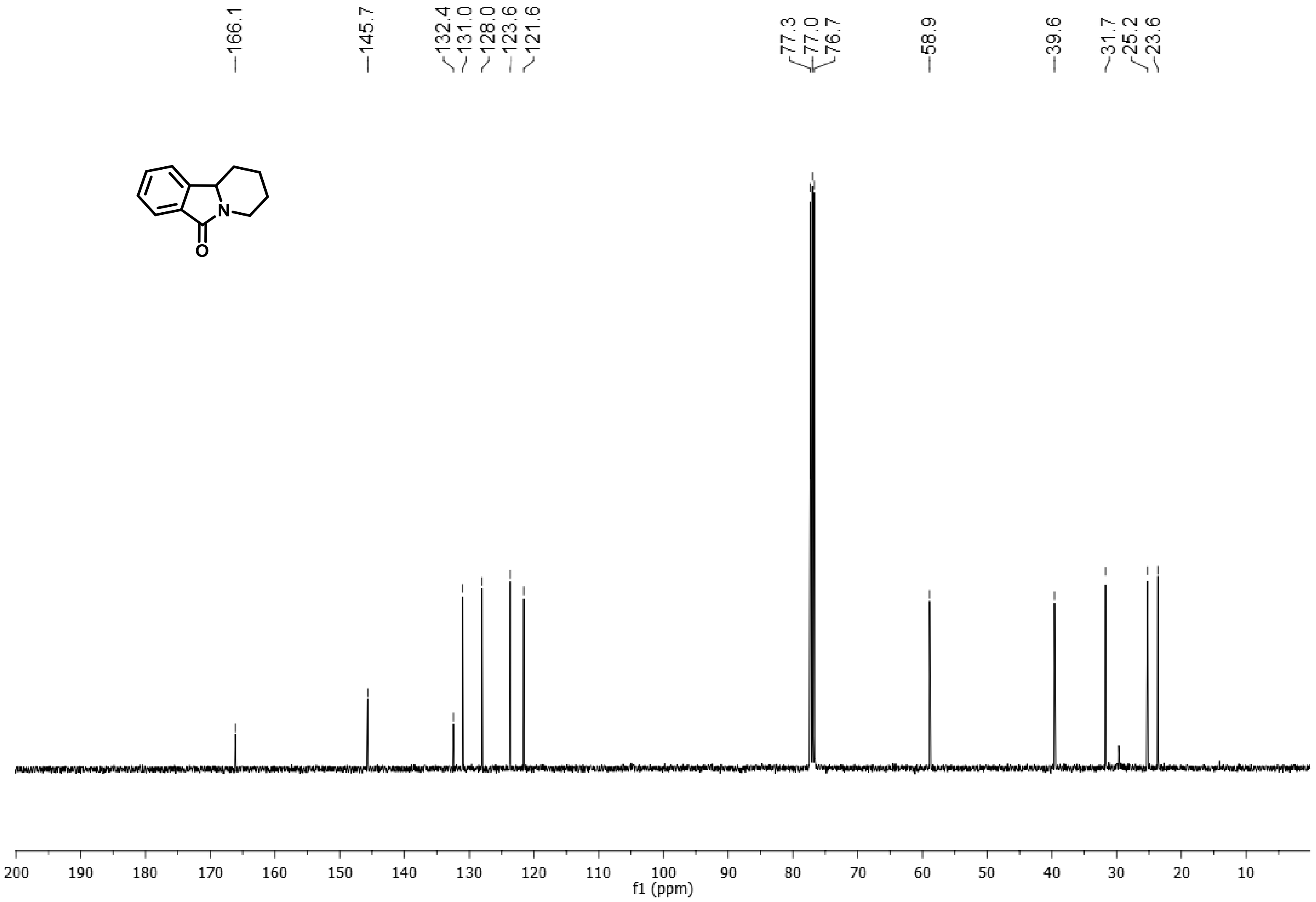

Supplement: Supplementary file 1 — ol3c02798_si_001.pdf [file ol3c02798_si_001.pdf]
